# Strategies to Deimplement Opioid Prescribing in Primary Care: A Cluster Randomized Clinical Trial

**DOI:** 10.1001/jamanetworkopen.2024.38325

**Published:** 2024-10-10

**Authors:** Andrew Quanbeck, James Robinson, Nora Jacobson, Xiang Li, Rose Hennessy-Garza, Jillian Landeck, Andrew Cohen, Lynn Madden, Alice Pulvermacher, Randall Brown

**Affiliations:** 1 Department of Family Medicine and Community Health, University of Wisconsin–Madison; 2 Forward Data Analytic Services, LLC, Verona, Wisconsin; 3 Institute for Clinical and Translational Research and School of Nursing, University of Wisconsin–Madison; 4 Zilber College of Public Health, University of Wisconsin–Milwaukee; 5 Bellin Health, Green Bay, Wisconsin; 6 APT Foundation, North Haven, Connecticut

## Abstract

**Importance:**

Centers for Disease Control and Prevention guidelines advocate reduced opioid prescribing for chronic pain, yet research on their implementation remains limited.

**Objective:**

To compare 4 deimplementation strategies to promote guideline-concordant opioid prescribing.

**Design, Setting, and Participants:**

This cluster randomized clinical trial was performed at 32 primary care clinics from 2 US health care systems from February 2020 to March 2022, using a hybrid type 3 sequential multiple-assignment design focused on patient outcomes. Clinics were recruited through volunteer sampling, including 268 clinicians and 8978 patients. Data were analyzed from September 2020 to March 2022.

**Intervention:**

Deimplementation strategies were targeted at the system, clinic, and prescriber levels. All clinics received a system-level strategy consisting of quarterly educational meetings with monthly audit and feedback (EMAF) reports. At month 3, half the clinics were randomized to receive practice facilitation (PF), a clinic-level strategy that targets clinic workflows. At month 9, half the clinics were again randomized to add prescriber peer consulting (PPC), a prescriber-level strategy focused on challenging patient cases.

**Main Outcomes and Measures:**

The primary outcome was change in mean morphine milligram equivalent (MME) dose in clinics receiving the least intensive bundle of deimplementation strategies (EMAF) vs the most intensive (EMAF plus PF plus PPC). Secondary outcomes included adherence to guideline metrics aimed at mitigating opioid risk.

**Results:**

Among the 8978 patients included in the analysis, 5142 (57.3%) were female; 42 (0.5%), American Indian or Alaska Native; 74 (0.8%), Asian or Pacific Islander; 411 (4.6%), Black; 187 (2.1%), Hispanic or Latino; 8127 (90.5%), White; and 137 (1.5%), other or unknown. Mean (SD) age was 58.3 (14.3) years. Eight clinics (including 66 prescibers and 2044 patients) assigned the most intensive strategy (EMAF plus PF plus PPC) had statistically significant effects on the primary outcome compared with 7 clinics (including 60 clinicians and 2427 patients) receiving the least intensive strategy (EMAF); clinics in the high-intensity group decreased the mean MME dose by 2.4 (95% CI, −4.3 to −0.5) mg/d more than the EMAF group (*P* = .02), representing a 6% reduction, and increased screening for pain severity, enjoyment of life, and general activity by 5.4% (95% CI, 0.4%-10.4% [*P* = .04]) more. Compared with EMAF, the most intensive strategy resulted in statistically significant decreases in urine drug screening (difference, −7.3% [95% CI, −11.5% to −3.0%]; *P* < .001) and use of treatment agreements (difference, −6.7% [95% CI, −11.1 to −2.3%]; *P* = .003), in the opposite direction of the hypothesis. There were no significant differences between groups in benzodiazepine coprescribing, mental health screening, or patients receiving an MME dose greater than or equal to 90.0 mg/d.

**Conclusions and Relevance:**

In this cluster randomized clinical trial, a high-intensity deimplementation strategy targeted at prescribers significantly decreased the MME dose and increased screening for pain intensity and pain-related interference while reducing use of treatment agreements and urine drug screening. Providing clinic- and prescriber-level deimplementation strategies may help health systems take positive steps toward reducing reliance on opioid medications for chronic pain management in primary care settings.

**Trial Registration:**

ClinicalTrials.gov Identifier: NCT04044521

## Key Points

**Question:**

What strategies are effective in deimplementing use of prescription opioids for chronic pain management in primary care settings?

**Findings:**

In this cluster randomized clinical trial including 8978 patients, clinics receiving clinic- and prescriber-level implementation strategies reduced their patients’ mean morphine equivalent daily dose by 2.4 units more than clinics receiving a less intensive health system–level strategy, representing a modest (6%) but statistically significant difference.

**Meaning:**

Providing clinic- and prescriber-level deimplementation strategies may help health systems take positive steps toward reducing reliance on opioid medications for chronic pain management in primary care settings.

## Teaser

This cluster randomized clinical trial tests a multilevel deimplementation strategy consisting of system-level educational meetings with audit and feedback reports, clinic-level practice facilitation, and prescriber peer consulting to treat patients prescribed opioids for pain management.

## Introduction

Berwick^1^ introduced dissemination and implementation research in health care in a 2003 *JAMA* article by highlighting the 264-year period between the vitamin C’s discovery as a cure for scurvy (in 1601) and its acceptance in general practice (1865). In modern health care, dissemination is often (but not always) slow; for example, prescription and dispensing of opioids in the US rose by 300% between 1991 and 2011, based on flimsy evidence,^2^ and was attended by an overdose and addiction crisis.^3^ In a step toward deimplementing opioids for chronic pain management, the Centers for Disease Control and Prevention (CDC) issued an opioid prescribing guideline in 2016 that included 12 recommendations for primary care clinicians to support clinical decision-making, improve communication between patients and clinicians, and ensure appropriate prescribing.^4,5^ The CDC updated and refined their guidelines in 2022 to include more specific recommendations for managing acute (duration, <1 month), subacute (duration, 1-3 months), and chronic (duration, >3 months) pain,^6^ based in part over concerns about overly rigid guideline implementation.

Many implementation strategies are available to support these efforts, including education, tracking systems, practice facilitation, and audit and feedback.^7^ For example, a systematic review^8^ showed that primary care clinics are 2.76 times more likely to adopt evidence-based guidelines through practice facilitation. The literature on deimplementation of harmful or ineffective interventions is mixed; a 2020 systematic review of deimplementation interventions that engage patients within patient-clinician interactions led to significant reductions in low-value care,^9^ while a 2021 scoping review identified 13 empirical deimplementation studies across health settings, with zero demonstrating effectiveness.^10^

Factors affecting deimplementation are multilevel, complex, and context specific.^11^ To address this gap, we tested a multilevel deimplementation strategy consisting of (1) system-level educational meetings with audit and feedback (EMAF) reports, (2) clinic-level practice facilitation (PF), and (3) prescriber peer consulting (PPC).^12^ The primary aim was to compare clinics that offered the most intensive sequence of strategies (EMAF plus PF plus PPC) with the clinics that were offered the least intensive strategy (EMAF) on change in clinic-level, mean morphine milligram equivalent (MME) daily dose over 18 months.

## Methods

### Trial Design

**Figure 1.**
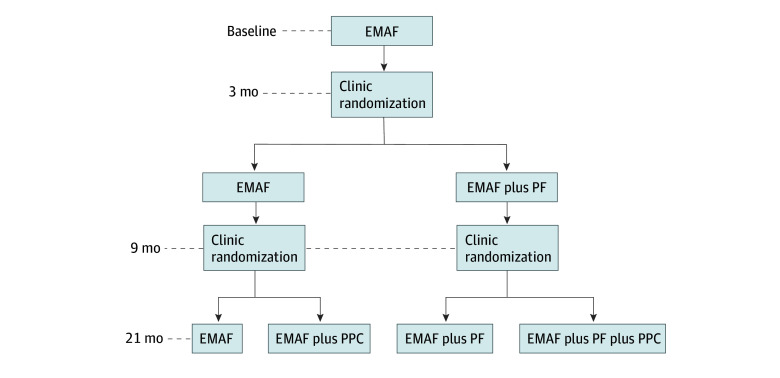
Study Design Diagram EMAF indicates educational meetings with audit and feedback; PF, practice facilitation; and PPC, prescriber peer consulting.

This cluster randomized clinical trial followed the Consolidated Standards of Reporting Trials (CONSORT) reporting guideline. The protocol was approved by the University of Wisconsin Health Sciences Institutional Review Board. Informed consent was obtained from clinic staff; a waiver was obtained for patients for use of deidentified data. The design was a hybrid type 3 effectiveness implementation, unrestricted, 2 × 2, clustered, sequential, multiple-assignment, randomized clinical trial.^13,14,15^ Clusters were defined as primary care clinics. In 2 randomization rounds, clinics were assigned to 4 intervention groups: EMAF, EMAF plus PF, EMAF plus PPC, or EMAF plus PF plus PPC (Figure 1).

All clinics received the EMAF intervention for the first 3 months. After 3 months, half the clinics were randomly assigned to receive the PF intervention for 18 months, and at 9 months, half the clinics were assigned to receive the PPC intervention for 12 months (in addition to previously assigned strategies). Clinics had an equal probability of being assigned to each implementation sequence. A 2020 protocol report provides detailed methodology.^12^

### Randomization

Randomization was stratified by health system, mean number of patients at the clinic prescribed opioids (above or below the median among all clinics), and mean MME dose over the first 3 months. The project statistician (J.R.) generated the random allocation sequence using a random number generator. Clinics were randomized in blocks of 4 in each stage. A study coordinator enrolled clinics and assigned them to groups.

### Participants

**Figure 2.**
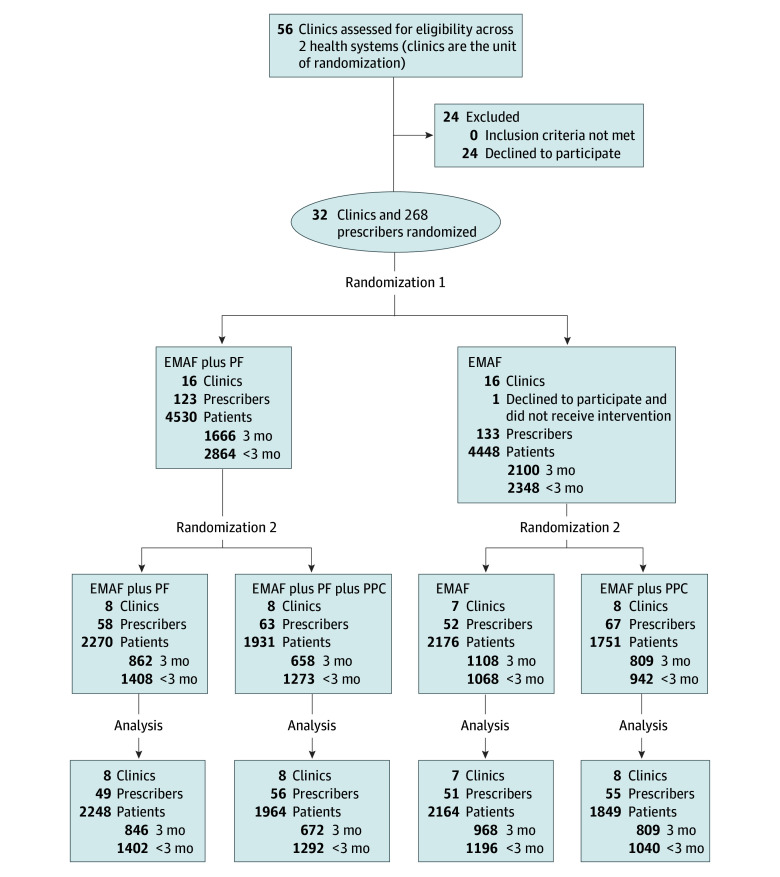
Participant Flow Diagram Due to the dynamic nature of the population, the number of patients and prescribers might vary at each stage. Counts reflect key points at first randomization, second randomization, and analysis, representing participants extracted by fixing the respective dates of these events. The number of prescribers represents the number with a patient receiving at least 1 opioid prescription in the prior 12 months at each time point. Numbers of patients indicate in scope patients over time, with subgroups for patients with opioid prescriptions in each of the most recent 3 months (3-month subgroup) vs patients with 0, 1, or 2 opioid prescriptions in the most recent 3 months (<3-month subgroup). EMAF indicates educational meetings with audit and feedback; PF, practice facilitation; and PPC, prescriber peer consulting.

Clinics were recruited from 2 health care systems from February 2020 to March 2022 in Wisconsin and Michigan representing multiple rural communities and 2 metropolitan areas. Eligible clinics (1) offered nonpediatric primary care, internal medicine, or family medicine; (2) did not receive the intervention prior to the study^16^; (3) did not prohibit opioid therapy; (4) had fewer than 80% of patients prescribed opioids with a treatment agreement (ie, a document jointly signed by the clinician and the patient prescribed an opioid medication outlining expectations and responsibilities) and urine drug screening in the last 12 months; and (5) had more than 10% of patients prescribed opioids receiving doses above the MME of 90 mg/d. Thirty-two clinics met eligibility criteria and participated; 24 declined participation (Figure 2).

While the intervention occurred at the clinic level, eligible prescribers were permanent primary care physicians or other clinicians with prescribing privileges. Prescription data for these clinicians were included for patients with at least 1 opioid prescription within the most recent 12 months documented in the electronic health record. Patients were excluded if they had a cancer diagnosis or were in hospice. Patient data on race and ethnicity were collected from electronic medical records, as required for National Institutes of Health–funded studies.

### Interventions

The interventions consisted of evidence-informed deimplementation strategies implemented at the system, clinic, and prescriber levels to increase adherence to CDC opioid prescribing guidelines while balancing opioid risks and benefits to manage chronic pain.^4,6,16,17,18^ At study onset and prior to randomization, each clinic’s medical director identified a change team leader and 3 to 7 clinic staff members to form a clinic-based implementation team that worked with implementation agents assigned by the research team to improve guideline concordance.

#### EMAF Intervention

The 6 quarterly meetings in the EMAF intervention were group-based, health system–level sessions focused on understanding guidelines for opioid prescribing targeted at implementation team members from all clinics. Except for the first in-person meeting, educational meetings were conducted synchronously online or watched via recording. Each clinic received monthly feedback reports on opioid prescribing metrics reflecting CDC guidelines aggregated at the clinician level. Metrics included (1) mean MME, (2) patients prescribed doses of more than an MME of 90 mg/d, (3) patients coprescribed benzodiazepines, (4) annual treatment agreements, (5) annual urine drug screens, (6) patients screened for depression with the Patient Health Questionnaire–2 (PHQ-2),^19^ and (7) patients screened for pain severity and functional impact with the PEG-3 (Pain, Enjoyment, General Activity) tool, a validated scale for assessing pain intensity and interference.^20^ Combined, EMAF constituted the least intensive intervention.

#### PF Intervention

The PF intervention designated an external change agent who worked with clinic implementation teams to introduce system engineering tools (eg, Plan-Do-Check-Act cycles) to monitor progress toward goals.^21^ The work was completed in 6 monthly meetings and 4 quarterly meetings with a practice facilitator via videoconferencing. Half the clinics were randomized at month 3 to receive the EMAF plus PF intervention.

#### PPC Intervention

The PPC intervention was offered by physician-trained external change agents with expertise in chronic pain and/or opioid use disorder treatment during 4 quarterly videoconferencing sessions with clinic prescribers, focused on addressing challenging patient cases. Half the clinics were randomized to receive PPC at month 9.

### Sample Size

The total sample size was based on the mean difference on change in MME from intervention months 3 to 21 between the lowest-dose implementation sequence (EMAF) vs the highest-dose sequence (EMAF plus PF plus PPC). Based on pilot data from a preceding trial,^16^ the intraclass correlation coefficient was estimated to be ρ = 0.14. Assuming a mean of 6 prescribers per clinic, a type I error rate of α = 5%, and ρ = 0.14, 256 prescribers would provide at least 80% power to detect a moderate effect size of Cohen *d* = 0.67 between the 2 implementation sequences on change in MME.

### Outcomes

The primary outcome was prescriber mean MME daily dose per patient receiving opioids, calculated over a 3-month period. These data were extracted from electronic health records to ease data collection burden and eliminate missing data. The primary outcome compared the mean difference on the change in MME from intervention months 3 to 21 between EMAF vs EMAF plus PF plus PPC.

Secondary outcome analyses included pairwise comparisons in MME between the other implementation sequences: EMAF vs EMAF plus PF; EMAF vs EMAF plus PPC; EMAF plus PF vs EMAF plus PPC; EMAF plus PF vs EMAF plus PF plus PPC; and EMAF plus PPC vs EMAF plus PF plus PPC. Secondary outcomes included coprescribing with benzodiazepines, annual urine drug screens, annual treatment agreements, prevalence of patients with MME of greater than or equal to 90 mg/d, and patient screening with the PHQ-2 and PEG-3. Other prespecified secondary outcomes (eg, qualitative and economic) will be published separately.^12^

### Statistical Analysis

Data were analyzed from September 2020 to March 2022. With the intention-to-treat approach, we used a longitudinal (repeated-measures) analysis for each primary and secondary outcome. Data were collected at intervention month 3 (immediately prior to randomization, September 2020, after a 4-month COVID-19 delay) and every month up to intervention month 21 (March 2022). Mean outcomes were calculated over 3 months; thus, there were 7 nonoverlapping measurement occasions. This was a 3-level analysis with repeated measures at the patient, prescriber, and clinic levels. A piecewise linear model with a knot at intervention month 9 (immediately before the second randomization) was used to model temporal trajectories in outcomes over months 4 to 21. Aggregated *P* values were used as a method for multiple-comparison correction. The complete primary aim analysis protocol was published as a supplementary appendix to the 2020 trial protocol.^12^ Supplement 1 provides the trial protocol and eMethods in Supplement 2 has a detailed specification of the analytic process.

The 2022 CDC guidelines provide distinct recommendations for acute and subacute vs chronic pain, with a 3-month threshold marking a transition to chronic pain.^5^ We ran an exploratory patient subgroup analysis to examine effects on patients with longer vs shorter periods of opioid use. Our study’s 3-month long-term or “chronic” opioid user group includes patients with opioid prescriptions overlapping each of the most recent 3 calendar months, whereas the group with less than 3 months includes patients with at least 1 opioid prescription in the prior 12 months but less than 3 prescriptions in the most recent 3 months. Due to the model structure, our definitions for less than 3 months or for 3 months do not match CDC definitions for chronic vs acute and subacute use exactly; however, they provide potentially useful proxy measures for chronic vs acute and subacute opioid use.

The study’s data safety and monitoring committee reported no harms. We ran a safety check to evaluate the number of clinicians in each group for whom opioid prescribing dropped by more than 20%.^22,23^

Two-sided *P* < .05 indicated statistical significance. Analyses were performed using SAS, version 9.4 (SAS Institute Inc).

## Results

### Baseline Data

Of the 8978 patients included in the analysis, 5142 (57.3%) were female and 3836 (42.7%) were male. Race and ethnicity obtained from electronic health records included 42 (0.5%) American Indian or Alaska Native individuals, 74 (0.8%) Asian or Pacific Islander individuals, 411 (4.6%) Black individuals, 187 (2.1%) Hispanic or Latino individuals, 8127 (90.5%) White individuals, and 137 (1.5%) individuals with unreported or unknown race or ethnicity. Mean (SD) age was 58.3 (14.3) years. Mean (SD) MME prescribed for the last quarter at baseline was 38.7 (12.4) mg/d across all groups, ranging from 35.0 (10.1) mg/d in the EMAF plus PF plus PPC group to 41.8 (13.2) mg/d in the EMAF plus PPC group. Percentages in compliance with CDC guidelines for coprescribing with benzodiazepines, annual drug urine screening, presence of a treatment agreement, and completion of PHQ-2 and PEG-3 at baseline are presented in Table 1.

**Table 1.** Baseline Outcomes by Study Arm for All Patients

Arm	Intervention group, No. (%)^a^				
	EMAF (n = 2427)	EMAF plus PPC (n = 2021)	EMAF plus PF (n = 2486)	EMAF plus PF plus PPC (n = 2044)ᵇ	Total (N = 8978)
Age, mean (SD), y	58.1 (14.1)	58.6 (14.1)	58.8 (14.3)	57.6 (14.5)	58.3 (14.3)
Sex					
Female	1366 (56.3)	1192 (59.0)	1450 (58.3)	1134 (55.5)	5142 (57.3v
Male	1061 (43.7)	829 (41.0)	1036 (41.7)	910 (44.5)	3836 ( 42.7)
Race and ethnicity^b^					
American Indian or Alaska Native	11 (0.5)	14 (0.7)	10 (0.4)	7 (0.3)	42 (0.5)
Asian or Pacific Islander	19 (0.8)	27 (1.3)	18 (0.7)	10 (0.5)	74 (0.8)
Black	112 (4.6)	179 (8.9)	77 (3.1)	43 (2.1)	411 (4.6)
Hispanic or Latino	36 (1.5)	64 (3.2)	57 (2.3)	30 (1.5)	187 (2.1)
White	2207 (91.0)	1708 (84.5)	2297 (92.4)	1915 (93.7)	8127 (90.5)
Unreported or unknown	42 (1.7)	29 (1.4)	27 (1.1)	39 (1.9)	137 (1.5)
MME prescribed in last quarter, mg/d^c^	40.7 (14.6)	41.8 (13.2)	37.4 (9.8)	35.0 (10.1)	38.7 (12.4)
MME ≥90 mg/d^c^	9.7 (7.8)	10.3 (7.5)	8.4 (5.2)	6.3 (5.8)	8.7 (6.8)
Benzodiazepines in last quarter^c^	12.5 (6.3)	9.8 (7.1)	9.4 (5.3)	9.2 (6.4)	10.3 (6.4)
Urine screening in last year^c^	19.0 (16.1)	18.0 (14.7)	12.7 (13.5)	12.0 (10.1)	15.4 (14.2)
Treatment agreement in last year^c^	12.0 (13.5)	7.8 (10.0)	7.6 (12.5)	10.3 (8.8)	9.5 (11.6)
PHQ-2 screening in last year^c^	25.0 (14.6)	22.6 (11.8)	23.5 (13.2)	27.7 (17.3)	24.7 (14.5)
PEG-3 screening in last year^c^	7.3 (12.2)	5.1 (10.5)	1.6 (3.6)	0.4 (1.2)	3.7 (8.8)

Abbreviations: EMAF, educational meetings with audit and feedback; MME, morphine milligram equivalent daily dose; PEG-3, Pain, Enjoyment, General Activity; PF, practice facilitation; PHQ-2, Patient Health Questionnaire–2; PPC, prescriber peer consulting.

^a^ Summarizes baseline outcome levels observed in the last calendar month prior to the first randomization. Includes all patients with at least 1 opioid order in the last 12 months. Demographic data represent patients assigned to the prescribers, not the prescribers themselves.

^b^ Obtained from electronic health records.

^c^ Indicates mean baseline outcomes at the prescriber level, weighted by the number of patients receiving opioids associated with each prescriber. Values in parentheses represent the weighted SD.

### Outcomes and Estimation

Seven outcomes were measured for the total patient population. Data for all of these outcomes are shown in Table 2.

**Table 2.** Estimated Impact of Study Interventions for All Patients Prescribed Opioids^a^

	EMAF 18-mo change	EMAF plus PF 18-mo change	EMAF plus PF vs EMAF	EMAF plus PPC 18-mo change	EMAF plus PPC vs EMAF	EMAF plus PF vs EMAF plus PPC	EMAF plus PF plus PPC 18-mo change	EMAF plus PF plus PPC vs EMAF	PF × PPC interaction^b^
**Mean MME** ^c^									
Estimate, mg/d (95% CI)	−1.1 (−2.5 to 0.3)	−2.4 (−3.8 to −1.0)	−1.3 (−3.2 to 0.6)	−2.7 (−4.2 to −1.3)	−1.6 (−3.5 to 0.2)	0.3 (−1.6 to 2.3)	−3.5 (−4.9 to −2.1)	−2.4 (−4.3 to −0.5)	0.5 (−2.0 to 3.1)
Cohen *d*	−0.05	−0.11	−0.06	−0.12	−0.07	0.01	−0.15	−0.47	0.02
*P* value	.13	<.001	.18	<.001	.08	.74	<.001	.02	.68
**MME ≥90 mg/d**									
Estimate, mg/d (95% CI)	−0.9 (−2.0 to 0.2)	−1.2 (−2.3 to −0.2)	−0.4 (−1.8 to 1.1)	−2.0 (−3.2 to 0.9)	−1.1 (−2.6 to 0.3)	0.8 (−0.7 to 2.2)	−1.4 (−2.6 to −0.3)	−0.6 (−2.1 to .0.9)	1.0 (−1.0 to 3.0)
Cohen *d*	−0.07	−0.10	−0.03	−0.15	−0.09	0.06	−0.11	−0.04	0.07
*P* value	.12	.02	.60	<.001	.13	.32	.01	.45	.36
**Benzodiazapine use**									
Estimate, % (95% CI)	−0.8 (−2.0 to 0.4)	−0.6 (−1.8 to 0.6)	−0.2 (−1.5 to 1.9)	−0.9 (−2.2 to 0.4)	−0.1 (−1.7 to 1.6)	0.3 (−1.4 to 2.0)	−0.2 (−1.4 to 1.1)	0.6 (−1.1 to 2.3)	0.5 (−1.8 to 2.8)
Cohen *d*	−0.06	−0.05	0.02	−0.07	−0.01	0.02	−0.01	0.05	0.04
*P* value	.18	.31	.82	.16	.91	.74	.77	.46	.65
**Urine screen**									
Estimate, % (95% CI)	6.8 (3.8 to 9.9)	0.5 (−2.5 to 3.6)	−6.3 (−10.6 to −2.1)	4.2 (1.2 to 7.2)	−2.7 (−6.6 to 1.3)	−3.7 (−7.9 to 0.6)	−0.4 (−3.4 to 2.6)	−7.3 (−11.5 to −3.4)	1.7 (−3.8 to 7.2)
Cohen *d*	0.45	0.04	−0.42	0.28	−0.18	−0.24	−0.03	−0.48	0.11
*P* value	<.001	.73	.004	.006	.18	.09	.78	<.001	.55
**Treatment agreement**									
Estimate, % (95% CI)	7.7 (4.5 to 10.9)	4.6 (1.4 to 7.8)	−3.1 (−7.5 to 1.3)	1.7 (−1.5 to 4.8)	−6.1 (−9.6 to −2.3)	2.9 (−1.4 to 7.3)	1.1 (−2.1 to 4.3)	−6.7 (−11.1 to −2.3)	2.5 (−2.9 to 7.9)
Cohen *d*	0.51	0.30	−0.21	0.11	−0.40	0.19	0.07	−0.44	0.17
*P* value	<.001	.005	.17	.30	.002	.19	.51	.003	.36
**PHQ-2 screen**									
Estimate, % (95% CI)	13.6 (9.4 to 17.8)	11.8 (7.6 to 15.9)	−1.9 (−7.7 to 4.0)	5.4 (1.3 to 9.5)	−8.2 (−13.2 to −3.2)	6.3 (0.6 to 12.1)	13.5 (9.4 to 17.7)	−0.1 (−5.9 to 5.7)	10.0 (2.9 to 17.1)
Cohen *d*	0.62	0.53	−0.08	0.25	−0.37	0.29	0.61	0	0.45
*P* value	<.001	<.001	.53	.01	.001	.03	<.001	.98	.006
**PEG-3 screen**									
Estimate, % (95% CI)	2.0 (−1.6 to 5.6)	12.3 (8.7 to 15.9)	10.2 (5.2 to 15.3)	−0.1 (−3.6 to 3.5)	−2.1 (−5.8 to 1.6)	12.3 (7.3 to 17.3)	7.4 (3.9 to 11.0)	5.4 (0.4 to 10.4)	−2.7 (−7.9 to 2.5)
Cohen *d*	0.14	0.84	0.70	0	−0.14	0.85	0.51	0.37	−0.19
*P* value	.27	<.001	<.001	.98	.27	<.001	<.001	.04	.31
**Aggregated *P* value^d^**	<.001	<.001	<.001	<.001	<.001	<.001	<.001	<.001	.14

Abbreviations: EMAF, educational meetings with audit and feedback; MME, morphine milligram equivalent daily dose; PEG-3, Pain, Enjoyment, General Activity; PF, practice facilitation; PHQ-2, Patient Health Questionnaire–2; PPC, prescriber peer consulting.

^a^ Values were estimated using a linear mixed-effects model with the restricted maximum likelihood method. This approach was applied to assess the change in the fixed-effect curve from baseline to the end of the intervention period (18 months), within and among the 4 study arms.

^b^ Focuses on the magnitude and statistical significance of the model interaction term. If this term can be taken to be zero, the 2 intervention effects are considered additive.

^c^ Percentages of prescribers who reduced MME more than 20% were 13.3% (8 of 60) for the EMAF group; 7.7% (5 of 65) for the EMAF plus PF group, 16.9% (13 of 77) for the EMAF plus PPC group, and 21.20% (14 of 66) for the EMAF plus PF plus PPC group.

^d^ We used the geometric mean of *P* values to calculate the aggregated *P* values, which can be used as a method for multiple-comparison correction to assess the null hypothesis that the true changes (or differences in changes) for all 5 of the outcomes are uniformly zero.

#### Mean MME

There was a negative, but not statistically significant, change in mean MME from baseline to the end of intervention for the EMAF group (−1.1 [95% CI, −2.5 to 0.3] mg/d; *P* = .13). Among the 3 more intensive groups, EMAF plus PF (−2.4 [95% CI, −3.8 to −1.0] mg/d), EMAF plus PPC (−2.7 [95% CI, −4.2 to −1.3] mg/d), and EMAF plus PF plus PPC (−3.5 [95% CI, −4.9 to −2.1] mg/d) had statistically significant reductions in mean MME (*P* < .001). The difference between the EMAF plus PF plus PPC and the EMAF reductions was statistically significant (−2.4 [95% CI, −4.3 to −0.5] mg/d; *P* = .02) in the direction of our hypothesis, representing a 6% reduction. There were no significant interaction effects between the PF and PPC interventions.

#### Patients With MME of Greater Than or Equal to 90 mg/d

There was a negative, but not statistically significant, change in the percentage of patients with a mean MME greater than or equal to 90 mg/d from baseline to end of intervention for EMAF (−0.9% [95% CI, −2.0% to 0.2%]). The 3 other groups had statistically significant reductions in percent of patients with an MME of greater than or equal to 90 mg/d: EMAF plus PF (−1.2% [95% CI, −2.3% to −0.2%]), EMAF plus PPC (−2.0% [95% CI, −3.2% to −0.9%]), and EMAF plus PF plus PPC (−1.4% [95% CI, −2.6% to −0.3%]) (*P* ≤ .02). There were no significant differences in this variable between any of the groups.

#### Patients Coprescribed Benzodiazepines in Prior 3 Months

There were estimated reductions for all 4 groups among patients coprescribed benzodiazepines in the prior 3 months but no statistically significant reductions or pairwise differences between groups. The group with the greatest intensity (EMAF plus PF plus PPC) had a change of −0.2% (95% CI, −1.4% to 1.1% [*P* = .77]).

#### Patients With Urine Drug Screening in Prior 12 Months

The EMAF group and EMAF plus PPC both had significant increases in urine drug screening rates of 6.8% (3.8% to 9.9% [*P* < .001]) and 4.2% (95% CI, 1.2% to 7.2% [*P* = .006]), respectively. The difference between these 2 groups was negative (−2.7% [95% CI, −6.6% to 1.3%]), but not statistically significant (*P* = .18). The difference between EMAF plus PF vs EMAF was negative (−6.3% [95% CI, −10.6% to −2.1%]) and significant (*P* = .004). Similarly, the difference between EMAF plus PF plus PPC and EMAF was negative (−7.3% [95% CI, −11.5% to −3.4%]) and significant (*P* < .001). In other words, these 2 higher-intensity groups had lower rates of urine drug screening than the low-intensity group (EMAF), in the opposite direction of our hypothesis.

#### Patients With Treatment Agreement in Prior 12 Months

The EMAF and EMAF plus PF interventions both had significant increases in treatment agreement rates of 7.7% (95% CI, 4.5%-10.9%) and 4.6% (95% CI, 1.4%-7.8%), respectively (*P* ≤ .005). The difference between these 2 groups was negative, but not statistically significant. The remaining 2 groups had no significant change. However, the pairwise differences between the EMAF plus PPC vs EMAF (−6.1% [95% CI, −9.6% to −2.3%]) and EMAF plus PF plus PPC vs EMAF (−6.7% [95% CI, −11.1% to −2.3%]) were negative and statistically significant in the opposite direction hypothesized (*P* ≤ .003).

#### Patients With PHQ-2 Depression Screening in Prior 12 Months

All study groups had significant increases in depression screening rates (EMAF, 13.6% [95% CI, 9.4%-17.8%]; EMAF plus PF, 11.8% [95% CI, 7.6%-15.9%]; EMAF plus PPC, 5.4% [95% CI, 1.3%-9.5%]; and EMAF plus PF plus PPC, 13.5% [95% CI, 9.4%-17.7%]). The difference between EMAF plus PPC and EMAF was negative (−8.2% [95% CI, −13.2% to −3.2%]) and statistically significant (*P* = .001), in the opposite direction hypothesized. There was a significant interaction effect between the PF and PPC groups.

#### Patients With PEG-3 Screening in Prior 12 Months

The EMAF plus PF and EMAF plus PF plus PPC interventions had significant improvements in PEG-3 screening rates (12.3% [8.7% to 15.9%] and 7.4% [95% CI, 3.9%-11.0%], respectively; *P* < .001 for both); no significant change was observed in the other 2 groups. Both improvements were significantly greater than that for EMAF (in the hypothesized direction) at 10.2% (95% CI, 5.2%-15.3% [*P* < .001]) and 5.4% (95% CI, 0.4%-10.4% [*P* = .04]), respectively.

### Patient Subgroup Analysis

Subgroup analysis was conducted to examine primary and secondary outcomes with statistically significant effects for the primary contrast (EMAF vs EMAF plus PF plus PPC). We compared patients with less than 3 months of consecutive opioid prescriptions in the past 3 months vs those with 3 months of consecutive prescriptions to examine potential differences in patients with longer- vs shorter-term prescribing patterns.

#### Less Than 3 Months

For patients with less than 3 months of consecutive opioid prescriptions, there was a significant reduction in mean MME of −2.2 (95% CI, −4.5 to 0.01) mg/d (*P* = .05) in the EMAF plus PF plus PPC group vs the EMAF group. There was a significant difference in urine drug screening of −5.1% (−8.0% to −2.1% [*P* < .001]) and treatment agreements of −3.1% (−6.0% to 0.3% [*P* = .03]) in the EMAF plus PF plus PPC vs EMAF groups. For PEG-3 screening, significant increases were observed in the EMAF plus PF plus PPC group (5.6% [95% CI, 0.8%-10.3%]; *P* = .02) compared with EMAF (Table 3).

**Table 3.** Patient Subgroup Analysis^a^

Item	<3-mo Subgroup			3-mo Subgroup		
	18-mo Change		EMAF plus PF plus PPC vs EMAF	18-mo Change		EMAF plus PF plus PPC vs EMAF
	EMAF	EMAF plus PF plus PPC		EMAF	EMAF plus PF plus PPC	
**Mean MME**						
Estimate, mg/d (95% CI)	−1.1 (−2.8 to 0.5)	−3.4 (−5.0 to −1.8)	−2.2 (−4.5 to 0.01)	−1.5 (−4.0 to 1.0)	−2.4 (−5.2 to 0.3)	−0.9 (−4.6 to 2.7)
Cohen *d*	−0.08	−0.24	−0.16	−0.05	−0.09	−0.03
*P* value	.18	<.001	.05	.24	.08	.62
**Urine screen**						
Estimate, % (95% CI)	4.5 (2.3 to 6.7)	−0.6 (−2.7 to 1.5)	−5.1 (−8.0 to −2.1)	13.3 (7.8 to 18.8)	−1.1 (−6.9 to 4.6)	−14.4 (−22.3 to −6.5)
Cohen *d*	0.39	−0.05	−0.44	0.45	−0.04	−0.48
*P* value	<.001	.59	<.001	<.001	.71	<.001
**Treatment agreement**						
Estimate, % (95% CI)	2.8 (0.7 to 4.9)	−0.3 (−2.4 to 1.7)	−3.1 (−6.0 to 0.3)	16.2 (10.1 to 22.2)	2.3 (−3.8 to 8.5)	−13.8 (−22.2 to −5.4)
Cohen *d*	0.30	−0.04	−0.34	0.55	0.08	−0.47
*P* value	.01	.75	.03	<.001	.46	.001
**PHQ-2 screen**						
Estimate, % (95% CI)	14.0 (9.8 to 18.2)	14.3 (10.2 to 18.4)	0.3 (−5.5 to 6.1)	16.5 (10.5 to 22.5)	11.2 (5.1 to 17.3)	−5.3 (−13.6 to 3.0)
Cohen *d*	0.61	0.62	0.01	0.54	0.36	−0.17
*P* value	<.001	<.001	.92	<.001	<.001	.21
**PEG-3 screen**						
Estimate, % (95% CI)	1.1 (−2.4 to 4.6)	6.7 (3.3 to 10.1)	5.6 (0.8 to 10.3)	4.5 (0.1 to 9.1)	8.6 (4.0 to 13.2)	4.1 (−2.4 to 10.6)
Cohen *d*	0.08	0.49	0.41	0.23	0.44	0.21
*P* value	.53	<.001	.02	.05	.04	.22
**Aggregated *P* value^b^**	<.001	<.001	<.001	<.001	<.001	<.001

Abbreviations: AF, audit and feedback; EM, educational meetings; MME, morphine milligram equivalent daily dose; PEG-3, Pain, Enjoyment, General Activity; PF, practice facilitation; PHQ-2, Patient Health Questionnaire–2; PPC, prescriber peer consulting.

^a^ Subgroups are patients with opioid prescriptions in each of the most recent 3 months (3-month subgroup) vs patients with 0, 1, or 2 opioid prescriptions in the most recent 3 months (<3-month subgroup). Values were estimated using a linear mixed-effects model with the restricted maximum likelihood method. This approach was applied to assess the change in the fixed-effect curve from baseline to the end of the intervention period (18 months), within and among the 4 study arms.

^b^ The geometric mean of *P* values was used to calculate the aggregated *P* values, which can be used as a method for multiple-comparison correction to assess the null hypothesis that the true changes (or differences in changes) for all 5 of the outcomes are uniformly zero.

#### 3 Months

For patients with opioid prescriptions in 3 consecutive months, there was no significant difference in MME between the EMAF vs EMAF plus PF plus PPC groups. The change in percentage of patients with urine drug screening in the last 12 months was 14.4% (95% CI, −22.3% to −6.5% [*P* < .001]) lower in the EMAF plus PF plus PPC vs EMAF groups. Use of treatment agreements was 13.8% (95% CI, −22.2% to −5.4% [*P* = .001]) lower in EMAF plus PF plus PPC vs EMAF groups.

## Discussion

In this cluster randomized clinical trial, we found that the most intensive implementation strategy (EMAF plus PF plus PPC) had a small but statistically significant effect on MME daily dose compared with the least intensive (EMAF) strategy (difference, −2.4 mg/d). Baseline value for MME was 38.7 mg/d across all groups, well below the 90.0 mg/d threshold emphasized in the 2016 CDC guidelines^4^; the difference in MME of −2.4 mg/d represents approximately a 6% reduction. A previous pilot test of the implementation strategy had higher baseline MME values (86.0 mg/d) and larger reductions (11%) compared with this fully powered trial, reflecting steeply declining opioid prescribing across the US between 2017 and 2021.^16,24^

Implementation research around opioid prescribing has been limited. A 2020 systematic review on deprescribing opioids for chronic pain identified 10 patient-randomized and 2 cluster randomized trials^25^; 0 of 10 patient interventions and only 1 clinician intervention reduced the opioid dose. Liebschutz et al^26^ increased the use of treatment agreements and urine drug screening (their primary outcomes) in a cluster randomized clinical trial by including a dedicated nurse case manager who worked directly with patients on long-term opioid therapy, and their intervention reduced MME as a secondary outcome. In a recent (2023) patient-level trial, Sandhu et al^27^ demonstrated effectiveness of a group-based educational intervention in discontinuing opioid use. Alderson et al^28^ showed reductions in opioid prescribing using system-level feedback approaches. Our study fills an evidence gap by testing deimplementation strategies directed at health systems, prescribers, and clinic staff.^29^ Our study is among the first deimplementation cluster randomized trials to show effectiveness on a prospectively specified primary outcome and among the first specifically addressing opioid prescribing.

The 2016 CDC guidelines were debated extensively and ultimately revised in February 2022, near the end of the intervention period, with particular emphasis on mitigating potential misapplication related to opioid dosing thresholds. In our subgroup analysis, reductions in MME were driven primarily by patients with less than 3 months of consecutive prescriptions, consistent with CDC guidance to start with low opioid doses; this has arguably been the most solidly backed recommendation contained in the guidelines associating MME with overdose risk.^30,31^ There was no significant reduction in MME for the patient subgroup with 3 months of consecutive opioid prescriptions, perhaps in response to concern over the harms of involuntary tapering among patients receiving long-term opioid therapy.^32,33^

The most intensive strategy (EMAF plus PF plus PPC) had negative effects on treatment agreements and urine drug screening compared with the least intensive strategy (EMAF), while the more intensive strategies increased PEG-3 screening. These findings may reflect changing opinions about the evidence base behind these guideline elements.^5,34^ Treatment agreements and urine drug screening represented initial metrics used to assess opioid guideline concordance^26^; however, more recent evidence indicates weak support for treatment agreements’ effectiveness in opioid misuse mitigation.^35^ Likewise, the evidence base for urine drug screening is weak, and testing can be stigmatizing and burdensome for patients.^36^ Clinics receiving the low-intensity strategy may have implemented these recommendations without clinical context that was reinforced in clinics that received PF and PPC interventions. Results suggest that more intensive implementation strategies led to more patient-centered screening efforts focused on pain severity and quality of life. These findings are in line with recent calls to shift quality measures for chronic pain management from process to outcomes.^37^

### Limitations

This study has some limitations. The study was conducted in 2 health systems serving relatively rural, mostly White populations, limiting generalizability. While all eligible clinics were invited to participate, those that volunteered may have had more motivated leadership. COVID-19 interrupted the study, diverting clinical focus away from opioid prescribing. Interactions between the implementation team and participants were virtual, potentially reducing the effectiveness of planned in-person strategies. COVID-19 also led to temporary clinic consolidation, complicating implementation strategies and possibly blurring study group boundaries. Due to the study’s pragmatic study design making inherent trade-offs that favor external over internal validity,^38^ blinding investigators or participants to group assignment was infeasible. Future research is needed to confirm effectiveness of the adaptive intervention.

## Conclusions

In this cluster randomized clinical trial, results support clinician- and clinic-targeted deimplementation strategies to promote evidence-based opioid prescribing. Altering the dose of a prescription opioid involves medically driven decision-making, requiring knowledge transfer and support. Clinic-level workflow changes related to screening are based on practice management. Thus, less intensive strategies (including EMAF) may be suitable for implementing procedural guideline elements. Implementation trials are challenging due to evolving clinical guidelines, reflecting the dynamic interaction between policy and practice as evidence for guideline elements accumulates over time. Health system leaders’ choice of implementation strategies may depend on the priority they assign to specific guideline elements, clinics’ baseline performance levels, and balancing intervention effectiveness with cost efficiency.

## Notes

Supplement 1.
Trial Protocol

Supplement 2. **eMethods.** Opioid Study Primary Analysis—Statistical Modeling
eReferences.

Supplement 3.
Data Sharing Statement
