# Supplementary material for: Strategies to Deimplement Opioid Prescribing in Primary Care: A Cluster Randomized Clinical Trial
Source: JAMA Netw Open. 2024 Oct 10;7(10):e2438325. doi: 10.1001/jamanetworkopen.2024.38325 (PMC11581553; doi:10.1001/jamanetworkopen.2024.38325)
Supplement: Supplement 1. — Trial Protocol [file jamanetwopen-e2438325-s001.pdf]

Promoting the implementation of clinical guidelines for opioid prescribing in primary care using systems consultation

Principal Investigator: **Andrew R. Quanbeck, PhD.**
University of Wisconsin – Madison
Department of Family Medicine and Community Health
800 University Bay Drive
Madison, WI 53705
Phone: 608.262.7385
Email: arquanbe@wisc.edu

Co-Investigators: **Randall Brown, M.D.**
University of Wisconsin – Madison
Department of Family Medicine and Community Health
1100 Delaplaine Court
Madison, WI 53715
Phone: 608.263.6558
Email: randy.brown@fammed.wisc.edu

Jillian Landeck, M.D.
University of Wisconsin – Madison
Department of Family Medicine and Community Health
1100 Delaplaine Court
Madison, WI 53715
Phone: 608.424.3384
Email: jillian.landeck@fammed.wisc.edu

Nora Jacobson, PhD.
University of Wisconsin – Madison
School of Nursing
701 Highland Avenue
Madison, WI 53705
Phone: 608.262.8034
Email: najacobson@wisc.edu

Paul Hunter, M.D.
University of Wisconsin – Madison
Department of Family Medicine and Community Health
1100 Delaplaine Court
Madison, WI 53715
Phone: 608.265.0477
Email: phhunter@wisc.edu

David H. Gustafson, PhD.
University of Wisconsin – Madison
College of Engineering – Industrial & Systems Engineering
1513 University Avenue
Madison, WI 53706
Phone: 608.263.4882

Email: dhgustafson@wisc.edu

Daniel Almirall, PhD.
University of Michigan
Institute for Social Research
426 Thompson Street
Ann Arbor, MI 48104
Phone: 734.936.3077
Email: dalmiral@umich.edu

Consultants: **Lynn Madden, PhD.**
University of Wisconsin – Madison
College of Engineering – Industrial & Systems Engineering
1513 University Avenue
Madison, WI 53706
Phone: NA
Email: lmadden3@wisc.edu

Funder: **National Institute of Health: NIDA**

Coordinating Center: **Implementation and Engineering Science Lab**
University of Wisconsin – Madison

IRB Serving of Record: **University of Wisconsin – Madison IRB**

Participating Institutions:

Bellin Health
Andrew Cohen
Site Principal Investigator
744 South Webster Avenue
Green Bay, WI 54305
Phone: 920-433-3642
Email: andrew.cohen@bellin.org

University of Michigan
500 S State Street
Ann Arbor, MI 48109

UW Health
600 Highland Avenue
Madison, WI 53792

Table of Contents

Abstract

1. Introduction/Background
2. Objectives
3. Selection of Clinics and Subjects
4. Registration Procedures
5. Treatment Plan
6. Measurement of Effect
7. Study Parameters
8. Statistical Considerations
9. Records to be Kept
10. References

Abstract

The proposed study uses a sequential, multiple assignment randomized trial (SMART) [1,2] to test an adaptive version of systems consultation[3] consisting of academic detailing, practice facilitation, and physician peer coaching to increase the uptake of safer opioid prescribing practices at three levels of primary care (health system, clinic, prescriber). Academic detailing will be provided to all clinics and prescribers to inform prescribers on the goals of the study, the importance of improving opioid prescribing in primary care, and a review of the latest standards of prescribing guidelines. After three months, clinics will be randomized to receive nothing or receive practice facilitation where clinics will get an in-person or online visit and proceed with monthly teleconferences to improve clinic workflow related to opioid prescribing. Six months later, clinics will receive physician peer coaching that will employ two experienced addiction specialists who will provide guidance to prescribers dealing with tough patient panels. Additionally, this study aims to capture and analyze contextual factors that influence implementation in order to create a simple tool that will guide quality improvement in opioid prescribing. This SMART will deliver these three strategies to 40 primary care clinics from three health systems to compare the effect on average morphine milligram dose of an adaptive systems consultation implementation strategy compared to academic detailing alone over a 21 month time period. To our knowledge, this study would be the first to randomize at two levels to test the efficacy of the implementation of system consultation to improve opioid prescribing in primary care.

1. Background & Significance

Importance of improving prescribing practices in primary care

Opioids are commonly prescribed in primary care to relieve chronic pain. Although potentially effective for this purpose, accompanying burdens have become clear and widespread. In 2017, drug overdose was the leading cause of accidental death in the United States. Although the volume of opioids prescribed in the US declined each year from 2010 to 2015, about 3 times more opioids were prescribed per person in 2015 as in 1999, and prescribing rates still vary greatly, with the highest-prescribing counties prescribing 6 times more opioids per person than the lowest-prescribing counties.[4] In 2015, 63.1% of drug-overdose deaths involved an opioid,[5] and approximately half of opioid-related deaths involved prescription opioids.[6] About half of opioid prescriptions are written in primary care.[7,8] Clinical guidelines for opioid prescribing in primary care have been advanced, most notably the guidelines issued by the Centers for Disease Control and Prevention in 2016.[8] Clinical guidelines have established consensus around a few basic ideas: (1) Physicians should discuss the risks and benefits of opioid therapy with patients by reviewing and signing formal treatment agreements before initiating the first opioid dose and throughout treatment.[8] (2) Clinicians should avoid prescribing opioids in doses higher than 90-100 morphine milligram equivalent (MME) daily; evidence shows that patients with a dose of 100 MME or greater are 11 times more likely to die from overdose than patients taking doses less than 20 MME.[9-11] (3) patients at increased risk for misuse (i.e., those with mental health or substance use disorders) are more likely to receive opioid prescriptions and higher doses; thus, screening for mental health and substance use disorders should be in place.[9,12-14] (4) Opioid-benzodiazepine co-prescribing in any combination of doses should be avoided to reduce the risk of overdose.[15] (5) Monitoring via urine drug testing should be instituted to ensure appropriate use of opioid medications.[16]

Strategies for implementing evidence-based practices

Despite the promotion of evidence-based practices (EBPs) related to opioid prescribing (and many other conditions), the healthcare system is notoriously slow in adopting EBPs.[17] Lau et al. conducted a 2015 review[18] of 91 studies aimed at determining the effectiveness of strategies for implementation of complex interventions in primary care settings. The most commonly used strategies were targeted at individual providers, generally demonstrating small to modest effects, with considerable variability between studies. The authors found little use of implementation strategies targeted at organizations or a wider context (e.g., health systems). Finally, the review found very limited data on the costs and cost-effectiveness of different implementation strategies should be used under what conditions, and that future research should study implementation strategies targeted at levels more broadly defined than individual providers. According to a systematic review focused specifically on the role of external change agents (e.g., coaches, facilitators, academic detailers, etc.) in primary care settings, the more successful implementation strategies tended to be those that offered regular, context-specific follow-up.[19]

Systems consultation

Systems consultation is a theoretically and empirically grounded,[3] blended implementation strategy[20,21] consisting of a bundle of 3 discrete strategies: (1) Academic detailing, in which an experienced physician provides recommendations and advice on how to improve clinical practice; (2) Practice facilitation, a team-based advising approach designed to tailor implementation to specific

clinical contexts; and (3) Physician peer coaching, in which an experienced physician provides one-on-one clinical advice on managing patient panels to other physicians (who may be less experienced with the selected clinical issue). Several other discrete strategies are also integrated into the systems consultation model, as categorized by Powell et al.'s (2015)[20] taxonomy of implementation strategies, including: use of community-academic partnership, where leaders from an academic medical center work with community- based care clinics to improve the health of their communities; audit and feedback , which consists of providing performance feedback to clinics; conducting local assessments to determine the need for implementation; and tailoring implementation strategies to address potential barriers and facilitators.

Adapting implementation strategies to specific contexts

Primary care clinics vary in their opioid prescribing practices and in their capacity to carry out implementation efforts. Clinics and prescribers are influenced by factors such as funding, regulations, geography, and policies. These considerations suggest that strategies for implementing EBPs will work differently in different clinics; moreover, different strategies might work best for different prescribers, depending on the prescriber's patient panel, experience in prescribing opioids, and other influences. Previous implementation research[22] suggests that implementation strategies tailored to specific clinics are the most effective, although evidence also suggests that the effect of tailoring varies, and tends to be small to moderate.[23] The literature does not specify the most effective approaches to adaptation or the cost-effectiveness of tailored strategies (compared with non-tailored strategies).[18,23]

2. Study Objectives

This study aims to understand the optimal sequencing and combination of implementation strategies that specific types of clinics and prescribers need to adopt clinical guidelines for opioid prescribing. The pragmatic goal is to give health systems a tool they can use to predict which clinics and prescribers will benefit most from which sequence and combination of implementation strategies. The bundle of strategies systems consultation consists of include academic detailing, practice facilitation, and physician peer coaching. We propose to recruit 40 clinics and 152 prescribers from 2 health systems for a sequential, multiple assignment randomized trial to receive academic detailing only, academic detailing + practice facilitation, academic detailing + practice facilitation + physician peer coaching, or academic detailing + physician peer coaching. We will use quantitative and qualitative analyses to compare outcomes of sequences and combinations with data collected every 3 months over the 21-month intervention period.

Primary Aim:

Compare the effect of an adaptive systems consultation implementation strategy vs. academic detailing alone on average morphine milligram equivalent dose over 21 months.

Secondary Aims:

- Develop an assessment of contextual factors that influence the effectiveness of different implementation strategies.

- Test 4 moderators and assess other factors that affect implementation to use to predict which implementation strategies will be most effective in different settings.
- Estimate the costs of delivering 4 different sequences and combinations of strategies, including the incremental cost effectiveness of adding facilitation and physician peer coaching.

Study Coordination

The UW-Madison Implementation Science and Engineering Lab is the coordinating site for this study. The UW study coordinator will oversee all activities at the 40 clinics which include:

- developing site specific recruitment and data collection processes that meet study objectives;
- training site staff prior to the study on protocol procedures to maintain compliance with the protocol and human subjects regulation;
- communicating with site staff via monthly correspondence to monitor progress, inform of protocol changes/distribute new version of protocol, and address unanticipated issues or challenges;
- and manage all study data.

<u>3. Selection of Subjects</u>

Patients are not the subject of the study. The subjects of study will be the clinic prescribers. Prescriber panel data will be aggregated to the clinic and prescriber levels. To be included in the aggregated, de-identified prescriber panel data, patients must:

1. have a primary care provider at the clinic;
2. prescriptions for opioid therapy for at least 3 consecutive months;
3. and no cancer diagnosis or reception of hospice care.

Clinics: A total of 40 clinics will be recruited from primary care clinics of the two health systems: UW Health and Bellin Health. This protocol will detail the process at UW Health primary care clinics for which the HS IRB is the IRB of record. Bellin Health will follow similar procedures with oversight from the UW study coordinator.

Clinics will be eligible for the study if they:

1. are a primary care clinic (non-pediatric primary care, internal medicine, or family medicine);
2. have not received the systems consultation intervention; and
3. do not explicitly prohibit initiating opioid therapy.

Change team: Up to 7 team members will be recruited and consented to participate in practice facilitation. Change teams include a change team leader (likely a clinic medical director or other clinician) and supporting clinic staff such as nurse practitioners, physician assistants, registered nurses, lab technicians, and medical assistants.

Prescribers: de-identified patient data will be aggregated at the level of the prescriber and clinic. In this context, prescribers will be known to the study team only by the code number assigned to them by HIP. Data will be obtained only from those clinicians who:

1. are primary care physicians or other providers with prescribing privileges;
2. are not temporary providers who do not manage stable panels or patients;

4. Registration Procedures

Clinic Recruitment

The study team will discuss the study with health system leaders who gave letters of support and identify clinics that may be interested in participating. The study team will present the study to clinicians at an all-provider meeting to build awareness and inform clinics that this study is available to join. Health care leaders will send an email on behalf of the study team to clinic medical directors of the health system. The email will notify medical directors that research is being done at clinics of their health system and instruct them how to opt-out if they wish to decline participation in the study and future contact from the study team. The email will instruct medical directors to direct any questions they have to the study team.

Medical directors who choose not to opt-out of the study will be invited via email to attend the regional academic detailing meetings. Medical directors will be asked to forward the email to any clinic staff who may be interested in participating in the academic detailing meeting. The email will instruct interested medical directors and clinic staff to call the study team if they are interested in attending or have any questions. Clinics will be asked to allow medical directors and interested clinic staff to attend regional academic detailing meetings on clinic time where the study team will explain the study objectives and participation expectations. Medical directors and clinicians will sign consent forms and will be handed information sheets after the study is explained, but before further information about opioid prescribing is presented. Those attending the academic detailing meeting via webinar will be sent the consent form electronically and sign and return the consent form before the meeting. All signed consent forms will be sent via email, fax, or postal service to the study team. Medical directors will be assured that there is no obligation to participate in the study and that their decision is voluntary. The study team will explain to medical directors that the clinic and clinicians can drop out from study participation at any time and that their clinical practice will in no way be affected by their choice to participate or not.

Staff Recruitment

Following the academic detailing meeting an email will be sent out to all clinic staff by the medical director of behalf of the study team. The email will inform clinic staff that research may be conducted at their clinic, what the research activities they may be asked to participate in include, and that they should contact the study team if they have any questions. While clinicians have the opportunity to opt-out of individual-level activities, they still may be involved in the research by virtue of doing their jobs in a clinic where the study is occurring. In a separate email the study team will provide the clinicians who could not make the academic detailing meeting with a link to a webinar. The study team will ask prescribers and clinicians who may participate on the change team who are interested in participating in the study to watch the webinar and sign the consent form. All signed consent forms will be sent via email, fax, or postal service to the study team.

The study team will call medical directors of clinics that have been randomized to receive practice facilitation to schedule the online or in-person clinic visit and ask the medical director to identify individuals who might be interested in participating on a change team. The medical director will

identify an appropriate individual who might be interested in being a change team leader, and up to 5 additional clinicians to serve as change team members. At the online or in-person clinic visit the study team will provide change team members with a virtual or hard copy information sheet about practice facilitation. The information sheet will inform change team members what is required of them and that their participation is voluntary. The change team will be told that there is no obligation to participate and that their clinical practice will in no way be affected by their choice to participate or not. The study team will assure clinicians that they can take their time to think about their participation and may leave the practice facilitation at any time. While most future meetings are anticipated to be virtual, any face-to-face contacts with clinician subjects will take place at the regional meeting, in the clinician subject's office or in a private room in the clinic at a convenient time for the clinician subject. Virtual meetings will be held securely over WebEx. Online practice facilitation sessions will be recorded to review for notes and accuracy. Notes will be kept securely on Box and recorded sessions will be destroyed.

Consent

A request for a waiver of informed consent will be made for health system leaders and for clinic staff in clinic-level interventions. The study team will ask health system leaders to send out an email on behalf of the study team, informing medical directors about the research and the ability to opt-out of future communication with the study team. Medical directors who do not opt-out by a specified date on the email will be invited to the academic detailing meetings and have the ability to forward the email to other interested clinicians. These subjects will sign consent forms and receive a study information sheet at the academic detailing meeting. Those attending the meeting via webinar will be asked to read, sign, and return consent forms prior to the meeting. Webinar attendees will send the signed consent form to the study team via email, fax, or mail.

After the academic detailing meetings, the medical director will send out an email on behalf of the study team to notify clinic employees that research may be done at their clinic and that they have the option to opt-out of the study activities. The study team will consent any prescriber and clinic staff who did not attend the academic detailing meeting, but wish to participate in the study via email. Prescribers will be sent an email with a link to the webinar of the academic detailing meeting and a consent form to read and sign. Prescribers will send the signed consent form to the study team via email, fax, or mail.

If participants decide to no longer participate in the study they will contact the PI, Andrew Quanbeck, to rescind their consent. Data that is already collected will be retained for analyses, but no further data of that participant will be provided to the study team.

A waiver of signed consent will be requested for qualitative interviews. Participants will be invited over email to participate in interviews. After a week, if the participant does not respond to the email the study team will call the participant to invite them to the study. The study team will call once. If there is no answer a new invite will be sent to a different participant. Participants who agree to be interviewed will be sent information sheets to read. This consent process is supplemental from the consent process participants went through at or before the educational meeting.

Randomization

Randomization will take place at the clinic level at month 3 (practice facilitation) and month 9 (physician peer coaching). A research team member will conduct the randomization using the urn randomization program.

Randomization of clinics will take place at intervention month 3 on a 1:1 ratio to assign clinics that will receive practice facilitation plus academic detailing or academic detailing only. At intervention month 9 all clinics will be randomized on a 1:1 ratio to assign clinics who will receive the current intervention strategy plus physician peer coaching or continue the current intervention strategy for 12 months. Randomizations will be stratified on clinic's 1) health system, 2) number of patients, and 3) average MME being greater or equal to the health system's clinic average.

5. Intervention Plan

While the intervention plan is continuing as intended, due to the COVID-19 pandemic, study intervention activities were put on hold between March 25th and July 15th. Study activities have since resumed as intended.

Subjects will receive a combination of the following strategies:

Academic detailing. Clinicians from participating clinics will be invited to a regionally hosted, in-person training session where they will be provided lunch. The study team will also have a webinar option (such as WebEx) for clinicians who cannot travel to the regional in-person meeting. The session will be designed to both inform and engage clinic staff in the study. The session will be led by Dr. Randall Brown and Jillian Landeck, who are experts in addiction medicine with extensive experience managing the care of long-term opioid patients. Invitees will be medical directors and prescribers (physicians, nurse practitioners, physician assistants), nurses, and staff directly involved in clinic workflows related to opioid prescribing (e.g., medical assistants, lab techs, etc.). We will ask each clinic to designate the clinic medical director to serve as contacts for the research team. The training will cover the goals of the study, the importance of improving opioid prescribing in primary care, a review of the latest standards of guidelines concordant care, how improvements in clinic workflows can affect patient outcomes, clinical topics such as how to address opioid-induced hyperalgesia and balancing patient-reported pain and function during dose reduction, and trainings on how to use electronic medical records to monitor key opioid prescribing outcomes. An assessment will be conducted during the session to elicit contextual characteristic that use the electronic health record to monitor key opioid prescribing outcomes. The assessment will be a survey given at meetings or online (for those joining via webinar)s. If clinicians are watching the academic detailing meeting on their own or via webinar, they can take the survey on REDCap. At the conclusion of the initial meeting, clinicians who attended the meeting will be asked to form change teams at their clinics.

A quarterly hour-long academic detailing meeting will be hosted via webinar (such as WebEx) to provide clinics and clinicians further information about the CDC opioid prescribing guidelines. Quarterly webinar meetings will be separate for each study arm.

Following the academic detailing meeting, the health systems will create and deliver audit & feedback reports to clinicians. Health systems are already doing this and will customize the reports to fit the study. These reports will let clinicians know about their clinic's opioid prescribing metrics such as average MME, % of patients with urine drug testing, treatment agreements, and mental health screens, and % of patients co-prescribed benzodiazepines. Reports will only be at the clinic level.

Practice facilitation. Research staff trained in practice facilitation and/or study team members will meet virtually with clinics using WebEx (or visit clinics in-person) and follow up over the course of up to 5 monthly then 4 quarterly videoconferences or teleconferences to help clinics improve processes related to opioid prescribing, such as ensuring that treatment agreements are signed by prescribers and patients at least once a year, and integrating urine drug testing into clinic workflows. Clinic medical directors will create change teams consisting of the clinic medical director and a physician change leader and up to 5 clinic staff. The online or in-person visit will begin with a meeting with the clinic medical director and the change team leader. At the meeting the facilitator will set a plan for the day and answer any questions about the meeting. The facilitator will tour the clinic in-person or virtually over WebEx.[24] The facilitator will then meet (in-person or virtually) with the change team for an hour to cover how to make workflow changes such as integrating treatment agreements and urine drug tests into clinic processes. The change team will be educated about the nominal group technique[25] and Plan-Do-Study-Act cycles[26] (PDSA cycles) to select the area of improvement for the first PDSA cycle. The practice facilitator will assist the change team in filling out the PDSA cycle and the practice facilitator will enter the info into the Change cycle data table for reference for the follow-up conference call. Then the facilitator will debrief with the medical director and change team leader to discuss the next steps for follow-up discussions. Over the next 18 months clinics will be able to call in or meet over WebEx for up to 5 monthly, 60 minute-long meetings, followed by 4 quarterly hour-long follow-up discussions about the workflow changes. Sessions will be recorded using encrypted audio recorders or WebEx's recording feature. Recordings will be used to ensure accurate note taking. Recordings from audio recorders will be stored on the facilitator's password-protected computer or laptop. Once note taking is completed the recordings will be destroyed.

Physician peer coaching. At UW Health, physician peer coaches will be Drs. Brown and Landeck. At Bellin Health, physicians with relevant experience in opioid prescribing will be nominated by health system leaders to become each systems' physician peer coaches. These coaches will become members of the research team. Drs. Brown and Landeck help their peers manage their patients on opioid therapy. Participating clinics randomized to physician peer coaching will receive up to 4 quarterly coaching sessions over 12 months. Physician peer coaches will provide help to prescribers to assist with tough panels through up to four quarterly coaching sessions over 12 months. Coaching consultations will occur in sessions via videoconference (WebEx) or teleconference. Participants attending the physician peer coaching sessions will be asked to take a survey before each session to let the coach know what topics the group needs help with. After each physician peer consulting session, participants will be asked to complete a post-session survey to assess how the session was received and how confident the participant feels about addressing the session topic with patients. After the fourth and last session, participants in the physician peer consultation interventions will be asked to take a final survey to evaluate how the intervention impacted their confidence and experiences treating patients prescribed opioids for chronic pain, and feedback on the intervention itself.

Data collection

Quantitative data. Evaluation data extracted from the electronic health records of patients on prescriber panels will be constructed and delivered by the Health Innovation Program (HIP) on behalf of the Wisconsin Collaborative for Healthcare Quality (WCHQ). All members of the collaborative (including UW Health) and other specially invited members (Bellin) submit patient level data extracted from health records to a central data repository (called RBS, or “Repository Based Data Submission”), which HIP is able to access through their Business Associate agreement with WCHQ. The electronic data from the WCHQ is accessed by HIP through a secure File Transfer Protocol (FTP) site that is set up by WCHQ. An FTP is a secure way of moving data from WCHQ to an outside organization such as HIP. Datasets for researchers are constructed from these identifiable datasets that have been transformed into de-identified datasets by HIP Programmers, who are not part of the study team. Patient-level data will be grouped by prescriber and clinic for this project, and prescribers and clinics will be coded so that the Programmers are able to deliver longitudinal data at the prescriber and clinic levels. The study team will not have access to a crosswalk or any other code that would allow re-identification of the de-identified dataset delivered by HIP to the research team. Datasets will be extracted and delivered from the start of the intervention to the end of the 6-month follow-up at intervention month 27. No sensitive information will be included in the analysis dataset. No individual PHI will be collected in the course of this study. Only aggregate statistical output representing groups of subjects will be released.

Qualitative data. The study team will conduct two sets of semi-structured interviews (exact questions will vary based on answers to other questions) using the UW sponsored HIPPA compliant WebEx. Semi-structured interviews will be conducted with two change team leaders or their designees (a total of 8 participants) selected randomly from each intervention group at each participating health system. The interviews will take place at intervention month 18 to better understand what practice facilitation activities were done at clinics, what worked and did not work, and feedback on the intervention. A second set of semi-structured interviews will be conducted with one prescriber (a total of 8 participants) selected randomly from each intervention group at each participating health system. These interviews will take place between intervention months 18-21 to better understand what peer support activities were done at clinics, what worked and did not work, what prescribers find problematic, how peer support groups helped prescribers address these issues, and feedback on the intervention. Qualitative interviews will be recorded and transcribed for analysis using WebEx’s recording and transcription feature. All identifying information will be coded and transcripts will be stored securely on a Box folder (no PHI will be uploaded to the Box folder). The recording will be destroyed after it is transcribed.

Assessments. This study has developed a tailoring assessment based on pilot data to test 4 moderators (existence of an opioid prescribing policy at the system level, experience of quality improvement at the clinic level, size of the clinic, and number of high dose patients) and assess other factors that affect implementation through open and closed-ended questions. At 0, 5, 9, and 21 months assessments will be administered online (via REDCap) and online or in-person surveys. If in-

person visits occur, participants will be asked to complete a hardcopy assessment. In-person assessment data will be entered into ICTR's REDCap.

Detailed contact logs. Research team members responsible for delivering the implementation strategies will keep detailed logs of contact with clinics and prescribers to estimate measures of adoption and implementation. All identifiable information will be de-identified by a research team member.

In the event that subjects (clinic or prescriber) choose to withdraw from the study, data that is already collected will be kept for analyses. No further data will be collected from that subject. If the clinic withdraws, all prescribers at that clinic will not have further data collected.

Physician peer consulting surveys

The study team will ask participants in the physician peer consulting intervention to take a pre- and post-session survey for each session and a final, post-session survey using UW Madison's version of Qualtrics. Surveys will ask questions about the topics prescribers want to discuss at the upcoming consultation session, how confident they are in addressing the issues discussed at the past session, and their experience with their consultant and the intervention. The surveys will only ask the participant to provide their health system and clinic they practice at so the study team will know which topics to discuss at each clinic's consulting session, understand how the consulting session was receive, and make any adaptations to future consulting sessions. For this reason, health system and clinic information will not be coded or de-identified. However, the research coordinator will code health system and clinic for the analysis.

Privacy and Confidentiality

To mitigate the risk of breaches of confidentiality, all subjects and clinics will be assigned a code number. A list of subject and clinic code numbers will be maintained by a research team member and stored in a password-protected spreadsheet. This data will be kept on a secure, limited access, password-protected file service on ICTR's REDCap and UW's Qualtircs.

Potential Risks: This research is aimed at improving clinical practice related to opioid prescribing, and falls ultimately under the context of increasing patient safety. As such, the study team believes the risks to clinician subjects and individual patients are minimal, and the intervention will improve patient safety. The potential risks of participation are:

1. Staff members could feel pressured to participate in the study. Opioid prescribing is a potentially controversial topic.
2. Prescribers may be uncomfortable discussing their prescribing practices and may resist attempts to change clinical practice. To mitigate any perceived pressure to participate in the study, the research team will make it clear, through written materials and oral instructions, that staff participation in the research is completely voluntary.
3. There could be a breach of confidentiality that could result in disclosure of research data outside the study team. To prevent this, all subjects will be assigned a code number. The lists will be kept in a locked file at HIP, and will not be shown to staff. Data collected will have the

names removed and the code attached by a research team member. Project staff who have access to data will not have access to subject names.

We have taken the following measures to reduce potential risk to subjects:

1. The primary units of analysis will be the clinic and prescribers; no staff members will ever be identified in presentations or publications.
2. The research team will emphasize the idea that the current implementation study is intended to improve opioid prescribing practices.
3. To address the risk of breach of confidentiality the research team will be provided only de-identified datasets and no individual provider will be able to be ascertained from these datasets nor will any member of the study team attempt to identify providers. Any qualitative data collected will be protected as well. Hard copy data will be kept in a locked cabinet and digital data will be stored on ICTR's REDCap.

UW-ICTR's Data Monitoring Committee.

This study will use UW-ICTR's Data Monitoring Committee. UW-ICTR has established a Data Monitoring Committee (DMC) to provide a key resource for UW-Madison investigators conducting clinical research. This DMC will provide investigators services to ensure appropriate measures are in place to promote subject safety, research integrity and compliance with federal regulations and local policies for individual clinical research protocols in need of DMC review (as determined by the Principal Investigator (PI), the funding agency, the local Scientific Review Committee, or the local IRB, and for which no DMC exists). For these studies, the UW ICTR DMC will be the primary data and safety advisory group for the Principal Investigator.

The DMC is supported in its mission of safety and compliance by experienced ICTR staff to provide administrative assistance, experienced members representing a diversity of backgrounds, skills and knowledge, and the use of the Research Electronic Data Capture (REDCap) tool which provides data management functionality by allowing the development of eCRFs and surveys to support data capture. In providing oversight for the conduct of this study, the ICTR DMC will meet every 12 months during the 5-year study. Additional meetings may be scheduled as determined by the DMC or as requested by the PI. The DMC members will review protocol-specific reports created by statisticians that serve a non-voting member role on the DMC using data pulled from REDCap. These standard reports will include an overview of study objectives, a review of actual and projected accrual rates, an evaluation of patient demographics for balance of randomization, and a summary of the number and seriousness of adverse events. An interim analysis of study results may be performed and source documents may be reviewed to allow the DMC to independently judge whether the overall integrity and conduct of the protocol remain acceptable based on data provided and reported by the Principal Investigator. The DMC will make recommendations to the Principal Investigator that could include actions of continuation, modification, suspension, or termination.

This study may be temporarily suspended or prematurely terminated if there is sufficient reasonable cause. Written notification, documenting the reason for study suspension or termination, will be provided by the suspending or terminating party to The National Institute on Drug Abuse. If the study

is prematurely terminated or suspended, the PI will promptly inform the IRB and will provide the reason(s) for the termination or suspension.

Circumstances that may warrant termination or suspension include, but are not limited to:
- Determination of unexpected, significant, or unacceptable risk to subjects
- Demonstration of efficacy that would warrant stopping
- Insufficient compliance to protocol requirements
- Data that are not sufficiently complete and/or evaluable
- Determination of futility

Study may resume once concerns about safety, protocol compliance, or data quality are addressed and satisfy the applicable federal and institutional regulatory authorities.

6. Measurement of Effect

This proposal uses the Reach, Effectiveness, Adoption, Implementation, and Maintenance (RE-AIM) model as an organizing evaluation framework[27] to examine the quality, speed, and impact of implementing the adaptive system consultation strategies. RE-AIM is a comprehensive evaluation framework that assesses implementation in five dimensions. While RE-AIM has been used to evaluate many diseases of fidelity.[28] Specific measures for each RE-AIM dimension are presented in Table 1.

7. Study Parameters

Table 1 Outcome measure by RE-AIM category

**Domain**	**Source**	**Pertains to aim:**	**Months* collected**
**Reach:** # and % of patients excluded vs. participating (incl. characteristics)	EHR	PA, SA1	1-21
**Effectiveness:** Overall rate of opioid prescribing and average MME per clinic and provider	EHR	PA	1-21
# and % of patients completing urine drug testing (past 12 mo.)	EHR	PA	1-21
# and % of patients screened for mental health using PHQ-2 (past 12 mo.)	EHR	PA	1-21
Mental health (PHQ-9) scores for patients screening positive on PHQ-2 (past 12 mo.)	EHR	PA	1-21
Overall rate and dose of opioid-benzodiazepine co-prescribing	EHR	PA	1-21
# and % of patients with treatment agreements (past 12 mo.)	EHR	PA	1-21
# and % of opioid prescriptions above 90 MME	EHR	PA, SA1	1-21
Patient attendance at scheduled clinic visits	EHR	PA, SA2	1-21
# and % of patients prescribed buprenorphine	EHR	PA	1-21
# and % of patients with PEG-3 score (past 12 mo.)	EHR	PA	1-21
PEG-3 scores (past 12 mo.)	EHR	PA	1-21
**Adoption (setting):** # and % of participating clinics vs. all clinics (incl. characteristics)	HS	SA1	1-21
**Adoption (staff):** # and % of participating staff vs. all eligible clinic staff (incl. characteristics)	Clinic	SA1	1-21
Clinician attendance at intervention meetings	RT	SA1	1-21
**Implementation:** Hours of intervention received per clinic & prescriber	RT	SA1	1-21
Adaptations made to protocols during intervention period	RT	SA1	1-21
Assessment of 4 moderators: system-level opioid prescribing policy, clinic-level experience in QI, size of clinic (# patients), # and % of patients on opioid doses > 90 MME	RT; EHR	SA1	0, 5, 9, 21
Qualitative assessment of mechanisms of action & factors influencing implementation	RT	SA1	1-21
Cost of each different implementation sequence & combination	RT	SA2	1-21

**Maintenance:** 6-mo. Follow-up on all effectiveness outcomes	EHR	All aims	22-27
***Months correspond to intervention months**<br>PA: Primary aim; SA1, Secondary aim 1; SA2, Secondary aim 2; RT: Research team; HS: Health system; EHR: Electronic health record			

Clinics will be randomized on a 1:1 ratio to either receive practice facilitation plus academic detailing or academic detailing alone at month 3. At month 9, all clinics will be randomized on a 1:1 ratio to either continue the current intervention or add physician peer coaching to the bundle of strategies. It is anticipated that up to 152 prescribers will be recruited from 40 primary care clinics among 2 healthcare systems. The intervention will last for 21 months followed by a 6 month follow-up period.

<u>8. Statistical Considerations</u>

Research Design

Analyses will be conducted at two levels, (1) at the clinic level and (2) at the prescriber level. UW Health and Bellin Health primary care clinics will be recruited for participation in the intervention and data collection activities. All clinics and all prescribers within clinics, once randomized, will be included in the intent-to-treat sample. The primary research outcome is morphine milligram equivalent (MME) and will be available for all prescribers within all clinics that consent, regardless of intervention engagement or drop-out.

This study will use a sequential, multiple assignment randomized trial (SMART), which is a factorial design (see Figure 1).[1,2] This SMART has 4 implementation strategies embedded within it.

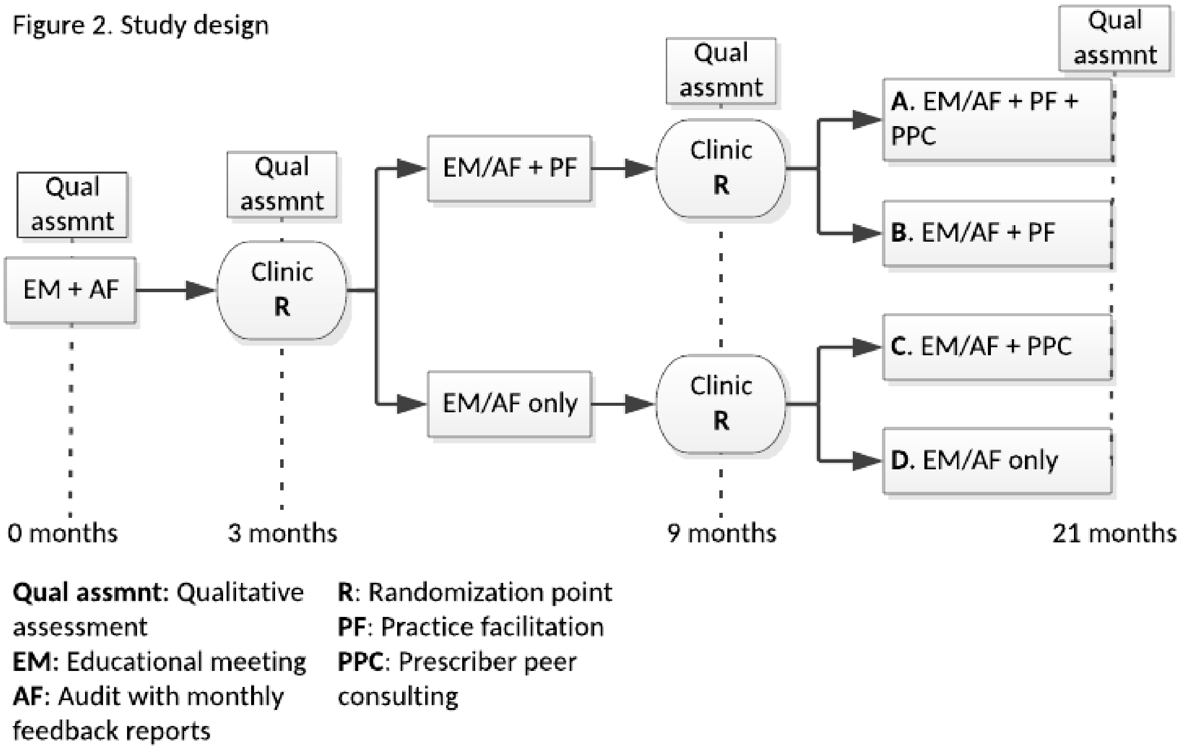

Quantitative data collection and analysis

Data are available for many study measures, allowing for time-series analysis of repeated measures to detect changes in a clinic over time (pre-intervention vs. post-intervention). Clinics and prescribers will be compared to clinics and prescribers receiving only academic detailing by accessing measures through the Wisconsin Collaborative Healthcare Quality system-wide data warehouse at Health Innovation Program (HIP). Programmers and compliance officers at HIP will prepare a de-identified dataset analysis purposes by the study statistician. The dataset will remain on HIP's secure data servers at all times.

The quantitative analysis of data from the electronic health record (EHR) will focus primarily on average daily morphine milligram equivalent for chronic pain patients at the clinic and prescriber levels. Changes in outcomes will be assessed through repeated monthly observations assessed retrospectively post-intervention. Data will be collected every three months throughout the 21 month intervention and 6 month follow-up period.

The study team will analyze the de-identified dataset delivered by HIP for evaluation purposes. The research team will not have access to any individual patient data or PHI. Prescriber identities will be protected; identifying information (such as staff names) will be replaced with code numbers by the HIP Programmers. The de-identified dataset will be coded by prescriber and clinic and the code key will be kept by HIP Programmers and not provided to the study team.

Due to delays in the data delivery from the Wisconsin Collaborative Healthcare Quality the research team will also receive and analyze data that the UW Clinical Research Data Services (CRDS) provides for the clinic feedback reports. The purpose for this is to speed up data analysis. CRDS will act as an honest broker and will not provide the research team with patient-level identifiable data. CRDS will provide data to the research team through ICTR REDCap's file repository feature.

Qualitative data collection and analysis

Interviews over WebEx for practice facilitation and physician peer coaching will be recorded and transcribed. Subjects will be reminded that calls are recorded. The recordings will be transcribed and coded by the study team. Qualitative analyses will be done using Nvivo.

Qualitative assessments will be conducted to assess contextual factors of systems consultation. These assessments will be coded to group data at the clinic level for analysis and take place at months 0, 5, 9, and 21 of the intervention.

Qualitative data will be collected via REDCap at the prescriber levels will be stored REDCap for analysis purposes. Any publication that results from the study data will not include the names of clinics or staff members where data were collected. All other results will be presented in anonymous aggregated form.

Statistical analysis

Statistical analysis will be conducted by Daniel Almirall at the University of Michigan. Dr. Almirall will be provided a secure, remote login to the password-protected servers at the Health Innovation Program to access the de-identified datasets.

The analysis will use a longitudinal (repeated-measures) analysis. Time will be coded such that t=0 denotes month 3 of the intervention period (the initial randomization); in the following text, data collected prior to t=0 is considered baseline data (including the MME prior to month 3). The primary outcome (MME) is a continuous measure and is collected at t=0 (at month 3, prior to randomization) and every 3 months up to t=18 (intervention month 21) for a total of 7 measurement occasions. (Note that the primary outcome is clustered: repeated measures, within prescribers, within clinics.) Longitudinal regression models, adapted for the analysis of a longitudinal SMART[29,30] and further extended for use with data arising from a cluster-randomized SMART,[31] will be used to contrast the 4 sequences and combinations of implementation strategies in terms of the average change in MME.

A piecewise-linear model with a knot at t=6 (MME collected immediately before the second randomization at intervention month 9) will be used to model the temporal trajectories over the course of months 10-21. The model the study plans to use has a linear trend from t=0 to t=3 for prescribers in academic detailing plus practice facilitation and academic detailing only clinics, and a linear trend from t=3 to month t=18 for each of the 4 sequences and combinations of implementation strategies. We allow for changes in the mean trajectory (i.e., deflections) at intervention month 9 (t=6) since this is the point at which prescribers may begin receiving physician peer coaching.

Cost analysis

This study will estimate the cost of delivering the four different sequences and combinations of strategies, including incremental cost effectiveness of adding practice facilitation and physician peer coaching. Methods and instruments used for cost data collection in the Systems Consultation R34[3] will be used. Detailed logs of call contacts between members of the research team and the clinic change teams will be kept to estimate the number of hours spent delivering the implementation strategy. The consultants will document the date and duration of each contact they have with clinic staff members, role of the staff member, and a summary of topics discussed. Cost estimates of the intervention will be calculated by multiplying hourly wage rates for physician peer coaches and practice facilitators. Costs for non-personnel may include travel to site visits, the cost of teleconferencing services for follow-up calls, etc.

Power

The estimated sample size for this study is based on the primary aim: a comparison on change in morphine milligram equivalent (MME) between implementation sequences of all three strategies vs. academic detailing only. Based on the pilot data, the inter-clinic correlation coefficient was 0.14. Assuming the average prescribers per clinic is 4, a type-1 error rate of 5%, and an early adoption rate of 50% gives a total of 40 primary care clinics at 80% power to detect an effect size of d=0.66 between the two implementation sequences on change in MME. In the pilot data we found a standard deviation of 35 MME, which is estimated to detect differences on MME as small as 18 MME.

9. Records to be Kept

Data to be collected	Storage	Type of Data
Clinician subject intake	REDCap/ Locked cabinet	Coded
Clinician subject demographics	REDCap/ Locked cabinet	Coded
Qualitative assessment data	REDCap/ Locked cabinet	Coded
Prescriber consent form	REDCap/ Locked cabinet	Coded
Systems consultation coaching log	Box folder/ Locked cabinet	Coded
De-identified patient electronic health record data	HIP	De-identified
Practice facilitation notes	Box folder	Coded
Audio recordings from PF/PPC sessions	Facilitator computer/laptop (audio) or WebEx cloud storage (video)	Recording
Audio recordings from interviews	WebEx secure cloud storage	Recording
Transcriptions of interviews	Box folder	De-identified
Physician peer consulting survey	UW Qualtrics	Coded

10. References

1. Murphy SA, Bingham D. Screening experiments for developing dynamic treatment regimes. J Am Stat Assoc. 2009;104(458):391-408. PMCID: PMC2892819.

2. Lei H, Nahum-Shani I, Lynch K, Oslin D, Murphy SA. A "SMART" design for building individualized treatment sequences. Annu Rev Clin Psychol. 2012;8:21-48. PMCID: PMC3887122.

3. Quanbeck AB, Brown RT, Zgierska AE, Jacobson N, Robinson JM, Johnson RA, et al. A randomized matched-pairs study of feasibility, acceptability, and effectiveness of systems consultation: a novel implementation strategy for adopting clinical guidelines for opioid prescribing in primary care. Implement Sci. 2018;13:21.

4. Centers for Disease Control and Prevention (CDC). Vital Signs: Opioid Prescribing. Atlanta, GA: U.S. Department of Health & Health Services. Sept 26, 2017. Accessed on: Jan 25, 2018. Available at: https://www.cdc.gov/vitalsigns/opioids/index.html.

5. Rudd RA, Seth P, David F, Scholl L. Increases in drug and opioid-involved overdose deaths - United States, 2010-2015. MMWR Morb Mortal Wkly Rep. 2016;65(5051):1445-52.

6. Centers for Disease Control and Prevention (CDC). CDC WONDER. Atlanta, GA: U.S. Department of Health & Human Services. Jan 10, 2018. Accessed on: Jan 25, 2018. Available at: https://wonder.cdc.gov/.

7. Daubresse M, Chang HY, Yu Y, Viswanathan S, Shah ND, Stafford RS, et al. Ambulatory diagnosis and treatment of nonmalignant pain in the United States, 2000-2010. Med Care. 2013;51(10):870-8. PMCID:PMC3845222.

8. Dowell D HT, Chou R. CDC guideline for prescribing opioids for chronic pain — United States, 2016. MMWR Recomm Rep. 2016;65(RR-1):1-49.

9. Edlund MJ, Martin BC, Fan MY, Devries A, Braden JB, Sullivan MD. Risks for opioid abuse and dependence among recipients of chronic opioid therapy: Results from the TROUP Study. Drug Alcohol Depen. 2010;112(1-2):90-8. PMCID: PMC2967631.

10. Bohnert ASB, Valenstein M, Bair MJ, Ganoczy D, McCarthy JF, Ilgen MA, et al. Association between opioid prescribing patterns and opioid overdose-related deaths. JAMA. 2011;305(13):1315-21.

11. Dunn KM, Saunders KW, Rutter CM, Banta-Green CJ, Merrill JO, Sullivan MD, et al. Opioid prescriptions for chronic pain and overdose: a cohort study. Ann Intern Med. 2010;152(2):85-92. PMCID: PMC3000551.

12. Edlund MJ, Martin BC, Devries A, Fan MY, Braden JB, Sullivan MD. Trends in use of opioids for chronic noncancer pain among individuals with mental health and substance use disorders: the TROUP study. Clin J Pain. 2010;26(1):1-8. PMCID: PMC2917238.

13. Weisner CM, Campbell CI, Ray GT, Saunders K, Merrill JO, Banta-Green C, et al. Trends in prescribed opioid therapy for non-cancer pain for individuals with prior substance use disorders. Pain. 2009;145(3):287-93. PMCID: PMC2929845.

14. Banta-Green CJ, Merrill JO, Doyle SR, Boudreau DM, Calsyn DA. Opioid use behaviors, mental health and pain--development of a typology of chronic pain patients. Drug Alcohol Depend. 2009;104(1-2):34-42. PMCID: PMC2716214.

15. Gandam Venkata H, Gaurav C, Ram S, Talari G. Co-prescription of opioids and benzodiazepines. BMJ. 2017;356(j760).

16. Chou R, Fanciullo GJ, Fine PG, Adler JA, Ballantyne JC, Davies P, et al. Clinical guidelines for the use of chronic opioid therapy in chronic noncancer pain. J Pain. 2009;10(2):113-30. PMCID: PMC4043401.

17. Levine DM, Linder JA, Landon BE. The quality of outpatient care delivered to adults in the United States, 2002 to 2013. JAMA Intern Med. 2016;176(12):1778-90. .

18. Lau R, Stevenson F, Ong BN, Dziedzic K, Treweek S, Eldridge S, et al. Achieving change in primary care—effectiveness of strategies for improving implementation of complex interventions: systematic review of reviews. BMJ Open. 2015;5(12):e009993. PMCID: PMC4802575.

19. Alagoz E, Chih MY, Hitchcock M, Brown RT, Quanbeck A. The use of external change agents to promote quality improvement and organizational change in healthcare organizations: A systematic review. BMC Health Serv Res. 2018;18:42. PMC Journal- In Process.

20. Powell BJ, Waltz TJ, Chinman MJ, Damschroder LJ, Smith JL, Matthieu MM, et al. A refined compilation of implementation strategies: results from the Expert Recommendations for Implementing Change (ERIC) project. Implement Sci. 2015;10:21. PMCID: PMC4328074.

21. Proctor EK, Powell BJ, McMillen JC. Implementation strategies: recommendations for specifying and reporting. Implement Sci. 2013;8:139. PMCID: PMC3882890.

22. Wensing M, Bosch M, Grol R. Developing and selecting interventions for translating knowledge to action. CMAJ. 2010;182(2):E85-8. PMCID: PMC2817342.

23. Baker R, Camosso-Stefinovic J, Gillies C, Shaw EJ, Cheater F, Flottorp S, et al. Tailored interventions to address determinants of practice. Cochrane Database Syst Rev. 2015(4):CD005470. PMCID: PMC4164371.

24. Gustafson DH, Johnson KA, Capoccia V, Cotter F, Ford II JH, et al. The NIATx model: process improvement in behavioral health. Madison, WI: University of Wisconsin-Madison; 2011.

25. Delbecq AL, Van de Ven AH, Gustafson DH. Group techniques for program planning: a guide to nominal group and Delphi processes. Glenview, Ill: Scott Foresman Company; 1975.

26. Deming WE. Out of the crisis. Cambridge, Mass.: Massachusetts Institute of Technology, Center for Advanced Engineering Study; 1986.

27. Glasgow RE, Vogt TM, Boles SM. Evaluating the public health impact of health promotion interventions: the RE-AIM framework. Am J Public Health. 1999;89(9):1322-7. PMCID: PMC1508772.

28. Gaglio B, Shoup JA, Glasgow RE. The RE-AIM Framework: A systematic review of use over time. Am J Public Health. 2013; 103:e38-e46.

29. Lu X, Nahum-Shani I, Kasari C, Lynch KG, Oslin DW, Pelham WE, et al. Comparing dynamic treatment regimes using repeated-measures outcomes: modeling considerations in SMART studies. Stat Med. 2016;35(10):1595-615. PMCID: PMC4876020.

30. Almirall D, DiStefano C, Chang YC, Shire S, Kaiser A, Lu X, et al. Longitudinal effects of adaptive interventions with a speech-generating device in minimally verbal children with ASD. J Clin Child Adolesc Psychol. 2016;45(4):442-56. PMCID: PMC4930379.

31. NeCamp T, Kilbourne A, Almirall D. Comparing cluster-level dynamic treatment regimens using sequential, multiple assignment, randomized trials: Regression estimation and sample size considerations. Stat Methods Med Res. 2017;26(4):1572-89.
