# Supplementary material for: Strategies to Deimplement Opioid Prescribing in Primary Care: A Cluster Randomized Clinical Trial
Source: JAMA Netw Open. 2024 Oct 10;7(10):e2438325. doi: 10.1001/jamanetworkopen.2024.38325 (PMC11581553; doi:10.1001/jamanetworkopen.2024.38325)
Supplement: Supplement 2. — eMethods. Opioid Study Primary Analysis—Statistical Modeling eReferences. [file jamanetwopen-e2438325-s002.pdf]

Supplementary Online Content

Quanbeck A, Robinson J, Jacobson N, et al. Strategies to deimplement opioid prescribing in primary care: a cluster randomized clinical trial. *JAMA Netw Open*. 2024;7(10):e2438325.
doi:10.1001/jamanetworkopen.2024.38325

eMethods. Opioid Study Primary Analysis—Statistical Modeling
eReferences.

This supplementary material has been provided by the authors to give readers additional information about their work.

© 2024 Quanbeck A et al. *JAMA Network Open*.

eMethods. Opioid Study Primary Analysis—Statistical Modeling

- Overview
  This outline provides statistical modeling results for the opioid study primary analysis. These building blocks can be filtered, rearranged and modified as necessary for creation of a journal submission. Some aspects may be held in reserve to address questions from reviewers or to flesh out conference presentations. The tables and graphs below were copied from an Excel spreadsheet that may be easily modified to adjust fonts, colors, headings, etc.

- Sample Outcomes by PCP and Study Month for a Randomly Selected Clinic
  The first three graphs below provide examples of the progression of observed quarterly outcomes for a typical study clinic over the 18-month study intervention period. The sample clinic has seven primary care physicians (PCPs, labeled A through F) responsible for opioid prescriptions to clinic patients. The graphs display three of the seven primary analysis outcomes, i.e., average opioid patient morphine milligram equivalent (MME) daily doses for the quarter, the percent of opioid patients with average MME values at or above 90 mg per day, and the percent of opioid patients with a urine drug screen in the last 12 months. The fourth graph displays the number of opioid patients associated with each PCP over the 18-month period.

  These graphs are intended to give the audience a sense of the data layout and the level of outcome variation between PCPs and quarter to quarter for each PCP. Similar graphs at the study arm level contain too many PCP lines to track the progression of values for individual PCPs.

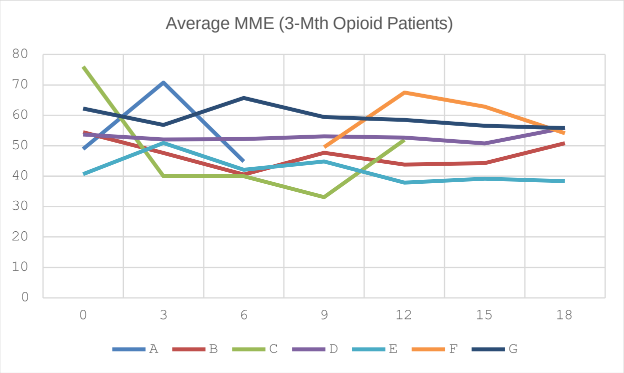

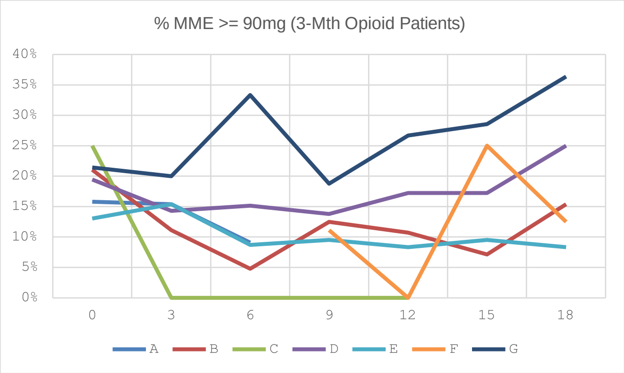

© 2024 Quanbeck A et al. *JAMA Network Open*.

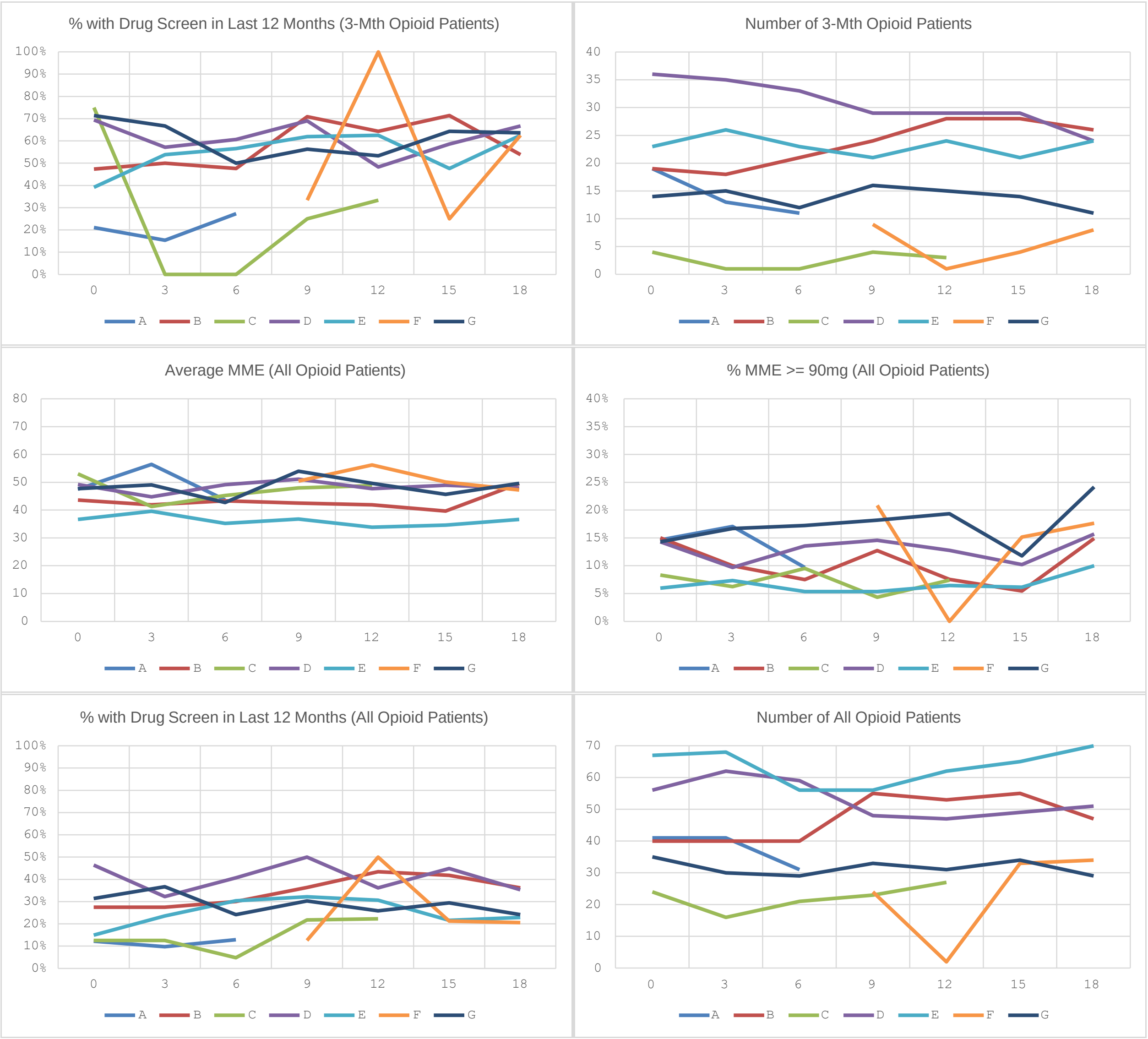

- The number of qualifying opioid patients varies significantly from PCP to PCP. The number of patients over time for a PCP varies to a lesser degree.
- Variance in outcomes over time is greater for PCPs with fewer patients.
- Variance in outcomes is greater for 3-Mth Opioid Patients than for All Opioid Patients – again due to smaller patient counts.
- Such large variations in outcomes over time may make it difficult to monitor/manage outcomes for PCPs with few opioid patients.

© 2024 Quanbeck A et al. *JAMA Network Open*.

- Note that some PCPs have missing observations at the end of the study (i.e., they have no qualifying opioid patients after a certain point) – see PCP C above.
- Note that some PCPs have missing observations at the start of the study (i.e., they have no qualifying opioid patients before a certain point, but are engaged with the clinic) – see PCP F above.

© 2024 Quanbeck A et al. *JAMA Network Open*.

- Baseline Descriptive Profile by Health System and Study Arm
  This table provides basic descriptive information at baseline, without delving into initial outcome levels. The columns highlighted in light green show "All Opioid Patient" counts, i.e., in-scope opioid patients satisfying all of the study criteria (e.g., non-cancer, adult, etc.) except for the requirement of opioid use in each of the most recent three calendar months. In-scope patients meeting the 3-month use requirement are referred to as "3-Mth Opioid Patients" and are displayed in the last four light blue columns. The intent is to indicate the impact of applying the 3-month recent use criteria on the number of patients used in the primary analysis. The light green columns can be easily hidden if not needed for the initial paper submission.

Baseline Profile by Study Arm

System	PF	PPC	Clinics	PCPs	PCPs w/ Opioid Patients	All Opioid Patients	All Opioid Patients w/MME	All Opioid Patients w/o MME	%	3-Mth Opioid Patients	3-Mth Opioid Patients w/MME	3-Mth Opioid Patients w/o MME	%	<3-Mth Opioid Patients	<3-Mth Opioid Patients w/MME	<3-Mth Opioid Patients w/o MME	%
**Bellin**	**0**	**0**	5	20	19	951	893	58	6.1%	478	457	21	4.4%	473	436	37	7.8%
**Bellin**	**0**	**1**	4	11	11	384	357	27	7.0%	112	101	11	9.8%	272	256	16	5.9%
**Bellin**	**1**	**0**	4	16	15	613	581	32	5.2%	188	178	10	5.3%	425	403	22	5.2%
**Bellin**	**1**	**1**	5	37	34	1,367	1,287	80	5.9%	370	358	12	3.2%	997	929	68	6.8%
**Bellin Total**			**18**	**84**	**79**	**3,315**	**3,118**	**197**	**5.9%**	**1,148**	**1,094**	**54**	**4.7%**	**2,167**	**2,024**	**143**	**6.6%**
**UW Health**	**0**	**0**	3	40	39	1,476	1,396	80	5.4%	697	669	28	4.0%	779	727	52	6.7%
**UW Health**	**0**	**1**	4	66	64	1,637	1,542	95	5.8%	813	789	24	3.0%	824	753	71	8.6%
**UW Health**	**1**	**0**	4	49	46	1,873	1,781	92	4.9%	797	784	13	1.6%	1,076	997	79	7.3%
**UW Health**	**1**	**1**	3	29	28	677	636	41	6.1%	311	300	11	3.5%	366	336	30	8.2%
**UW Health Total**			**14**	**184**	**177**	**5,663**	**5,355**	**308**	**5.4%**	**2,618**	**2,542**	**76**	**2.9%**	**3,045**	**2,813**	**232**	**7.6%**
**Grand Total**			**32**	**268**	**256**	**8,978**	**8,473**	**505**	**5.6%**	**3,766**	**3,636**	**130**	**3.5%**	**5,212**	**4,837**	**375**	**7.2%**

- While Bellin has more study clinics (18) than UW Health (14), the number of Bellin PCPs (84) is less than half of that for UW Health (184). Similarly, the number of Bellin opioid patients (1,148 with three consecutive months of opioid use) is less than half that for UW Health (2,618).

© 2024 Quanbeck A et al. *JAMA Network Open*.

- Despite randomly assigning clinics within each health system to the study arms, the large variation in clinic size (even within size strata) resulted in significant variation in average clinic size by study arm. This is true whether size is measured in number of PCPs or number of opioid patients.
- This contributes to the uneven distribution by health system of study PCPs within each study arm. For example, the no-intervention arm has 33% of PCPs from Bellin, while the PF/PPC arm has 56% of PCPs from Bellin. To the extent that the two health systems might react to the study interventions differently, such uneven distributions within each arm will likely affect the relative observed changes in outcomes by study arm for the combined data. Note that the primary model includes a fixed effect term allowing the two health systems to have different outcome levels, but does not include any system-specific terms related to the change in outcomes over time associated with the study interventions.
- The requirement that study patients have used opioids for three consecutive months reduces the number of qualifying patients by 65% for Bellin and by 54% for UW Health.
- The percent of 3-month opioid patients with missing opioid MME values ranges from 1.6% to 9.8% by health system and study arm.
- Note that all of these variations by study arm resulted despite the random assignment of clinics to each arm. Other randomizations would likely have resulted in similar levels of variation by arm, but with different arms being “high” or “low” with respect to a given characteristic. The RCT

© 2024 Quanbeck A et al. *JAMA Network Open*.

nature of the study obliges us to use the clinic data as assigned to each study arm in the primary model analysis. Secondary post-hoc analyses can be employed to explore the possible impacts of these uneven characteristics by study arm.

- Ending Descriptive Profile by Health System and Study Arm
  This table provides the same information as the previous baseline table, but as of the final month of the 18-month study intervention period. The intent is to determine which baseline characteristics, if any, have changed significantly over the intervention period. Since the primary analysis uses a fixed PCP cohort, the total number of PCPs remains constant at 268, but the number of PCPs with opioid patients is expected to drop as some PCPs leave the clinic or health system.

Ending Profile by Study Arm

System	PF	PPC	Clinics	PCPs	PCPs w/ Opioid Patients	All Opioid Patients	All Opioid Patients w/MME	All Opioid Patients w/o MME	%	3-Mth Opioid Patients	3-Mth Opioid Patients w/MME	3-Mth Opioid Patients w/o MME	%	<3-Mth Opioid Patients	<3-Mth Opioid Patients w/MME	<3-Mth Opioid Patients w/o MME	%
**Bellin**	**0**	**0**	5	20	19	832	764	68	8.2%	343	333	10	2.9%	489	431	58	11.9%
**Bellin**	**0**	**1**	4	11	10	347	332	15	4.3%	81	80	1	1.2%	266	252	14	5.3%
**Bellin**	**1**	**0**	4	16	14	526	494	32	6.1%	165	160	5	3.0%	361	334	27	7.5%
**Bellin**	**1**	**1**	5	37	33	1,266	1,190	76	6.0%	359	346	13	3.6%	907	844	63	6.9%
**Bellin Total**			**18**	**84**	**76**	**2,971**	**2,780**	**191**	**6.4%**	**948**	**919**	**29**	**3.1%**	**2,023**	**1,861**	**162**	**8.0%**
**UW Health**	**0**	**0**	3	40	32	1,332	1,273	59	4.4%	625	614	11	1.8%	707	659	48	6.8%
**UW Health**	**0**	**1**	4	66	45	1,502	1,440	62	4.1%	728	718	10	1.4%	774	722	52	6.7%
**UW Health**	**1**	**0**	4	49	35	1,722	1,646	76	4.4%	681	675	6	0.9%	1,041	971	70	6.7%
**UW Health**	**1**	**1**	3	29	23	698	670	28	4.0%	313	304	9	2.9%	385	366	19	4.9%
**UW Health Total**			**14**	**184**	**135**	**5,254**	**5,029**	**225**	**4.3%**	**2,347**	**2,311**	**36**	**1.5%**	**2,907**	**2,718**	**189**	**6.5%**
**Grand Total**			**32**	**268**	**211**	**8,225**	**7,809**	**416**	**5.1%**	**3,295**	**3,230**	**65**	**2.0%**	**4,930**	**4,579**	**351**	**7.1%**

© 2024 Quanbeck A et al. *JAMA Network Open*.

- The ending profile is based on data at the end of the 18-month intervention period.
- The number of study PCPs with any opioid patients drops from 248 at baseline to 201 by the end of the intervention period.
- The percent of patients with missing opioid MME values drops from 3.5% at baseline to 2% at the end of the intervention period.
- Other characteristics by arm seem to remain reasonably constant (proportionally) over the study period.

© 2024 Quanbeck A et al. *JAMA Network Open*.

- Baseline outcome measures
  This table summarizes baseline outcome levels, i.e., those observed in study month t=0, the last calendar month prior to implementation of the Practice Facilitation (PF) intervention.

Baseline Outcomes by Study Arm			Bellin					UW Health					Grand Total
			No Interv.	PPC Only	PF Only	PF & PPC	Total	No Interv.	PPC Only	PF Only	PF & PPC	Total	
Average MME Prescribed in Last Quarter	All Opioid Patients	Wgt Avg	32.8	31.7	31.4	31.0	31.7	45.8	44.1	39.4	43.0	42.9	38.7
		Wgt SD	8.5	4.2	4.4	7.7	7.2	15.3	13.5	10.3	9.8	12.9	12.4
	3-Mth Opioid Patients	Wgt Avg	34.8	29.8	28.4	26.0	30.4	51.9	51.4	43.4	49.3	48.8	43.3
		Wgt SD	12.0	11.9	8.0	6.2	10.6	25.1	19.7	16.5	14.3	20.2	19.7
	<3-Mth Opioid Patients	Wgt Avg	30.6	32.4	32.8	33.0	32.4	40.2	36.5	38.1	37.4	38.1	35.7
		Wgt SD	5.3	3.7	4.4	9.0	7.0	7.8	8.6	5.4	11.8	8.0	8.2
Percent w/ MME ≥ 90 mg/day	All Opioid Patients	Wgt Avg	5.3%	5.8%	5.5%	4.2%	4.9%	12.5%	11.4%	9.3%	10.6%	10.9%	8.7%
		Wgt SD	4.2%	3.5%	3.9%	3.5%	3.9%	8.2%	7.8%	5.2%	7.1%	7.2%	6.8%
	3-Mth Opioid Patients	Wgt Avg	4.8%	3.8%	3.3%	2.7%	3.8%	15.9%	14.5%	12.0%	13.0%	13.9%	10.9%
		Wgt SD	4.8%	5.5%	4.2%	3.2%	4.4%	12.9%	10.7%	10.4%	11.3%	11.4%	10.9%
	<3-Mth Opioid Patients	Wgt Avg	5.8%	6.6%	6.5%	4.7%	5.6%	9.4%	8.1%	7.3%	8.4%	8.2%	7.1%
		Wgt SD	4.8%	3.6%	4.7%	4.2%	4.5%	6.6%	8.9%	4.5%	10.6%	7.3%	6.4%
Percent w/ Benzo in Last Quarter	All Opioid Patients	Wgt Avg	12.1%	4.7%	5.2%	8.2%	8.4%	12.8%	11.0%	10.8%	11.2%	11.4%	10.3%
		Wgt SD	7.1%	2.7%	5.1%	6.9%	6.9%	5.6%	7.3%	4.5%	4.7%	5.8%	6.4%
	3-Mth Opioid Patients	Wgt Avg	16.3%	4.5%	4.8%	13.5%	12.4%	15.8%	12.8%	12.5%	13.5%	13.6%	13.2%
		Wgt SD	9.3%	5.0%	6.4%	12.6%	10.9%	9.2%	11.2%	7.9%	8.7%	9.5%	10.0%
	<3-Mth Opioid Patients	Wgt Avg	7.8%	4.8%	5.4%	6.2%	6.2%	10.1%	9.2%	9.1%	9.3%	9.4%	8.1%
		Wgt SD	5.7%	3.6%	6.0%	5.6%	5.6%	4.8%	8.2%	5.8%	5.7%	6.3%	6.2%
Percent w/ Urine Screen in Last Year	All Opioid Patients	Wgt Avg	12.7%	15.6%	19.1%	7.8%	12.2%	23.0%	18.5%	10.6%	20.5%	17.3%	15.4%
		Wgt SD	18.5%	14.2%	18.4%	5.0%	14.6%	12.9%	14.7%	10.6%	12.2%	13.6%	14.2%

© 2024 Quanbeck A et al. *JAMA Network Open*.

	3-Mth Opioid Patients	Wgt Avg	21.8%	33.9%	42.0%	16.8%	24.7%	40.7%	32.6%	21.8%	37.6%	32.1%	29.8%
		Wgt SD	27.9%	19.5%	28.0%	14.9%	25.4%	20.9%	21.0%	20.3%	21.3%	22.1%	23.4%
	<3-Mth Opioid Patients	Wgt Avg	3.6%	8.1%	8.9%	4.5%	5.6%	7.1%	4.6%	2.1%	6.0%	4.5%	5.0%
		Wgt SD	4.5%	9.8%	10.7%	4.0%	7.1%	7.3%	7.7%	2.7%	8.4%	6.7%	6.9%
**Percent w/ Treatment Agreement in Last Year**	All Opioid Patients	Wgt Avg	21.5%	17.7%	19.6%	9.8%	15.9%	6.0%	5.5%	3.7%	11.2%	5.7%	9.5%
		Wgt SD	14.2%	10.6%	19.9%	8.6%	14.2%	8.7%	8.2%	3.8%	9.0%	7.7%	11.6%
	3-Mth Opioid Patients	Wgt Avg	31.8%	42.0%	41.5%	20.3%	30.7%	9.0%	9.5%	6.1%	19.3%	9.5%	16.0%
		Wgt SD	17.4%	14.3%	30.7%	16.7%	21.4%	14.8%	13.3%	7.9%	15.0%	13.2%	18.8%
	<3-Mth Opioid Patients	Wgt Avg	11.0%	7.7%	9.9%	5.9%	8.0%	3.2%	1.6%	1.3%	4.4%	2.2%	4.6%
		Wgt SD	11.3%	6.0%	10.7%	5.7%	8.6%	5.4%	3.1%	1.7%	7.0%	4.3%	7.1%
**Percent w/ PHQ-8/9 in Last Year**	All Opioid Patients	Wgt Avg	19.0%	24.2%	24.3%	31.8%	25.9%	28.9%	22.2%	23.2%	19.5%	23.9%	24.7%
		Wgt SD	12.8%	12.1%	19.6%	18.9%	17.6%	14.5%	11.7%	10.3%	9.3%	12.2%	14.5%
	3-Mth Opioid Patients	Wgt Avg	17.8%	23.2%	21.8%	31.4%	23.3%	34.9%	23.5%	25.6%	21.2%	26.9%	25.8%
		Wgt SD	13.2%	18.5%	22.6%	24.2%	20.3%	19.8%	15.2%	13.6%	14.5%	16.8%	18.0%
	<3-Mth Opioid Patients	Wgt Avg	20.3%	24.6%	25.4%	32.0%	27.2%	23.5%	20.9%	22.1%	18.0%	21.6%	24.0%
		Wgt SD	13.5%	10.3%	20.6%	18.5%	17.8%	13.1%	13.5%	12.0%	12.1%	12.8%	15.3%
**Percent w/ PEG3 in Last Year**	All Opioid Patients	Wgt Avg	0.0%	0.0%	0.0%	0.1%	0.1%	12.1%	6.4%	2.1%	0.9%	5.8%	3.7%
		Wgt SD	0.0%	0.0%	0.0%	0.6%	0.4%	13.7%	11.3%	4.0%	1.7%	10.5%	8.8%
	3-Mth Opioid Patients	Wgt Avg	0.0%	0.0%	0.0%	0.3%	0.1%	20.8%	10.5%	3.3%	1.3%	9.9%	6.9%
		Wgt SD	0.0%	0.0%	0.0%	0.8%	0.5%	23.4%	16.7%	6.2%	2.0%	17.3%	15.1%
	<3-Mth Opioid Patients	Wgt Avg	0.0%	0.0%	0.0%	0.1%	0.0%	4.2%	2.3%	0.7%	0.5%	2.0%	1.2%
		Wgt SD	0.0%	0.0%	0.0%	0.5%	0.4%	7.3%	4.6%	1.7%	1.7%	4.8%	3.8%

- The “Wgt Avg” rows display average PCP baseline outcomes, weighted by the number of opioid patients associated with each PCP. The “Wgt SD” rows display the weighted PCP-to-PCP standard deviation of outcomes. These standard deviations indicate wide variation in outcomes from PCP to PCP.

© 2024 Quanbeck A et al. *JAMA Network Open*.

- Bellin has consistently lower prescribed levels of opioids than UW Health, both in terms of average MME and the percent of patients with MME greater than or equal to 90 mg per day.
- For UW Health, average MME and percent of patients with MME ≥ 90 mg/day are somewhat greater for 3-month opioid patients than for all in-scope opioid patients. The reverse is the case for Bellin. Note that some in-scope opioid patients that do not satisfy the 3-month opioid use criteria may not have any opioid prescriptions in the 3-month period used to compute the MME values for these two outcomes. For these patients, the measures are treated as missing (i.e., the values are not set to zero) and the patients are excluded from the outcome calculations. So, the MME-based "All Opioid Patient" outcomes might better be described as "All Opioid Patients w/ At Least 1 Opioid Order in the Last 3 Months". Ditto for the Benzo co-prescribing outcome.
- As expected, urine drug screening, PEG-3 pain screening, and treatment agreements rates are greater for 3-month opioid patients than for all in-scope opioid patients. PHQ-8/9 depression screening rates are about the same for 3-month and all opioid patients.
- Treatment agreement rates are higher for Bellin than for UW Health opioid patients.
- PEG-3 pain screening rates for Bellin are nearly zero at baseline, while UW Health rates vary significantly from arm to arm.
- Other baseline outcomes are similar for Bellin and UW Health.
- The variation in baseline outcomes by health system is accommodated in the primary model by a fixed effect intercept adjustment.
- There is also variation in baseline outcomes within each health system by study arm. As with other baseline characteristics, this variation occurs, despite the random assignment of clinics to study arms, due to the high levels or variation in baseline outcome levels from clinic to clinic and, within clinic, from PCP to PCP. The primary model includes clinic-level and PCP-level random intercept effects to accommodate these baseline differences.

© 2024 Quanbeck A et al. *JAMA Network Open*.

Primary Data Model

$Y(ijkt)$ = outcome value for study month t, health system i, clinic j, PCP k

$= \eta(i) + \alpha\, x(ij) + \beta_1 t_7 + \beta_2 PF(ij)\, t_7 + \beta_3 t_{13} + \beta_4 PF(ij)\, t_{13} + \beta_5 PPC(ij)\, t_{13} + \beta_6 PF(ij)\, PPC(ij)\, t_{13}$

$+ u(ij) + v_0(ijk) + v_1(ijk)\, t_7 + v_2(ijk)\, t_{13} + \varepsilon(ijkt)$,

where,

$\eta(i)$ = baseline fixed effect intercept for health system i, where i = 1 (UW Health) or 2 (Bellin)

α = fixed effect coefficient for impact of baseline clinic size

$x(ij)$ = baseline clinic size, i.e., opioid patient count for clinic ij minus statewide average opioid patient count

$t_7 = \max(0, t-7)$, i.e., months from study month 7 (month of clinic PF randomization)

$t_{13} = \max(0, t-13)$, i.e., months from study month 13 (month of clinic PPC randomization)

$PF(ij) = 1$, if clinic ij is randomized to receive PF

$PPC(ij) = 1$, if clinic ij is randomized to receive PPC

$\beta_1, \ldots, \beta_6$ = fixed effect monthly slopes

$u(ij)$ = random effect intercept for clinic ij and $u(ij) \sim \text{iid } N(0, \sigma_u^2)$

$v_0(ijk)$, $v_1(ijk)$ and $v_2(ijk)$ = random effect intercept and slopes for PCP ijk, and,

$\underline{v}(ijk) \sim \text{iid } N(0, \Sigma_v)$, where vector $\underline{v}(ijk) = [v_0(ijk), v_1(ijk), v_2(ijk)]'$

$\varepsilon(ijkt)$ = random error for PCP ijk at time t, where $\varepsilon(ijkt) \sim \text{iid } N(0, \sigma_\varepsilon^2/n(ijkt))$ and

$n(ijkt)$ = opioid patient count associated with $Y(ijkt)$

- Comments on model structure
  - Assumes a fixed effect piecewise linear spline with knots at t=7 and t=13, where the slopes depend on the interventions (PF and/or PPC) applicable to the clinic
    - The knots correspond to the advent of study interventions.
    - An interaction term is included to facilitate testing the hypothesis of additive intervention effects on/after t=13.
  - Includes baseline fixed effect intercept adjustments for the health system and the clinic size

© 2024 Quanbeck A et al. *JAMA Network Open*.

- The health system intercept impact is modeled as a fixed effect rather than a random effect, since there are only two health systems in the study.
- The clinic size term was included because clinic size was a stratification variable that might have an impact on outcomes.
- Note that these terms cancel when considering changes in outcome values over time. So, these terms have no direct role in explaining the impact of the study interventions.

© 2024 Quanbeck A et al. *JAMA Network Open*.

- The random effects determine the correlation structure of the observed values
    - As with the health-system-level adjustments, the clinic-level random effects are limited to intercept adjustments. Given the presence of PCP-level random intercept and slope effects, it was found that clinic-level intercept effects were modest and clinic-level slope effects were not needed. Again, the clinic-level random intercept effects cancel when considering changes in outcomes over time.
    - The PCP-level random intercept effect and two slope effects are assumed to be iid Normal with variance-covariance matrix Σ_v, where the intercept and slope effects for a specific PCP are allowed to be correlated, as determined by the data in the model fitting. For example, the intercept and slope effects might be negatively correlated so that a positive deviation in the outcome level for a PCP level would generally be associated with a negative deviation in one or both of the slopes.
    - The random PCP-level intercept generates positive correlation among the outcome observations over time from the same PCP. Without the random PCP-level slope effects, this correlation would be roughly constant regardless of the time interval between the observations. The random PCP-level slope effects generally cause the positive correlation to shrink as the time interval increases.
    - The random PCP-level slope effects also model the variation in intervention impact from PCP to PCP. As with the system-level and clinic-level intercept effects, the PCP-level intercept effects cancel out when considering any changes in outcomes over time.
    - The fixed effect slope estimates are based, in large part, on the observed quarterly changes in outcomes for each PCP. Without the random slope effects, such quarterly changes would each be viewed by the model (after accounting for the differences in independent error terms) as an independent estimator of the underlying fixed effect slope. The random slope structure introduces positive correlation among quarterly changes from the same PCP (again, as determined by the data in model fitting), reducing the effective sample size (power) of the resulting slope estimates, resulting in larger p-values for tests of intervention impact.
- Note that all random effects are assumed to have variances unaffected by the number of patients associated with the clinic or PCP. This structure could be generalized in future modeling to allow the variance, in whole or part, to be inversely proportional to the number of opioid patients managed by the clinic or PCP. This could be accomplished by dividing the spline component (i.e., the constant 1 column or the t_7 and/or t_{13} columns in the design matrix) by the square root of the number of opioid patients for the clinic or PCP.

  So, $u(ij) + v_0(ijk) + v_1(ijk)\ t_7 + v_2(ijk)\ t_{13}$ might become,

  $u(ij)/n(ij)^{1/2} + [v_0(ijk) + v_1(ijk)\ t_7 + v_2(ijk)\ t_{13}]/n(ijk)^{1/2}$,

  where n(ij) is the average opioid patient count for clinic ij, and,
  n(ijk) is the average opioid patient count for PCP ijk, and,
  the variances of u() and v() are constant wrt n(ij) and n(ijk).

- The model error term does assume the variance is inversely proportional to the number of opioid patients underlying each observation. All of the outcome measures are averages of values for each underlying opioid patient. If the patient-to-patient outcome variation from the model fixed and random effects are assumed independent, then this variance structure is appropriate. In the absence of random effects, this would result in fixed effect estimates based on weighted averages of system/clinic/PCP outcomes, where the weight assigned to each value would be the opioid patient count in the denominator of the measure. With the random effects having variances that don’t vary by clinic or PCP, the model uses a blend of unweighted and weighted averages.

© 2024 Quanbeck A et al. *JAMA Network Open*.

© 2024 Quanbeck A et al. *JAMA Network Open*.

- Model Estimation
    - SAS Proc Mixed was used to estimate the model for each outcome measure
        - Average MME per day for most recent 3 months
        - Percent of patients with average MME greater than or equal to 90 mg/day
        - Percent of patients with urine drug screen in last 12 months
        - Percent of patients with treatment in last 12 months
        - Percent of patients with PHQ-9 depression screen in last 12 months
        - Percent of patients with PEG-3 pain screen in last 12 months
    - The same model structure was employed with each outcome measure
        - See model structure above
        - Method = REML, residual/restricted maximum likelihood estimation, which produces less biased variance/covariance parameter estimates than conventional maximum likelihood estimation
        - Intervention interaction term was retained throughout, even if found to be statistically insignificant
    - Best Linear Unbiased Predictors (BLUPs) can be generated for each PCP, if needed for secondary analyses. This might be a useful basis for obtaining estimates for missing values.

© 2024 Quanbeck A et al. *JAMA Network Open*.

- Table of Intervention Effect Estimates

Estimated Impact of Study Interventions (3-Mth Opioid Patients)									Aggregated p-values		
Contrast	Item	Avg MME	MME >= 90 mg/day	Benzo Use	Urine Screening	Treatment Agreement	PHQ-9 Screening	PEG-3 Screening	Min p-value	Number of p-values ≤ 5%	Geom. Mean p-value
**No-Intervention 18-Mth Chg**	**Estimate**	-1.50	-1.9%	-1.1%	13.3%	16.2%	16.5%	4.5%	*0.07%*	*0.02%*	*0.00%*
	**Std Dev**	1.28	0.9%	1.0%	2.8%	3.1%	3.0%	2.3%			
	**p-value**	0.2425	0.0249	0.2936	<.0001	<.0001	<.0001	0.0547	0.01%	4	0.52%
**PF Only 18-Mth Chg**	**Estimate**	-1.30	-1.3%	-0.5%	2.3%	11.3%	13.3%	17.7%	*0.07%*	*0.38%*	*0.00%*
	**Std Dev**	1.36	0.9%	1.1%	2.9%	3.2%	3.1%	2.4%			
	**p-value**	0.3380	0.1605	0.6505	0.4226	0.0004	<.0001	<.0001	0.01%	3	1.29%
**PF Only vs No-Intervention**	**Estimate**	0.20	0.7%	0.6%	-11.0%	-4.9%	-3.2%	13.2%	*0.07%*	*4.44%*	*0.16%*
	**Std Dev**	1.83	1.2%	1.5%	4.0%	4.3%	4.2%	3.3%			
	**p-value**	0.9145	0.5899	0.6962	0.0064	0.2576	0.4502	<.0001	0.01%	2	8.33%
**PPC Only 18-Mth Chg**	**Estimate**	-0.35	-0.5%	-1.1%	8.2%	3.5%	1.3%	-0.3%	*2.36%*	*30.17%*	*17.08%*
	**Std Dev**	1.32	0.9%	1.1%	2.8%	3.0%	3.0%	2.3%			
	**p-value**	0.7924	0.5993	0.3298	0.0034	0.2528	0.6606	0.9082	0.34%	1	26.02%
**PPC Only vs No-Intervention**	**Estimate**	1.15	1.5%	0.0%	-5.1%	-12.7%	-15.2%	-4.8%	*0.07%*	*4.44%*	*0.00%*
	**Std Dev**	1.60	1.2%	1.4%	3.7%	3.7%	3.8%	2.8%			
	**p-value**	0.4721	0.1999	0.9943	0.1648	0.0007	<.0001	0.0869	0.01%	2	3.70%
**PF Only (18 mths) vs PPC Only (12 mths)**	**Estimate**	-0.96	-0.8%	0.6%	-5.9%	7.8%	12.0%	17.9%	*0.07%*	*4.44%*	*0.02%*
	**Std Dev**	1.85	1.2%	1.5%	4.0%	4.3%	4.2%	3.3%			
	**p-value**	0.6065	0.5118	0.7100	0.1431	0.0689	0.0046	<.0001	0.01%	2	5.18%
**PF Only (12 mths) vs PPC Only (12 mths)**	**Estimate**	-0.18	-0.4%	-0.2%	-6.8%	11.1%	12.6%	12.9%	*0.07%*	*0.38%*	*0.00%*
	**Std Dev**	1.68	1.2%	1.6%	3.8%	3.9%	3.9%	2.8%			
	**p-value**	0.9153	0.7369	0.9228	0.0790	0.0044	0.0012	<.0001	0.01%	3	3.07%
**PF & PPC 18-Mth Chg**	**Estimate**	-2.42	-1.3%	-0.3%	-1.1%	2.3%	11.2%	8.6%	*0.21%*	*4.44%*	*0.01%*
	**Std Dev**	1.40	0.9%	1.2%	2.9%	3.2%	3.1%	2.4%			
	**p-value**	0.0847	0.1541	0.7872	0.7053	0.4586	0.0004	0.0003	0.03%	2	4.54%
**PF & PPC vs No-Intervention**	**Estimate**	-0.93	0.6%	0.8%	-14.4%	-13.8%	-5.3%	4.1%	*0.28%*	*4.44%*	*0.06%*
	**Std Dev**	1.86	1.3%	1.5%	4.0%	4.3%	4.3%	3.3%			
	**p-value**	0.6195	0.6339	0.6180	0.0004	0.0014	0.2133	0.2178	0.04%	2	6.74%
**PF & PPC Interaction**	**Estimate**	-2.27	-1.5%	0.2%	1.6%	3.7%	13.1%	-4.3%	*11.12%*	*30.17%*	*25.20%*
	**Std Dev**	2.35	1.7%	2.1%	5.3%	5.4%	5.5%	4.0%			
	**p-value**	0.3332	0.3584	0.9331	0.7571	0.4871	0.0167	0.2850	1.67%	1	29.53%

  - The row categories within the table correspond to the estimated change in the fixed effect curve from baseline to the end of the intervention period (18 months later) within and between the four study arms. Each category includes three detail rows, showing the estimated 18-month change (or the difference in 18-month changes from one arm to another), the standard deviation of the estimated value, and the p-value of the estimate (i.e., testing the null hypothesis that the true value is zero). Significant p-values (i.e., those less than or equal to 5%) are highlighted in gray and the corresponding estimated changes (or differences in changes) are highlighted in green (if the direction of the change is favorable) or orange (if the direction of the change is unfavorable).
  - Note that the final row category focuses on the magnitude and statistical significance of the model interaction term. If this term can be taken to be zero, the two intervention effects are considered additive.
  - The columns of the table focus on each of the seven primary model outcomes. The final columns provide three different aggregate p-values that can be used to assess the null hypothesis that the true changes (or differences in changes) for all seven of the row category outcomes are

© 2024 Quanbeck A et al. *JAMA Network Open*.

uniformly zero.

© 2024 Quanbeck A et al. *JAMA Network Open*.

- Although the PHQ-9 screening outcome has an interaction term p-value less than 5% (i.e., 1.67%), all three of the aggregated p-values for the test that all interaction effects are zero are greater than 10%. That is, observing one of seven outcome p-values below 5% is not unexpected even if all seven of the true effects are zero. So, we can't reject the assumption that the PF and PPC intervention effects, if any, are additive.
- Note that each of the other row categories (other than the interaction term category) have at least one significant aggregate p-value, indicating that at least one of the outcomes has a statistically significant estimated change (or difference in change). We now consider each of the seven outcomes for the "3-Mth Opioid Patient" population.

© 2024 Quanbeck A et al. *JAMA Network Open*.

- Average MME
    - There are no statistically significant changes in average MME levels from baseline to the end of the intervention period for any study arm or difference in study arms.
    - All four study arms had estimated changes that were negative (but not statistically significant).
- Percent of patients with MME less than or equal to 90 mg per day
    - Only the baseline arm had a statistically significant reduction (-1.9% with p-value of 2.5%).
    - The remaining three arms had insignificant reductions.
- Percent of patients with co-prescribed Benzodiazepine in the last quarter
    - Similar to average MME levels, there were estimated reductions for all four arms, but none of these reductions were statistically significant.
- Percent of patients with urine drug screening in the last 12 months
    - The no-intervention arm and the PPC-only arm both had significant increases in drug screening rates (13.3% and 8.2%, respectively). The PF-only and PF&PPC arms had no significant change.
    - Consequently, the difference between the PF-only arm and the no-intervention arm changes was negative (-11.0%) and significant (p-value of 0.6%).
    - Similarly, the difference between the PF&PPC arm and the no-intervention arm changes was negative (-14.4%) and significant (p-value of 0.04%).
- Percent of patients with a treatment agreement in the last 12 months
    - The no-intervention arm and the PF-only arm both had significant increases in treatment agreement rates (16.2% and 11.3%, respectively). The PPC-only and PF&PPC arms had no significant change.
    - Consequently, the difference between the PPC-only arm and the no-intervention arm changes was negative (-12.7%) and significant (p-value of 0.07%).
    - Similarly, the difference between the PF&PPC arm and the no-intervention arm changes was negative (-13.8%) and significant (p-value of 0.14%).
- Percent of patients with a PHQ-8/9 depression screening in last 12 months
    - The no-intervention arm, the PF-only arm and the PF&PPC arms all had significant increases in depression screening rates (16.5%, 13.3% and 11.2%, respectively). The PPC-only arm had no significant change.
    - Consequently, the difference between the PPC-only arm and the no-intervention arm changes was negative (-15.2%) and significant (p-value < 0.01%).
    - The differences between the PF-only, PF&PPC and the no-intervention arm changes were not statistically significant.
- Percent of patients with a PEG-3 pain screening in the last 12 months
    - The PF-only and PF&PPC arms had significant improvements (17.7% and 8.6%, respectively) in pain screening rates.
    - The no-intervention arm had an insignificant improvement and the PPC-only arm had an insignificant decrease.
    - The PF-only arm improvement was significantly greater than the no-intervention arm (13.2% with a p-value < 0.01%).

© 2024 Quanbeck A et al. *JAMA Network Open*.

- The PF&PPC arm improvement was not significantly greater than the no-intervention arm improvement (4.1% with a p-value of 22%).

© 2024 Quanbeck A et al. *JAMA Network Open*.

Estimated Impact of Study Interventions (All Opioid Patients)									Aggregated p-values		
**Contrast**	**Item**	**Avg MME**	**MME >= 90 mg/day**	**Benzo Use**	**Urine Screening**	**Treatment Agreement**	**PHQ-9 Screening**	**PEG-3 Screening**	**Min p-value**	**Number of p-values ≤ 5%**	**Geom. Mean p-value**
**No-Intervention 18-Mth Chg**	**Estimate**	-1.09	-0.9%	-0.8%	6.8%	7.7%	13.6%	2.0%	***0.07%***	***0.38%***	***0.00%***
	**Std Dev**	0.71	0.6%	0.6%	1.6%	1.6%	2.1%	1.8%			
	**p-value**	0.1276	0.1214	0.1806	<.0001	<.0001	<.0001	0.2682	0.01%	3	0.69%
**PF Only 18-Mth Chg**	**Estimate**	-2.39	-1.2%	-0.6%	0.5%	4.6%	11.8%	12.3%	***0.07%***	***0.00%***	***0.00%***
	**Std Dev**	0.70	0.5%	0.6%	1.5%	1.6%	2.1%	1.8%			
	**p-value**	0.0007	0.0229	0.3052	0.7319	0.0049	<.0001	<.0001	0.01%	5	0.56%
**PF Only vs No-Intervention**	**Estimate**	-1.31	-0.4%	0.2%	-6.3%	-3.1%	-1.9%	10.2%	***0.07%***	***4.44%***	***0.03%***
	**Std Dev**	0.96	0.7%	0.8%	2.2%	2.3%	3.0%	2.6%			
	**p-value**	0.1757	0.6028	0.8160	0.0037	0.1668	0.5304	<.0001	0.01%	2	6.01%
**PPC Only 18-Mth Chg**	**Estimate**	-2.72	-2.0%	-0.9%	4.2%	1.7%	5.4%	-0.1%	***0.21%***	***0.02%***	***0.00%***
	**Std Dev**	0.74	0.6%	0.7%	1.5%	1.6%	2.1%	1.8%			
	**p-value**	0.0003	0.0006	0.1626	0.0061	0.2973	0.0102	0.9751	0.03%	4	1.76%
**PPC Only vs No-Intervention**	**Estimate**	-1.64	-1.1%	-0.1%	-2.7%	-6.1%	-8.2%	-2.1%	***0.98%***	***4.44%***	***0.02%***
	**Std Dev**	0.92	0.7%	0.8%	2.0%	1.9%	2.6%	1.9%			
	**p-value**	0.0766	0.1254	0.9138	0.1834	0.0018	0.0014	0.2673	0.14%	2	5.24%
**PF Only (18 mths) vs PPC Only (12 mths)**	**Estimate**	0.33	0.8%	0.3%	-3.7%	2.9%	6.3%	12.3%	***0.07%***	***4.44%***	***0.07%***
	**Std Dev**	0.99	0.8%	0.9%	2.1%	2.2%	3.0%	2.6%			
	**p-value**	0.7380	0.3179	0.7416	0.0888	0.1883	0.0321	<.0001	0.01%	2	7.13%
**PF Only (12 mths) vs PPC Only (12 mths)**	**Estimate**	0.52	0.4%	0.8%	-4.6%	5.2%	6.5%	8.5%	***0.07%***	***0.02%***	***0.00%***
	**Std Dev**	0.97	0.8%	0.9%	2.0%	1.9%	2.6%	1.9%			
	**p-value**	0.5948	0.5976	0.3578	0.0227	0.0077	0.0113	<.0001	0.01%	4	3.06%
**PF & PPC 18-Mth Chg**	**Estimate**	-3.49	-1.4%	-0.2%	-0.4%	1.1%	13.5%	7.4%	***0.07%***	***0.02%***	***0.00%***
	**Std Dev**	0.73	0.6%	0.6%	1.5%	1.6%	2.1%	1.8%			
	**p-value**	<.0001	0.0131	0.7715	0.7840	0.5087	<.0001	<.0001	0.01%	4	0.88%
**PF & PPC vs No-Intervention**	**Estimate**	-2.40	-0.6%	0.6%	-7.3%	-6.7%	-0.1%	5.4%	***0.56%***	***0.02%***	***0.01%***
	**Std Dev**	0.99	0.8%	0.9%	2.2%	2.2%	3.0%	2.6%			
	**p-value**	0.0152	0.4536	0.4607	0.0008	0.0031	0.9753	0.0350	0.08%	4	4.29%
**PF & PPC Interaction**	**Estimate**	0.54	1.0%	0.5%	1.7%	2.5%	10.0%	-2.7%	***4.06%***	***30.17%***	***14.45%***
	**Std Dev**	1.30	1.0%	1.2%	2.8%	2.7%	3.6%	2.7%			
	**p-value**	0.6756	0.3601	0.6522	0.5462	0.3582	0.0059	0.3081	0.59%	1	24.72%

- Note that each of row categories (including the interaction term category) have at least one significant aggregate p-value, indicating that at least one of the outcomes has a statistically significant estimated change (or difference in change). We now consider each of the seven outcomes for the "All Opioid Patient population".
  - Average MME
    - There is a negative, but not statistically significant, change in average MME level from baseline to the end of the intervention period for the no-intervention study arm. All three of the intervention study arms have statistically significant reductions in average MME. Only the difference between the PF & PPC arm reduction and the no-intervention arm reduction is statistically significant.
  - Percent of patients with MME less than or equal to 90 mg per day
    - There is a negative, but not statistically significant, change in the percent of patients with MME above 90 mg per day from baseline to the end of the intervention period for the no-intervention study arm. All three of the intervention study arms have

© 2024 Quanbeck A et al. *JAMA Network Open*.

statistically significant reductions in the percent of patients above 90 mg/day. However, none of the differences between the intervention arm reductions and the no-intervention arm reduction are statistically significant.
    - The remaining three arms had insignificant reductions.
- Percent of patients with co-prescribed Benzodiazepine in the last quarter
    - There are estimated reductions for all four arms, but none of these reductions are statistically significant.
- Percent of patients with urine drug screening in the last 12 months
    - The no-intervention arm and the PPC-only arm both had significant increases in drug screening rates (6.8% and 4.2%, respectively). The PF-only and PF&PPC arms had no significant change.
    - The difference between the PPC-only arm and the no-intervention arm was negative, but statistically insignificant.
    - The difference between the PF-only arm and the no-intervention arm changes was negative (-6.3%) and significant (p-value of 0.4%).
    - Similarly, the difference between the PF&PPC arm and the no-intervention arm changes was negative (-7.3%) and significant (p-value of 0.08%).
- Percent of patients with a treatment agreement in the last 12 months
    - The no-intervention arm and the PF-only arm both had significant increases in treatment agreement rates (7.7% and 4.6%, respectively). The PPC-only and PF&PPC arms had no significant change.
    - The difference between the PF-only arm and the no-intervention arm was negative, but statistically insignificant.
    - Consequently, the difference between the PPC-only arm and the no-intervention arm changes was negative (-6.1%) and significant (p-value of 0.18%).
    - Similarly, the difference between the PF&PPC arm and the no-intervention arm changes was negative (-6.7%) and significant (p-value of 0.31%).
- Percent of patients with a PHQ-8/9 depression screening in last 12 months
    - The no-intervention arm, the PF-only arm, the PPC-only, and the PF&PPC arms all had significant increases in depression screening rates (13.6%, 11.8%, 5.4% and 13.5%, respectively).
    - The difference between the PPC-only arm and the no-intervention arm was negative (-8.2%) and statistically significant (p-value of 0.14%).
    - The differences between the PF-only, PF&PPC and the no-intervention arm changes were not statistically significant.
- Percent of patients with a PEG-3 pain screening in the last 12 months
    - The PF-only and PF&PPC arms had significant improvements (12.3% and 7.4%, respectively) in pain screening rates.
    - The no-intervention arm had an insignificant improvement and the PPC-only arm had an insignificant decrease.
    - The PF-only arm improvement was significantly greater than the no-intervention arm (10.2% with a p-value < 0.01%).
    - The PF&PPC arm improvement was also significantly greater than the no-intervention arm improvement (5.4% with a p-value of 3.5%).

© 2024 Quanbeck A et al. *JAMA Network Open*.

Appendix A – Primary Model Results (3-Mth Opioid Patients)

- This appendix summarizes key model results for each outcome measure
    - Graphs of PCP outcome changes over the 18-month intervention period by study arm
        - Outcome change for each PCP vs. number of opioid patients for PCP
        - Cumulative distribution function of PCP outcome change, weighted by number of opioid patients for each PCP
    - Time series plot of weighted average quarterly outcomes by study arm
        - Average plus/minus 1 standard deviation (standard deviation of weighted average assuming independent patients within PCP) This provides some sense of the reliability (or lack thereof) of any exhibited trends without requiring any model assumptions
        - Note that the four study arms are grouped into two arms (PF vs. non-PF) in the first six months when only the PF intervention is being applied.
        - Baseline values for each PCP are subtracted to display changes from baseline to each quarterly observation date.
    - Table and time series plot of fitted fixed effect trends (again, changes from baseline)
        - Table includes standard deviation of quarterly fixed effect estimates
        - Table includes differences between intervention arm results and the no-intervention arm, including standard deviations and t-ratios for the differences
        - Time series plot only shows fitted fixed-effect spline for each study arm
    - SAS Proc Mixed output tables and graphs
        - Random effect and error term variance/covariance estimates
        - Fixed effect estimates
        - Variety of fixed effect contrasts relating to the significance of intervention effects
        - Residual plots (marginal and conditional)
            - Marginal residuals are estimates of $u(ij) + v_0(ijk) + v_1(ijk)\ t_7 + v_2(ijk)\ t_{13} + \varepsilon(ijkt)$.
            - Conditional residuals are estimates of $\varepsilon(ijkt)$.

© 2024 Quanbeck A et al. *JAMA Network Open*.

Appendix A1 – Average MME Prescribed in Last 3 Months

The first graph below shows a scatter plot of study PCP values. The horizontal axis is the observed (not fitted) change in the outcome (average MME in this case) from baseline to the end of the 18-month intervention period. Values on this axis range from a decrease of 50 mg/day to an increase of 50 mg/day. The vertical axis displays the number of opioid patients associated with the PCP. The color of the plotted points indicates the study arm to which the PCP is assigned. The intent is to provide a visual sense of the variation of outcome changes observed by PCP and how that variation is affected by study arm and the size of the PCP's opioid patient cohort.

Note that the arms are labeled "00" for the no-intercept arm, "01" for the PPC-only arm, "10" for the PF-only arm and "11" for the PF & PPC arm.

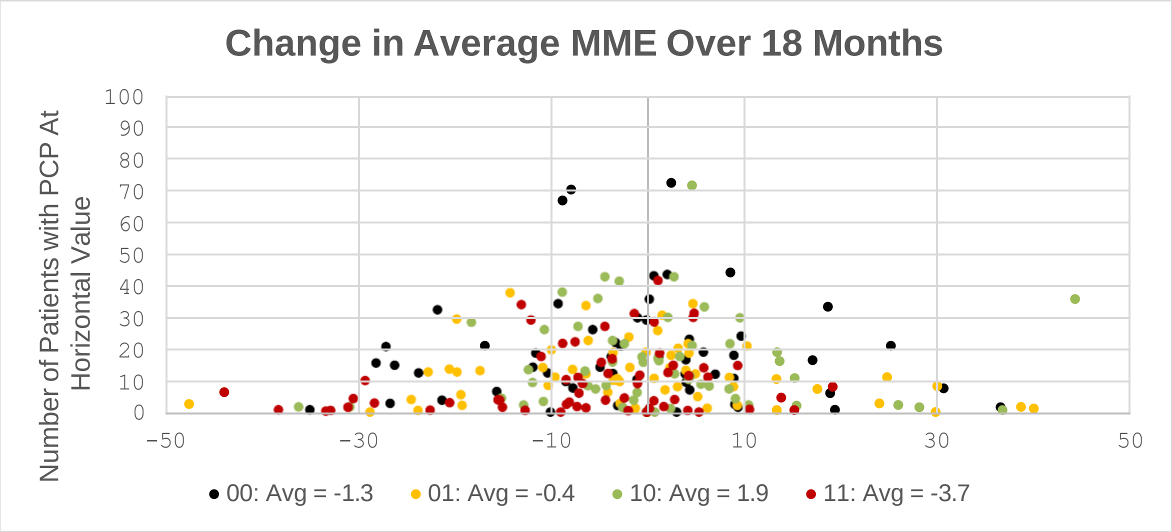

To provide a better sense of the difference in the distribution of PCP outcome changes, the following graph shows the cumulative distribution function for each of the four study arms.

© 2024 Quanbeck A et al. *JAMA Network Open*.

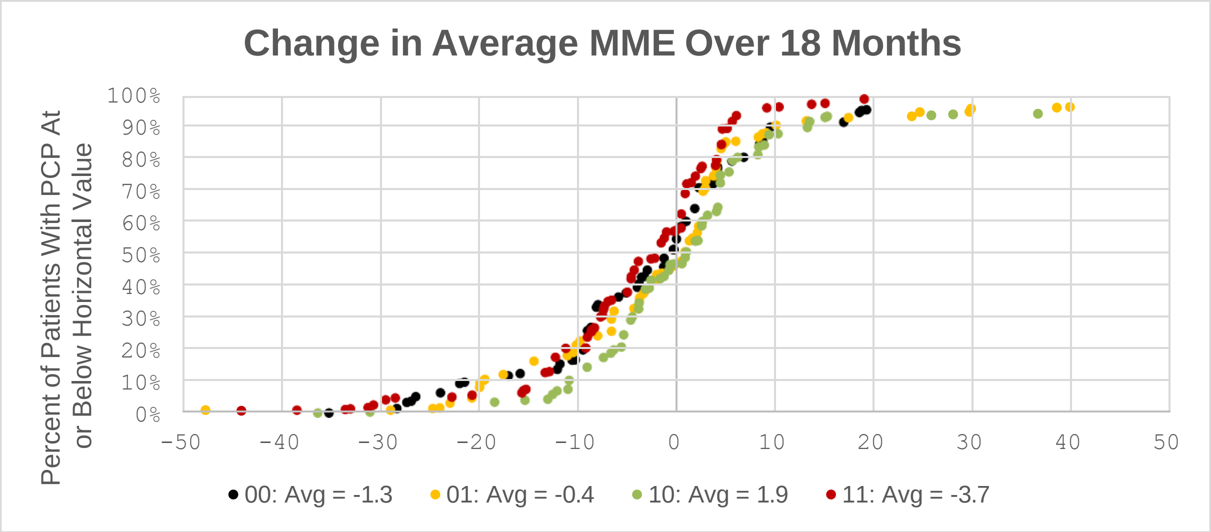

All four arms exhibit a similar wide-ranging distribution of average MME changes from t=0 to t=18. Nevertheless, there are some modest differences. For example, the red dots tends to be further to the left, indicating that the PF & PPC arm has slightly smaller (more negative) changes in average MME compared to PCPs in other study arms.

The graph below displays the progression of changes in observed average MME values by arm over time. There are two lines for each arm, representing the arm average observed change in MME plus or minus one standard deviation. So, for example, the two red lines indicate that at t=3 roughly 2/3 of the observed changes in average MME for PCPs in the PF & PPC study arm were in the range from -1.2 mg/day to 0.5 mg/day. By t=18, this 2/3 capture band shifted to a range from -4.5 mg/day to -0.5 mg/day. The average result for each arm is the mid-point of the band. The intent is to convey the trend for each arm while also showing the overlapping range of PCP-specific values.

Note that the no-intervention and PPC-only bands are combined through t=6, since no intervention implemented for either during this period. Similarly, the PF-only and PF & PPC arms are combined through t=6, since both are subject to only the PF intervention during this period.

© 2024 Quanbeck A et al. *JAMA Network Open*.

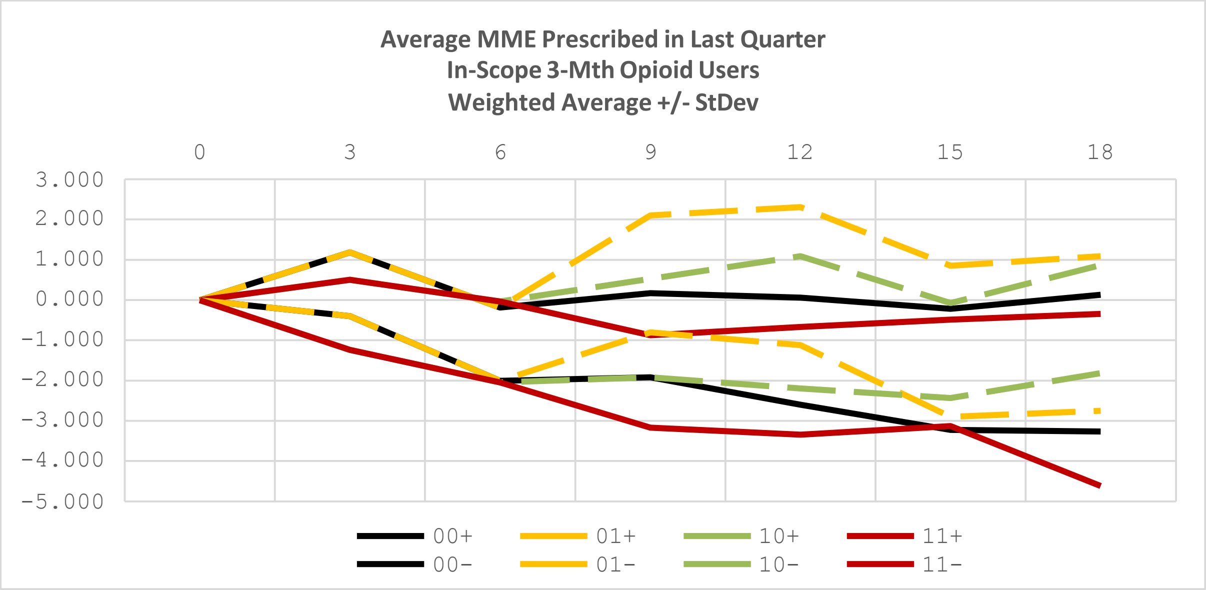

The graph below shows the fitted model estimates for the change in outcome by arm. These fitted curves can be compared to the progression of observed outcomes in the previous graph to provide a sense of the variation of the PCP-level source data to which the estimated curves are fitted.

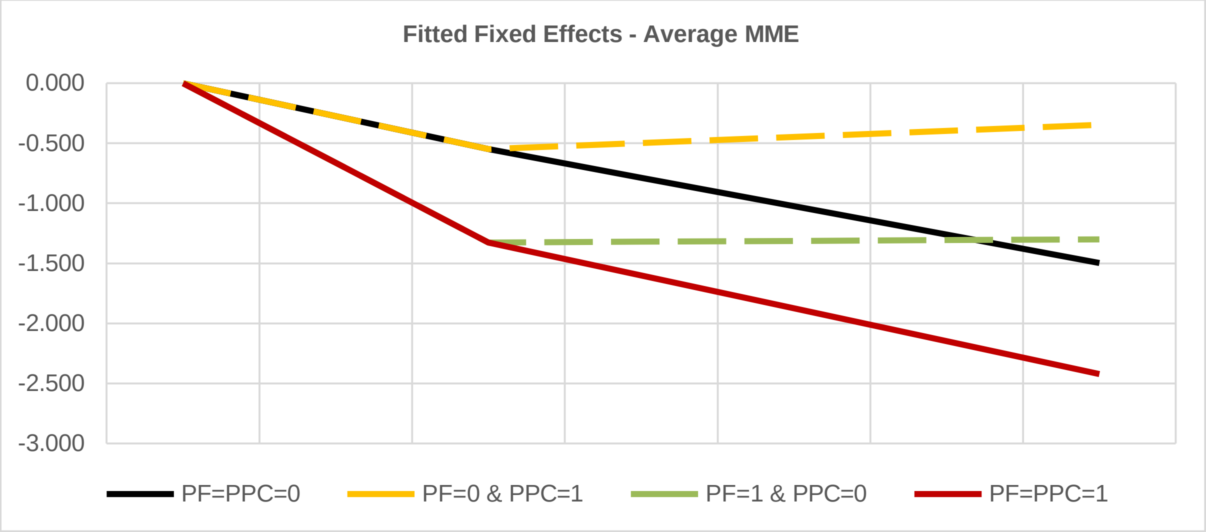

© 2024 Quanbeck A et al. *JAMA Network Open*.

The table below provides additional model output related to the previous graph of fitted changes to outcomes (average MME in this case). The top portion of second through fifth columns show the numerical values of the estimated changes in the previous graph. The next portion below shoes the estimated standard deviation related to each of the estimated outcome changes in the top portion. The final portion at the bottom shows the corresponding t-ratios associated with testing the null hypothesis that the true change from baseline is zero. So, for example, the PF & PPC arm shows an estimated change from t=0 to t=18 in average MME of -2.422 mg/day. This value has an estimated standard deviation of 1.404 mg/day. This implies the estimated values is -1.73 standard deviations from zero (the t-ratio). Roughly speaking, t-ratios greater than 2.00 in absolute value might be considered statistically significant and providing support to reject the null hypothesis of a zero true change in the outcome.

The three columns on the right side of the table show the differences between the estimated changes for each of the three intervention arms and the no-intervention arm. The appropriate standard deviations and t-ratios for these differences in changes are included. So, for example, the PF & PPC arm has an estimated 18-month decrease in average MME from baseline that is 0.925 mg/day greater than the change for the no-intervention arm. The corresponding standard deviation is 1.863 mg/day and the resulting t-ratio is only 0.5 standard deviations from zero. So, while there is some evidence that the PF & PPC arm experienced a greater drop in average MME than the no-intervention arm, that difference is not statistically significant.

Average MME

Study Month	Estimated Fixed Effect Curves				Differences		
	PF=PPC=0	PF=0 & PPC=1	PF=1 & PPC=0	PF=PPC=1	PF=0 & PPC=1 minus PF=PPC=0	PF=1 & PPC=0 minus PF=PPC=0	PF=PPC=1 minus PF=PPC=0
0	0.000	0.000	0.000	0.000	0.000	0.000	0.000
3	-0.275	-0.275	-0.663	-0.663	0.000	-0.388	-0.388
6	-0.550	-0.550	-1.326	-1.326	0.000	-0.776	-0.776
9	-0.787	-0.499	-1.320	-1.600	0.288	-0.533	-0.813
12	-1.024	-0.448	-1.313	-1.874	0.576	-0.290	-0.851
15	-1.261	-0.397	-1.307	-2.148	0.863	-0.047	-0.888
18	-1.498	-0.346	-1.301	-2.422	1.151	0.197	-0.925
**Std Dev of Estimated Values**							
0	0.000	0.000	0.000	0.000	0.000	0.000	0.000
3	0.393	0.393	0.423	0.423	0.000	0.561	0.561
6	0.785	0.785	0.845	0.845	0.000	1.122	1.122
9	0.802	0.795	0.855	0.849	0.400	1.134	1.130
12	0.907	0.907	0.961	0.965	0.800	1.282	1.285
15	1.074	1.090	1.138	1.161	1.200	1.526	1.543
18	1.280	1.315	1.357	1.404	1.600	1.829	1.863
**t-ratios of Estimated Values**							
0							

© 2024 Quanbeck A et al. *JAMA Network Open*.

3	-0.70	-0.70	-1.57	-1.57		-0.69	-0.69
6	-0.70	-0.70	-1.57	-1.57		-0.69	-0.69
9	-0.98	-0.63	-1.54	-1.89	0.72	-0.47	-0.72
12	-1.13	-0.49	-1.37	-1.94	0.72	-0.23	-0.66
15	-1.17	-0.36	-1.15	-1.85	0.72	-0.03	-0.58
18	-1.17	-0.26	-0.96	-1.73	0.72	0.11	-0.50

The tables below are taken directly from the SAS PROC Mixed output.

The first table below summarizes the estimated model variances/covariances associated with the random effects and the model error term. The values on the right side show the corresponding standard deviations and correlation coefficients. In this case, for example, the clinic-level intercept random effect has a standard deviation of 1.63 mg/day, while the PCP-level intercept random effect has a standard deviation of 19.47 mg/day. That is, much of the observed variation arises at the PCP level. The PCP-level slope random effect standard deviations are 0.66 mg/day and 0.48 mg/day (both per month of intervention duration) for the first 6 months and the last 12 months of the intervention period, respectively. Note also that the PCP-level random effects are correlated. For example, the two slope effects are 85% negatively correlated, meaning that when the first slope effect is positive(negative), the second tends to be negative(positive).

The model error term has an estimated standard deviation of 22.92 mg/day. Recall, however, that this value must be adjusted by the number of opioid patients in the denominator of the observed outcome measure. So, if a PCP has 10 opioid patients, this residual standard deviation will be $22.92/10^{1/2}$, or 7.25 mg/day.

Covariance Parameter Estimates

Cov Parm	Subject	Estimate		Std Dev & Correlation		
**Intercept**	Clinic	2.7	Clinic	1.63		
**UN(1,1)**	PCP	379.2				
**UN(2,1)**	PCP	(3.71)				
**UN(2,2)**	PCP	0.432	PCP	19.47		
**UN(3,1)**	PCP	1.78		-29.0%	0.66	
**UN(3,2)**	PCP	(0.27)		19.0%	-85.3%	0.48
**UN(3,3)**	PCP	0.232				
**Residual**		525.1	Residual	22.92		

The next table shows the estimated fixed effect coefficients. Here we can see the estimated health system intercept effects, i.e., 29.769 mg/day for Bellin and 46.526 mg/day for UW Health. We also see the estimated coefficient applied to the standardized clinic size. This clinic size effect is found to be insignificant for all study outcomes.

© 2024 Quanbeck A et al. *JAMA Network Open*.

Solution for Fixed Effects						
**Effect**	**system**	**Estimate**	**Standard Error**	**DF**	**t Value**	**Pr > |t|**
**system**	Bellin	29.769	2.251	802	13.23	<.0001
**system**	UW Health	46.526	1.754	802	26.53	<.0001
**clinic_size**		0.000	0.000	802	0.10	0.919
**t7**		-0.092	0.131	256	-0.70	0.485
**PF_t7**		-0.129	0.187	802	-0.69	0.489
**t13**		0.013	0.169	204	0.07	0.940
**PF_t13**		0.211	0.247	802	0.85	0.393
**PPC_t13**		0.096	0.133	802	0.72	0.472
**PFandPPC_t13**		-0.189	0.196	802	-0.97	0.333

© 2024 Quanbeck A et al. *JAMA Network Open*.

The next table shows estimates for various 18-month changes in outcomes by study arm and differences among these changes. The p-value column indicates which of these changes or differences in changes are significant given the volume of data underlying the estimates. Note that the estimates, standard errors and t-values correspond to the 18-month values presented in the table above.

Estimates					
Label	Estimate	Standard Error	DF	t Value	Pr > |t|
NOINT Chg	-1.4976	1.2805	802	-1.17	0.2425
PF Only Chg	-1.3013	1.3575	802	-0.96	0.3380
PPC Only Chg	-0.3463	1.3150	802	-0.26	0.7924
PF & PPC Chg	-2.4232	1.4036	802	-1.73	0.0847
PF Only vs NOINT	0.1963	1.8287	802	0.11	0.9145
PPC Only vs NOINT	1.1513	1.6002	802	0.72	0.4721
PF Only vs PPC Only 1	-0.9550	1.8534	802	-0.52	0.6065
PF Only vs PPC Only 2	-0.1785	1.6768	802	-0.11	0.9153
PF & PPC Interaction	-2.2732	2.3477	802	-0.97	0.3332
PF & PPC vs NOINT	-0.9255	1.8634	802	-0.50	0.6195

© 2024 Quanbeck A et al. *JAMA Network Open*.

The next two sets of plots display the behavior of the fitted model residuals. The first set focuses on marginal residuals, i.e., estimates of u(ij) + v_0(ijk) + v_1(ijk) t_7 + v_2(ijk) t_{13} + ε(ijkt), the difference between the observed outcome and the fixed effect estimate. These residuals include estimated random effects together with the model error term. Since these random components have differing variances and are blended with varying weights, we don't expect these to necessarily have stable variances or to be distributed normally. Indeed, we see some heavy-tailed behavior in these plots.

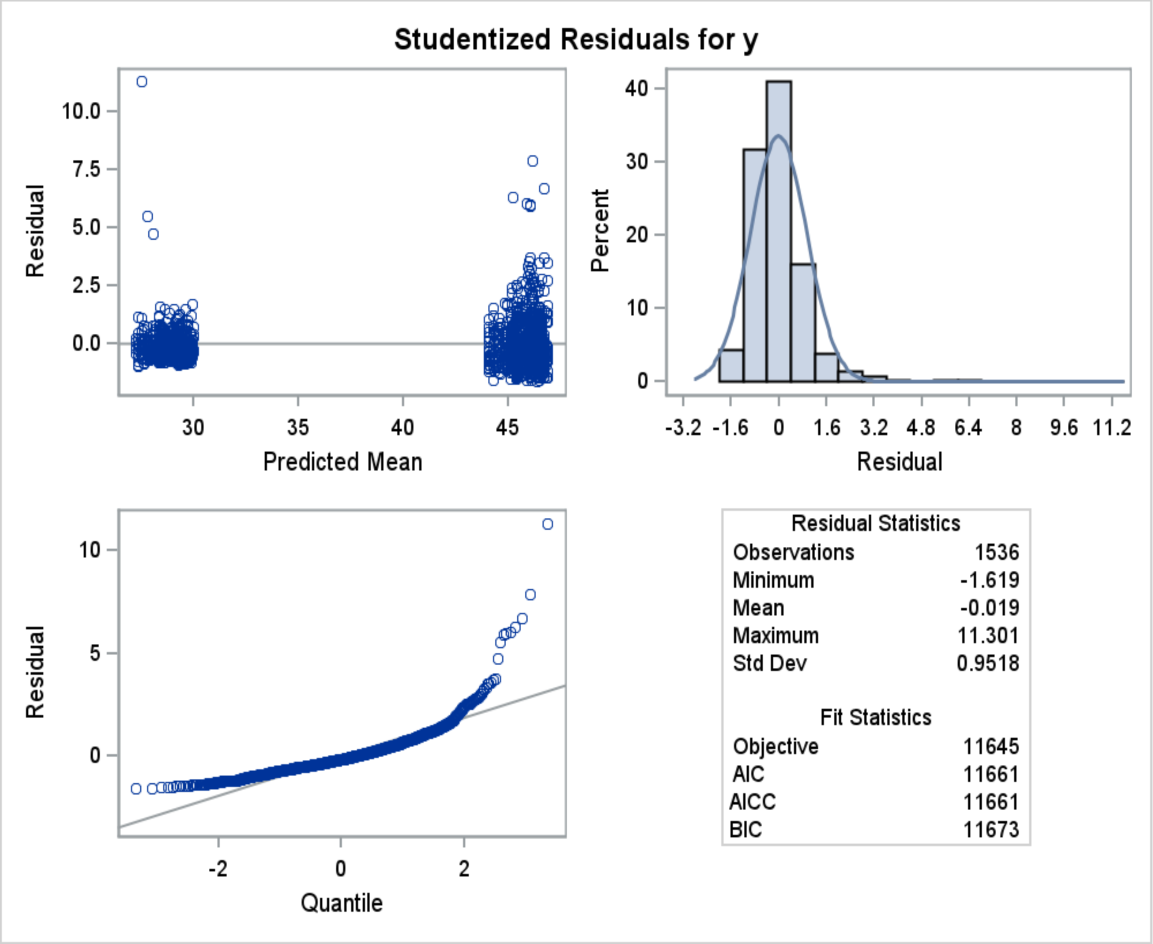

© 2024 Quanbeck A et al. *JAMA Network Open*.

The second set of plots focuses on conditional residuals, i.e., estimates of the model error term ε(ijkt) alone. These values are standardized, so provision has been made for the assumption that the error variance is inversely proportional to the number of opioid patients in the measure denominator. While the distribution appears more symmetric that the marginal residual plots, the tails appear heavier than would be expected with a Normal distribution. This indicates that the estimated model p-values may be under-stated (i.e., the significance of estimated effects may over-estimated). The increasing residual variance with increasing predicted values shown in the first plot below also suggests that a variance stabilizing transformation (such as a log) of the outcome variable might be appropriate (if we continued with addition model building steps).

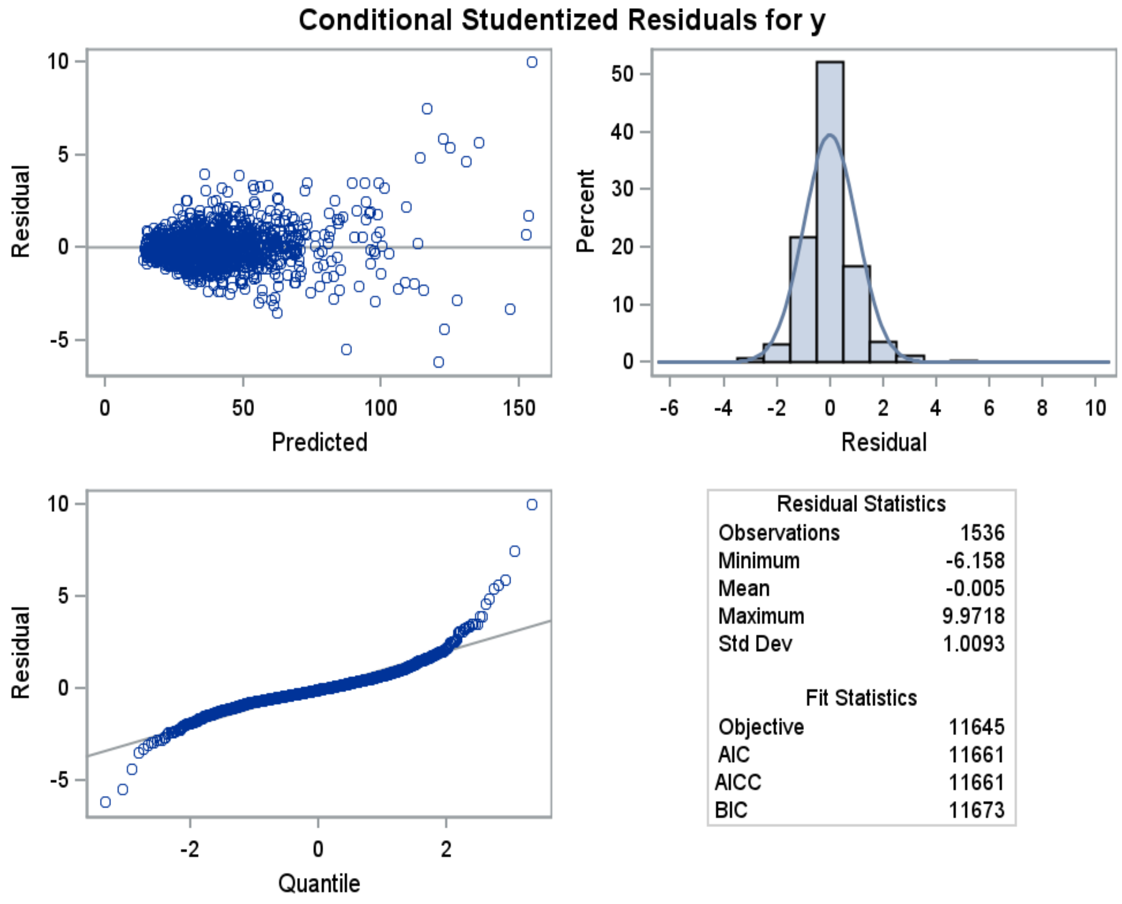

Residual Statistics	
Observations	1536
Minimum	-6.158
Mean	-0.005
Maximum	9.9718
Std Dev	1.0093

Fit Statistics	
Objective	11645
AIC	11661
AICC	11661
BIC	11673

© 2024 Quanbeck A et al. *JAMA Network Open*.

Appendix A2 – Percent of Opioid Patients with Average MME ≥ 90 mg/day

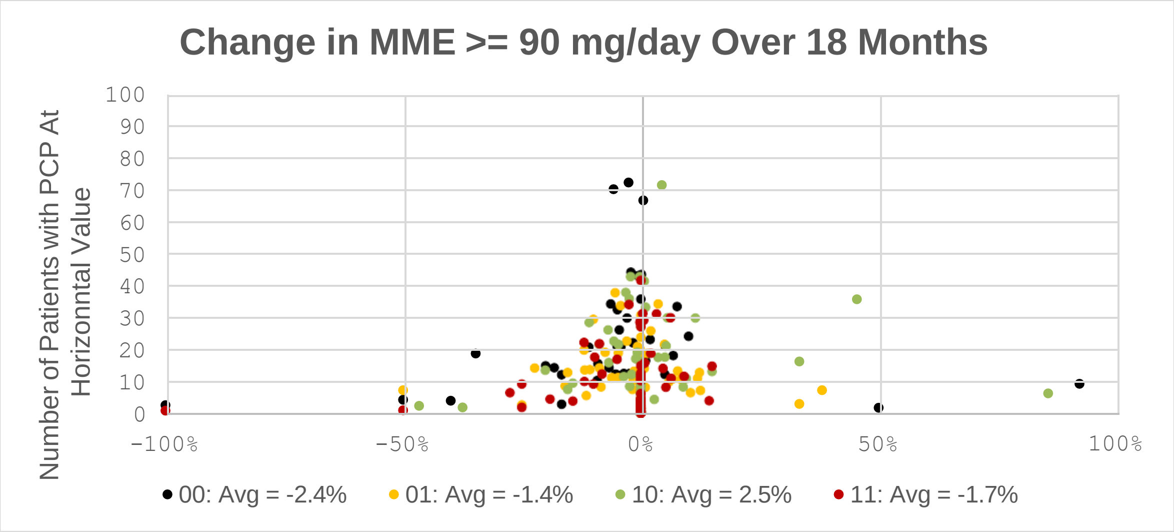

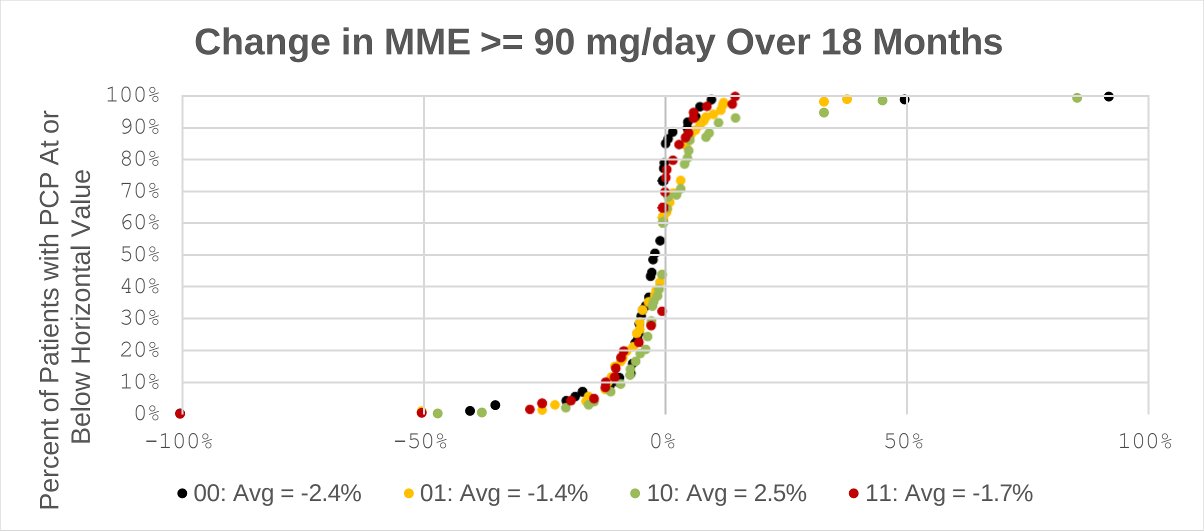

© 2024 Quanbeck A et al. *JAMA Network Open*.

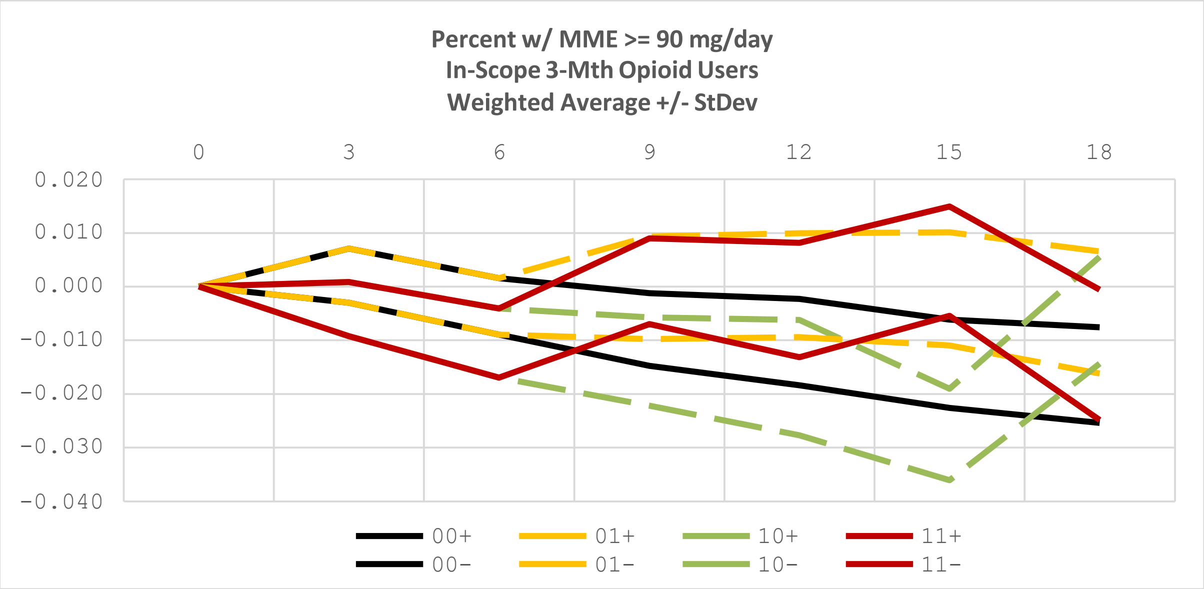

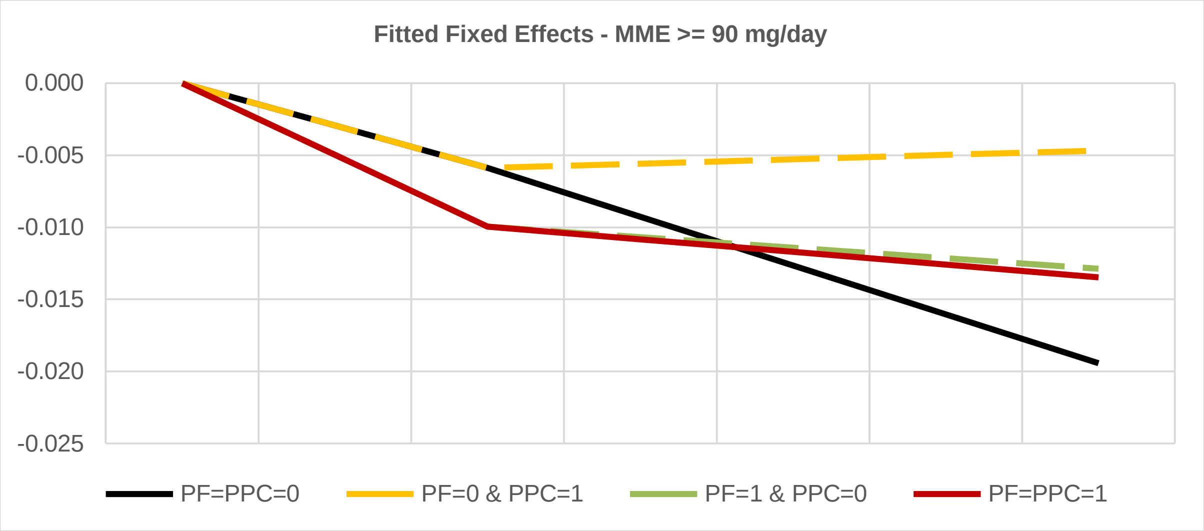

MME >= 90 mg/day		
	**Estimated Fixed Effect Curves**	**Differences**

© 2024 Quanbeck A et al. *JAMA Network Open*.

Study Month	PF=PPC=0	PF=0 & PPC=1	PF=1 & PPC=0	PF=PPC=1	PF=0 & PPC=1 minus PF=PPC=0	PF=1 & PPC=0 minus PF=PPC=0	PF=PPC=1 minus PF=PPC=0
0	0.000	0.000	0.000	0.000	0.000	0.000	0.000
3	-0.003	-0.003	-0.005	-0.005	0.000	-0.002	-0.002
6	-0.006	-0.006	-0.010	-0.010	0.000	-0.004	-0.004
9	-0.009	-0.006	-0.011	-0.011	0.004	-0.001	-0.002
12	-0.013	-0.005	-0.011	-0.012	0.007	0.001	0.001
15	-0.016	-0.005	-0.012	-0.013	0.011	0.004	0.003
18	-0.019	-0.005	-0.013	-0.013	0.015	0.007	0.006
**Std Dev of Estimated Values**							
0	0.000	0.000	0.000	0.000	0.000	0.000	0.000
3	0.002	0.002	0.003	0.003	0.000	0.003	0.003
6	0.005	0.005	0.005	0.005	0.000	0.007	0.007
9	0.005	0.005	0.005	0.005	0.003	0.007	0.007
12	0.006	0.006	0.006	0.006	0.006	0.008	0.008
15	0.007	0.007	0.007	0.007	0.009	0.010	0.010
18	0.009	0.009	0.009	0.009	0.012	0.012	0.012
**t-ratios of Estimated Values**							
0							
3	-1.25	-1.25	-1.95	-1.95		-0.60	-0.60
6	-1.25	-1.25	-1.95	-1.95		-0.60	-0.60
9	-1.96	-1.18	-2.10	-2.14	1.28	-0.21	-0.23
12	-2.26	-0.94	-1.93	-1.96	1.28	0.16	0.12
15	-2.30	-0.70	-1.67	-1.68	1.28	0.40	0.35
18	-2.25	-0.53	-1.43	-1.45	1.28	0.54	0.48

© 2024 Quanbeck A et al. *JAMA Network Open*.

Covariance Parameter Estimates

Cov Parm	Subject	Estimate		Std Dev & Correlation		
Intercept	Clinic	0.0002	Clinic	0.0123		
UN(1,1)	PCP	0.0091				
UN(2,1)	PCP	0.0000				
UN(2,2)	PCP	0.0000	PCP	0.0953		
UN(3,1)	PCP	(0.0002)		16.8%	0.0023	
UN(3,2)	PCP	(0.0000)		-73.2%	-18.4%	0.0029
UN(3,3)	PCP	0.0000				
Residual		0.0234	Residual	0.1530		

Solution for Fixed Effects

Effect	system	Estimate	Standard Error	DF	t Value	Pr > |t|
system	Bellin	0.0369	0.0118	802	3.14	0.0018
system	UW Health	0.1269	0.0094	802	13.49	<.0001
clinic_size		0.0000	0.0000	802	-Infty	<.0001
t7		-0.0010	0.0008	256	-1.26	0.211
PF_t7		-0.0007	0.0011	802	-0.59	0.553
t13		-0.0002	0.0011	204	-0.13	0.897
PF_t13		0.0016	0.0017	802	0.93	0.350
PPC_t13		0.0012	0.0010	802	1.28	0.200
PFandPPC_t13		-0.0013	0.0014	802	-0.92	0.358

Estimates

Label	Estimate	Standard Error	DF	t Value	Pr > |t|
NOINT Chg	-0.0194	0.0086	802	-2.25	0.025
PF Only Chg	-0.0128	0.0091	802	-1.40	0.161
PPC Only Chg	-0.0047	0.0089	802	-0.53	0.599
PF & PPC Chg	-0.0135	0.0094	802	-1.43	0.154
PF Only vs NOINT	0.0066	0.0123	802	0.54	0.590

© 2024 Quanbeck A et al. *JAMA Network Open*.

**PPC Only vs NOINT**	0.0148	0.0115	802	1.28	0.200
**PF Only vs PPC Only 1**	-0.0082	0.0124	802	-0.66	0.512
**PF Only vs PPC Only 2**	-0.0041	0.0122	802	-0.34	0.737
**PF & PPC Interaction**	-0.0154	0.0168	802	-0.92	0.358
**PF & PPC vs NOINT**	0.0060	0.0125	802	0.48	0.634

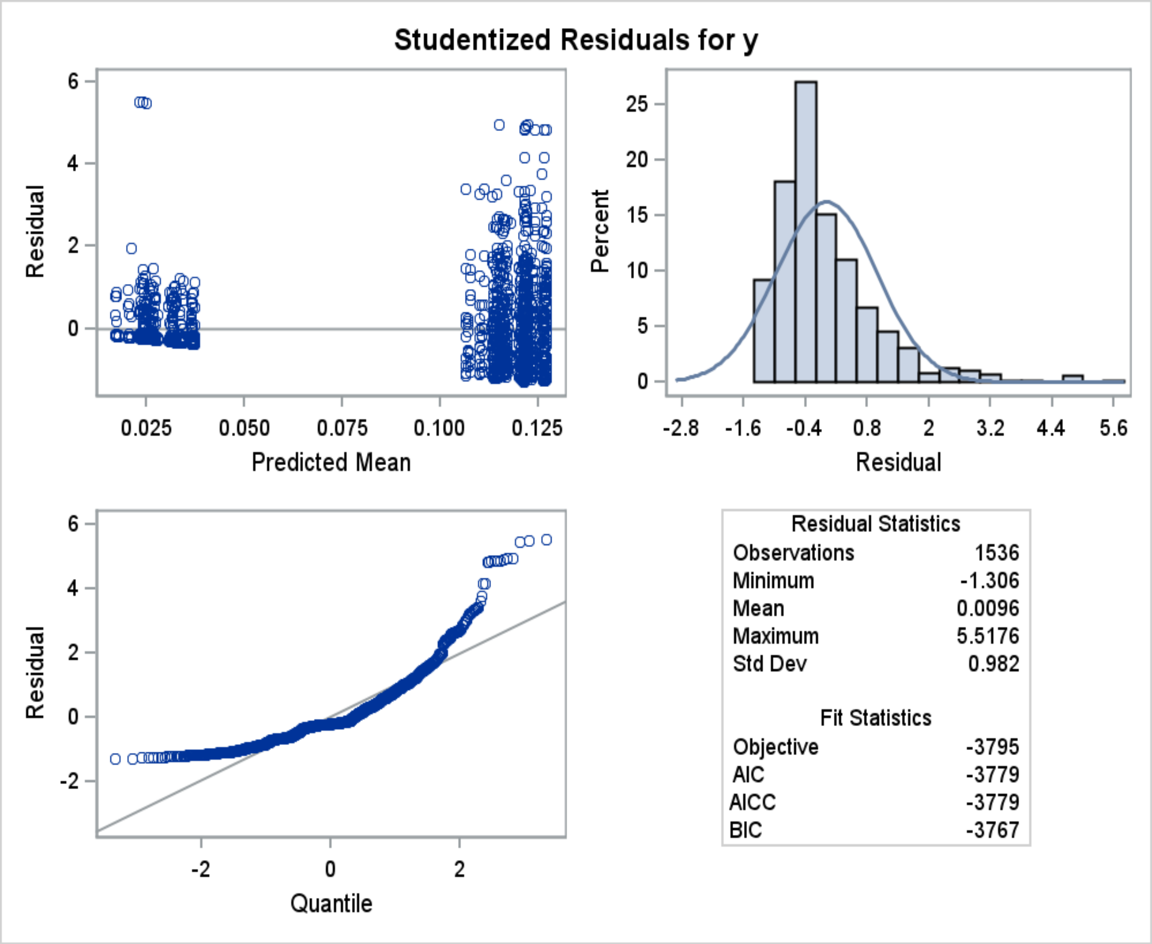

© 2024 Quanbeck A et al. *JAMA Network Open*.

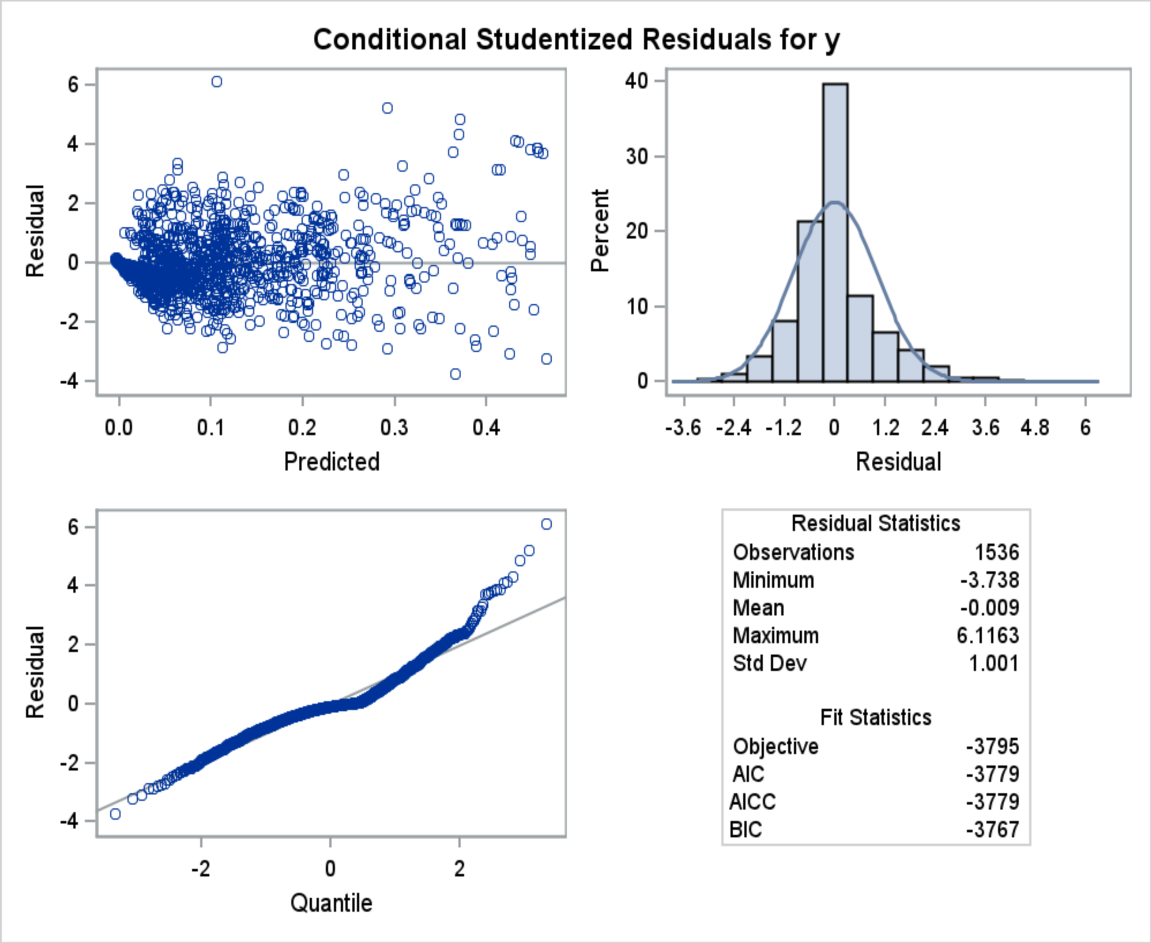

© 2024 Quanbeck A et al. *JAMA Network Open*.

Appendix A3 – Percent of Opioid Patients with Benzo's in Last 3 Months

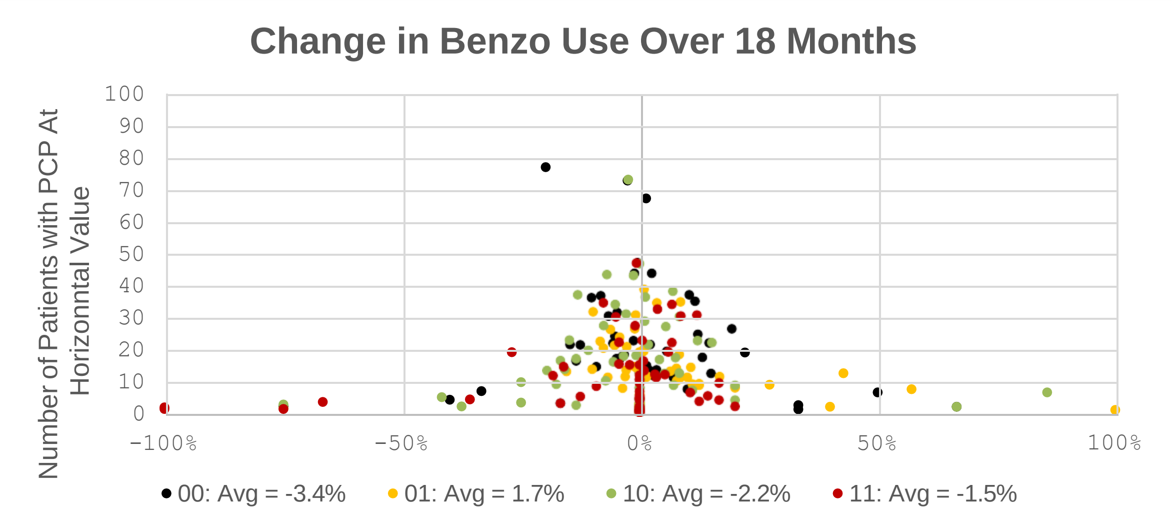

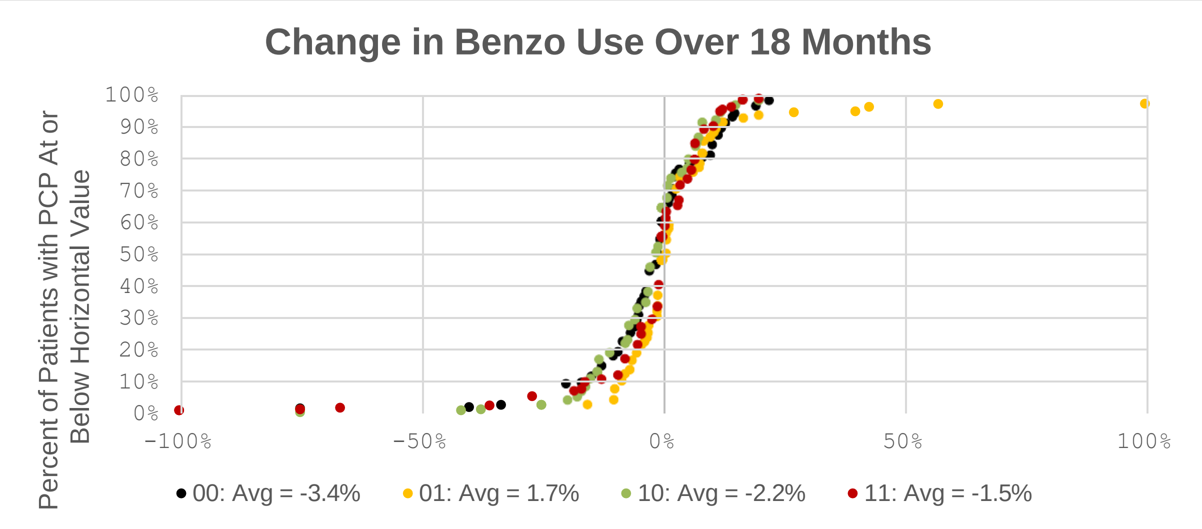

© 2024 Quanbeck A et al. *JAMA Network Open*.

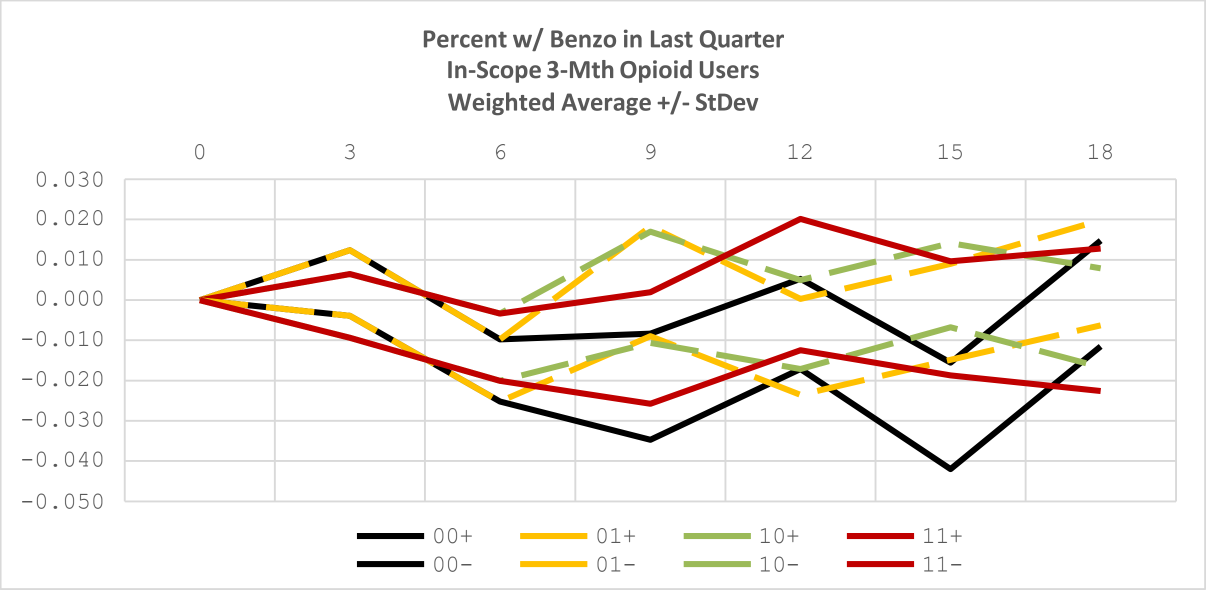

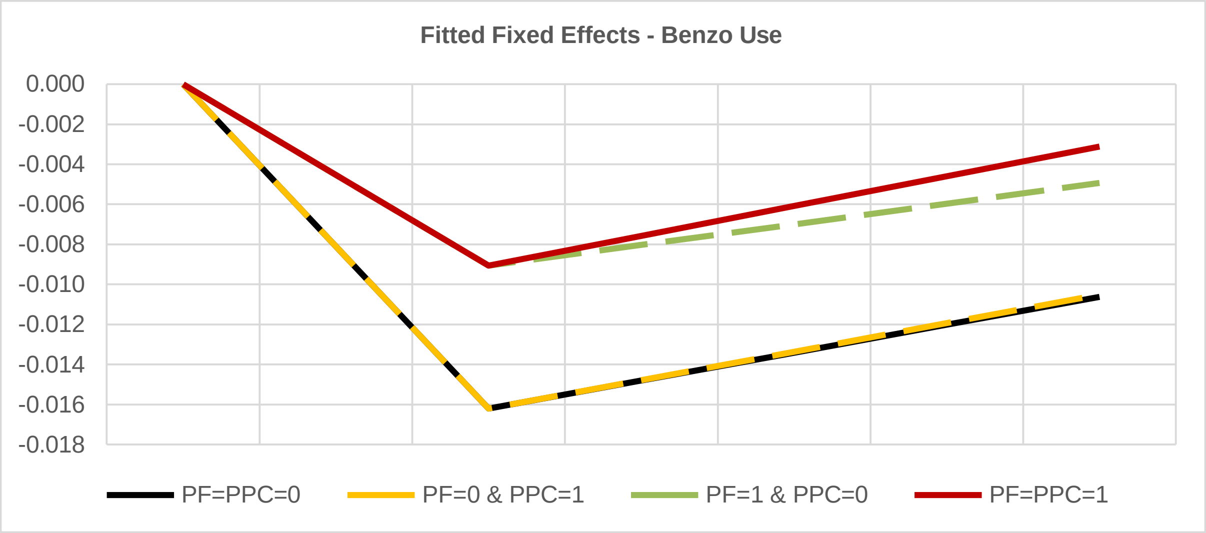

Benzo Use		
	Estimated Fixed Effect Curves	Differences

© 2024 Quanbeck A et al. *JAMA Network Open*.

Study Month	PF=PPC=0	PF=0 & PPC=1	PF=1 & PPC=0	PF=PPC=1	PF=0 & PPC=1 minus PF=PPC=0	PF=1 & PPC=0 minus PF=PPC=0	PF=PPC=1 minus PF=PPC=0
0	0.000	0.000	0.000	0.000	0.000	0.000	0.000
3	-0.008	-0.008	-0.005	-0.005	0.000	0.004	0.004
6	-0.016	-0.016	-0.009	-0.009	0.000	0.007	0.007
9	-0.015	-0.015	-0.008	-0.008	0.000	0.007	0.007
12	-0.013	-0.013	-0.007	-0.006	0.000	0.006	0.007
15	-0.012	-0.012	-0.006	-0.005	0.000	0.006	0.007
18	-0.011	-0.011	-0.005	-0.003	0.000	0.006	0.008
**Std Dev of Estimated Values**							
0	0.000	0.000	0.000	0.000	0.000	0.000	0.000
3	0.004	0.004	0.004	0.004	0.000	0.005	0.005
6	0.007	0.007	0.008	0.008	0.000	0.010	0.010
9	0.007	0.007	0.007	0.007	0.004	0.009	0.009
12	0.007	0.007	0.008	0.008	0.007	0.010	0.010
15	0.008	0.009	0.009	0.010	0.011	0.012	0.012
18	0.010	0.011	0.011	0.012	0.014	0.015	0.015
**t-ratios of Estimated Values**							
0							
3	-2.29	-2.29	-1.18	-1.18		0.72	0.72
6	-2.29	-2.29	-1.18	-1.18		0.72	0.72
9	-2.23	-2.22	-1.12	-1.05	0.01	0.74	0.78
12	-1.88	-1.82	-0.91	-0.77	0.01	0.64	0.72
15	-1.42	-1.34	-0.66	-0.48	0.01	0.51	0.60
18	-1.04	-0.96	-0.45	-0.27	0.01	0.39	0.50

© 2024 Quanbeck A et al. *JAMA Network Open*.

Covariance Parameter Estimates						
Cov Parm	Subject	Estimate		Std Dev & Correlation		
Intercept	Clinic	0.0003	Clinic	0.0182		
UN(1,1)	PCP	0.0065				
UN(2,1)	PCP	(0.0001)				
UN(2,2)	PCP	0.0000	PCP	0.0806		
UN(3,1)	PCP	0.0001		-31.0%	0.0040	
UN(3,2)	PCP	(0.0000)		29.3%	-100.0%	0.0050
UN(3,3)	PCP	0.0000				
Residual		0.0569	Residual	0.2386		

Solution for Fixed Effects						
Effect	system	Estimate	Standard Error	DF	t Value	Pr > |t|
system	Bellin	0.1156	0.0119	805	9.69	<.0001
system	UW Health	0.1290	0.0101	805	12.84	<.0001
clinic_size		0.0000	0.0000	805	Infty	<.0001
t7		-0.0027	0.0012	256	-2.29	0.023
PF_t7		0.0012	0.0016	805	0.72	0.470
t13		0.0032	0.0017	204	1.88	0.061
PF_t13		-0.0013	0.0024	805	-0.54	0.588
PPC_t13		0.0000	0.0012	805	0.01	0.994
PFandPPC_t13		0.0001	0.0017	805	0.08	0.933

Estimates					
Label	Estimate	Standard Error	DF	t Value	Pr > |t|
NOINT Chg	-0.0107	0.0102	805	-1.05	0.294
PF Only Chg	-0.0050	0.0109	805	-0.45	0.651
PPC Only Chg	-0.0106	0.0108	805	-0.98	0.330
PF & PPC Chg	-0.0031	0.0116	805	-0.27	0.787
PF Only vs NOINT	0.0057	0.0147	805	0.39	0.696
PPC Only vs NOINT	0.0001	0.0140	805	0.01	0.994

© 2024 Quanbeck A et al. *JAMA Network Open*.

**PF Only vs PPC Only 1**	0.0056	0.0151	805	0.37	0.710
**PF Only vs PPC Only 2**	-0.0015	0.0156	805	-0.10	0.923
**PF & PPC Interaction**	0.0017	0.0205	805	0.08	0.933
**PF & PPC vs NOINT**	0.0075	0.0151	805	0.50	0.618

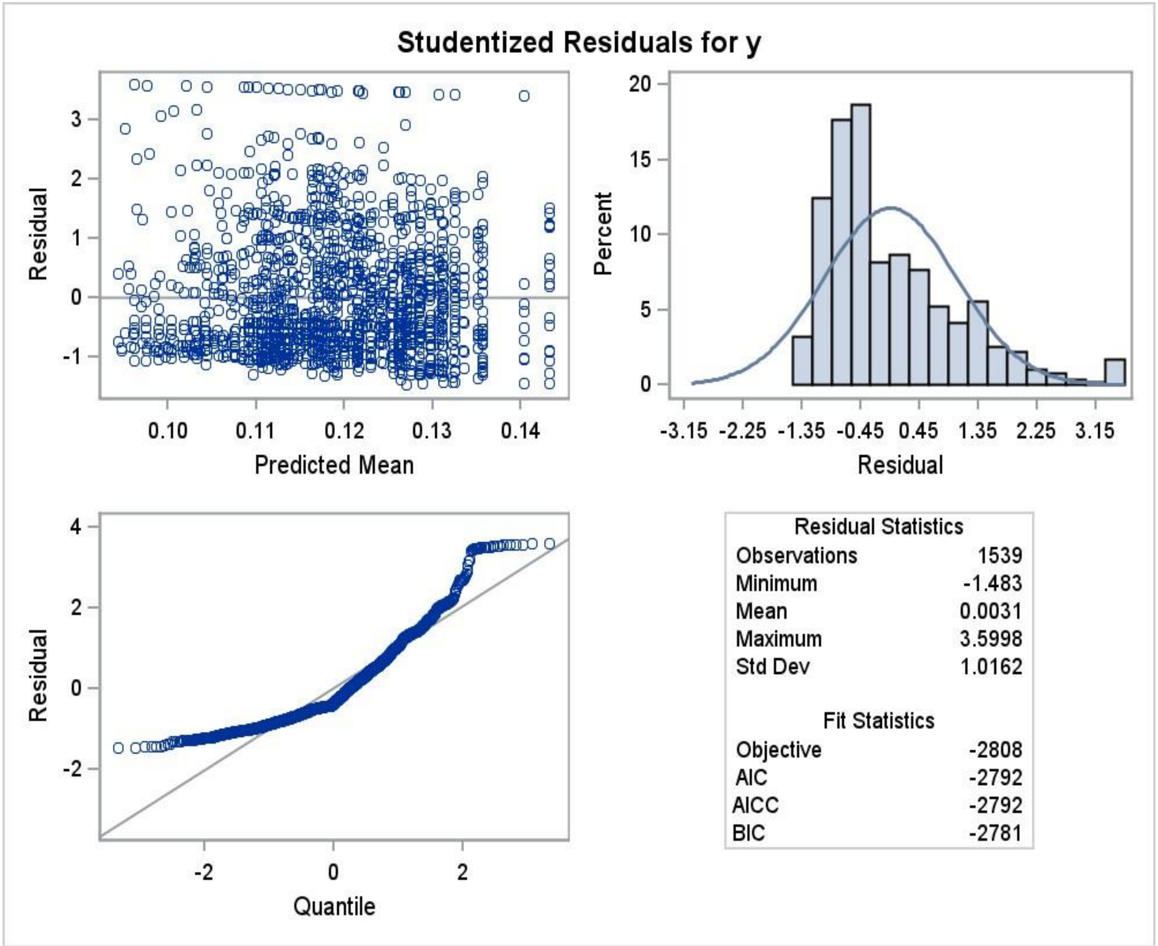

© 2024 Quanbeck A et al. *JAMA Network Open*.

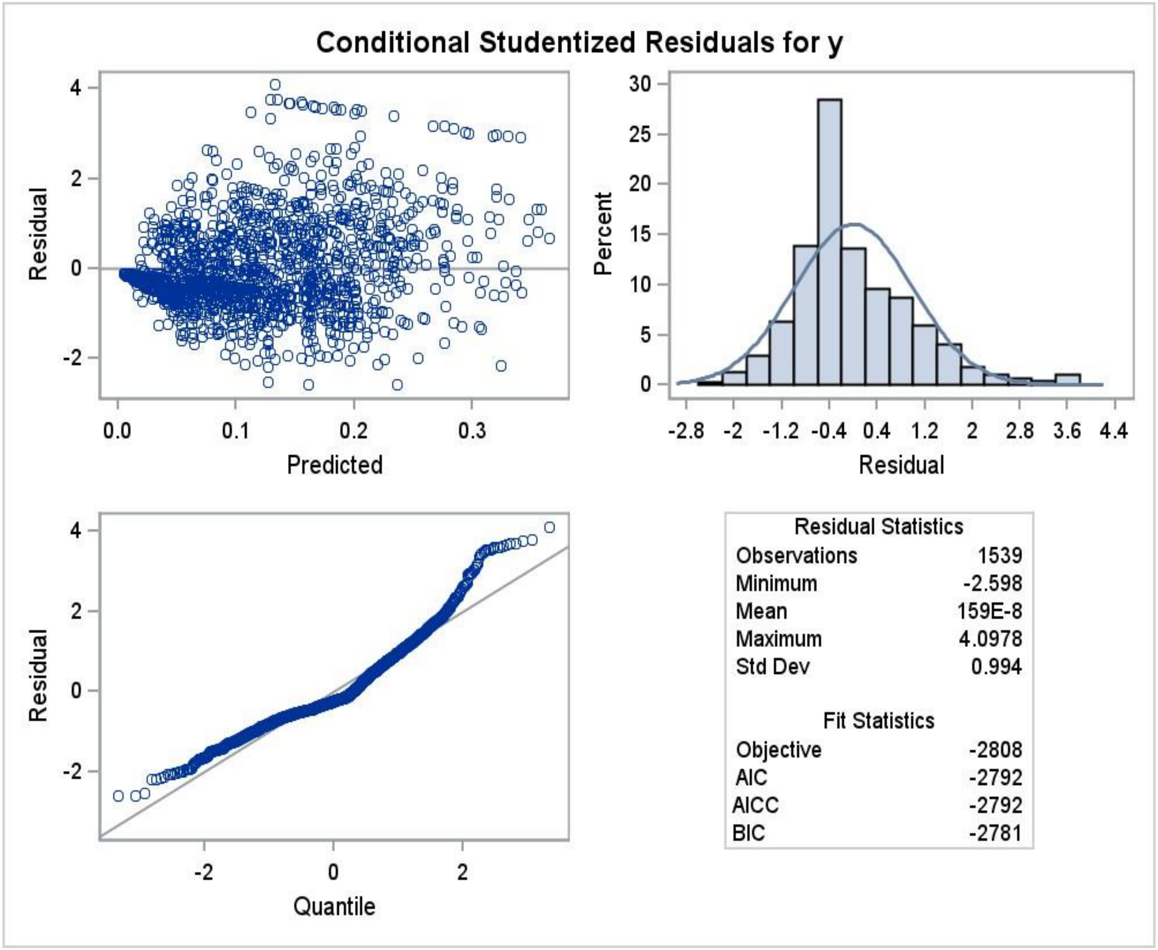

© 2024 Quanbeck A et al. *JAMA Network Open*.

Appendix A4 – Percent of Opioid Patients with Urine Drug Screen in Last 12 Months

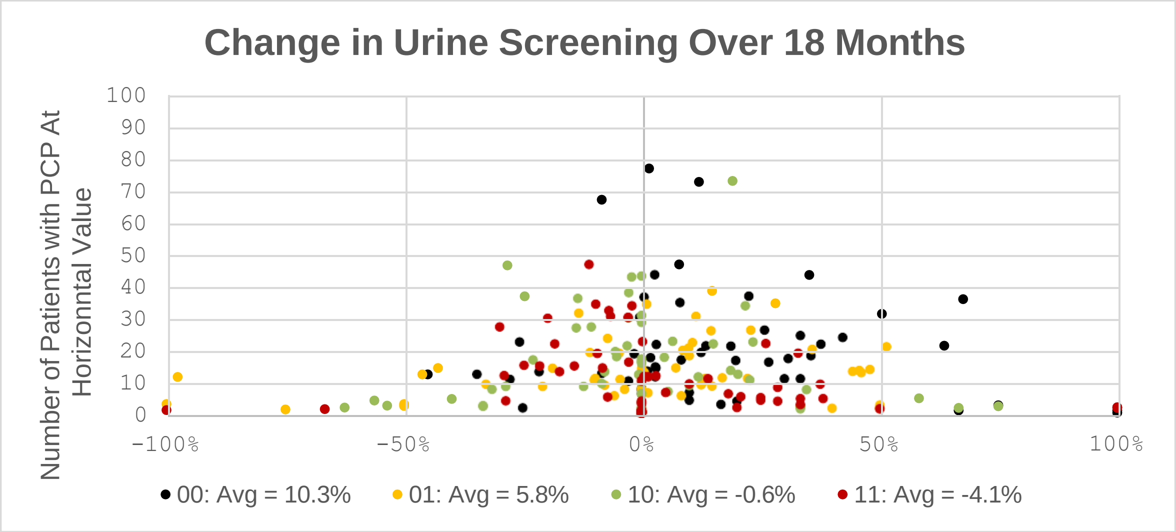

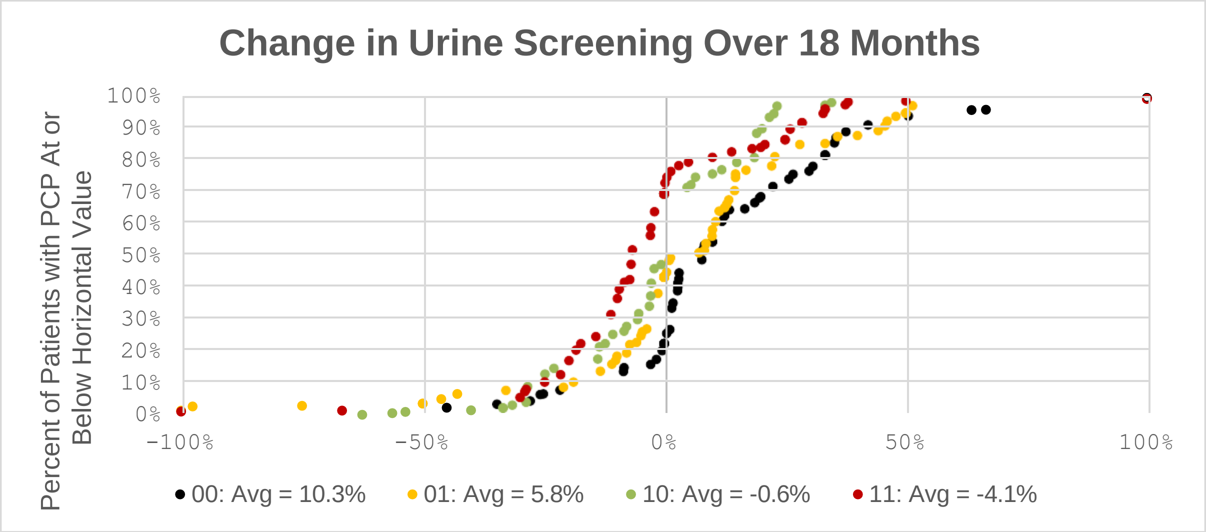

© 2024 Quanbeck A et al. *JAMA Network Open*.

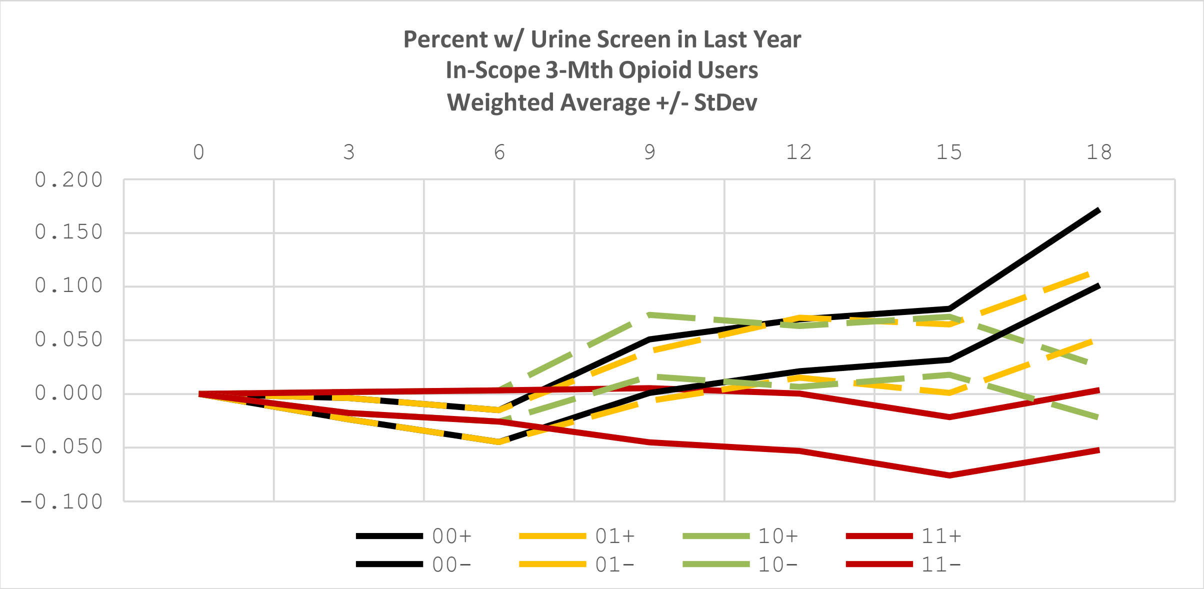

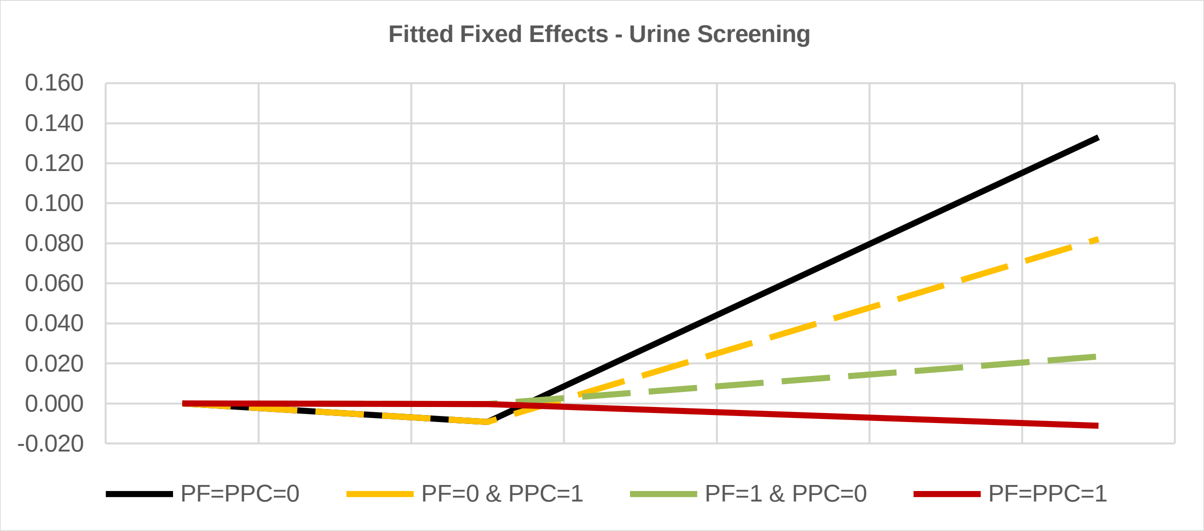

Urine Screening		
	Estimated Fixed Effect Curves	Differences

© 2024 Quanbeck A et al. *JAMA Network Open*.

Study Month	PF=PPC=0	PF=0 & PPC=1	PF=1 & PPC=0	PF=PPC=1	PF=0 & PPC=1 minus PF=PPC=0	PF=1 & PPC=0 minus PF=PPC=0	PF=PPC=1 minus PF=PPC=0
0	0.000	0.000	0.000	0.000	0.000	0.000	0.000
3	-0.005	-0.005	0.000	0.000	0.000	0.004	0.004
6	-0.009	-0.009	0.000	0.000	0.000	0.009	0.009
9	0.026	0.014	0.006	-0.003	-0.013	-0.021	-0.029
12	0.062	0.036	0.012	-0.006	-0.025	-0.050	-0.068
15	0.097	0.059	0.017	-0.008	-0.038	-0.080	-0.106
18	0.133	0.082	0.023	-0.011	-0.051	-0.110	-0.144
**Std Dev of Estimated Values**							
0	0.000	0.000	0.000	0.000	0.000	0.000	0.000
3	0.008	0.008	0.009	0.009	0.000	0.012	0.012
6	0.016	0.016	0.017	0.017	0.000	0.023	0.023
9	0.016	0.016	0.016	0.016	0.009	0.020	0.020
12	0.019	0.019	0.020	0.020	0.018	0.020	0.019
15	0.023	0.023	0.027	0.027	0.027	0.022	0.021
18	0.029	0.029	0.036	0.035	0.037	0.026	0.025
**t-ratios of Estimated Values**							
0							
3	-0.57	-0.57	-0.02	-0.02		0.38	0.38
6	-0.57	-0.57	-0.02	-0.02		0.38	0.38
9	1.61	0.84	0.35	-0.19	-1.39	-1.02	-1.45
12	3.25	1.93	0.57	-0.29	-1.39	-2.53	-3.47
15	4.15	2.54	0.64	-0.32	-1.39	-3.63	-4.98
18	4.61	2.86	0.66	-0.32	-1.39	-4.19	-5.75

© 2024 Quanbeck A et al. *JAMA Network Open*.

Covariance Parameter Estimates						
**Cov Parm**	**Subject**	**Estimate**		**Std Dev & Correlation**		
**Intercept**	Clinic	0.0157	Clinic	0.1253		
**UN(1,1)**	PCP	0.0320				
**UN(2,1)**	PCP	(0.0018)				
**UN(2,2)**	PCP	0.0004	PCP	0.1789		
**UN(3,1)**	PCP	0.0013		-47.8%	0.0208	
**UN(3,2)**	PCP	(0.0005)		28.2%	-86.0%	0.0257
**UN(3,3)**	PCP	0.0007				
**Residual**		0.1129	Residual	0.3360		

Solution for Fixed Effects						
**Effect**	**system**	**Estimate**	**Standard Error**	**DF**	**t Value**	**Pr > |t|**
**system**	Bellin	0.2485	0.0392	805	6.35	<.0001
**system**	UW Health	0.3536	0.0399	805	8.86	<.0001
**clinic_size**		0.0000	0.0000	805	-2.05	0.040
**t7**		-0.0015	0.0027	256	-0.57	0.571
**PF_t7**		0.0015	0.0039	805	0.38	0.703
**t13**		0.0134	0.0038	204	3.49	0.001
**PF_t13**		-0.0114	0.0055	805	-2.05	0.041
**PPC_t13**		-0.0042	0.0030	805	-1.39	0.165
**PFandPPC_t13**		0.0014	0.0044	805	0.31	0.757

Estimates					
**Label**	**Estimate**	**Standard Error**	**DF**	**t Value**	**Pr > |t|**
**NOINT Chg**	0.1330	0.0281	805	4.73	<.0001
**PF Only Chg**	0.0234	0.0292	805	0.80	0.423
**PPC Only Chg**	0.0821	0.0280	805	2.93	0.003
**PF & PPC Chg**	-0.0111	0.0293	805	-0.38	0.705
**PF Only vs NOINT**	-0.1095	0.0401	805	-2.73	0.006
**PPC Only vs NOINT**	-0.0509	0.0366	805	-1.39	0.165

© 2024 Quanbeck A et al. *JAMA Network Open*.

**PF Only vs PPC Only 1**	-0.0586	0.0400	805	-1.47	0.143
**PF Only vs PPC Only 2**	-0.0675	0.0384	805	-1.76	0.079
**PF & PPC Interaction**	0.0164	0.0529	805	0.31	0.757
**PF & PPC vs NOINT**	-0.1440	0.0402	805	-3.59	0.000

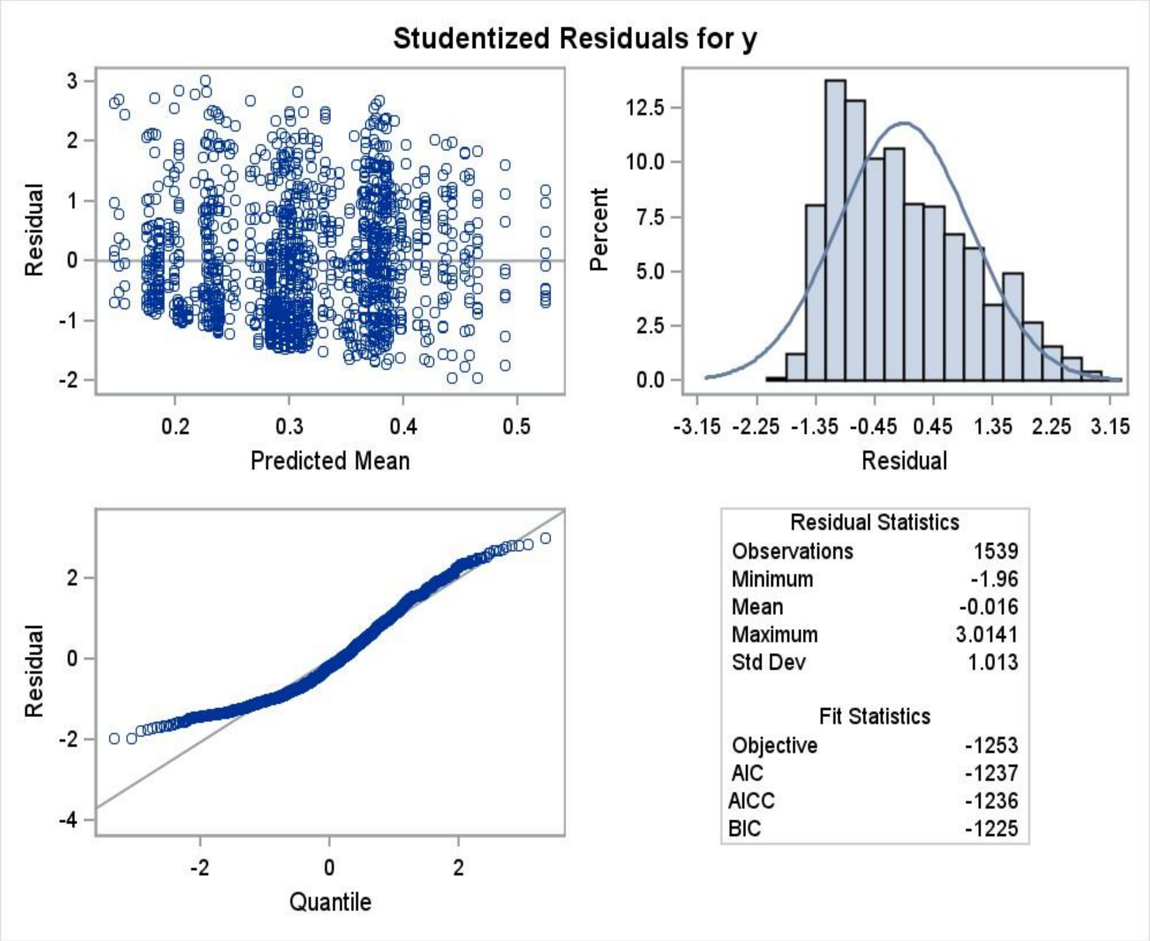

© 2024 Quanbeck A et al. *JAMA Network Open*.

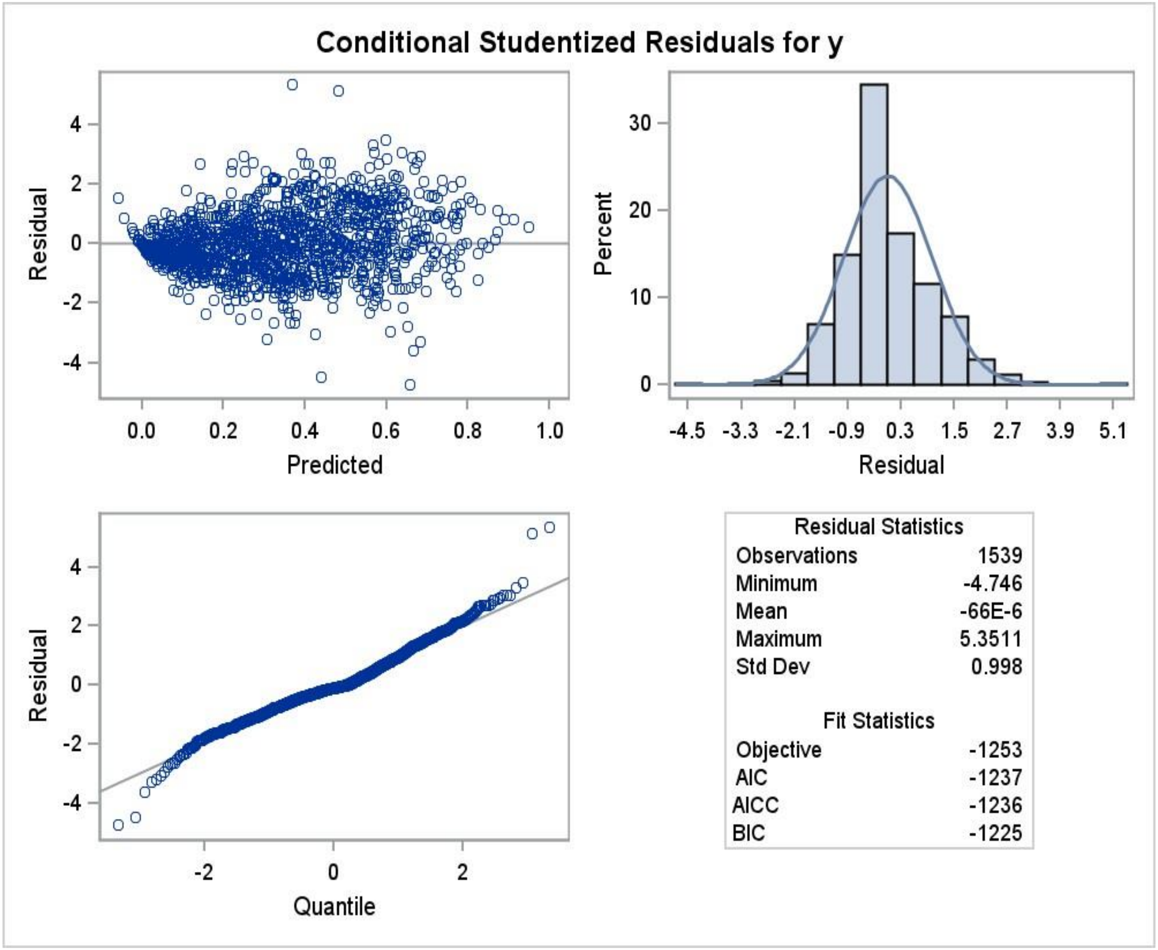

© 2024 Quanbeck A et al. *JAMA Network Open*.

Appendix A5 – Percent of Opioid Patients with Treatment Agreement in Last 12 Months

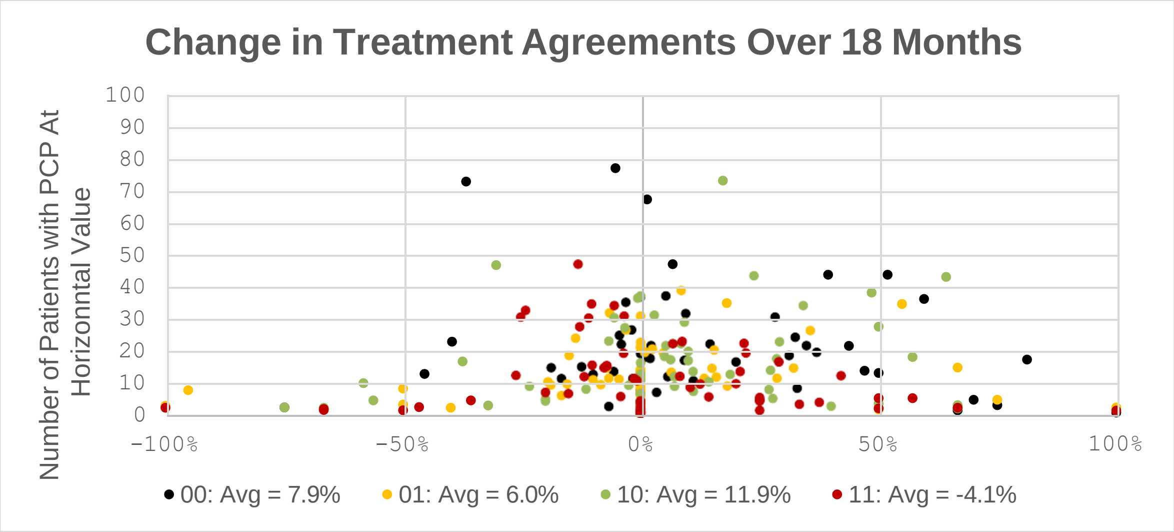

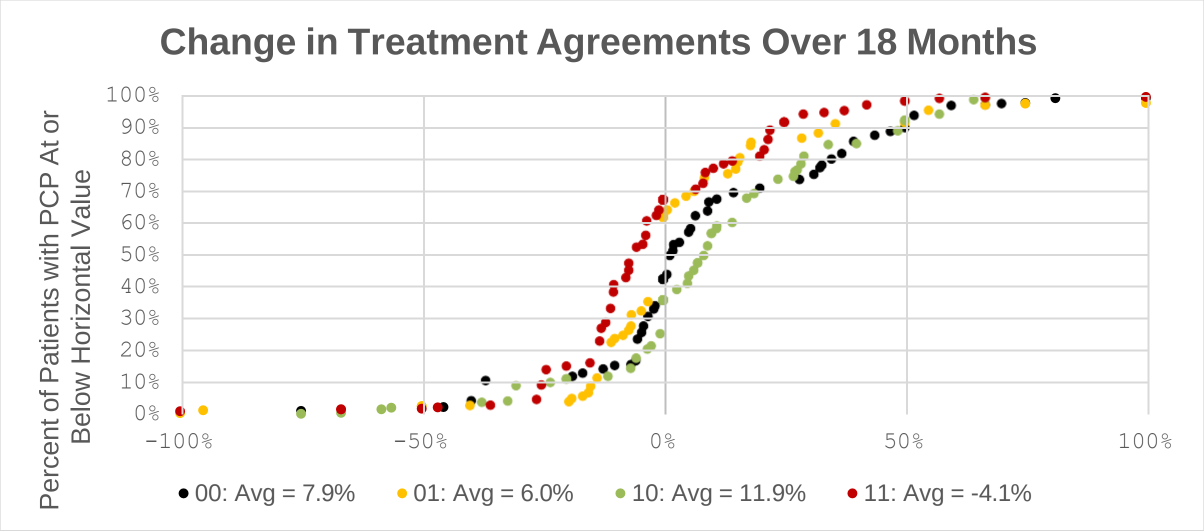

© 2024 Quanbeck A et al. *JAMA Network Open*.

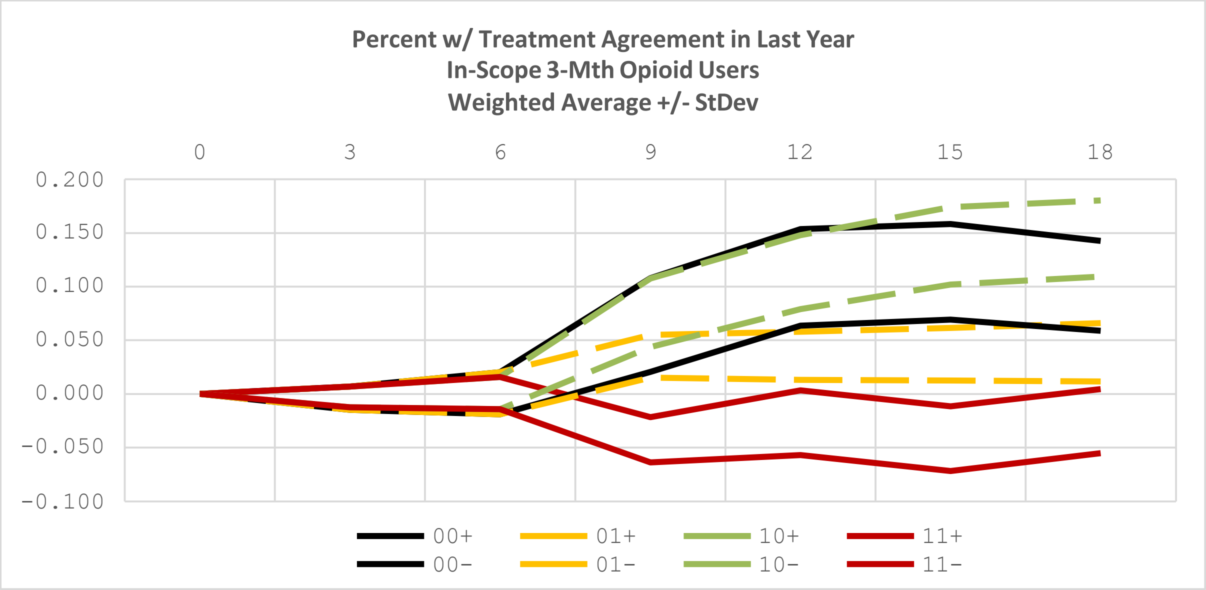

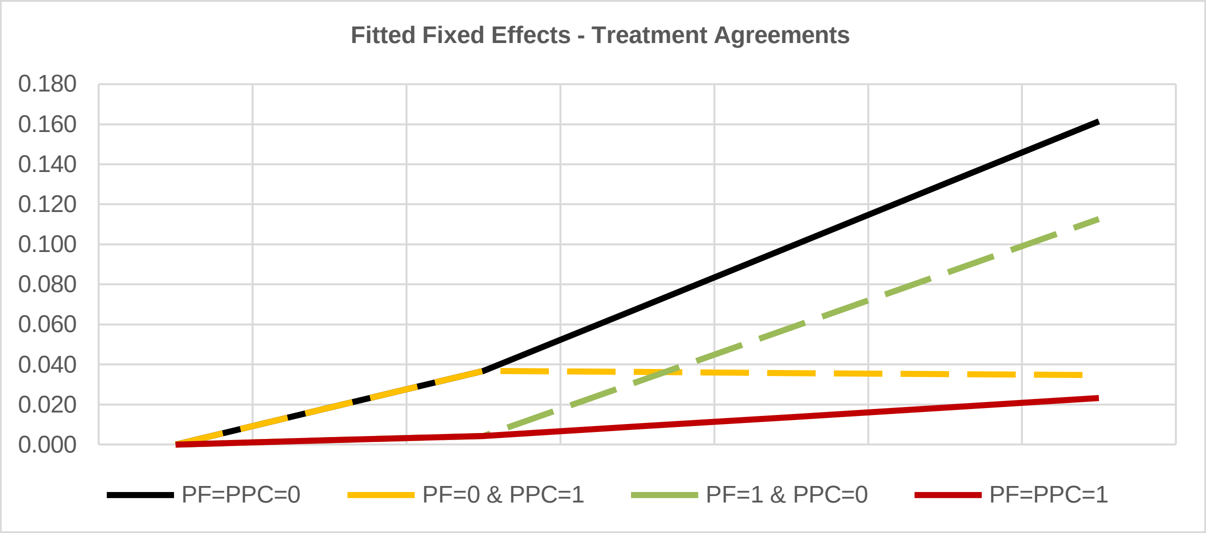

**Treatment Agreements**		
	**Estimated Fixed Effect Curves**	**Differences**

© 2024 Quanbeck A et al. *JAMA Network Open*.

Study Month	PF=PPC=0	PF=0 & PPC=1	PF=1 & PPC=0	PF=PPC=1	PF=0 & PPC=1 minus PF=PPC=0	PF=1 & PPC=0 minus PF=PPC=0	PF=PPC=1 minus PF=PPC=0
0	0.000	0.000	0.000	0.000	0.000	0.000	0.000
3	0.018	0.018	0.002	0.002	0.000	-0.016	-0.016
6	0.037	0.037	0.004	0.004	0.000	-0.033	-0.033
9	0.068	0.036	0.031	0.009	-0.032	-0.037	-0.059
12	0.099	0.036	0.058	0.014	-0.063	-0.041	-0.085
15	0.130	0.035	0.086	0.018	-0.095	-0.045	-0.112
18	0.161	0.035	0.113	0.023	-0.127	-0.049	-0.138
**Std Dev of Estimated Values**							
0	0.000	0.000	0.000	0.000	0.000	0.000	0.000
3	0.009	0.009	0.010	0.010	0.000	0.013	0.013
6	0.019	0.019	0.020	0.020	0.000	0.027	0.027
9	0.021	0.020	0.023	0.023	0.009	0.029	0.029
12	0.025	0.024	0.029	0.028	0.019	0.035	0.035
15	0.030	0.030	0.035	0.035	0.028	0.044	0.044
18	0.037	0.037	0.042	0.041	0.037	0.054	0.053
**t-ratios of Estimated Values**							
0							
3	1.94	1.94	0.21	0.21		-1.21	-1.21
6	1.94	1.94	0.21	0.21		-1.21	-1.21
9	3.30	1.78	1.34	0.39	-3.40	-1.25	-2.02
12	4.01	1.46	2.04	0.48	-3.40	-1.15	-2.42
15	4.28	1.17	2.45	0.53	-3.40	-1.02	-2.55
18	4.37	0.94	2.70	0.56	-3.40	-0.91	-2.59

© 2024 Quanbeck A et al. *JAMA Network Open*.

Covariance Parameter Estimates

Cov Parm	Subject	Estimate		Std Dev & Correlation		
Intercept	Clinic	0.0072	Clinic	0.0849		
UN(1,1)	PCP	0.0176				
UN(2,1)	PCP	(0.0022)				
UN(2,2)	PCP	0.0008	PCP	0.1328		
UN(3,1)	PCP	0.0016		-57.5%	0.0288	
UN(3,2)	PCP	(0.0008)		38.3%	-91.0%	0.0320
UN(3,3)	PCP	0.0010				
Residual		0.1026	Residual	0.3203		

Solution for Fixed Effects

Effect	system	Estimate	Standard Error	DF	t Value	Pr > |t|
system	Bellin	0.3030	0.0277	805	10.94	<.0001
system	UW Health	0.1238	0.0278	805	4.45	<.0001
clinic_size		0.0000	0.0000	805	-1.24	0.215
t7		0.0061	0.0032	256	1.90	0.059
PF_t7		-0.0054	0.0045	805	-1.21	0.228
t13		0.0043	0.0042	204	1.02	0.307
PF_t13		0.0041	0.0060	805	0.68	0.496
PPC_t13		-0.0106	0.0031	805	-3.40	0.001
PFandPPC_t13		0.0031	0.0045	805	0.70	0.487

Estimates

Label	Estimate	Standard Error	DF	t Value	Pr > |t|
NOINT Chg	0.1615	0.0307	805	5.26	<.0001
PF Only Chg	0.1127	0.0316	805	3.56	0.000
PPC Only Chg	0.0347	0.0303	805	1.14	0.253
PF & PPC Chg	0.0234	0.0315	805	0.74	0.459
PF Only vs NOINT	-0.0488	0.0431	805	-1.13	0.258
PPC Only vs NOINT	-0.1268	0.0373	805	-3.40	0.001

© 2024 Quanbeck A et al. *JAMA Network Open*.

**PF Only vs PPC Only 1**	0.0780	0.0428	805	1.82	0.069
**PF Only vs PPC Only 2**	0.1105	0.0387	805	2.86	0.004
**PF & PPC Interaction**	0.0374	0.0538	805	0.70	0.487
**PF & PPC vs NOINT**	-0.1381	0.0430	805	-3.21	0.001

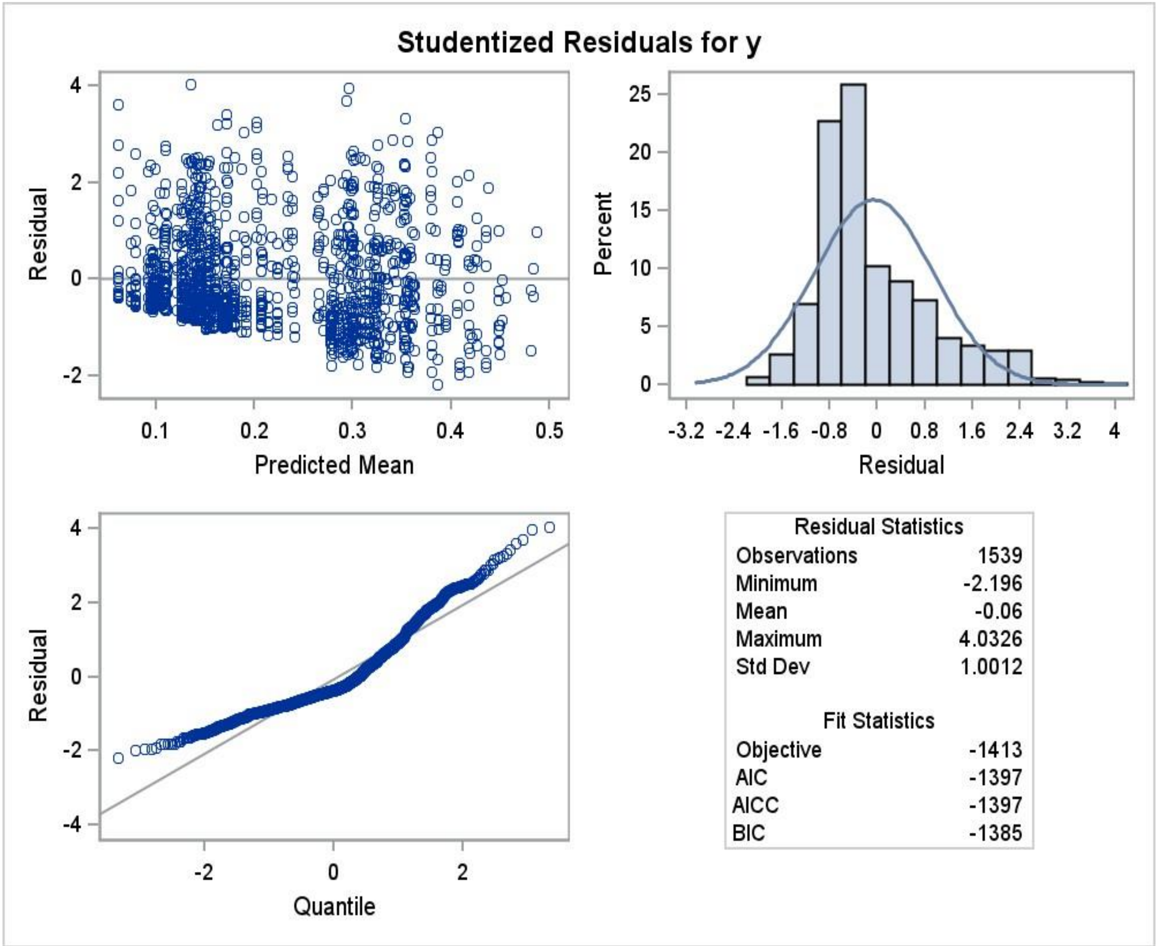

© 2024 Quanbeck A et al. *JAMA Network Open*.

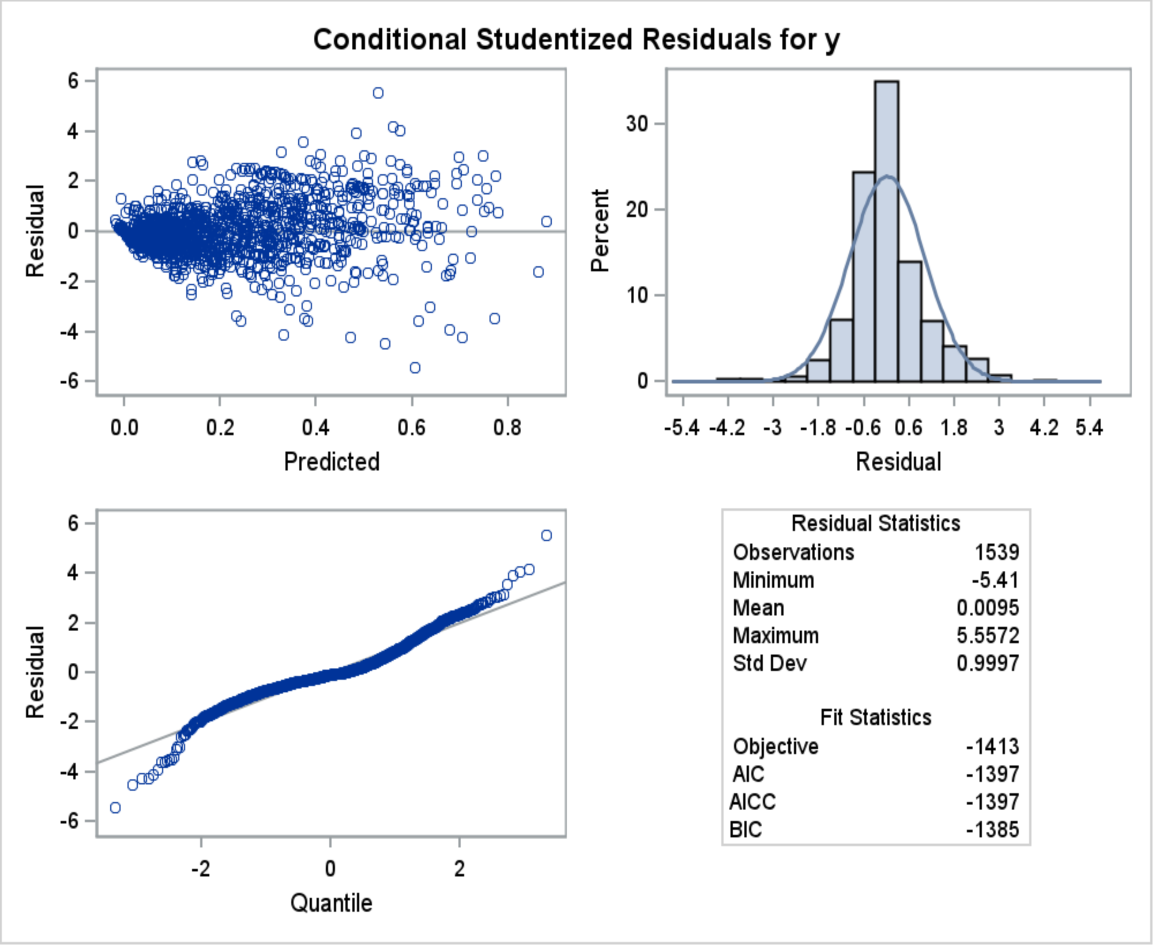

© 2024 Quanbeck A et al. *JAMA Network Open*.

Appendix A6 – Percent of Opioid Patients with PHQ-9 Depression Screen in Last 12 Months

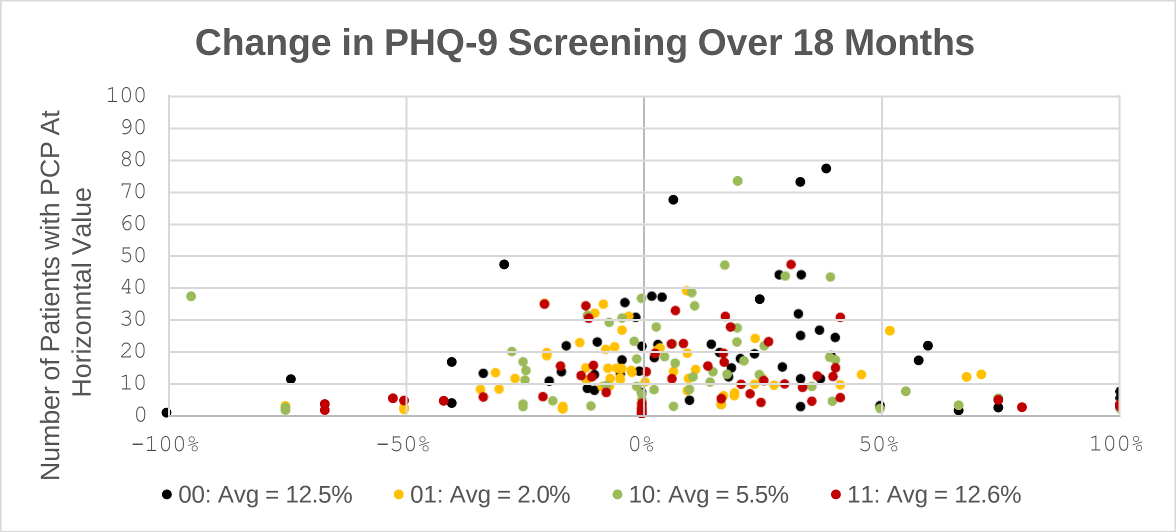

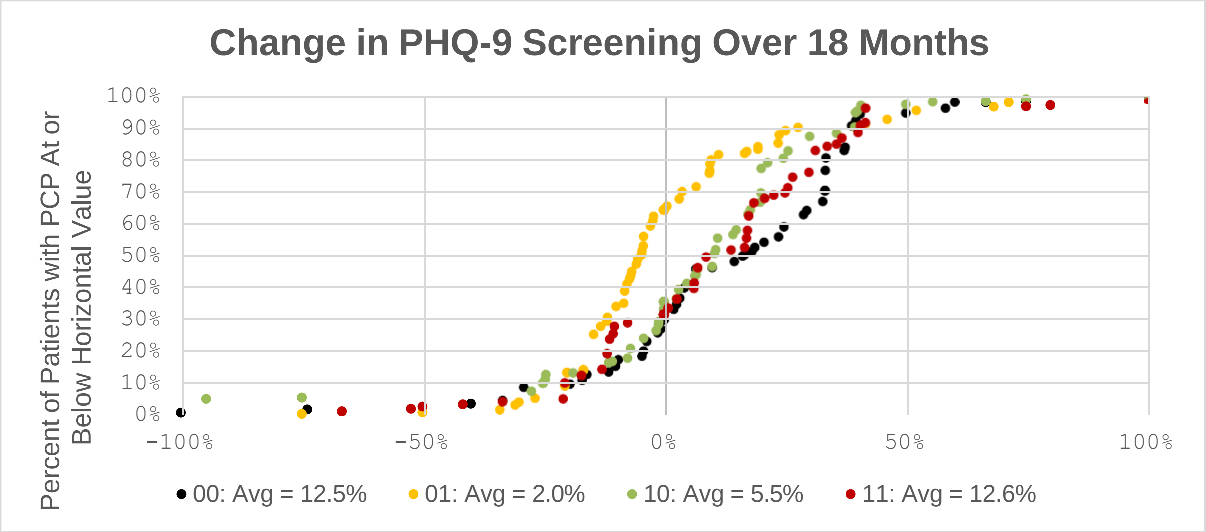

© 2024 Quanbeck A et al. *JAMA Network Open*.

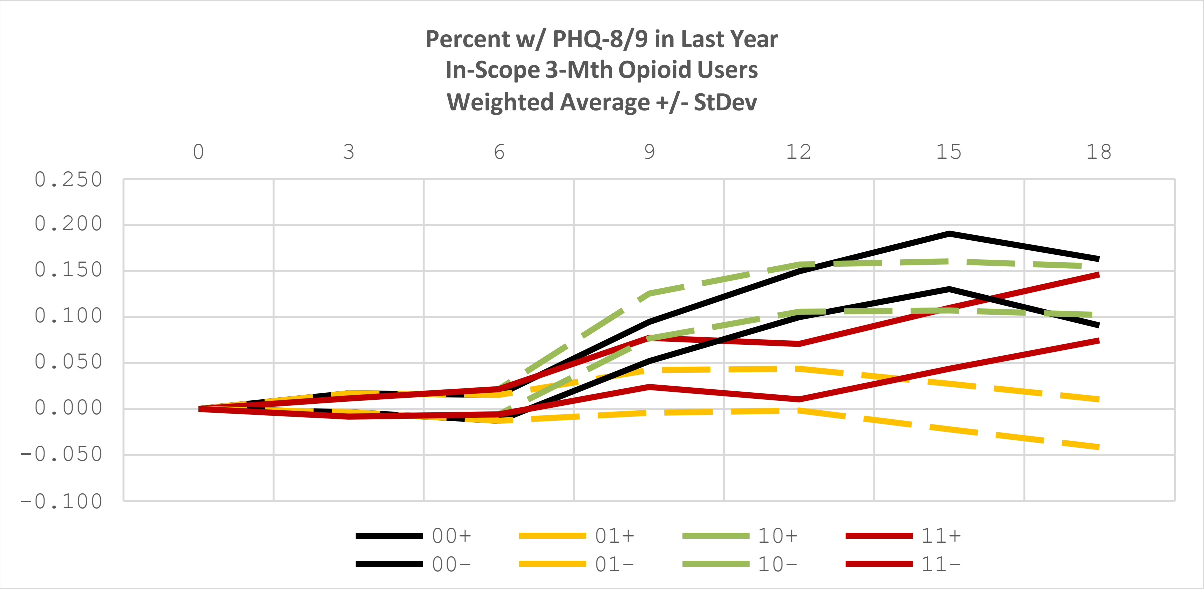

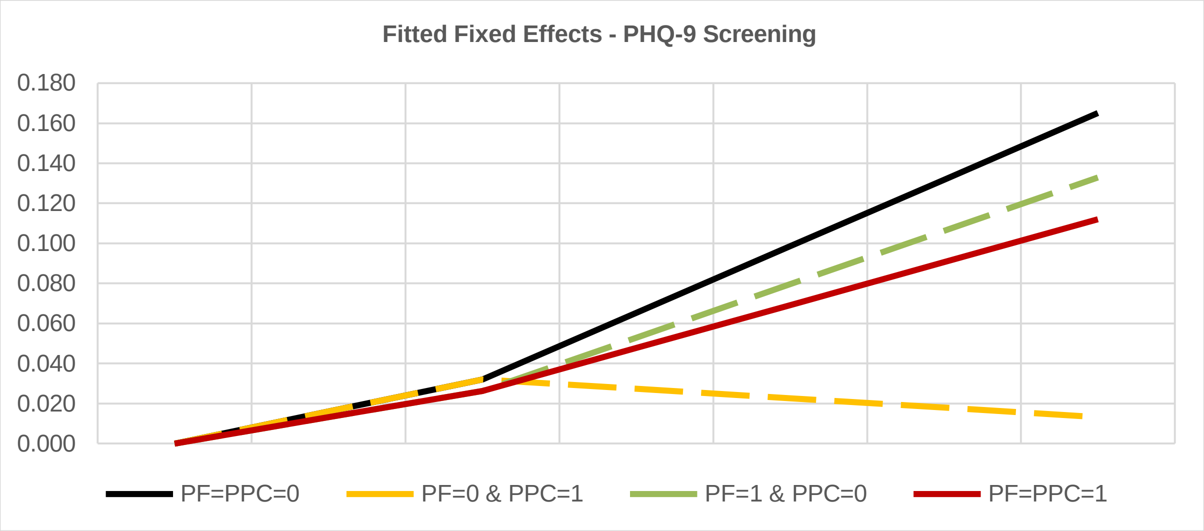

PHQ-9 Screening		
	**Estimated Fixed Effect Curves**	**Differences**

© 2024 Quanbeck A et al. *JAMA Network Open*.

Study Month	PF=PPC=0	PF=0 & PPC=1	PF=1 & PPC=0	PF=PPC=1	PF=0 & PPC=1 minus PF=PPC=0	PF=1 & PPC=0 minus PF=PPC=0	PF=PPC=1 minus PF=PPC=0
0	0.000	0.000	0.000	0.000	0.000	0.000	0.000
3	0.016	0.016	0.013	0.013	0.000	-0.003	-0.003
6	0.032	0.032	0.026	0.026	0.000	-0.006	-0.006
9	0.065	0.027	0.053	0.048	-0.038	-0.012	-0.018
12	0.099	0.023	0.080	0.069	-0.076	-0.019	-0.029
15	0.132	0.018	0.106	0.091	-0.114	-0.026	-0.041
18	0.165	0.013	0.133	0.112	-0.152	-0.032	-0.053
**Std Dev of Estimated Values**							
0	0.000	0.000	0.000	0.000	0.000	0.000	0.000
3	0.007	0.007	0.008	0.008	0.000	0.010	0.010
6	0.015	0.015	0.015	0.015	0.000	0.021	0.021
9	0.016	0.016	0.021	0.021	0.009	0.025	0.025
12	0.019	0.019	0.030	0.030	0.019	0.034	0.034
15	0.024	0.023	0.040	0.040	0.028	0.044	0.044
18	0.029	0.029	0.050	0.051	0.038	0.055	0.055
**t-ratios of Estimated Values**							
0							
3	2.17	2.17	1.71	1.71		-0.28	-0.28
6	2.17	2.17	1.71	1.71		-0.28	-0.28
9	4.10	1.74	2.50	2.26	-4.01	-0.49	-0.69
12	5.10	1.20	2.65	2.30	-4.01	-0.56	-0.86
15	5.50	0.76	2.66	2.25	-4.01	-0.58	-0.93
18	5.63	0.46	2.64	2.21	-4.01	-0.58	-0.96

© 2024 Quanbeck A et al. *JAMA Network Open*.

Covariance Parameter Estimates

Cov Parm	Subject	Estimate		Std Dev & Correlation		
Intercept	Clinic	0.0080	Clinic	0.0892		
UN(1,1)	PCP	0.0289				
UN(2,1)	PCP	(0.0012)				
UN(2,2)	PCP	0.0003	PCP	0.1699		
UN(3,1)	PCP	(0.0003)		-37.7%	0.0184	
UN(3,2)	PCP	(0.0003)		-9.8%	-73.7%	0.0199
UN(3,3)	PCP	0.0004				
Residual		0.1029	Residual	0.3208		

Solution for Fixed Effects

Effect	system	Estimate	Standard Error	DF	t Value	Pr > |t|
system	Bellin	0.3320	0.0308	805	10.77	<.0001
system	UW Health	0.2539	0.0303	805	8.39	<.0001
clinic_size		0.0000	0.0000	805	-0.36	0.719
t7		0.0053	0.0025	256	2.17	0.031
PF_t7		-0.0010	0.0035	805	-0.27	0.784
t13		0.0057	0.0034	204	1.70	0.091
PF_t13		-0.0012	0.0049	805	-0.25	0.801
PPC_t13		-0.0127	0.0032	805	-4.01	<.0001
PFandPPC_t13		0.0109	0.0046	805	2.40	0.017

Estimates

Label	Estimate	Standard Error	DF	t Value	Pr > |t|
NOINT Chg	0.1651	0.0304	805	5.42	<.0001
PF Only Chg	0.1330	0.0313	805	4.25	<.0001
PPC Only Chg	0.0132	0.0299	805	0.44	0.661
PF & PPC Chg	0.1121	0.0313	805	3.58	0.000
PF Only vs NOINT	-0.0321	0.0425	805	-0.76	0.450
PPC Only vs NOINT	-0.1519	0.0379	805	-4.01	<.0001

© 2024 Quanbeck A et al. *JAMA Network Open*.

**PF Only vs PPC Only 1**	0.1199	0.0422	805	2.84	0.005
**PF Only vs PPC Only 2**	0.1256	0.0387	805	3.25	0.001
**PF & PPC Interaction**	0.1311	0.0547	805	2.40	0.017
**PF & PPC vs NOINT**	-0.0530	0.0425	805	-1.25	0.213

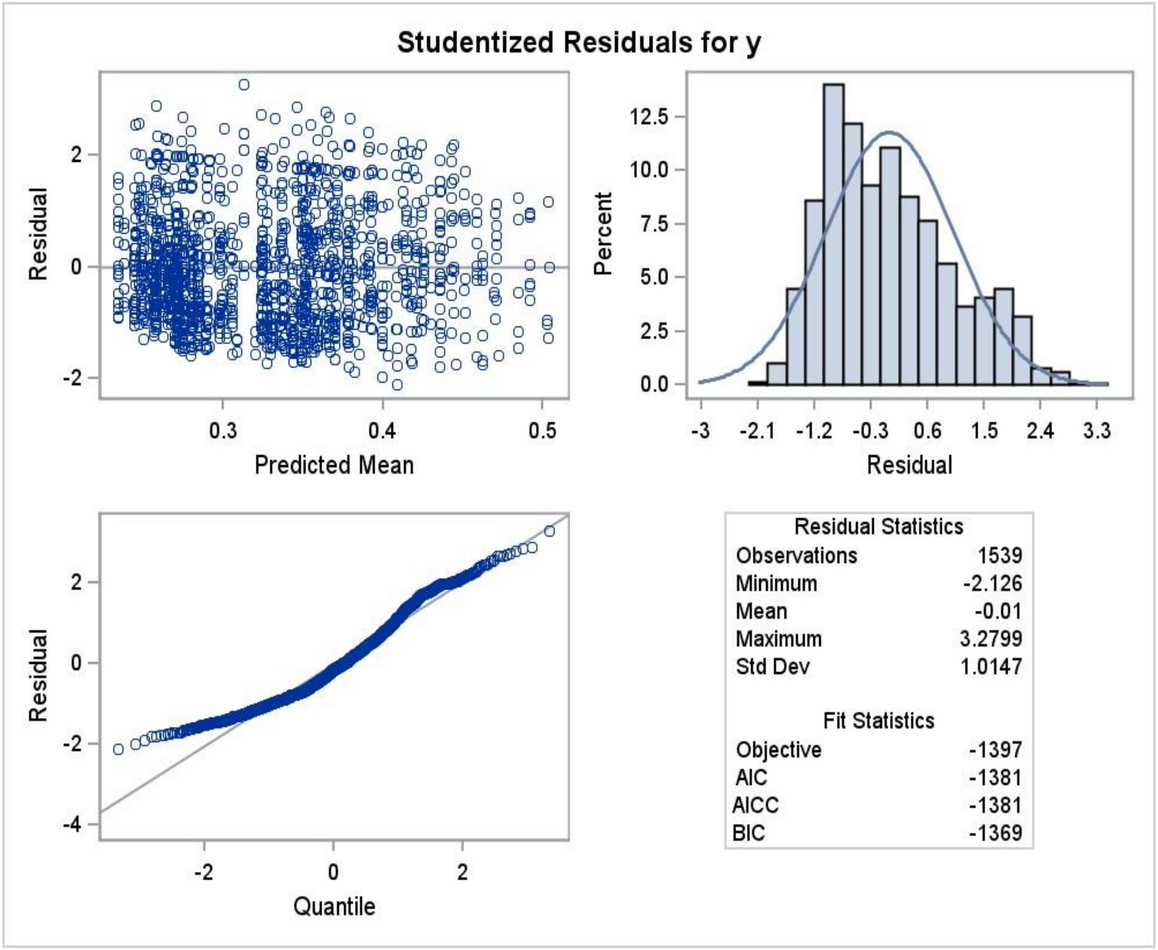

© 2024 Quanbeck A et al. *JAMA Network Open*.

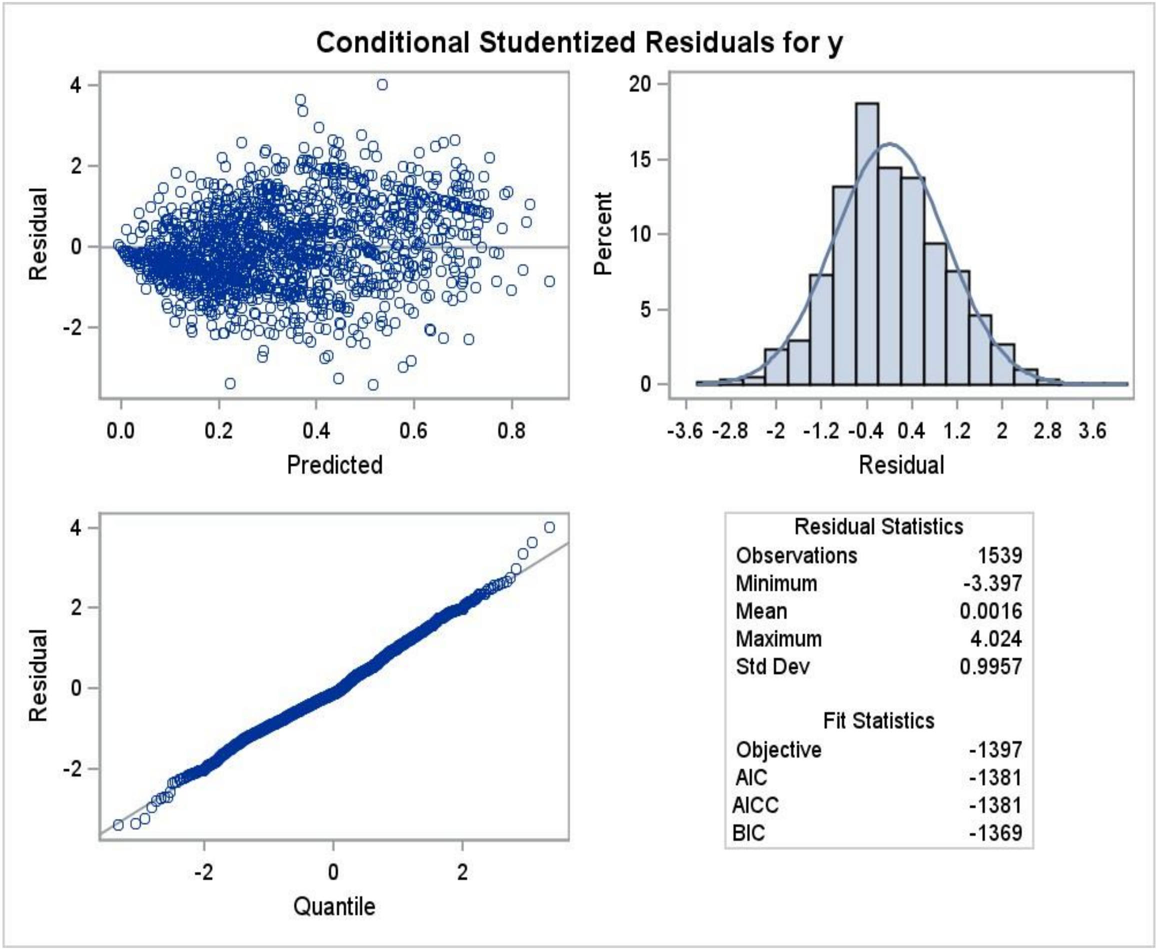

© 2024 Quanbeck A et al. *JAMA Network Open*.

Appendix A7 – Percent of Opioid Patients with PEG-3 Pain Screen in Last 12 Months

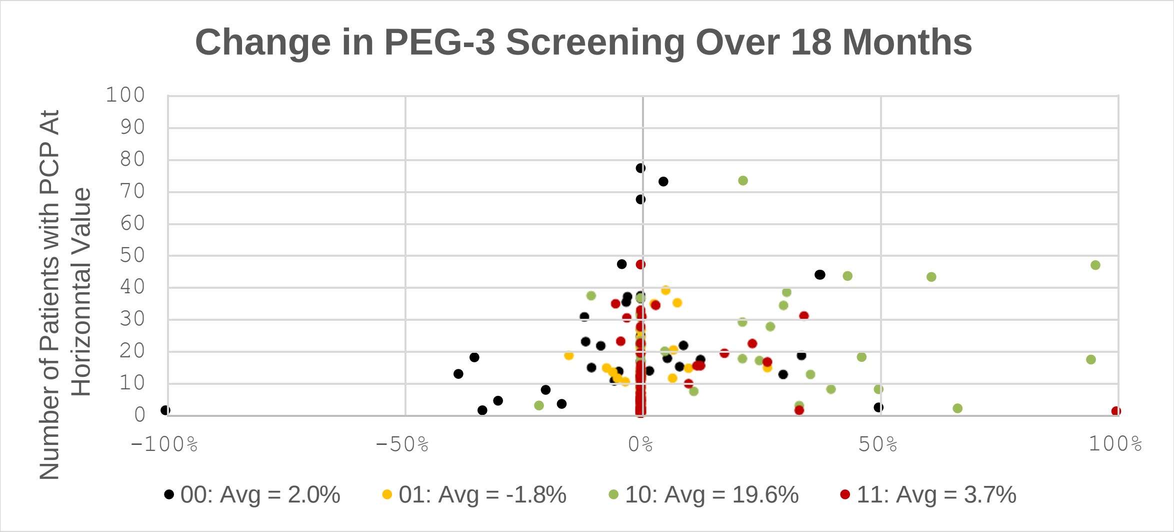

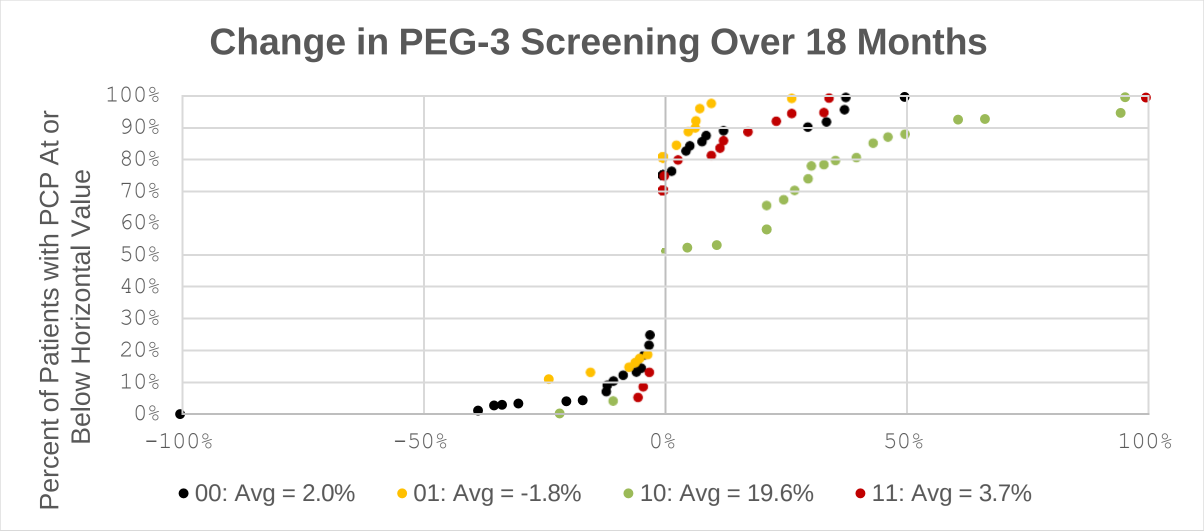

© 2024 Quanbeck A et al. *JAMA Network Open*.

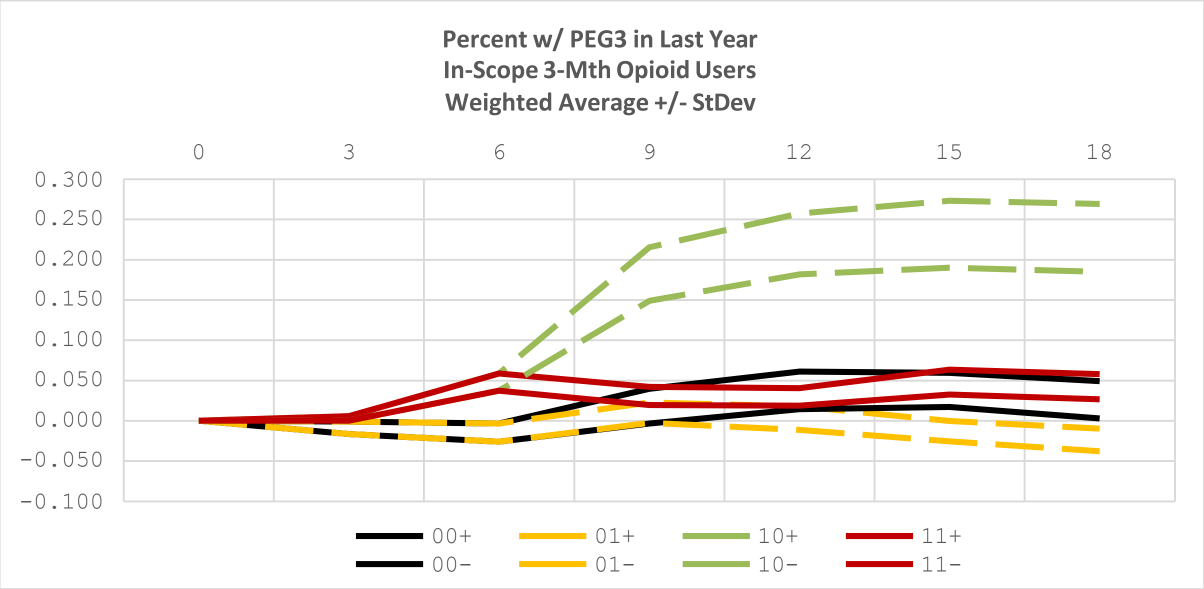

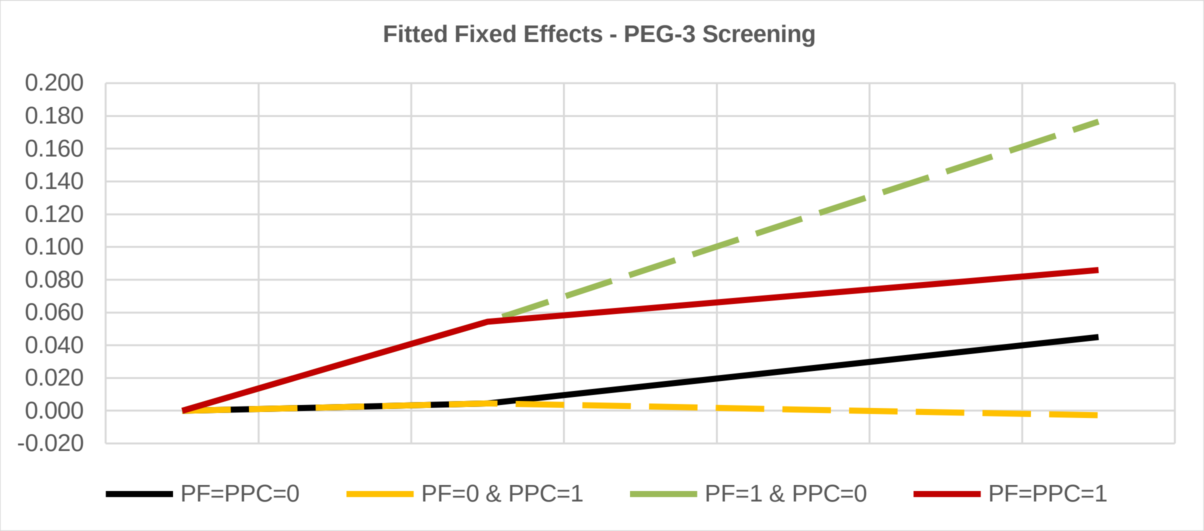

PEG-3 Screening		
	**Estimated Fixed Effect Curves**	**Differences**

© 2024 Quanbeck A et al. *JAMA Network Open*.

Study Month	PF=PPC=0	PF=0 & PPC=1	PF=1 & PPC=0	PF=PPC=1	PF=0 & PPC=1 minus PF=PPC=0	PF=1 & PPC=0 minus PF=PPC=0	PF=PPC=1 minus PF=PPC=0
0	0.000	0.000	0.000	0.000	0.000	0.000	0.000
3	0.002	0.002	0.027	0.027	0.000	0.025	0.025
6	0.004	0.004	0.054	0.054	0.000	0.050	0.050
9	0.015	0.003	0.085	0.062	-0.012	0.070	0.048
12	0.025	0.001	0.115	0.070	-0.024	0.091	0.045
15	0.035	-0.001	0.146	0.078	-0.036	0.111	0.043
18	0.045	-0.003	0.177	0.086	-0.048	0.132	0.041
**Std Dev of Estimated Values**							
0	0.000	0.000	0.000	0.000	0.000	0.000	0.000
3	0.006	0.006	0.007	0.007	0.000	0.009	0.009
6	0.013	0.013	0.013	0.013	0.000	0.018	0.018
9	0.014	0.013	0.014	0.014	0.007	0.019	0.019
12	0.016	0.016	0.017	0.016	0.014	0.023	0.023
15	0.019	0.019	0.021	0.020	0.021	0.028	0.028
18	0.023	0.023	0.025	0.024	0.028	0.034	0.033
**t-ratios of Esimated Values**							
0							
3	0.35	0.35	4.14	4.14		2.78	2.78
6	0.35	0.35	4.14	4.14		2.78	2.78
9	1.08	0.20	6.02	4.48	-1.72	3.64	2.49
12	1.54	0.05	6.86	4.29	-1.72	3.94	2.00
15	1.79	-0.05	7.08	3.91	-1.72	3.95	1.56
18	1.93	-0.12	7.06	3.56	-1.72	3.86	1.22

© 2024 Quanbeck A et al. *JAMA Network Open*.

Covariance Parameter Estimates

Cov Parm	Subject	Estimate		Std Dev & Correlation		
Intercept	Clinic	0.0081	Clinic	0.0902		
UN(1,1)	PCP	0.0107				
UN(2,1)	PCP	(0.0007)				
UN(2,2)	PCP	0.0003	PCP	0.1035		
UN(3,1)	PCP	0.0006		-35.9%	0.0177	
UN(3,2)	PCP	(0.0003)		30.3%	-86.5%	0.0182
UN(3,3)	PCP	0.0003				
Residual		0.0479	Residual	0.2189		

Solution for Fixed Effects

Effect	system	Estimate	Standard Error	DF	t Value	Pr > |t|
system	Bellin	0.0124	0.0274	805	0.45	0.6505
system	UW Health	0.0577	0.0281	805	2.06	0.0401
clinic_size		0.0000	0.0000	805	1.25	0.213
t7		0.0007	0.0021	256	0.35	0.726
PF_t7		0.0083	0.0030	805	2.78	0.006
t13		0.0027	0.0027	204	1.00	0.318
PF_t13		-0.0015	0.0038	805	-0.40	0.690
PPC_t13		-0.0040	0.0023	805	-1.71	0.087
PFandPPC_t13		-0.0036	0.0033	805	-1.07	0.285

Estimates

Label	Estimate	Standard Error	DF	t Value	Pr > |t|
NOINT Chg	0.0451	0.0234	805	1.92	0.055
PF Only Chg	0.1766	0.0241	805	7.34	<.0001
PPC Only Chg	-0.0027	0.0230	805	-0.12	0.908
PF & PPC Chg	0.0859	0.0237	805	3.63	0.000
PF Only vs NOINT	0.1315	0.0334	805	3.94	<.0001
PPC Only vs NOINT	-0.0477	0.0278	805	-1.71	0.087

© 2024 Quanbeck A et al. *JAMA Network Open*.

**PF Only vs PPC Only 1**	0.1792	0.0331	805	5.41	<.0001
**PF Only vs PPC Only 2**	0.1293	0.0284	805	4.56	<.0001
**PF & PPC Interaction**	-0.0429	0.0401	805	-1.07	0.285
**PF & PPC vs NOINT**	0.0409	0.0332	805	1.23	0.218

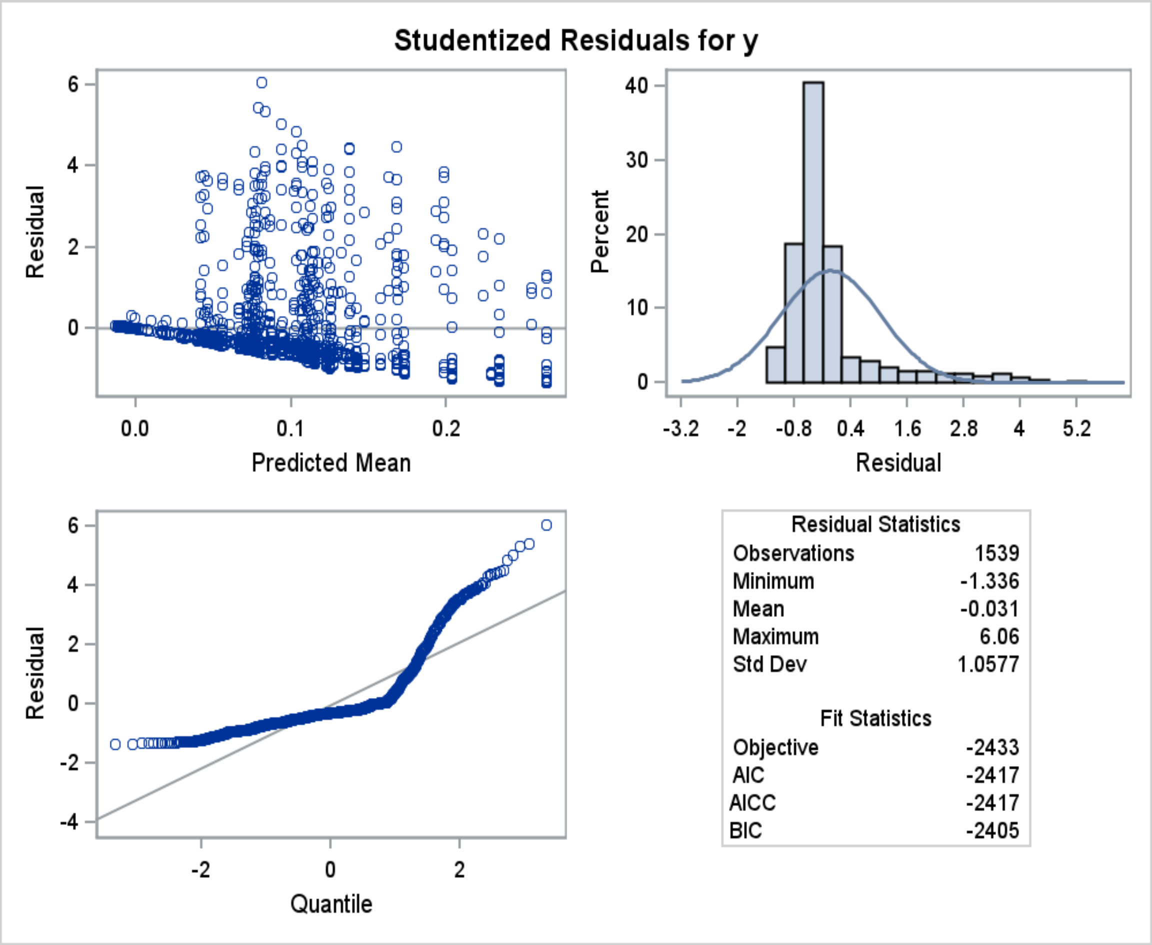

© 2024 Quanbeck A et al. *JAMA Network Open*.

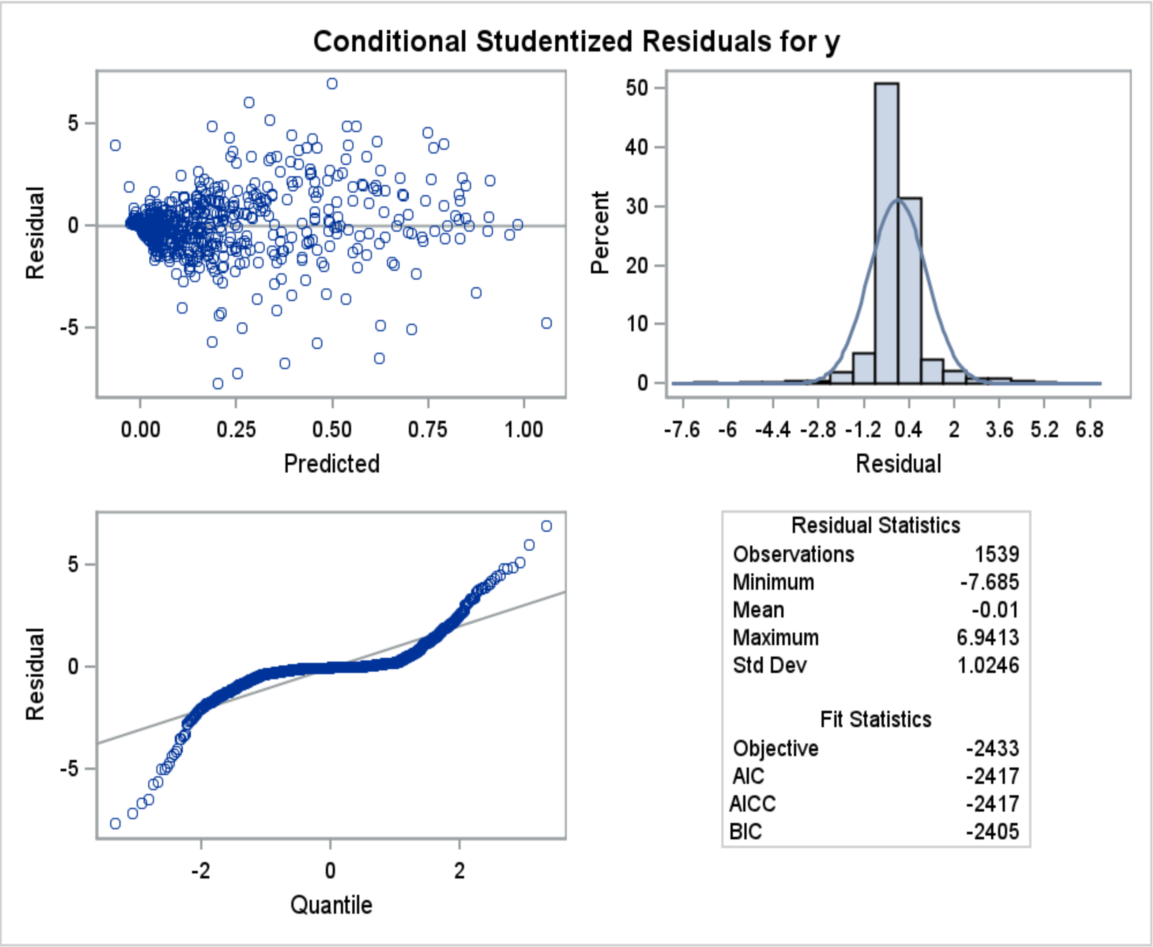

© 2024 Quanbeck A et al. *JAMA Network Open*.

Appendix B – Primary Model Results (All Opioid Patients)

© 2024 Quanbeck A et al. *JAMA Network Open*.

Appendix B1 – Average MME Prescribed in Last 3 Months (All Opioid Patients)

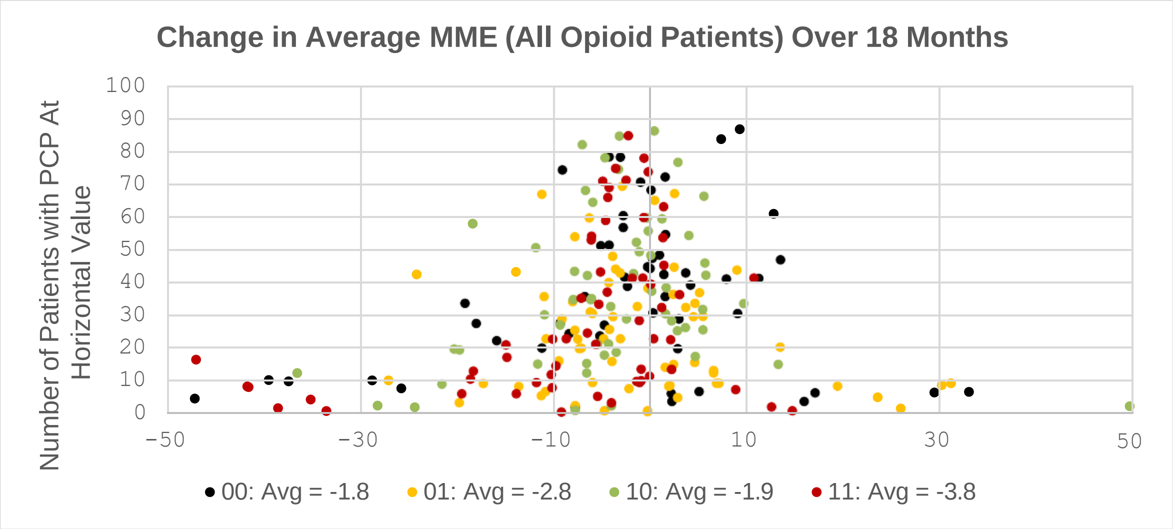

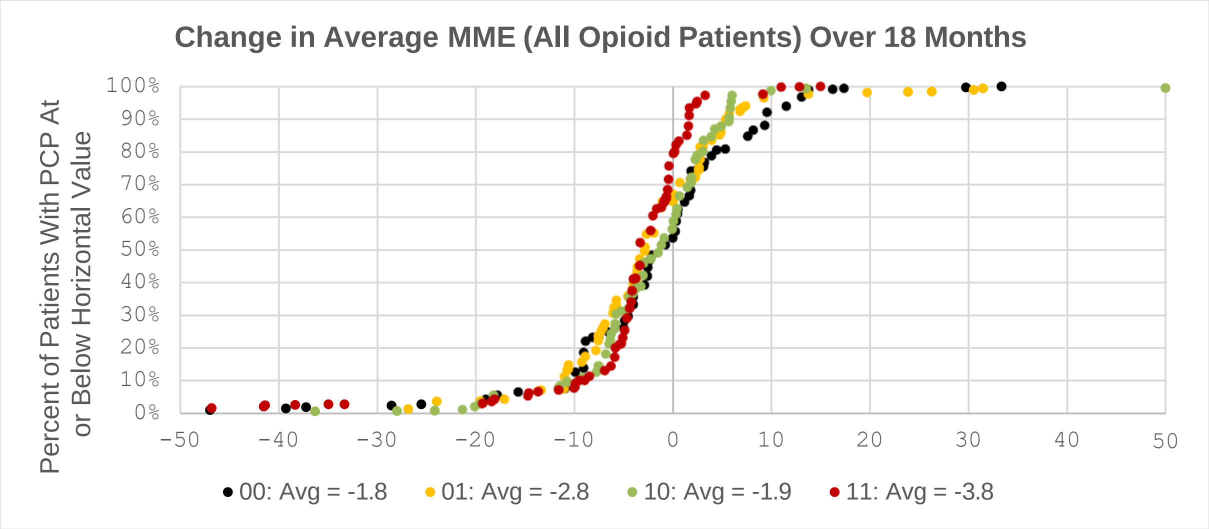

© 2024 Quanbeck A et al. *JAMA Network Open*.

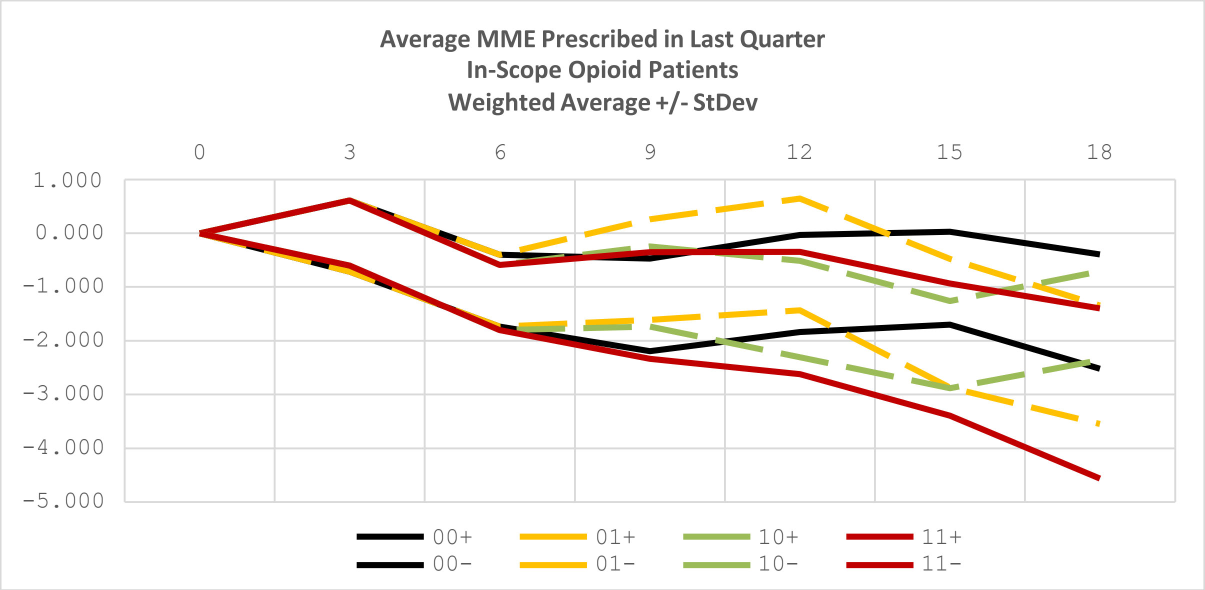

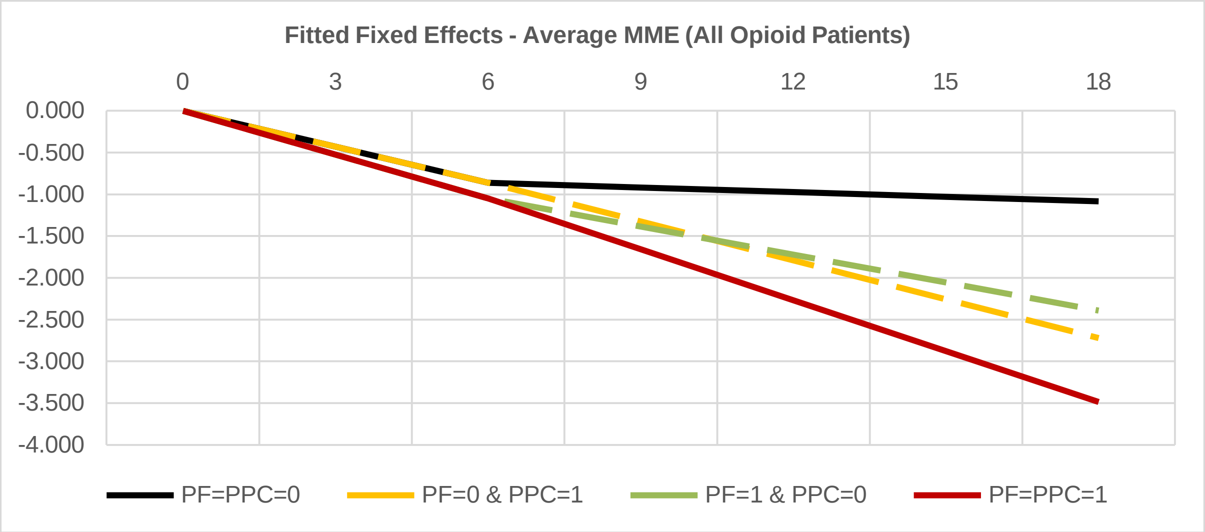

© 2024 Quanbeck A et al. *JAMA Network Open*.

Average MME (All Opioid Patients)							
Study Month	Estimated Fixed Effect Curves				Differences		
	PF=PPC=0	PF=0 & PPC=1	PF=1 & PPC=0	PF=PPC=1	PF=0 & PPC=1 minus PF=PPC=0	PF=1 & PPC=0 minus PF=PPC=0	PF=PPC=1 minus PF=PPC=0
0	0.000	0.000	0.000	0.000	0.000	0.000	0.000
3	-0.431	-0.431	-0.525	-0.525	0.000	-0.094	-0.094
6	-0.862	-0.862	-1.050	-1.050	0.000	-0.189	-0.189
9	-0.917	-1.327	-1.386	-1.659	-0.409	-0.468	-0.742
12	-0.973	-1.792	-1.721	-2.268	-0.818	-0.748	-1.295
15	-1.029	-2.257	-2.056	-2.877	-1.228	-1.027	-1.848
18	-1.085	-2.722	-2.392	-3.486	-1.637	-1.307	-2.402
Std Dev of Estimated Values							
0	0.000	0.000	0.000	0.000	0.000	0.000	0.000
3	0.260	0.260	0.252	0.252	0.000	0.352	0.352
6	0.519	0.519	0.505	0.505	0.000	0.703	0.703
9	0.493	0.493	0.480	0.482	0.231	0.661	0.662
12	0.523	0.529	0.512	0.521	0.462	0.700	0.708
15	0.601	0.618	0.590	0.610	0.693	0.810	0.825
18	0.711	0.740	0.699	0.731	0.924	0.965	0.990
t-ratios of Estimated Values							
0							
3	-1.66	-1.66	-2.08	-2.08		-0.27	-0.27
6	-1.66	-1.66	-2.08	-2.08		-0.27	-0.27
9	-1.86	-2.69	-2.88	-3.44	-1.77	-0.71	-1.12
12	-1.86	-3.38	-3.36	-4.35	-1.77	-1.07	-1.83
15	-1.71	-3.65	-3.49	-4.71	-1.77	-1.27	-2.24
18	-1.53	-3.68	-3.42	-4.77	-1.77	-1.35	-2.43

© 2024 Quanbeck A et al. *JAMA Network Open*.

Covariance Parameter Estimates						
Cov Parm	Subject	Estimate		Std Dev & Correlation		
Intercept	Clinic	3.6	Clinic	1.90		
UN(1,1)	PCP	85.4				
UN(2,1)	PCP	(0.83)				
UN(2,2)	PCP	0.066	PCP	9.24		
UN(3,1)	PCP	(0.18)		-35.0%	0.26	
UN(3,2)	PCP	(0.02)		#DIV/0!	#DIV/0!	-
UN(3,3)	PCP	-				
Residual		628.6	Residual	25.07		

Solution for Fixed Effects						
Effect	system	Estimate	Standard Error	DF	t Value	Pr > |t|
system	Bellin	31.948	1.176	865	27.17	<.0001
system	UW Health	43.227	1.002	865	43.15	<.0001
clinic_size		0.000	0.000	865	-0.51	0.613
t7		-0.144	0.087	263	-1.66	0.098
PF_t7		-0.031	0.117	865	-0.27	0.789
t13		0.125	0.117	214	1.07	0.287
PF_t13		-0.062	0.162	865	-0.38	0.703
PPC_t13		-0.136	0.077	865	-1.77	0.077
PFandPPC_t13		0.045	0.108	865	0.42	0.676

© 2024 Quanbeck A et al. *JAMA Network Open*.

Estimates					
**Label**	**Estimate**	**Standard Error**	**DF**	**t Value**	**Pr > |t|**
**NOINT Chg**	-1.0854	0.7118	865	-1.52	0.1276
**PF Only Chg**	-2.3925	0.7002	865	-3.42	0.0007
**PPC Only Chg**	-2.7227	0.7406	865	-3.68	0.0003
**PF & PPC Chg**	-3.4877	0.7310	865	-4.77	<.0001
**PF Only vs NOINT**	-1.3071	0.9645	865	-1.36	0.1757
**PPC Only vs NOINT**	-1.6373	0.9237	865	-1.77	0.0766
**PF Only vs PPC Only 1**	0.3302	0.9868	865	0.33	0.7380
**PF Only vs PPC Only 2**	0.5187	0.9748	865	0.53	0.5948
**PF & PPC Interaction**	0.5421	1.2951	865	0.42	0.6756
**PF & PPC vs NOINT**	-2.4023	0.9879	865	-2.43	0.0152

© 2024 Quanbeck A et al. *JAMA Network Open*.

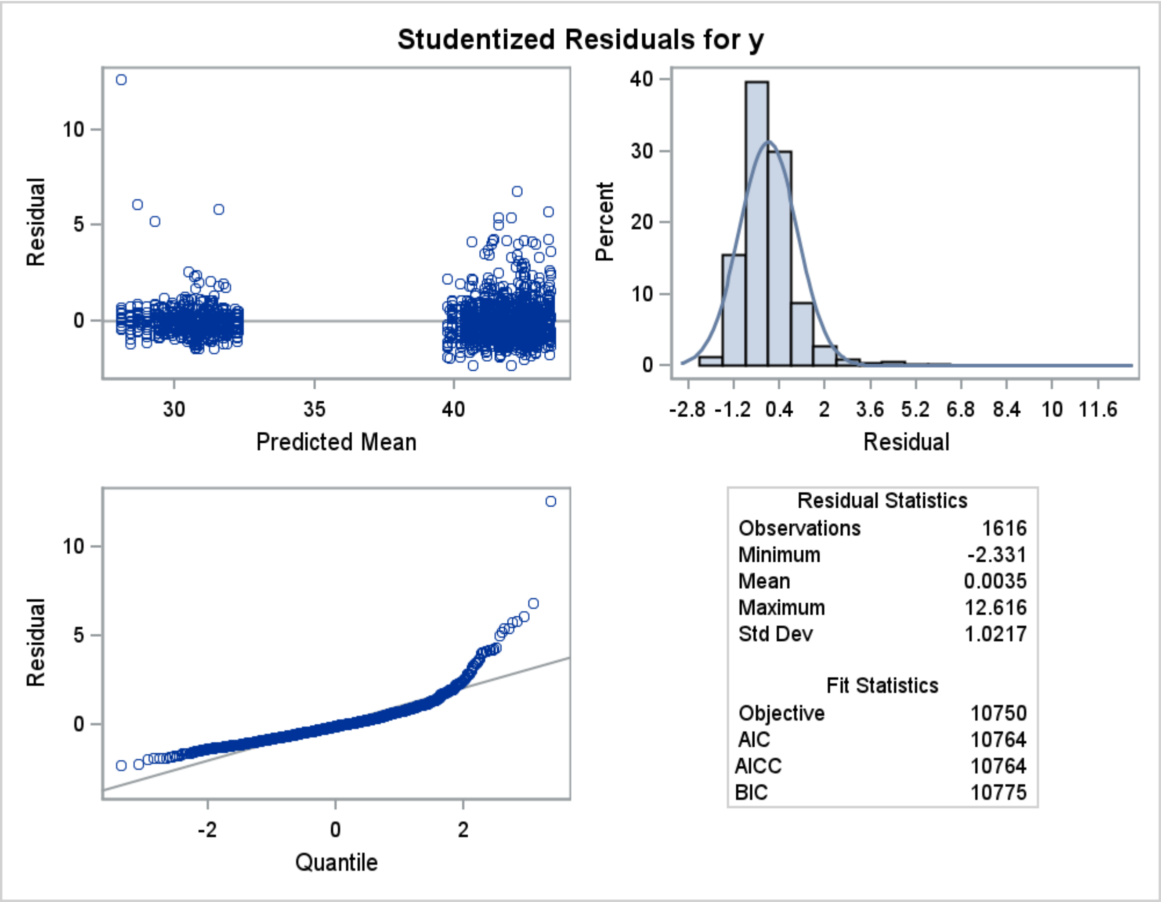

© 2024 Quanbeck A et al. *JAMA Network Open*.

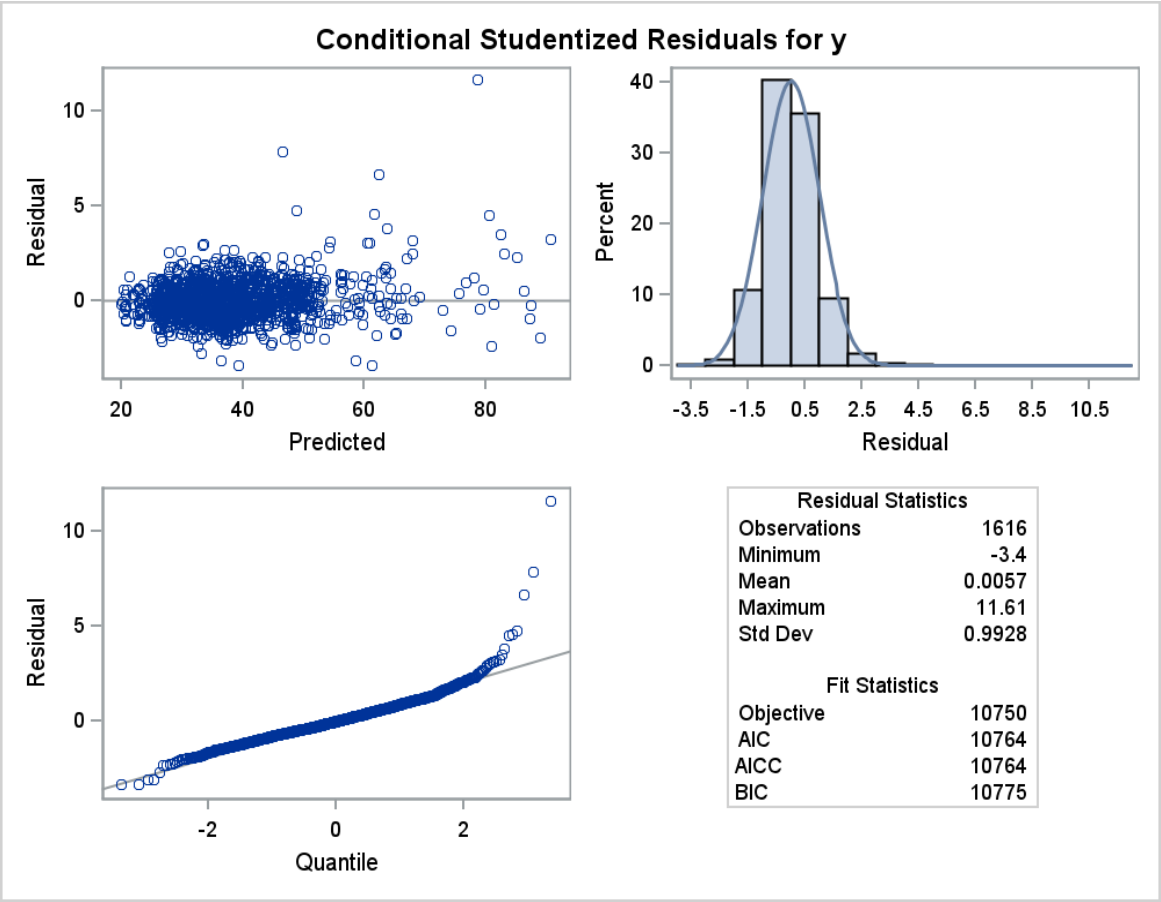

© 2024 Quanbeck A et al. *JAMA Network Open*.

Appendix B2 – Percent of Opioid Patients with Average MME ≥ 90 mg/day (All Opioid Patients)

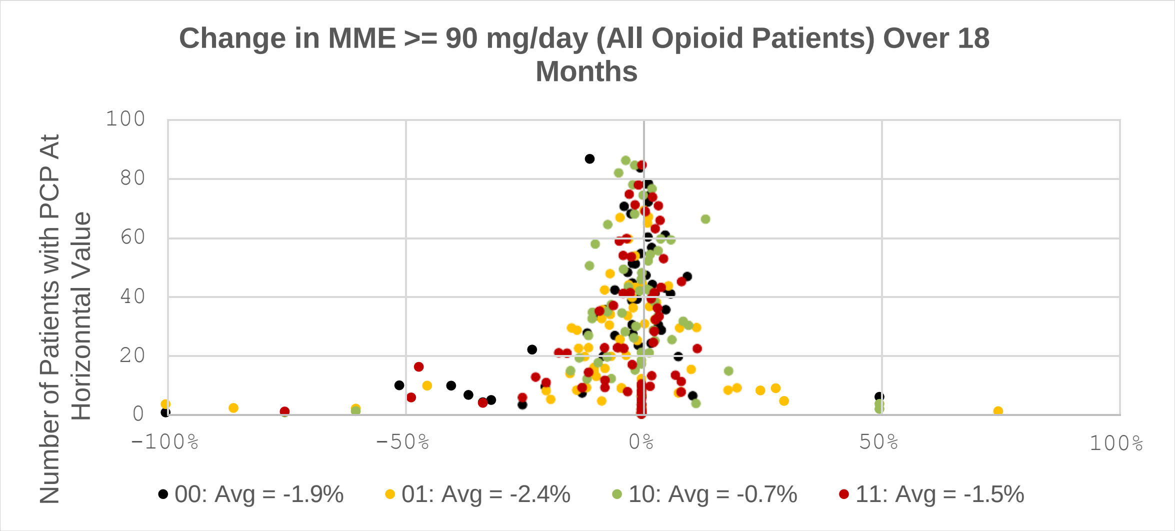

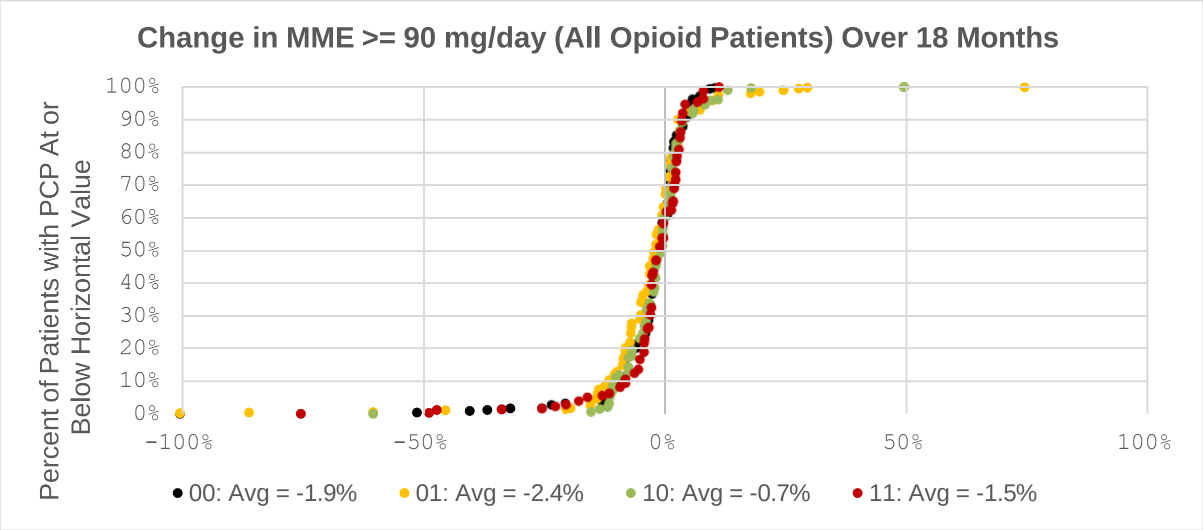

© 2024 Quanbeck A et al. *JAMA Network Open*.

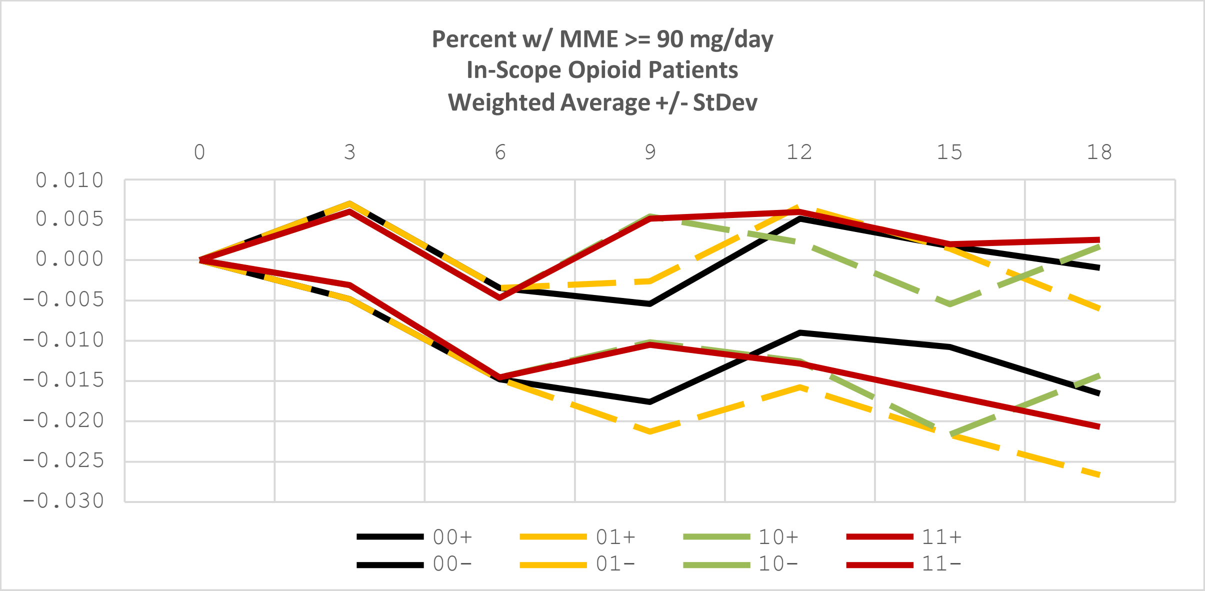

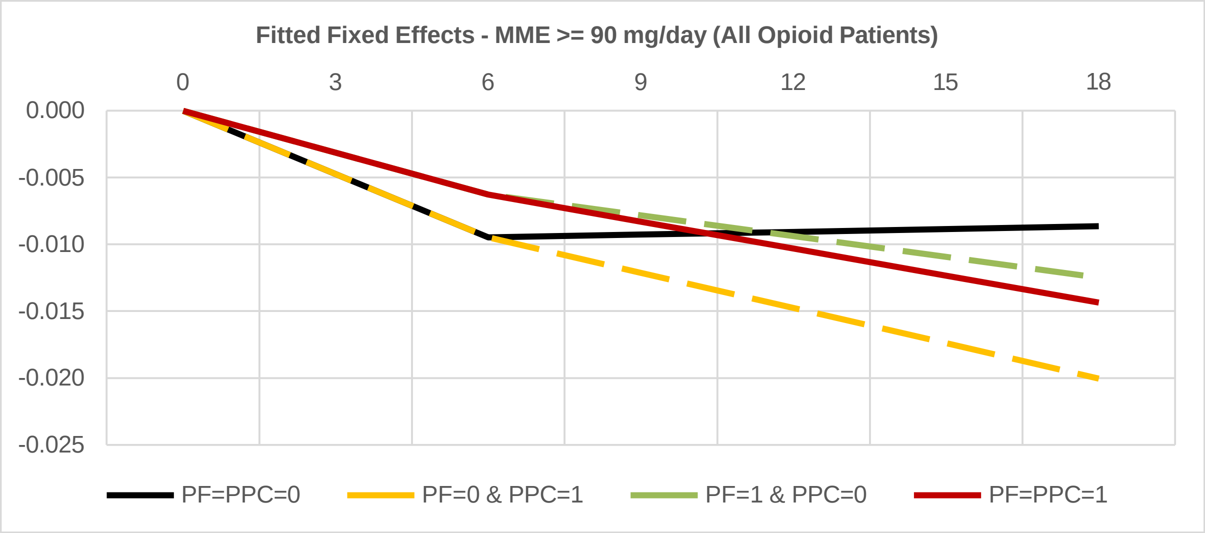

© 2024 Quanbeck A et al. *JAMA Network Open*.

MME >= 90 mg/day (All Opioid Patients)							
Study Month	Estimated Fixed Effect Curves				Differences		
	PF=PPC=0	PF=0 & PPC=1	PF=1 & PPC=0	PF=PPC=1	PF=0 & PPC=1 minus PF=PPC=0	PF=1 & PPC=0 minus PF=PPC=0	PF=PPC=1 minus PF=PPC=0
0	0.000	0.000	0.000	0.000	0.000	0.000	0.000
3	-0.005	-0.005	-0.003	-0.003	0.000	0.002	0.002
6	-0.009	-0.009	-0.006	-0.006	0.000	0.003	0.003
9	-0.009	-0.012	-0.008	-0.008	-0.003	0.001	0.001
12	-0.009	-0.015	-0.009	-0.010	-0.006	0.000	-0.001
15	-0.009	-0.017	-0.011	-0.012	-0.009	-0.002	-0.003
18	-0.009	-0.020	-0.012	-0.014	-0.011	-0.004	-0.006
Std Dev of Estimated Values							
0	0.000	0.000	0.000	0.000	0.000	0.000	0.000
3	0.002	0.002	0.002	0.002	0.000	0.003	0.003
6	0.004	0.004	0.004	0.004	0.000	0.006	0.006
9	0.004	0.004	0.004	0.004	0.002	0.005	0.005
12	0.004	0.004	0.004	0.004	0.004	0.005	0.005
15	0.005	0.005	0.004	0.005	0.006	0.006	0.006
18	0.006	0.006	0.005	0.006	0.007	0.007	0.008
t-ratios of Estimated Values							
0							
3	-2.23	-2.23	-1.52	-1.52		0.56	0.56
6	-2.23	-2.23	-1.52	-1.52		0.56	0.56
9	-2.39	-3.11	-2.08	-2.19	-1.53	0.28	0.19
12	-2.26	-3.60	-2.41	-2.58	-1.53	-0.06	-0.24
15	-1.91	-3.62	-2.44	-2.62	-1.53	-0.35	-0.56
18	-1.55	-3.43	-2.32	-2.52	-1.53	-0.53	-0.76

© 2024 Quanbeck A et al. *JAMA Network Open*.

Covariance Parameter Estimates						
Cov Parm	Subject	Estimate		Std Dev & Correlation		
Intercept	Clinic	0.0001	Clinic	0.0077		
UN(1,1)	PCP	0.0019				
UN(2,1)	PCP	0.0000				
UN(2,2)	PCP	-	PCP	0.0431		
UN(3,1)	PCP	(0.0001)		#DIV/0!	-	
UN(3,2)	PCP	0.0000		#DIV/0!	#DIV/0!	-
UN(3,3)	PCP	-				
Residual		0.0479	Residual	0.2188		

Solution for Fixed Effects						
Effect	system	Estimate	Standard Error	DF	t Value	Pr > |t|
system	Bellin	0.0484	0.0059	865	8.23	<.0001
system	UW Health	0.1141	0.0051	865	22.42	<.0001
clinic_size		0.0000	0.0000	865	-Infty	<.0001
t7		-0.0016	0.0007	263	-2.23	0.027
PF_t7		0.0005	0.0010	865	0.56	0.576
t13		0.0017	0.0010	214	1.63	0.105
PF_t13		-0.0011	0.0014	865	-0.80	0.424
PPC_t13		-0.0010	0.0006	865	-1.53	0.125
PFandPPC_t13		0.0008	0.0009	865	0.92	0.360

© 2024 Quanbeck A et al. *JAMA Network Open*.

Estimates					
**Label**	**Estimate**	**Standard Error**	**DF**	**t Value**	**Pr > |t|**
**NOINT Chg**	-0.0087	0.0056	865	-1.55	0.121
**PF Only Chg**	-0.0125	0.0055	865	-2.28	0.023
**PPC Only Chg**	-0.0200	0.0058	865	-3.43	0.001
**PF & PPC Chg**	-0.0144	0.0058	865	-2.49	0.013
**PF Only vs NOINT**	-0.0038	0.0074	865	-0.52	0.603
**PPC Only vs NOINT**	-0.0114	0.0074	865	-1.53	0.125
**PF Only vs PPC Only 1**	0.0076	0.0076	865	1.00	0.318
**PF Only vs PPC Only 2**	0.0044	0.0083	865	0.53	0.598
**PF & PPC Interaction**	0.0095	0.0104	865	0.92	0.360
**PF & PPC vs NOINT**	-0.0057	0.0076	865	-0.75	0.454

© 2024 Quanbeck A et al. *JAMA Network Open*.

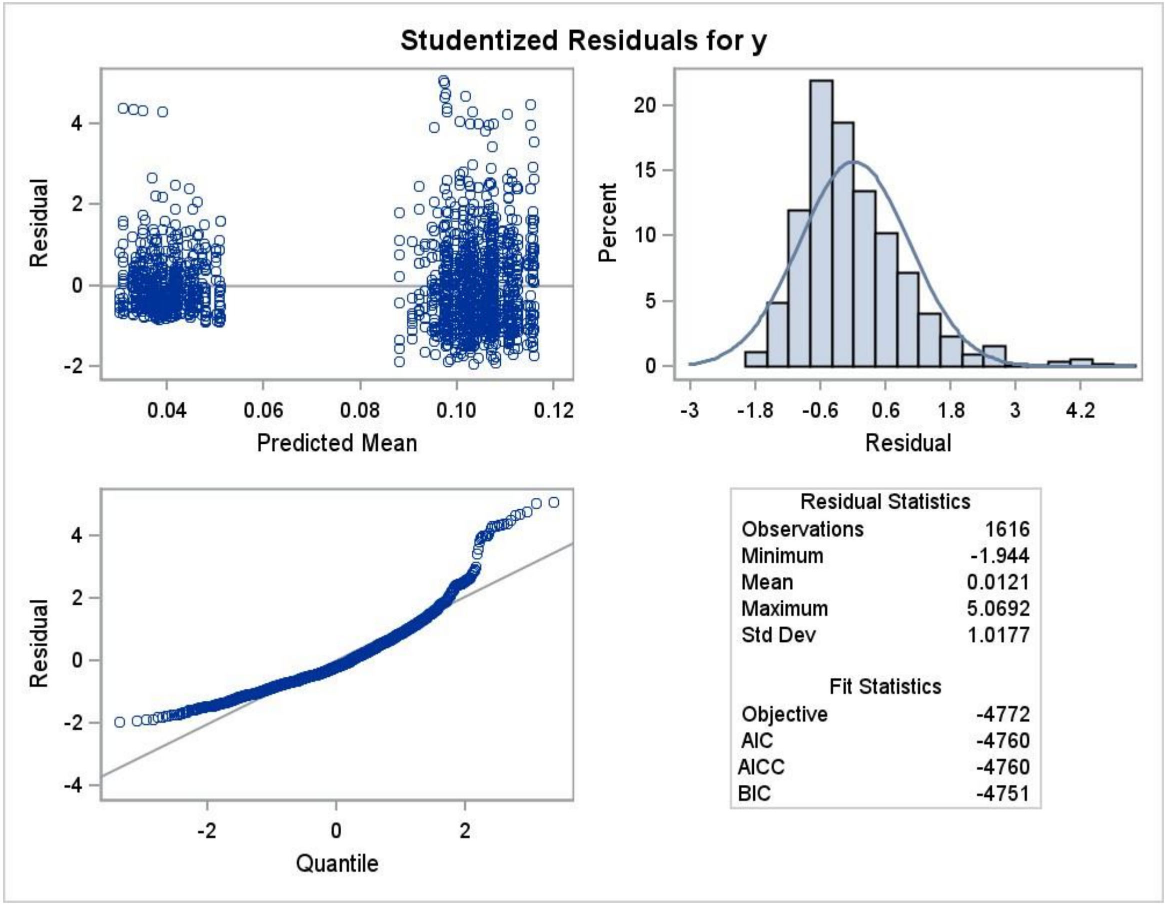

© 2024 Quanbeck A et al. *JAMA Network Open*.

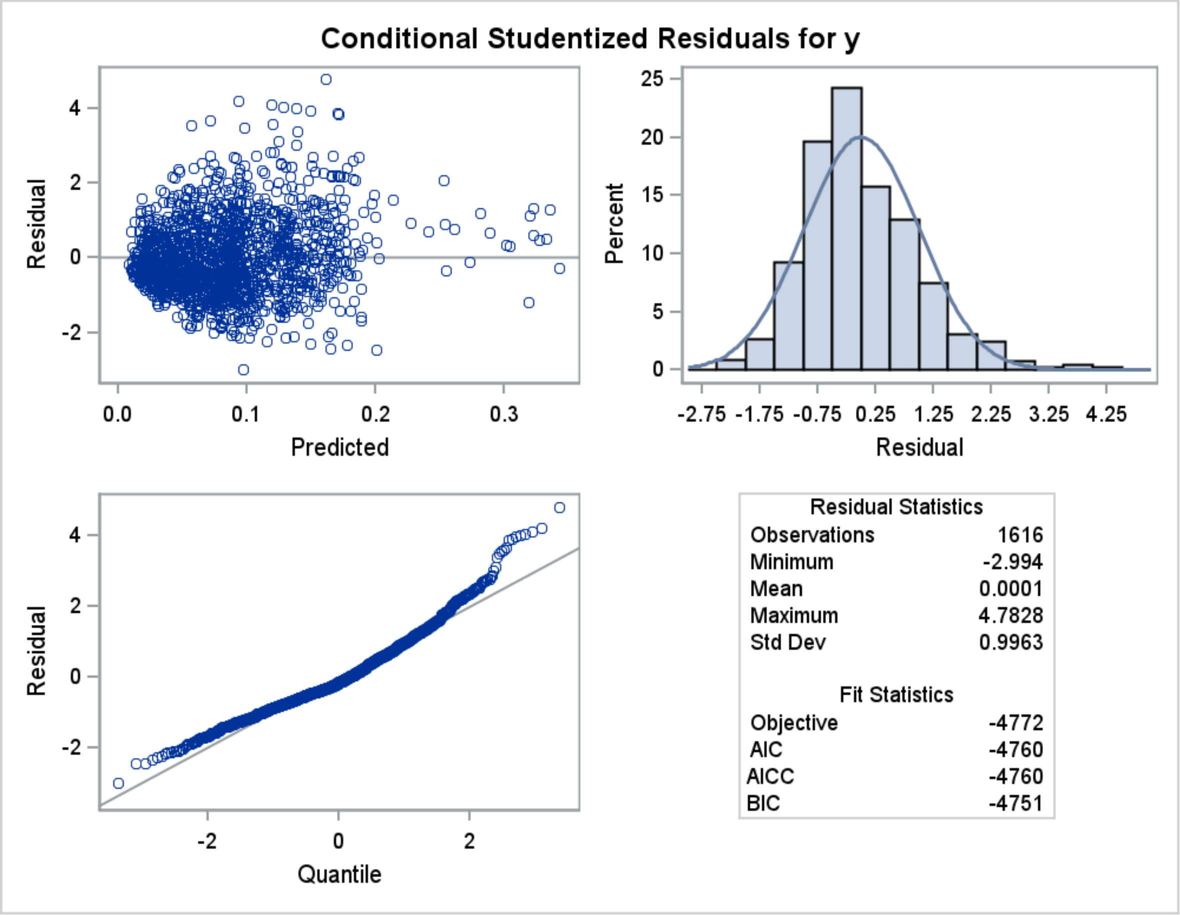

Appendix B3 – Percent of Opioid Patients with Benzo’s in Last 3 Months (All Opioid Patients)

© 2024 Quanbeck A et al. *JAMA Network Open*.

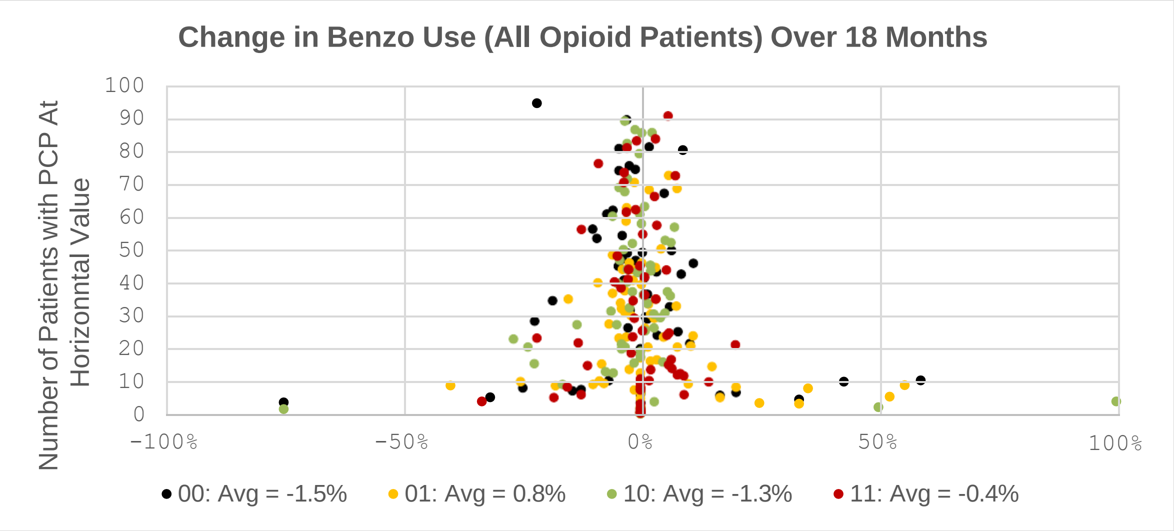

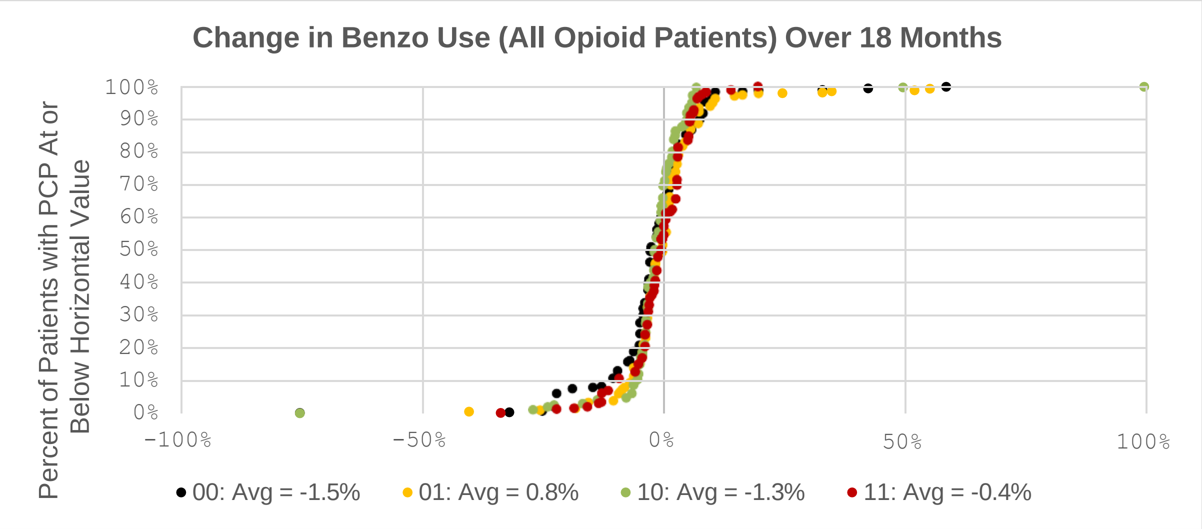

© 2024 Quanbeck A et al. *JAMA Network Open*.

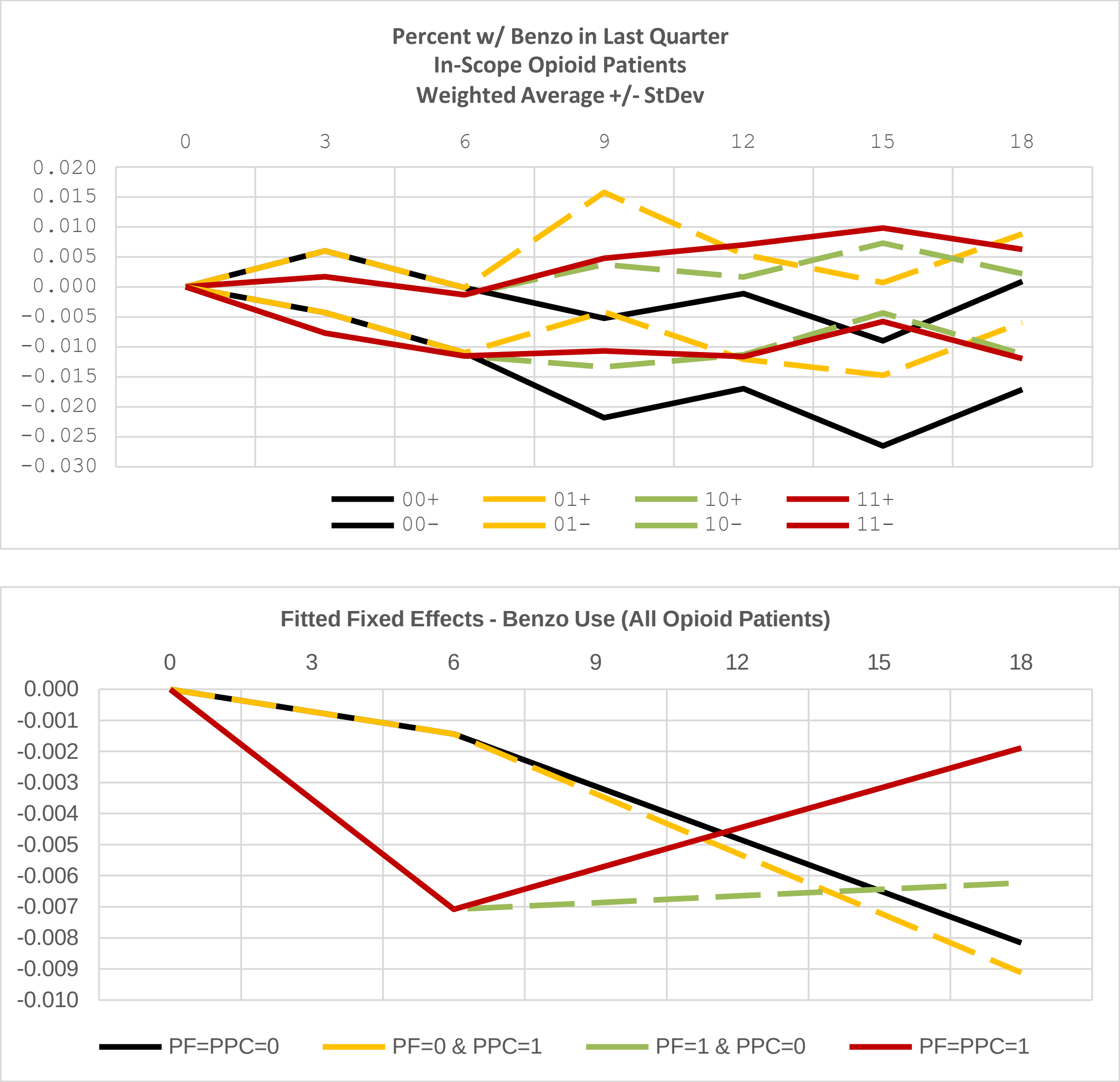

© 2024 Quanbeck A et al. *JAMA Network Open*.

Benzo Use (All Opioid Patients)							
Study Month	Estimated Fixed Effect Curves				Differences		
	PF=PPC=0	PF=0 & PPC=1	PF=1 & PPC=0	PF=PPC=1	PF=0 & PPC=1 minus PF=PPC=0	PF=1 & PPC=0 minus PF=PPC=0	PF=PPC=1 minus PF=PPC=0
0	0.000	0.000	0.000	0.000	0.000	0.000	0.000
3	-0.001	-0.001	-0.004	-0.004	0.000	-0.003	-0.003
6	-0.001	-0.001	-0.007	-0.007	0.000	-0.006	-0.006
9	-0.003	-0.003	-0.007	-0.006	0.000	-0.004	-0.003
12	-0.005	-0.005	-0.007	-0.004	0.000	-0.002	0.000
15	-0.006	-0.007	-0.006	-0.003	-0.001	0.000	0.003
18	-0.008	-0.009	-0.006	-0.002	-0.001	0.002	0.006
Std Dev of Estimated Values							
0	0.000	0.000	0.000	0.000	0.000	0.000	0.000
3	0.002	0.002	0.002	0.002	0.000	0.003	0.003
6	0.005	0.005	0.005	0.005	0.000	0.006	0.006
9	0.004	0.004	0.004	0.004	0.002	0.006	0.006
12	0.005	0.005	0.004	0.005	0.004	0.006	0.006
15	0.005	0.005	0.005	0.005	0.006	0.007	0.007
18	0.006	0.007	0.006	0.006	0.008	0.008	0.009
t-ratios of Estimated Values							
0							
3	-0.30	-0.30	-1.49	-1.49		-0.89	-0.89
6	-0.30	-0.30	-1.49	-1.49		-0.89	-0.89
9	-0.71	-0.76	-1.59	-1.33	-0.11	-0.66	-0.47
12	-1.06	-1.14	-1.50	-0.99	-0.11	-0.31	0.05
15	-1.26	-1.33	-1.27	-0.61	-0.11	0.01	0.47
18	-1.33	-1.40	-1.03	-0.30	-0.11	0.23	0.74

© 2024 Quanbeck A et al. *JAMA Network Open*.

Covariance Parameter Estimates						
Cov Parm	Subject	Estimate		Std Dev & Correlation		
Intercept	Clinic	0.0001	Clinic	0.0097		
UN(1,1)	PCP	0.0022				
UN(2,1)	PCP	(0.0001)				
UN(2,2)	PCP	0.0000	PCP	0.0469		
UN(3,1)	PCP	0.0001		-36.9%	0.0035	
UN(3,2)	PCP	(0.0000)		40.5%	-64.5%	0.0045
UN(3,3)	PCP	0.0000				
Residual		0.0564	Residual	0.2375		

Solution for Fixed Effects						
Effect	system	Estimate	Standard Error	DF	t Value	Pr > |t|
system	Bellin	0.0771	0.0066	866	11.68	<.0001
system	UW Health	0.1036	0.0057	866	18.06	<.0001
clinic_size		0.0000	0.0000	866	Infty	<.0001
t7		-0.0002	0.0008	263	-0.30	0.766
PF_t7		-0.0009	0.0011	866	-0.89	0.373
t13		-0.0003	0.0011	214	-0.28	0.780
PF_t13		0.0016	0.0015	866	1.03	0.305
PPC_t13		-0.0001	0.0007	866	-0.11	0.914
PFandPPC_t13		0.0004	0.0010	866	0.45	0.652

© 2024 Quanbeck A et al. *JAMA Network Open*.

Estimates					
**Label**	**Estimate**	**Standard Error**	**DF**	**t Value**	**Pr > |t|**
**NOINT Chg**	-0.0082	0.0061	866	-1.34	0.181
**PF Only Chg**	-0.0062	0.0061	866	-1.03	0.305
**PPC Only Chg**	-0.0091	0.0065	866	-1.40	0.163
**PF & PPC Chg**	-0.0018	0.0063	866	-0.29	0.772
**PF Only vs NOINT**	0.0020	0.0084	866	0.23	0.816
**PPC Only vs NOINT**	-0.0009	0.0084	866	-0.11	0.914
**PF Only vs PPC Only 1**	0.0029	0.0087	866	0.33	0.742
**PF Only vs PPC Only 2**	0.0085	0.0092	866	0.92	0.358
**PF & PPC Interaction**	0.0053	0.0118	866	0.45	0.652
**PF & PPC vs NOINT**	0.0063	0.0086	866	0.74	0.461

© 2024 Quanbeck A et al. *JAMA Network Open*.

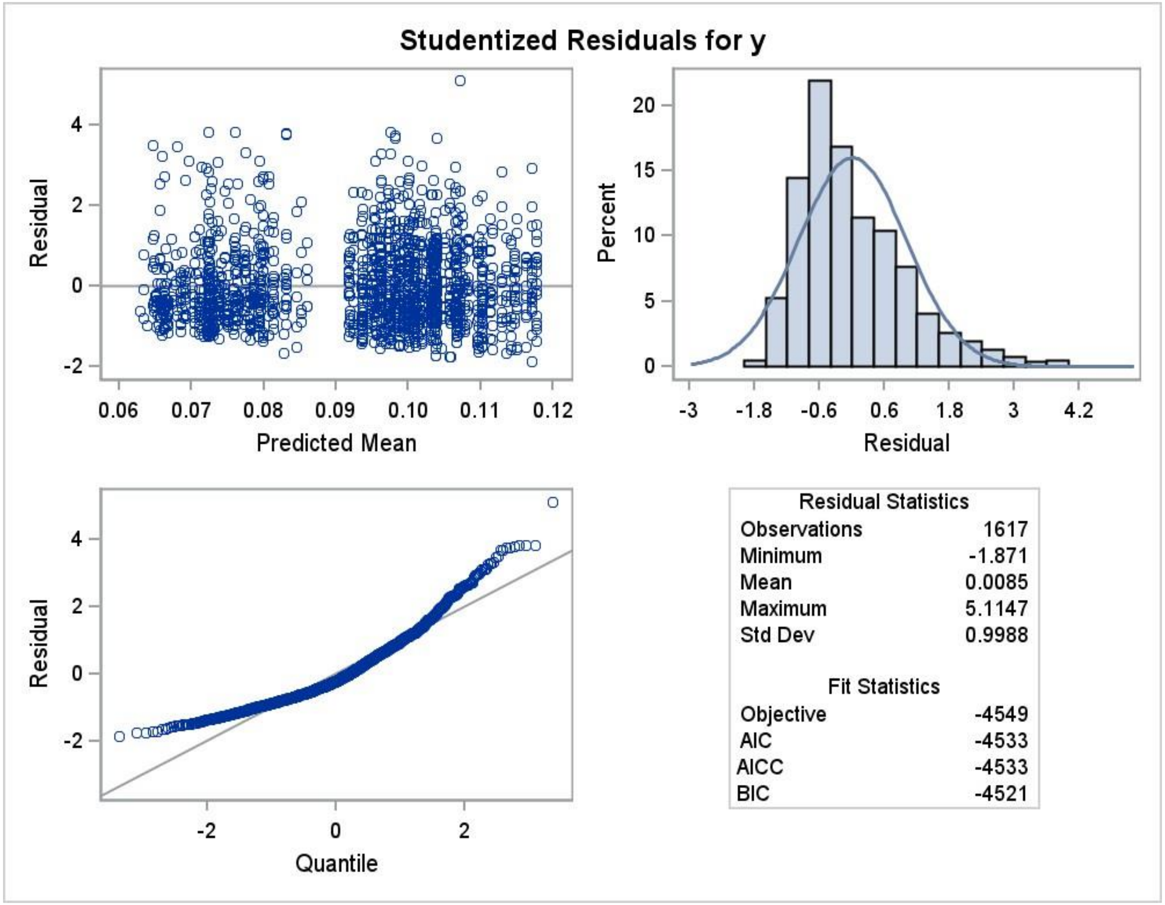

© 2024 Quanbeck A et al. *JAMA Network Open*.

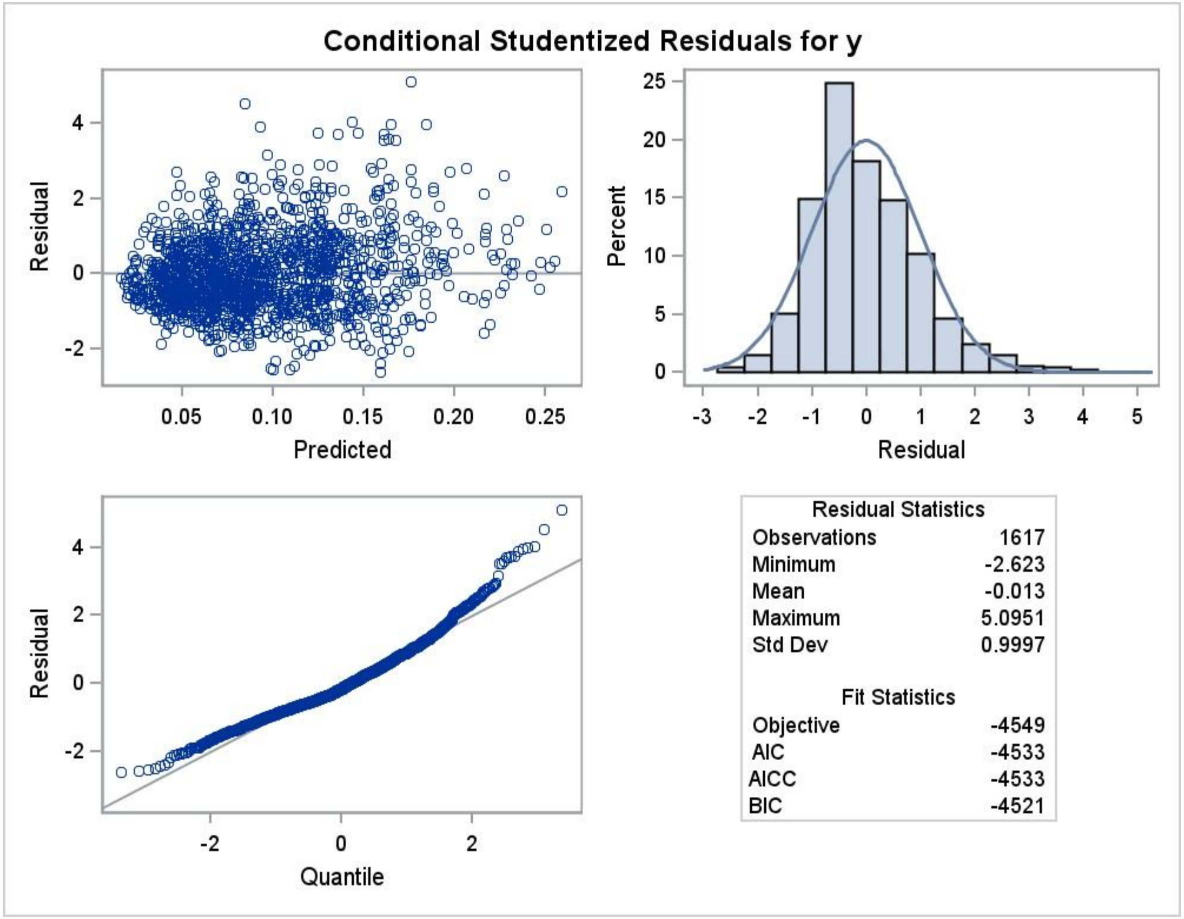

Appendix B4 – Percent of Opioid Patients with Urine Drug Screen in Last 12 Months (All opioid Patients)

© 2024 Quanbeck A et al. *JAMA Network Open*.

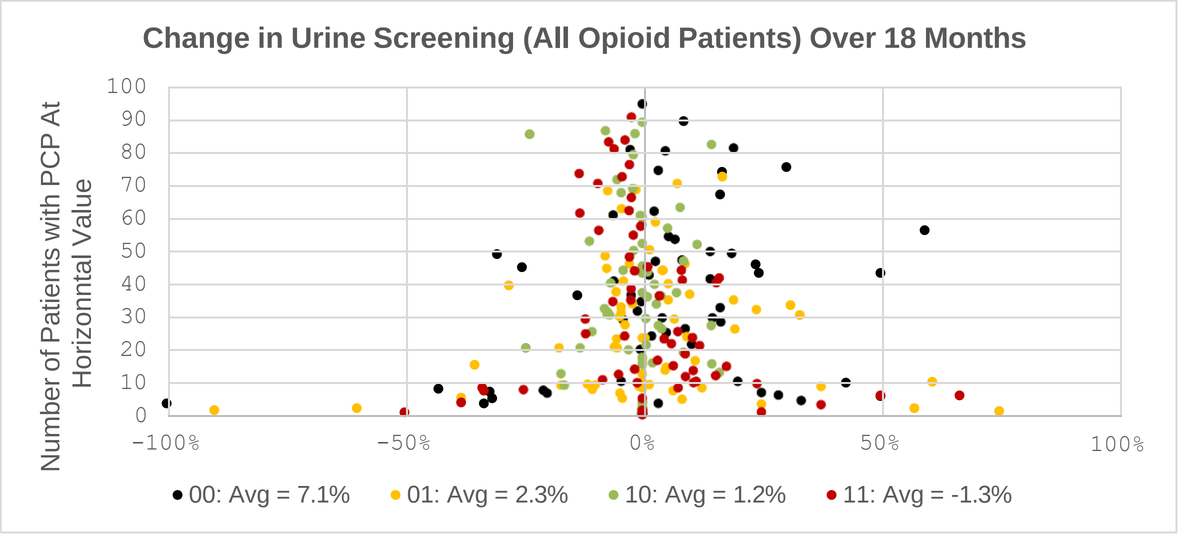

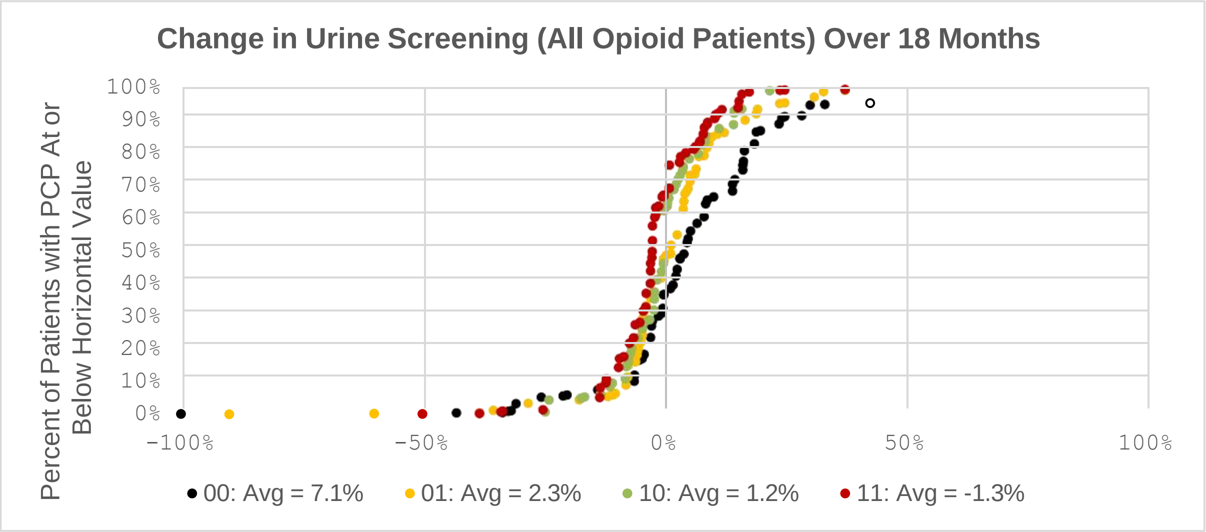

© 2024 Quanbeck A et al. *JAMA Network Open*.

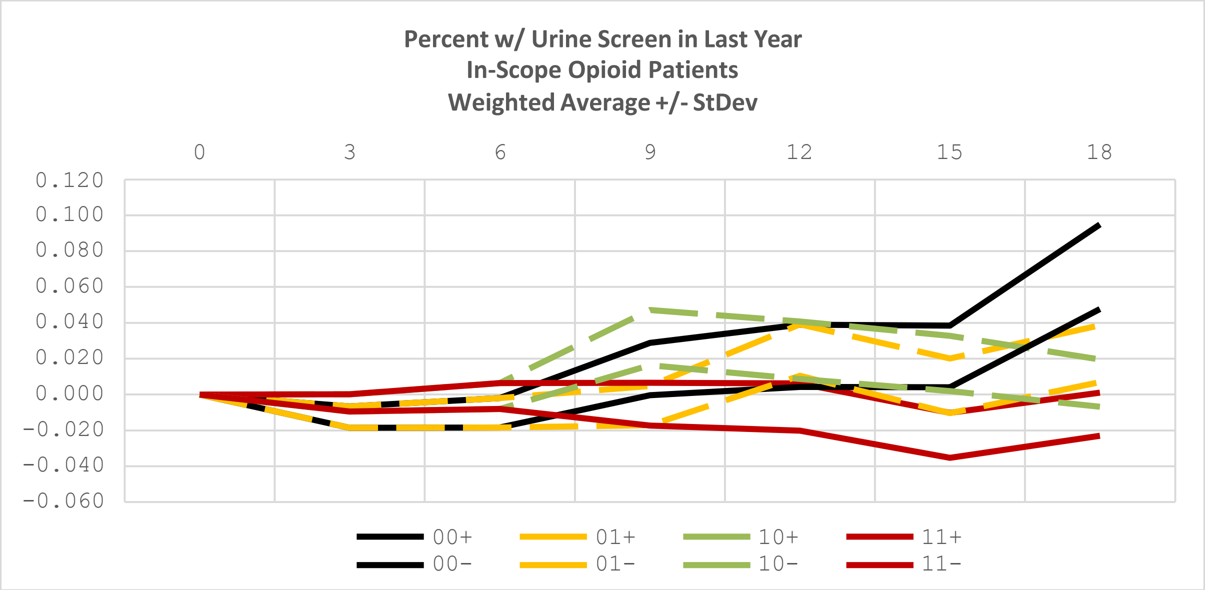

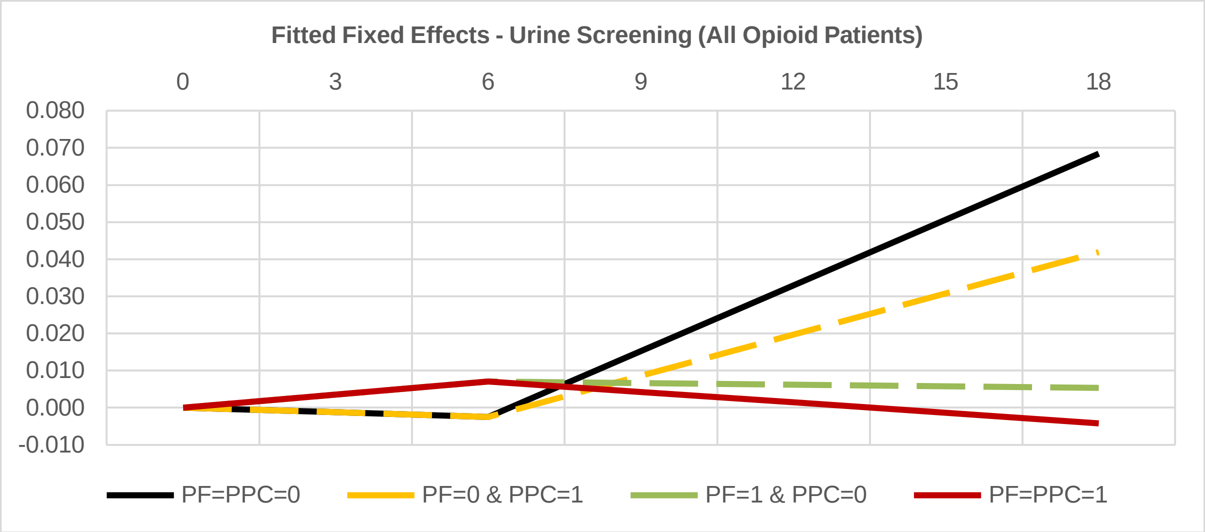

© 2024 Quanbeck A et al. *JAMA Network Open*.

Urine Screening (All Opioid Patients)							
Study Month	Estimated Fixed Effect Curves				Differences		
	PF=PPC=0	PF=0 & PPC=1	PF=1 & PPC=0	PF=PPC=1	PF=0 & PPC=1 minus PF=PPC=0	PF=1 & PPC=0 minus PF=PPC=0	PF=PPC=1 minus PF=PPC=0
0	0.000	0.000	0.000	0.000	0.000	0.000	0.000
3	-0.001	-0.001	0.004	0.004	0.000	0.005	0.005
6	-0.002	-0.002	0.007	0.007	0.000	0.010	0.010
9	0.015	0.009	0.007	0.004	-0.007	-0.009	-0.011
12	0.033	0.020	0.006	0.001	-0.013	-0.027	-0.032
15	0.051	0.031	0.006	-0.001	-0.020	-0.045	-0.052
18	0.068	0.042	0.005	-0.004	-0.027	-0.063	-0.073
Std Dev of Estimated Values							
0	0.000	0.000	0.000	0.000	0.000	0.000	0.000
3	0.004	0.004	0.004	0.004	0.000	0.006	0.006
6	0.009	0.009	0.009	0.009	0.000	0.012	0.012
9	0.009	0.009	0.009	0.009	0.005	0.012	0.012
12	0.010	0.010	0.010	0.010	0.010	0.014	0.014
15	0.013	0.012	0.013	0.013	0.015	0.018	0.018
18	0.016	0.015	0.015	0.015	0.020	0.022	0.022
t-ratios of Estimated Values							
0							
3	-0.29	-0.29	0.83	0.83		0.80	0.80
6	-0.29	-0.29	0.83	0.83		0.80	0.80
9	1.74	0.99	0.76	0.49	-1.33	-0.71	-0.91
12	3.21	1.95	0.61	0.14	-1.33	-1.87	-2.21
15	4.00	2.48	0.46	-0.11	-1.33	-2.54	-2.95
18	4.41	2.75	0.35	-0.27	-1.33	-2.91	-3.35

© 2024 Quanbeck A et al. *JAMA Network Open*.

Covariance Parameter Estimates						
Cov Parm	Subject	Estimate		Std Dev & Correlation		
Intercept	Clinic	0.0049	Clinic	0.0697		
UN(1,1)	PCP	0.0113				
UN(2,1)	PCP	(0.0005)				
UN(2,2)	PCP	0.0001	PCP	0.1063		
UN(3,1)	PCP	0.0003		-44.4%	0.0108	
UN(3,2)	PCP	(0.0001)		24.3%	-85.6%	0.0130
UN(3,3)	PCP	0.0002				
Residual		0.0817	Residual	0.2858		

Solution for Fixed Effects						
Effect	system	Estimate	Standard Error	DF	t Value	Pr > |t|
system	Bellin	0.1134	0.0221	866	5.14	<.0001
system	UW Health	0.1854	0.0224	866	8.28	<.0001
clinic_size		0.0000	0.0000	866	-1.59	0.113
t7		-0.0004	0.0014	263	-0.28	0.776
PF_t7		0.0016	0.0020	866	0.80	0.426
t13		0.0063	0.0020	214	3.13	0.002
PF_t13		-0.0076	0.0028	866	-2.71	0.007
PPC_t13		-0.0022	0.0017	866	-1.33	0.183
PFandPPC_t13		0.0014	0.0024	866	0.60	0.546

© 2024 Quanbeck A et al. *JAMA Network Open*.

Estimates					
**Label**	**Estimate**	**Standard Error**	**DF**	**t Value**	**Pr > |t|**
**NOINT Chg**	0.0684	0.0155	866	4.41	<.0001
**PF Only Chg**	0.0053	0.0154	866	0.34	0.732
**PPC Only Chg**	0.0419	0.0152	866	2.75	0.006
**PF & PPC Chg**	-0.0042	0.0154	866	-0.27	0.784
**PF Only vs NOINT**	-0.0632	0.0217	866	-2.91	0.004
**PPC Only vs NOINT**	-0.0265	0.0199	866	-1.33	0.183
**PF Only vs PPC Only 1**	-0.0366	0.0215	866	-1.70	0.089
**PF Only vs PPC Only 2**	-0.0461	0.0202	866	-2.28	0.023
**PF & PPC Interaction**	0.0170	0.0282	866	0.60	0.546
**PF & PPC vs NOINT**	-0.0727	0.0217	866	-3.35	0.001

© 2024 Quanbeck A et al. *JAMA Network Open*.

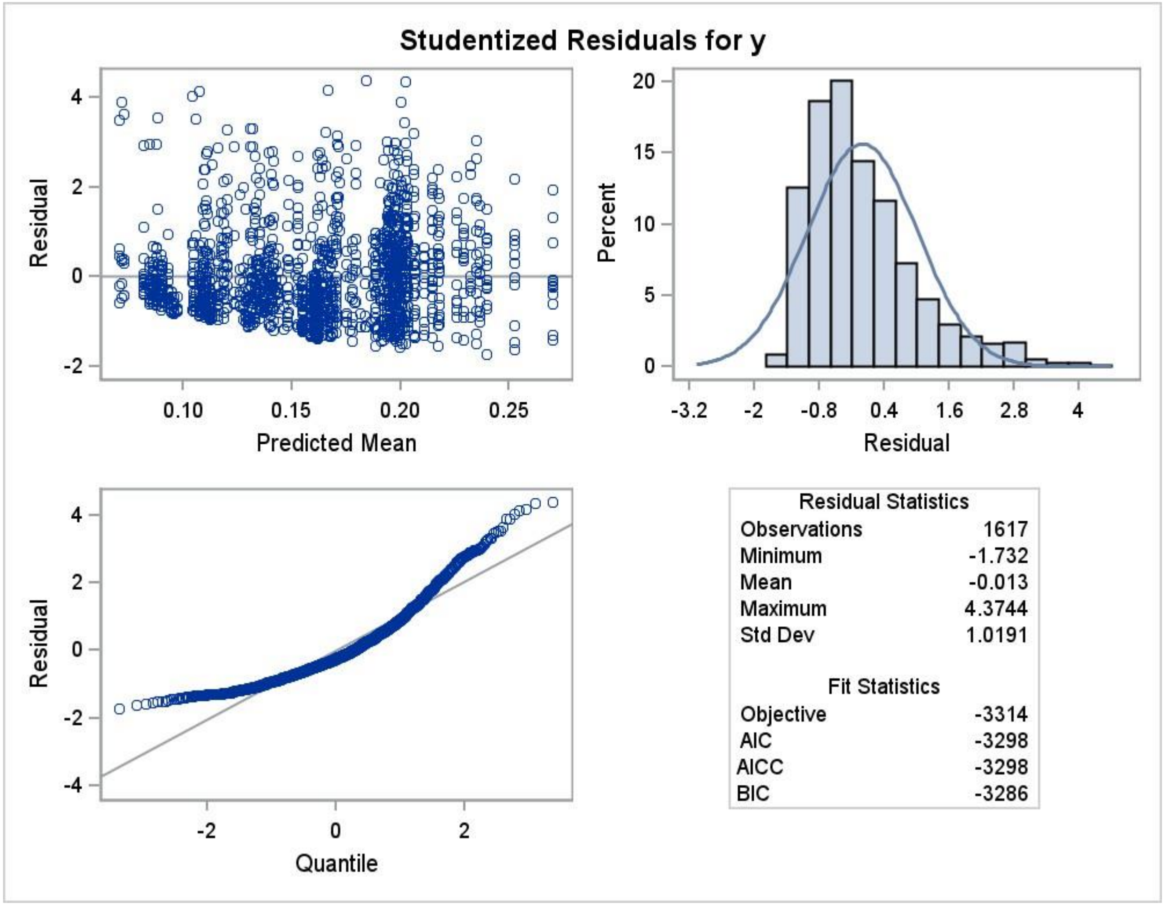

© 2024 Quanbeck A et al. *JAMA Network Open*.

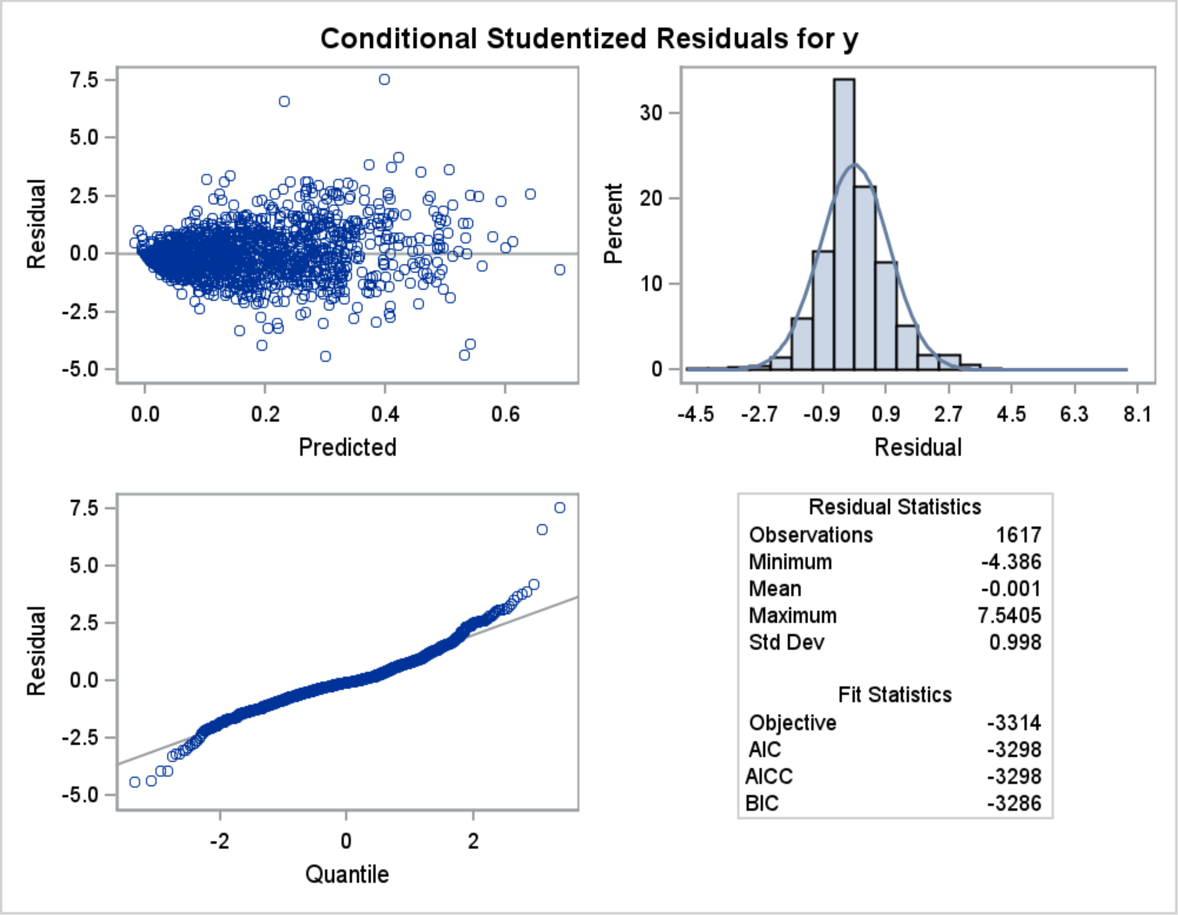

Appendix B5 – Percent of Opioid Patients with Treatment Agreement in Last 12 Months (All Opioid Patients)

© 2024 Quanbeck A et al. *JAMA Network Open*.

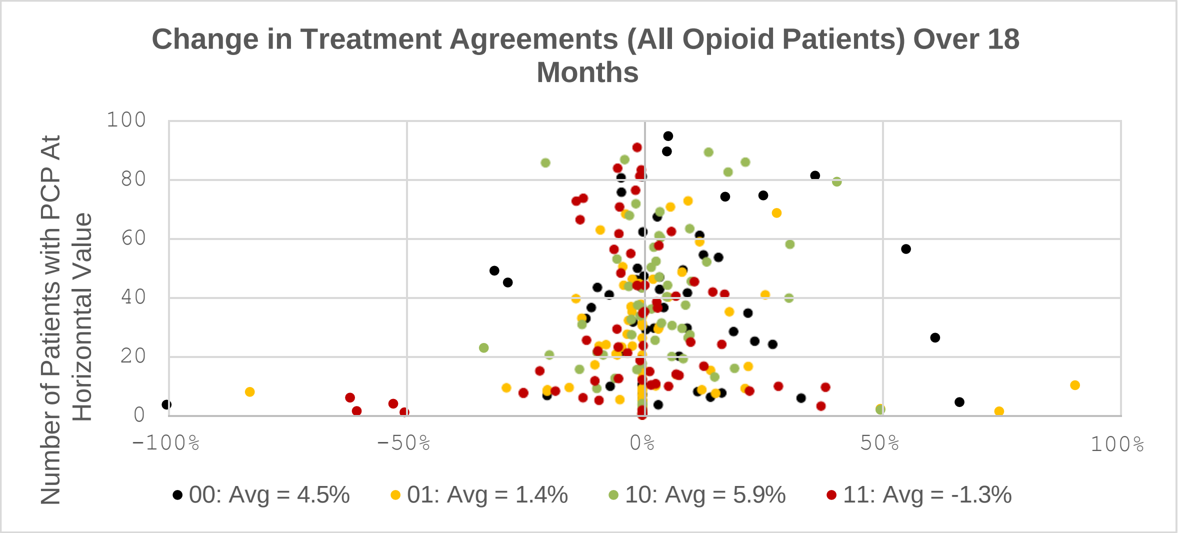

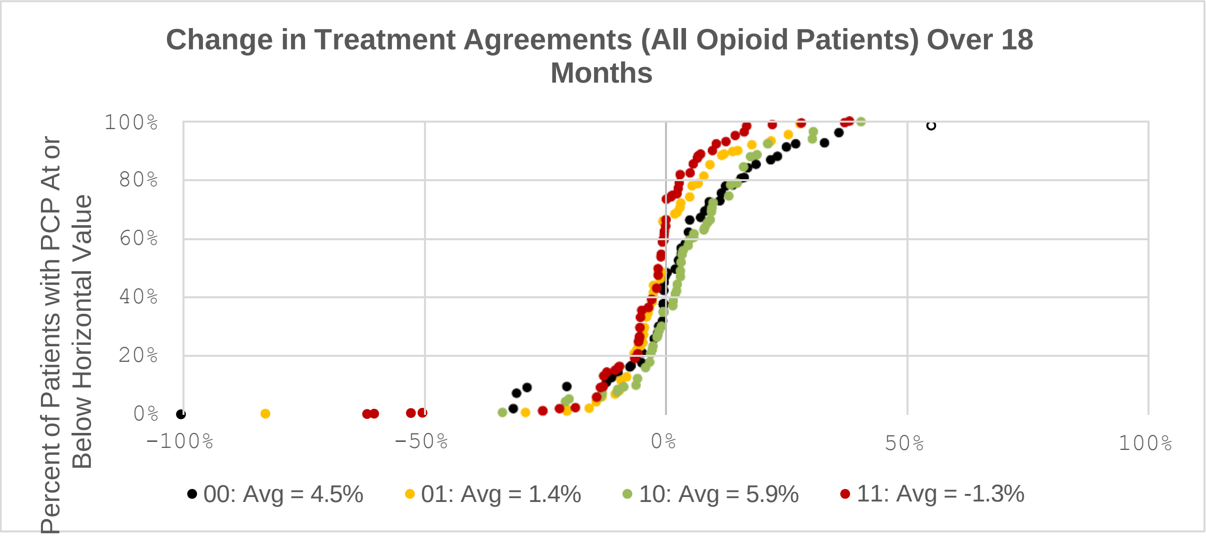

© 2024 Quanbeck A et al. *JAMA Network Open*.

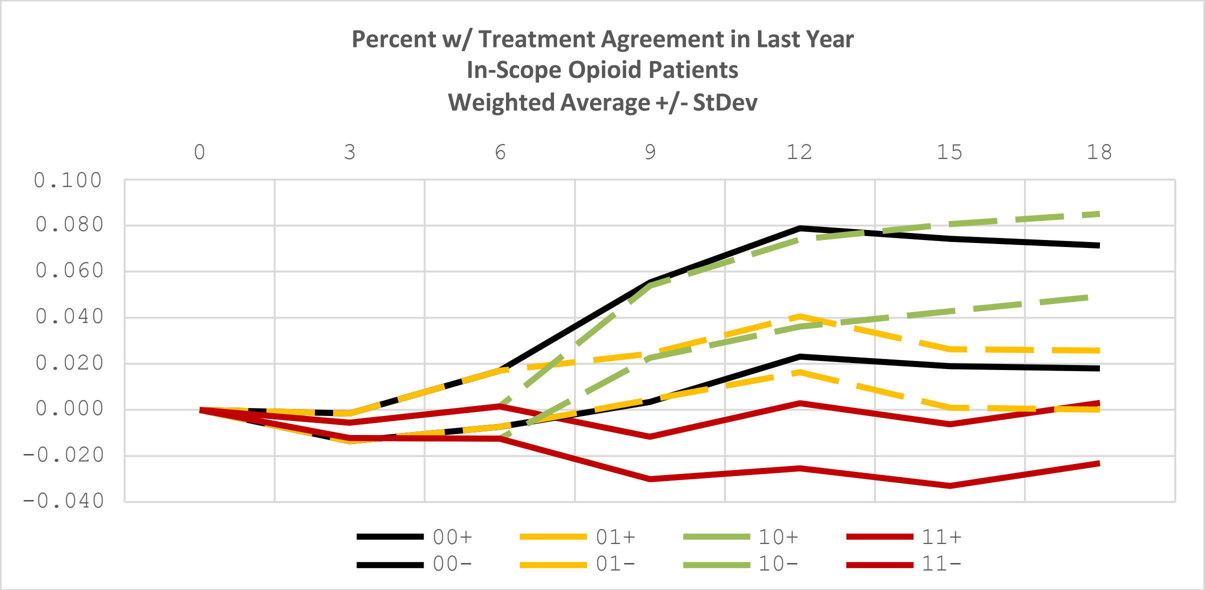

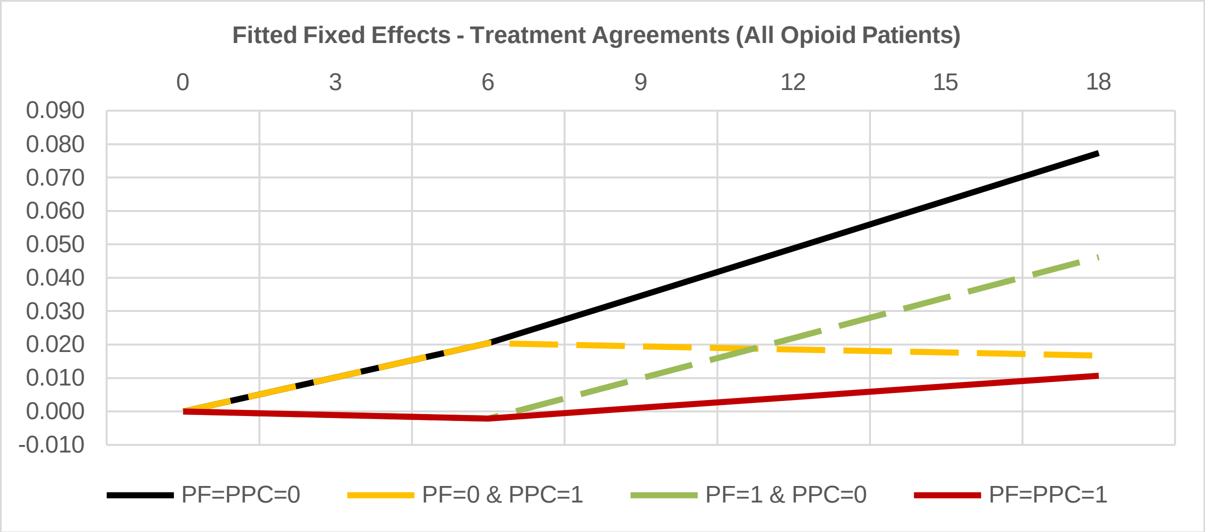

© 2024 Quanbeck A et al. *JAMA Network Open*.

Treatment Agreements (All Opioid Patients)							
Study Month	Estimated Fixed Effect Curves				Differences		
	PF=PPC=0	PF=0 & PPC=1	PF=1 & PPC=0	PF=PPC=1	PF=0 & PPC=1 minus PF=PPC=0	PF=1 & PPC=0 minus PF=PPC=0	PF=PPC=1 minus PF=PPC=0
0	0.000	0.000	0.000	0.000	0.000	0.000	0.000
3	0.010	0.010	-0.001	-0.001	0.000	-0.011	-0.011
6	0.020	0.020	-0.002	-0.002	0.000	-0.023	-0.023
9	0.035	0.019	0.010	0.001	-0.015	-0.025	-0.034
12	0.049	0.019	0.022	0.004	-0.030	-0.027	-0.045
15	0.063	0.018	0.034	0.007	-0.045	-0.029	-0.056
18	0.077	0.017	0.046	0.011	-0.061	-0.031	-0.067
Std Dev of Estimated Values							
0	0.000	0.000	0.000	0.000	0.000	0.000	0.000
3	0.005	0.005	0.005	0.005	0.000	0.007	0.007
6	0.010	0.010	0.010	0.010	0.000	0.014	0.014
9	0.010	0.010	0.010	0.010	0.005	0.014	0.014
12	0.012	0.012	0.012	0.012	0.010	0.016	0.016
15	0.014	0.014	0.014	0.014	0.015	0.019	0.019
18	0.016	0.016	0.016	0.016	0.019	0.022	0.022
t-ratios of Estimated Values							
0							
3	2.00	2.00	-0.21	-0.21		-1.63	-1.63
6	2.00	2.00	-0.21	-0.21		-1.63	-1.63
9	3.32	1.88	0.95	0.10	-3.13	-1.75	-2.39
12	4.16	1.61	1.87	0.37	-3.13	-1.69	-2.81
15	4.57	1.30	2.46	0.55	-3.13	-1.54	-2.96
18	4.73	1.04	2.82	0.66	-3.13	-1.39	-2.97

© 2024 Quanbeck A et al. *JAMA Network Open*.

Covariance Parameter Estimates						
**Cov Parm**	**Subject**	**Estimate**		**Std Dev & Correlation**		
Intercept	Clinic	0.0016	Clinic	0.0403		
UN(1,1)	PCP	0.0073				
UN(2,1)	PCP	(0.0008)				
UN(2,2)	PCP	0.0003	PCP	0.0857		
UN(3,1)	PCP	0.0006		-56.8%	0.0160	
UN(3,2)	PCP	(0.0003)		41.5%	-90.8%	0.0172
UN(3,3)	PCP	0.0003				
Residual		0.0623	Residual	0.2496		

Solution for Fixed Effects						
**Effect**	**system**	**Estimate**	**Standard Error**	**DF**	**t Value**	**Pr > |t|**
system	Bellin	0.1405	0.0144	866	9.77	<.0001
system	UW Health	0.0631	0.0141	866	4.47	<.0001
clinic_size		0.0000	0.0000	866	-0.39	0.697
t7		0.0034	0.0017	263	2.00	0.047
PF_t7		-0.0038	0.0023	866	-1.63	0.104
t13		0.0013	0.0022	214	0.62	0.533
PF_t13		0.0030	0.0030	866	1.02	0.308
PPC_t13		-0.0051	0.0016	866	-3.13	0.002
PFandPPC_t13		0.0021	0.0023	866	0.92	0.358

© 2024 Quanbeck A et al. *JAMA Network Open*.

Estimates					
**Label**	**Estimate**	**Standard Error**	**DF**	**t Value**	**Pr > |t|**
**NOINT Chg**	0.0773	0.0164	866	4.72	<.0001
**PF Only Chg**	0.0462	0.0164	866	2.82	0.005
**PPC Only Chg**	0.0168	0.0161	866	1.04	0.297
**PF & PPC Chg**	0.0107	0.0162	866	0.66	0.509
**PF Only vs NOINT**	-0.0312	0.0225	866	-1.38	0.167
**PPC Only vs NOINT**	-0.0606	0.0194	866	-3.13	0.002
**PF Only vs PPC Only 1**	0.0294	0.0223	866	1.32	0.188
**PF Only vs PPC Only 2**	0.0520	0.0195	866	2.67	0.008
**PF & PPC Interaction**	0.0252	0.0274	866	0.92	0.358
**PF & PPC vs NOINT**	-0.0666	0.0225	866	-2.97	0.003

© 2024 Quanbeck A et al. *JAMA Network Open*.

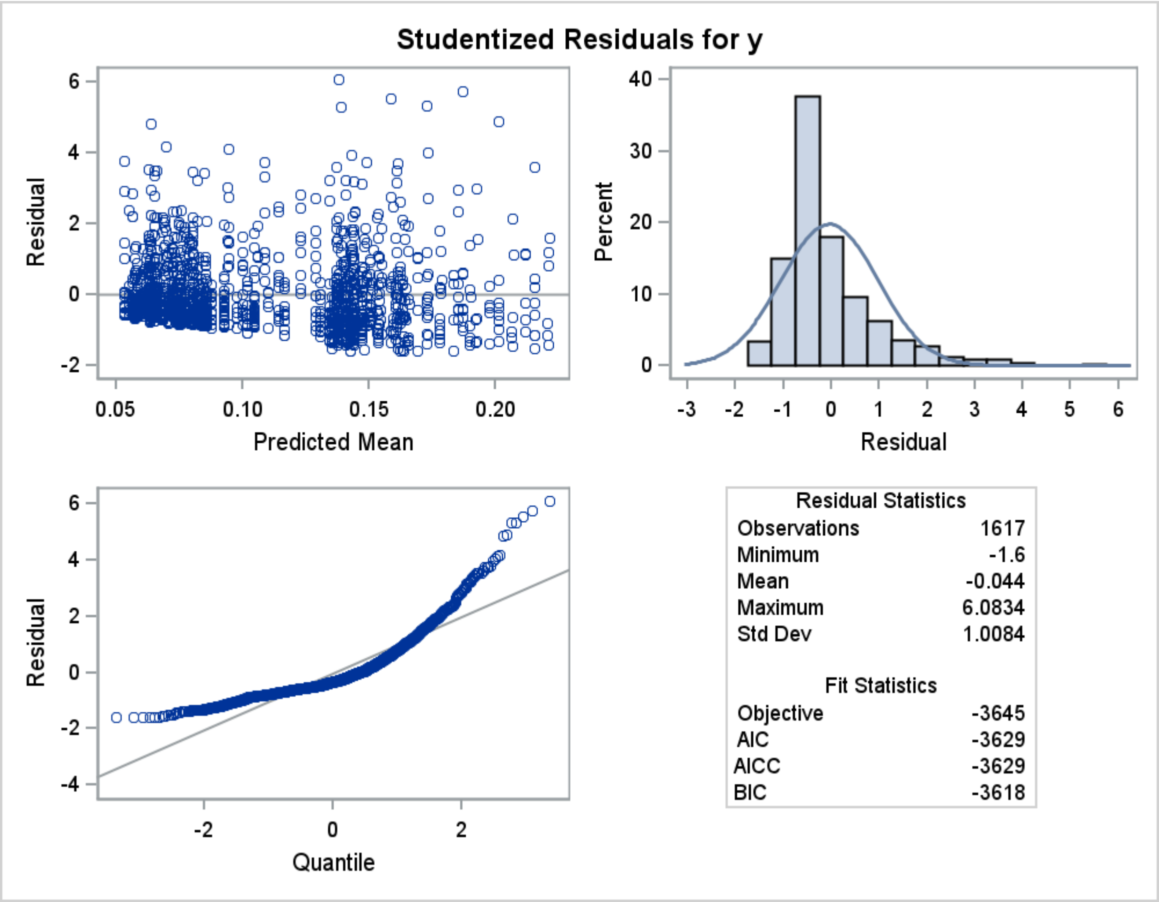

© 2024 Quanbeck A et al. *JAMA Network Open*.

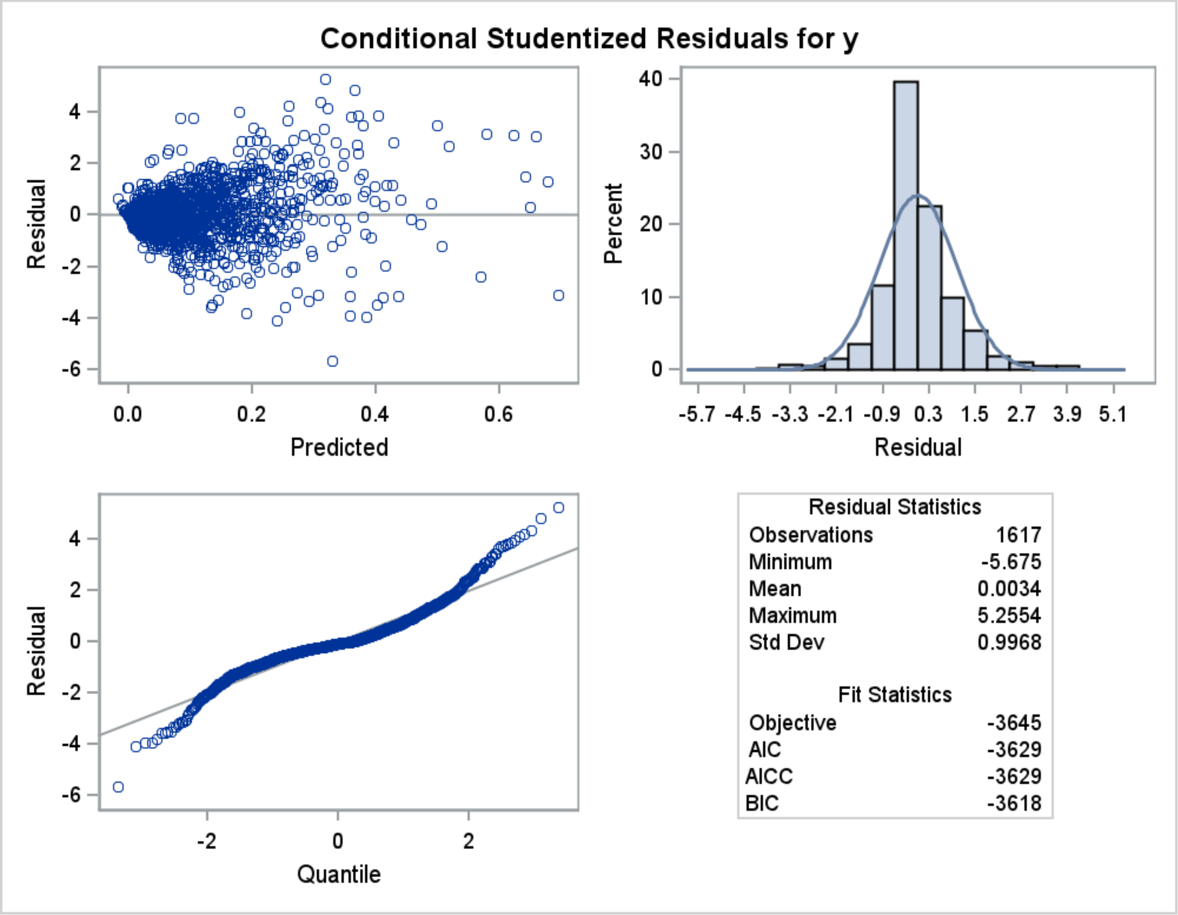

Appendix B6 – Percent of Opioid Patients with PHQ-9 Depression Screen in Last 12 Months (All Opioid Patients)

© 2024 Quanbeck A et al. *JAMA Network Open*.

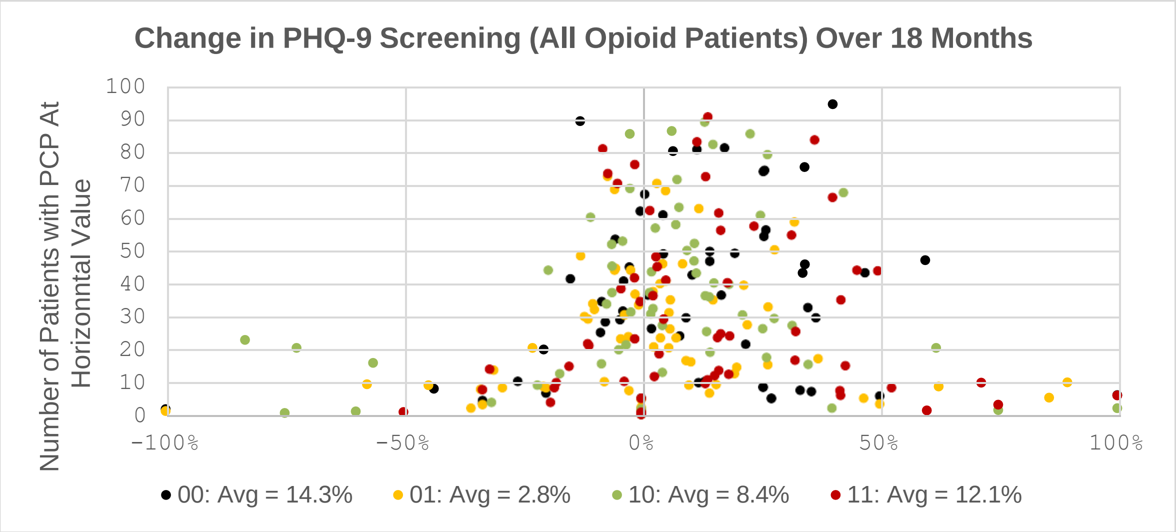

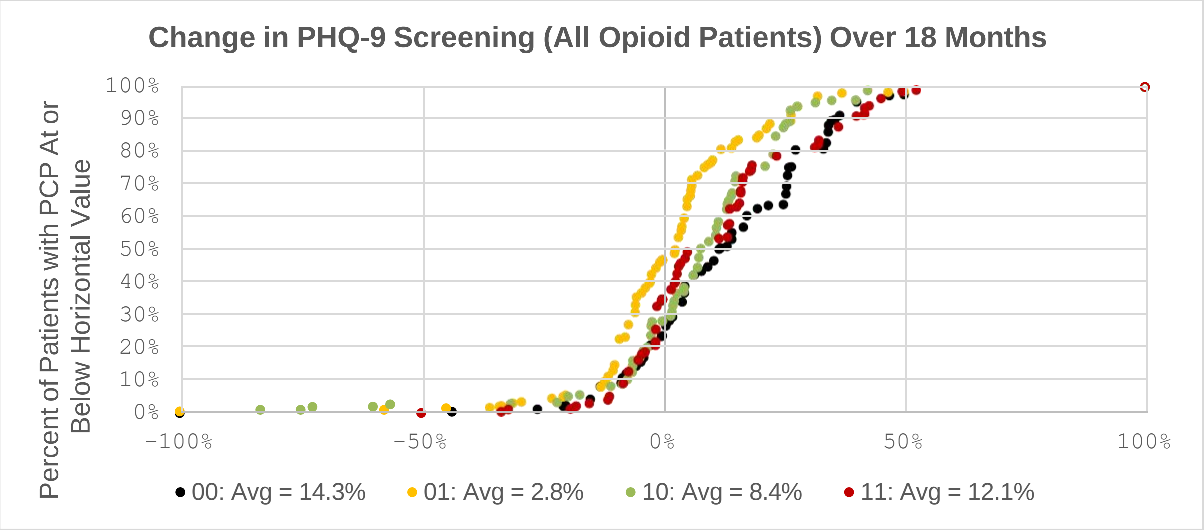

© 2024 Quanbeck A et al. *JAMA Network Open*.

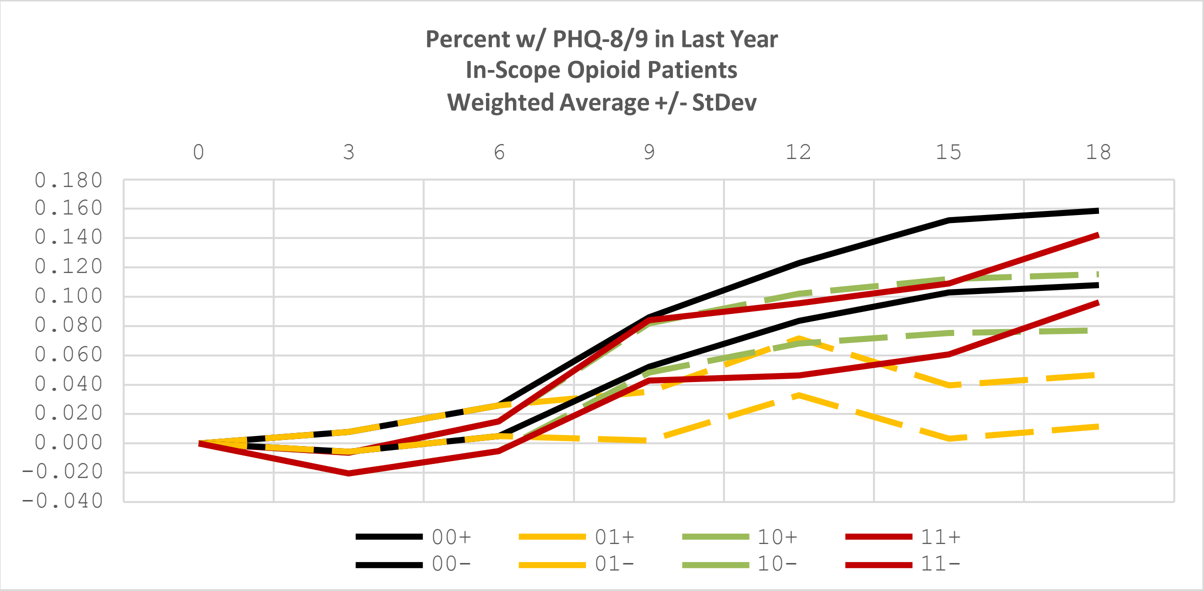

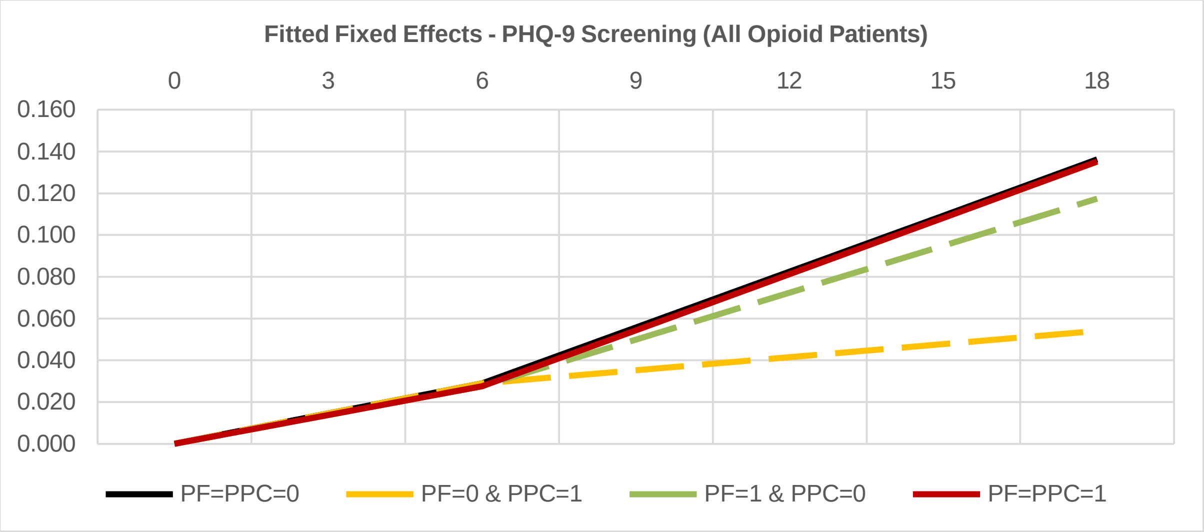

© 2024 Quanbeck A et al. *JAMA Network Open*.

PHQ-9 Screening (All Opioid Patients)							
Study Month	Estimated Fixed Effect Curves				Differences		
	PF=PPC=0	PF=0 & PPC=1	PF=1 & PPC=0	PF=PPC=1	PF=0 & PPC=1 minus PF=PPC=0	PF=1 & PPC=0 minus PF=PPC=0	PF=PPC=1 minus PF=PPC=0
0	0.000	0.000	0.000	0.000	0.000	0.000	0.000
3	0.015	0.015	0.014	0.014	0.000	-0.001	-0.001
6	0.029	0.029	0.028	0.028	0.000	-0.002	-0.002
9	0.056	0.035	0.050	0.054	-0.021	-0.006	-0.001
12	0.083	0.042	0.072	0.081	-0.041	-0.010	-0.001
15	0.109	0.048	0.095	0.108	-0.062	-0.014	-0.001
18	0.136	0.054	0.117	0.135	-0.082	-0.019	-0.001
Std Dev of Estimated Values							
0	0.000	0.000	0.000	0.000	0.000	0.000	0.000
3	0.006	0.006	0.006	0.006	0.000	0.008	0.008
6	0.011	0.011	0.011	0.011	0.000	0.016	0.016
9	0.012	0.012	0.012	0.012	0.006	0.017	0.017
12	0.015	0.014	0.014	0.014	0.013	0.020	0.020
15	0.018	0.017	0.017	0.017	0.019	0.024	0.024
18	0.021	0.021	0.020	0.020	0.026	0.029	0.029
t-ratios of Estimated Values							
0							
3	2.56	2.56	2.44	2.44		-0.10	-0.10
6	2.56	2.56	2.44	2.44		-0.10	-0.10
9	4.56	2.94	4.16	4.57	-3.20	-0.35	-0.08
12	5.68	2.93	5.18	5.88	-3.20	-0.51	-0.06
15	6.17	2.76	5.67	6.53	-3.20	-0.60	-0.05
18	6.36	2.58	5.89	6.82	-3.20	-0.65	-0.03

© 2024 Quanbeck A et al. *JAMA Network Open*.

Covariance Parameter Estimates				Std Dev & Correlation		
Cov Parm	Subject	Estimate				
Intercept	Clinic	0.0066	Clinic	0.0812		
UN(1,1)	PCP	0.0132				
UN(2,1)	PCP	(0.0008)				
UN(2,2)	PCP	0.0002	PCP	0.1150		
UN(3,1)	PCP	0.0003		-46.7%	0.0147	
UN(3,2)	PCP	(0.0002)		17.9%	-82.8%	0.0140
UN(3,3)	PCP	0.0002				
Residual		0.1375	Residual	0.3708		

Solution for Fixed Effects

Effect	system	Estimate	Standard Error	DF	t Value	Pr > |t|
system	Bellin	0.3037	0.0251	866	12.11	<.0001
system	UW Health	0.2204	0.0258	866	8.54	<.0001
clinic_size		0.0000	0.0000	866	-0.37	0.711
t7		0.0048	0.0019	263	2.56	0.011
PF_t7		-0.0003	0.0026	866	-0.09	0.925
t13		0.0041	0.0024	214	1.70	0.091
PF_t13		-0.0012	0.0034	866	-0.35	0.725
PPC_t13		-0.0068	0.0021	866	-3.20	0.001
PFandPPC_t13		0.0083	0.0030	866	2.76	0.006

© 2024 Quanbeck A et al. *JAMA Network Open*.

Estimates					
Label	Estimate	Standard Error	DF	t Value	Pr > |t|
NOINT Chg	0.1362	0.0215	866	6.35	<.0001
PF Only Chg	0.1175	0.0214	866	5.50	<.0001
PPC Only Chg	0.0541	0.0210	866	2.57	0.010
PF & PPC Chg	0.1353	0.0213	866	6.36	<.0001
PF Only vs NOINT	-0.0187	0.0298	866	-0.63	0.530
PPC Only vs NOINT	-0.0821	0.0256	866	-3.20	0.001
PF Only vs PPC Only 1	0.0634	0.0295	866	2.15	0.032
PF Only vs PPC Only 2	0.0648	0.0255	866	2.54	0.011
PF & PPC Interaction	0.0999	0.0362	866	2.76	0.006
PF & PPC vs NOINT	-0.0009	0.0298	866	-0.03	0.975

© 2024 Quanbeck A et al. *JAMA Network Open*.

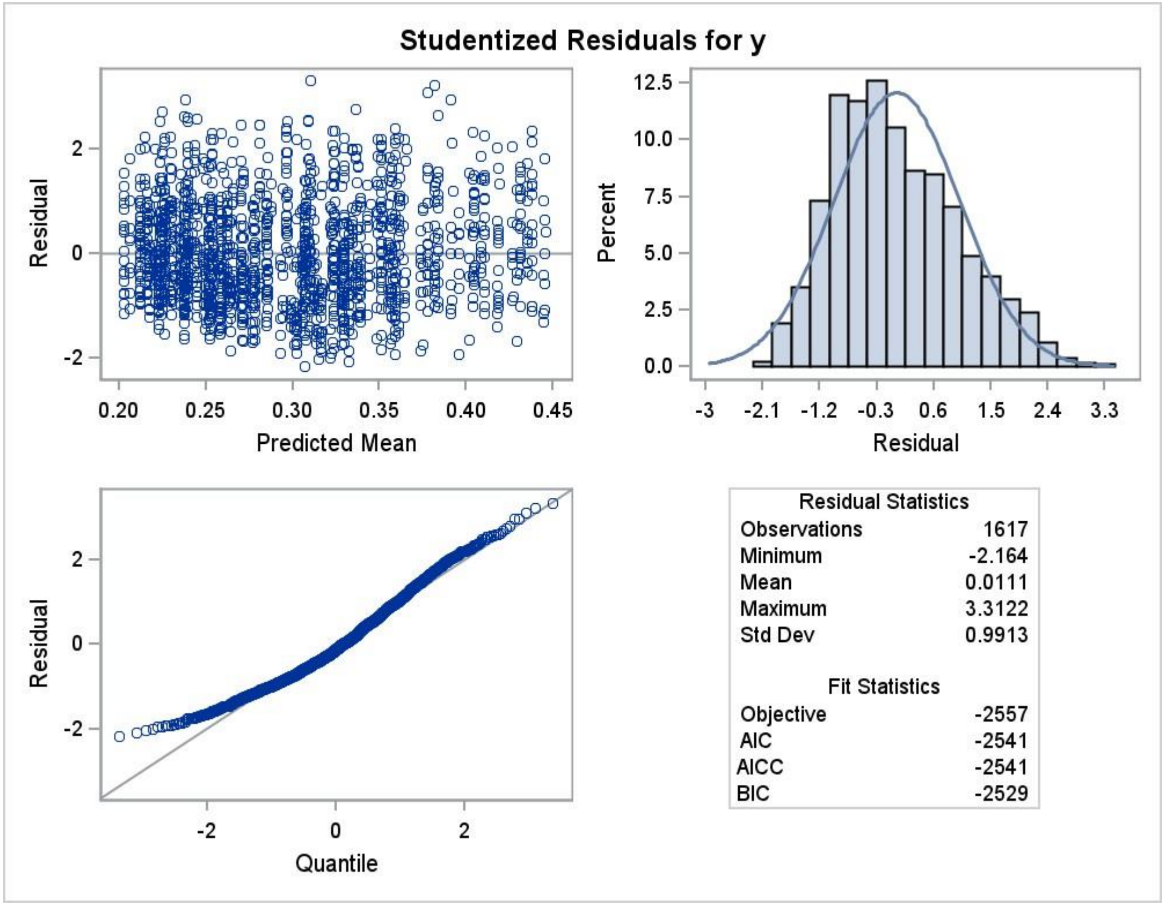

© 2024 Quanbeck A et al. *JAMA Network Open*.

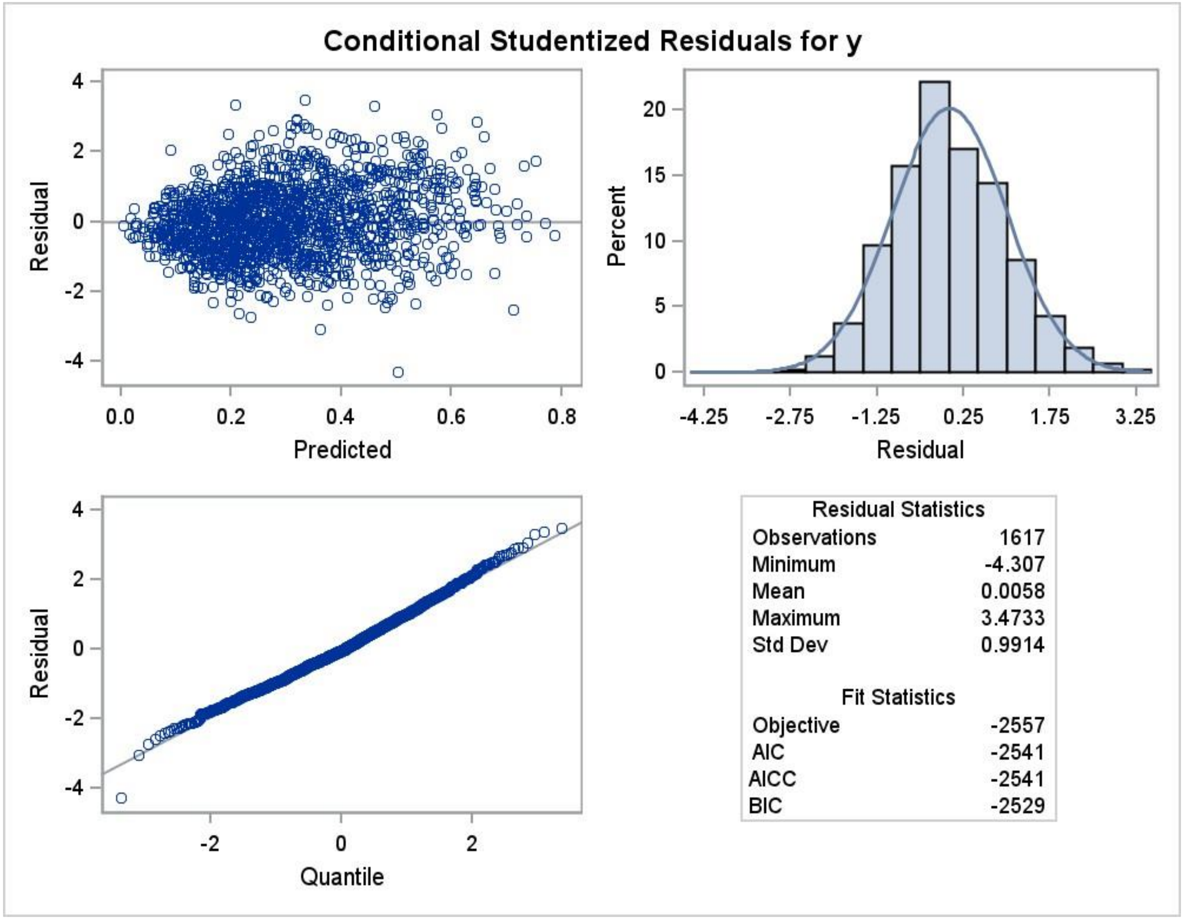

Appendix B7 – Percent of Opioid Patients with PEG-3 Pain Screen in Last 12 Months (All Opioid Patients)

© 2024 Quanbeck A et al. *JAMA Network Open*.

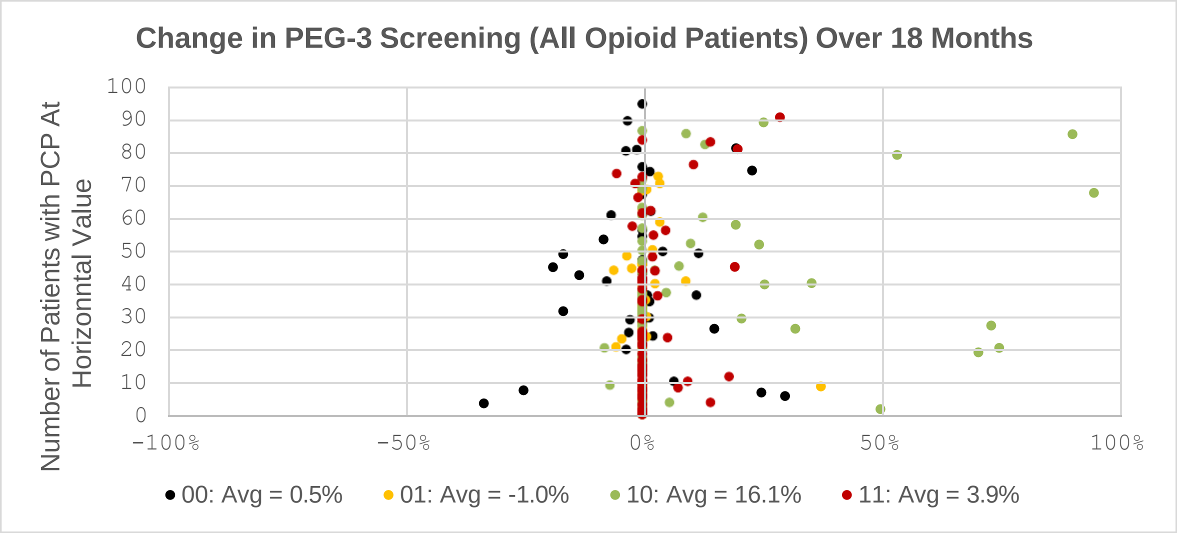

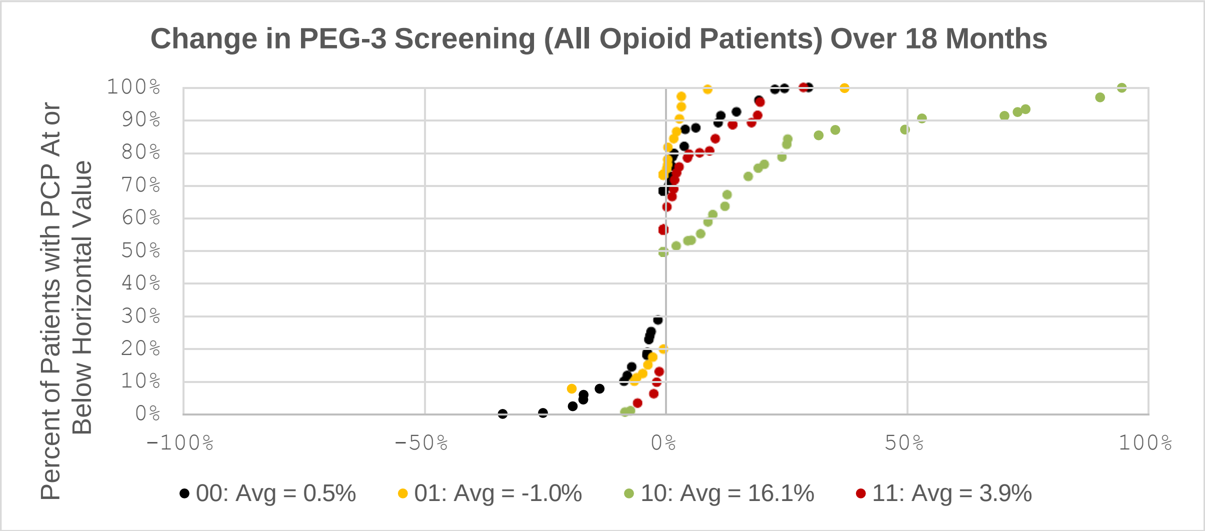

© 2024 Quanbeck A et al. *JAMA Network Open*.

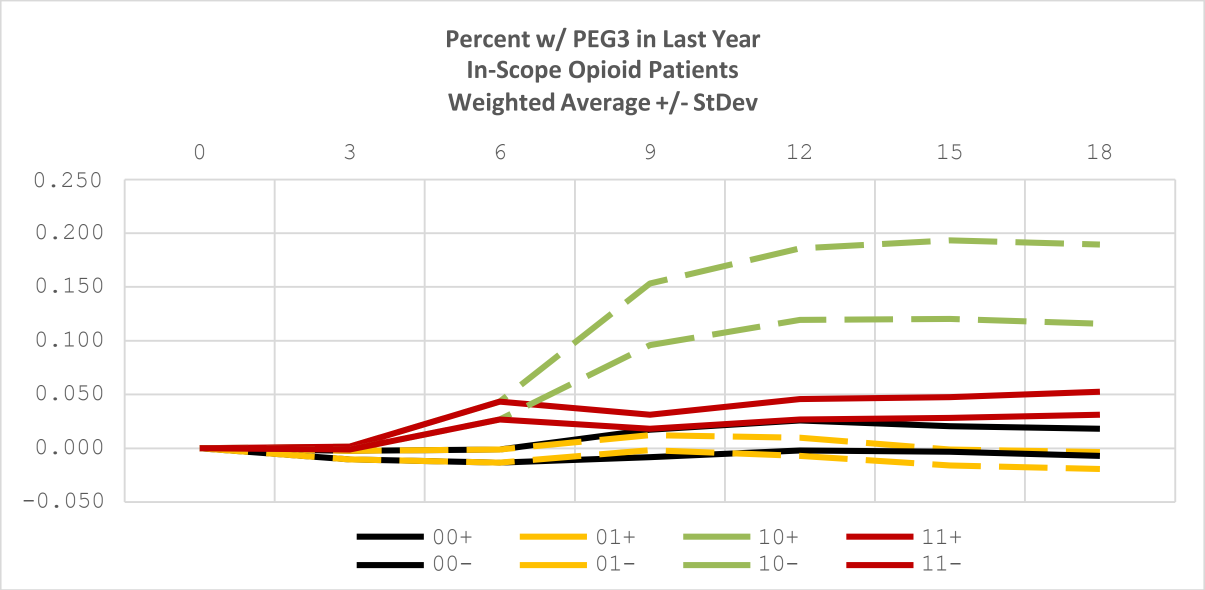

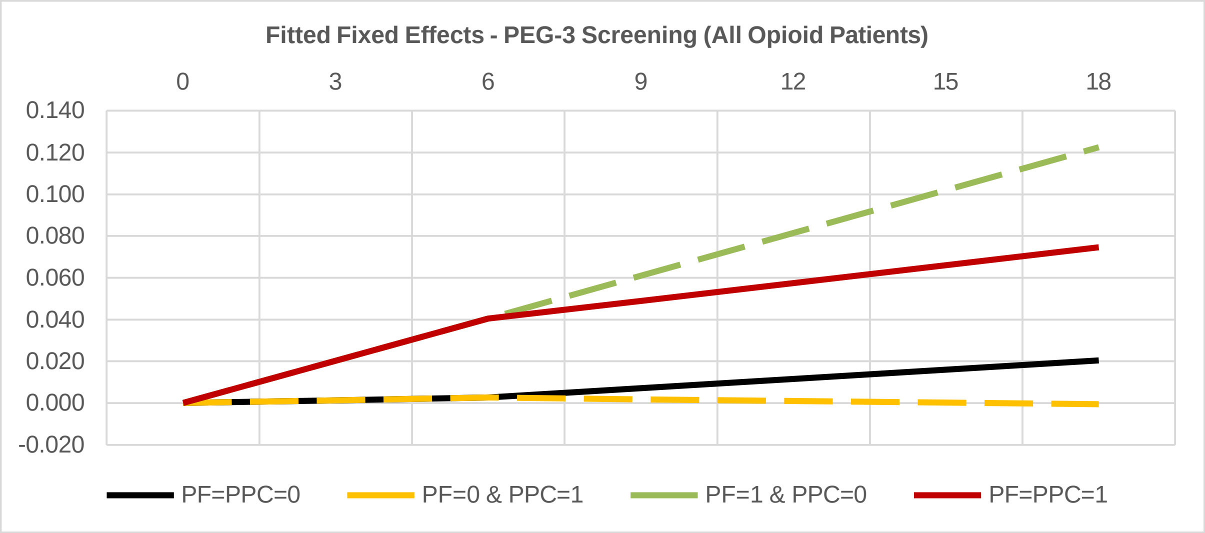

© 2024 Quanbeck A et al. *JAMA Network Open*.

PEG-3 Screening (All Opioid Patients)							
Study Month	Estimated Fixed Effect Curves				Differences		
	PF=PPC=0	PF=0 & PPC=1	PF=1 & PPC=0	PF=PPC=1	PF=0 & PPC=1 minus PF=PPC=0	PF=1 & PPC=0 minus PF=PPC=0	PF=PPC=1 minus PF=PPC=0
0	0.000	0.000	0.000	0.000	0.000	0.000	0.000
3	0.001	0.001	0.020	0.020	0.000	0.019	0.019
6	0.003	0.003	0.040	0.040	0.000	0.038	0.038
9	0.007	0.002	0.061	0.049	-0.005	0.054	0.042
12	0.011	0.001	0.081	0.057	-0.010	0.070	0.046
15	0.016	0.000	0.102	0.066	-0.016	0.086	0.050
18	0.020	-0.001	0.123	0.075	-0.021	0.102	0.054
Std Dev of Estimated Values							
0	0.000	0.000	0.000	0.000	0.000	0.000	0.000
3	0.004	0.004	0.004	0.004	0.000	0.006	0.006
6	0.009	0.009	0.009	0.009	0.000	0.012	0.012
9	0.010	0.010	0.010	0.010	0.005	0.015	0.014
12	0.013	0.012	0.013	0.012	0.009	0.018	0.018
15	0.015	0.015	0.015	0.015	0.014	0.022	0.021
18	0.018	0.018	0.018	0.018	0.019	0.026	0.026
t-ratios of Estimated Values							
0							
3	0.29	0.29	4.53	4.53		3.03	3.03
6	0.29	0.29	4.53	4.53		3.03	3.03
9	0.68	0.18	5.85	4.75	-1.11	3.70	2.89
12	0.91	0.08	6.42	4.61	-1.11	3.94	2.61
15	1.04	0.02	6.63	4.37	-1.11	3.98	2.34
18	1.11	-0.03	6.69	4.13	-1.11	3.96	2.12

© 2024 Quanbeck A et al. *JAMA Network Open*.

Covariance Parameter Estimates						
**Cov Parm**	**Subject**	**Estimate**		**Std Dev & Correlation**		
**Intercept**	Clinic	0.0018	Clinic	0.0428		
**UN(1,1)**	PCP	0.0031				
**UN(2,1)**	PCP	(0.0002)				
**UN(2,2)**	PCP	0.0002	PCP	0.0560		
**UN(3,1)**	PCP	0.0001		-29.4%	0.0140	
**UN(3,2)**	PCP	(0.0001)		20.1%	-86.1%	0.0108
**UN(3,3)**	PCP	0.0001				
**Residual**		0.0440	Residual	0.2099		

Solution for Fixed Effects						
**Effect**	**system**	**Estimate**	**Standard Error**	**DF**	**t Value**	**Pr > |t|**
**system**	Bellin	0.0029	0.0135	866	0.21	0.8320
**system**	UW Health	0.0317	0.0137	866	2.31	0.0213
**clinic_size**		0.0000	0.0000	866	1.29	0.197
**t7**		0.0004	0.0015	263	0.29	0.770
**PF_t7**		0.0063	0.0021	866	3.03	0.003
**t13**		0.0010	0.0016	214	0.65	0.515
**PF_t13**		-0.0009	0.0023	866	-0.42	0.676
**PPC_t13**		-0.0017	0.0016	866	-1.11	0.267
**PFandPPC_t13**		-0.0023	0.0022	866	-1.02	0.308

© 2024 Quanbeck A et al. *JAMA Network Open*.

Estimates					
**Label**	**Estimate**	**Standard Error**	**DF**	**t Value**	**Pr > |t|**
**NOINT Chg**	0.0204	0.0184	866	1.11	0.268
**PF Only Chg**	0.1225	0.0184	866	6.67	<.0001
**PPC Only Chg**	-0.0006	0.0180	866	-0.03	0.975
**PF & PPC Chg**	0.0745	0.0181	866	4.12	<.0001
**PF Only vs NOINT**	0.1022	0.0258	866	3.95	<.0001
**PPC Only vs NOINT**	-0.0209	0.0189	866	-1.11	0.267
**PF Only vs PPC Only 1**	0.1231	0.0256	866	4.81	<.0001
**PF Only vs PPC Only 2**	0.0853	0.0194	866	4.39	<.0001
**PF & PPC Interaction**	-0.0271	0.0266	866	-1.02	0.308
**PF & PPC vs NOINT**	0.0541	0.0256	866	2.11	0.035

© 2024 Quanbeck A et al. *JAMA Network Open*.

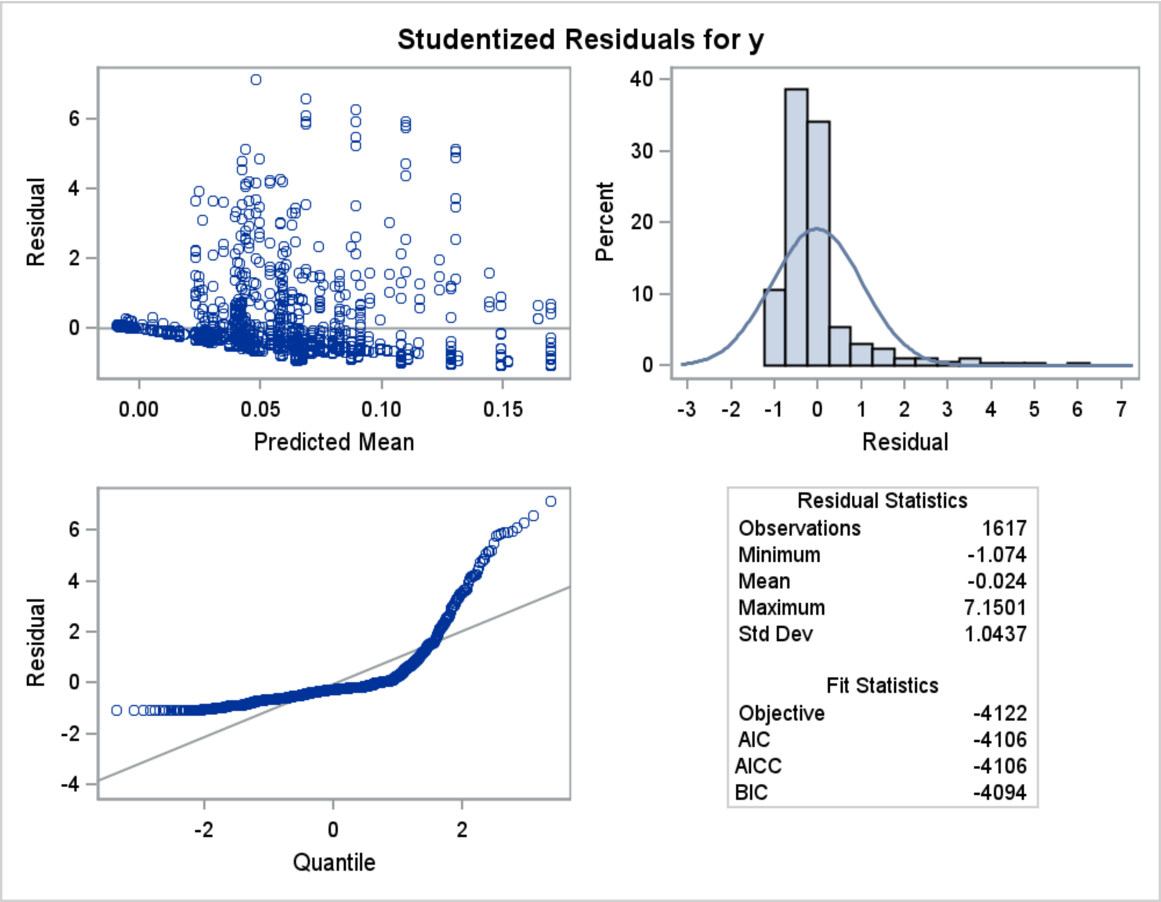

© 2024 Quanbeck A et al. *JAMA Network Open*.

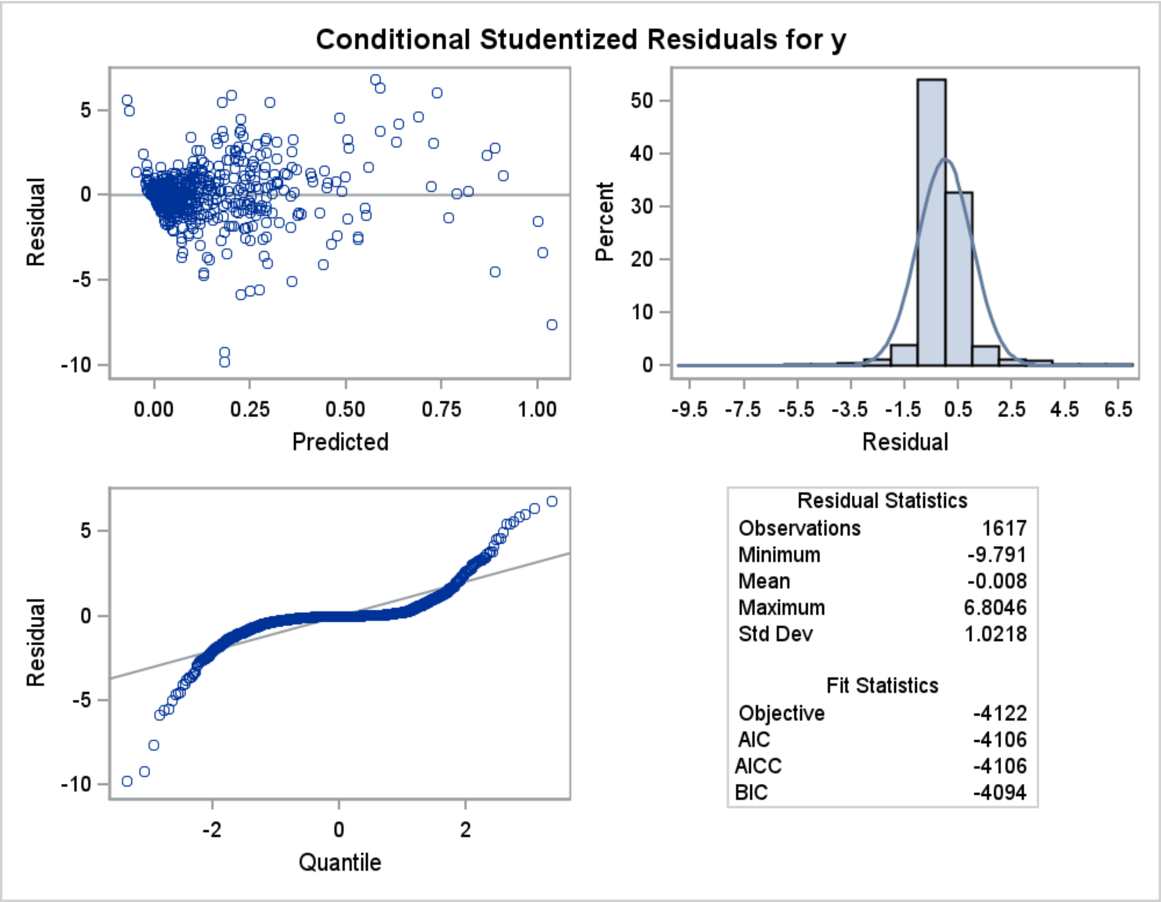

© 2024 Quanbeck A et al. *JAMA Network Open*.

Appendix C – Primary Model Results (Less than 3-Mth Opioid Patients)

Appendix C1 – Average MME Prescribed in Last 3 Months (Less than 3-Mth Opioid Patients)

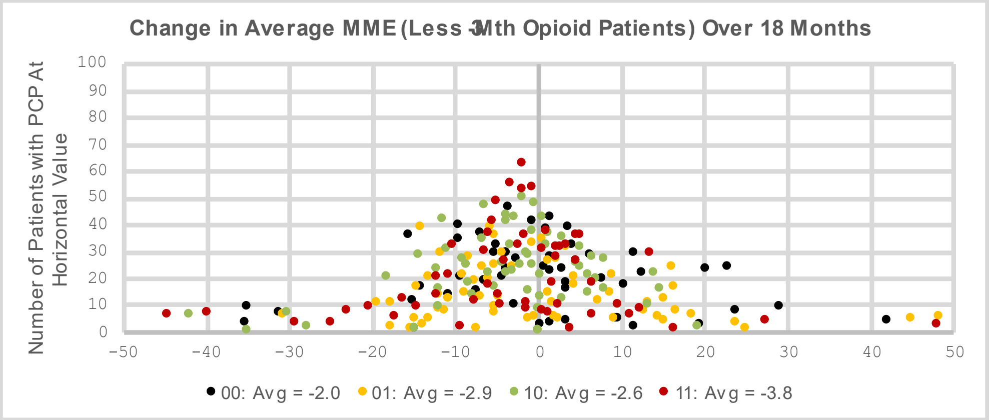

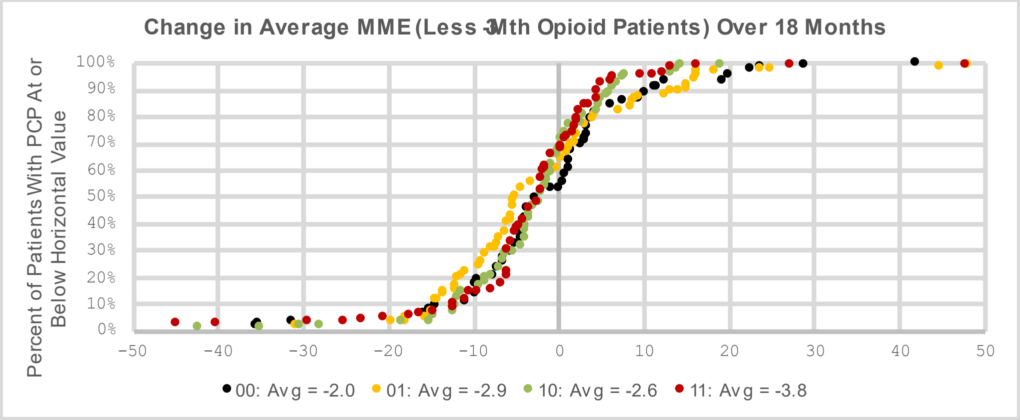

© 2024 Quanbeck A et al. *JAMA Network Open*.

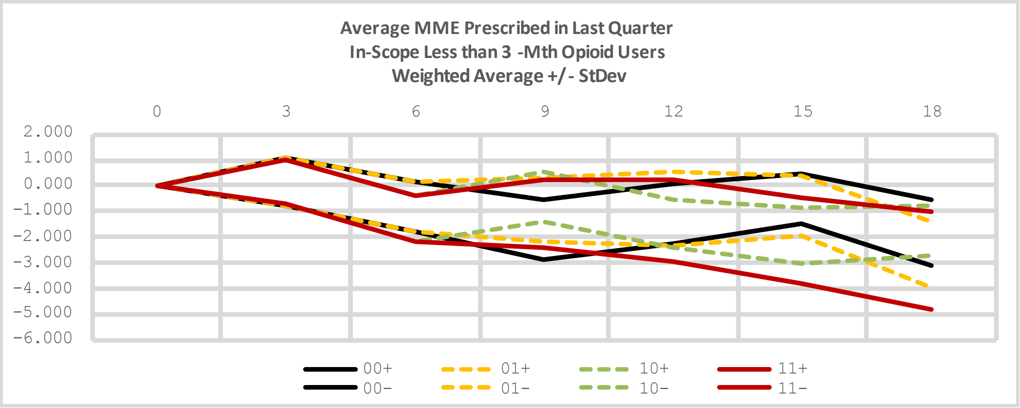

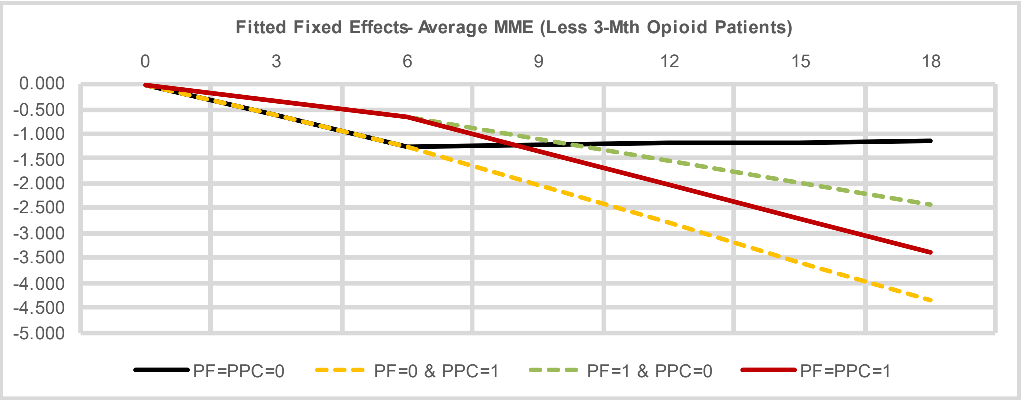

© 2024 Quanbeck A et al. *JAMA Network Open*.

Average MME (Less 3-Mth Opioid Patients)							
Study Month	Estimated Fixed Effect Curves				Differences		
	PF=PPC=0	PF=0 & PPC=1	PF=1 & PPC=0	PF=PPC=1	PF=0 & PPC=1	PF=1 & PPC=0	PF=PPC=1 minus
0	0.000	0.000	0.000	0.000	0.000	0.000	0.000
3	-0.622	-0.622	-0.326	-0.326	0.000	0.296	0.296
6	-1.244	-1.244	-0.651	-0.651	0.000	0.592	0.592
9	-1.218	-2.023	-1.094	-1.331	-0.804	0.124	-0.113
12	-1.193	-2.801	-1.536	-2.011	-1.609	-0.343	-0.819
15	-1.167	-3.580	-1.979	-2.691	-2.413	-0.811	-1.524
18	-1.142	-4.359	-2.421	-3.371	-3.217	-1.279	-2.230
Std Dev of Estimated Values							
0	0.000	0.000	0.000	0.000	0.000	0.000	0.000
3	0.334	0.334	0.299	0.299	0.000	0.426	0.426
6	0.669	0.669	0.598	0.598	0.000	0.853	0.853
9	0.608	0.608	0.550	0.554	0.284	0.766	0.768
12	0.625	0.631	0.574	0.586	0.568	0.790	0.800
15	0.715	0.729	0.660	0.684	0.852	0.918	0.936
18	0.854	0.878	0.790	0.825	1.136	1.113	1.140
t-ratios of Estimated Values							
0							
3	-1.86	-1.86	-1.09	-1.09		0.69	0.69
6	-1.86	-1.86	-1.09	-1.09		0.69	0.69
9	-2.00	-3.32	-1.99	-2.40	-2.83	0.16	-0.15
12	-1.91	-4.44	-2.68	-3.43	-2.83	-0.43	-1.02
15	-1.63	-4.91	-3.00	-3.93	-2.83	-0.88	-1.63
18	-1.34	-4.96	-3.06	-4.08	-2.83	-1.15	-1.96

© 2024 Quanbeck A et al. *JAMA Network Open*.

Covariance Parameter Estimates						
**Cov Parm**	**Subject**	**Estimate**		**Std Dev & Correlation**		
**Intercept**	Clinic	5.1232	Clinic	2.26		
**UN(1,1)**	PCP	10.8787				
**UN(2,1)**	PCP	0.1419				
**UN(2,2)**	PCP	0.0333	PCP	3.30		
**UN(3,1)**	PCP	(0.8948)		23.6%	0.18	
**UN(3,2)**	PCP	(0.0027)		#DIV/0!	#DIV/0!	-
**UN(3,3)**	PCP	-				
**Residual**		607.1300	Residual	24.64		

Solution for Fixed Effects						
**Effect**	**System**	**Estimate**	**Standard Error**	**DF**	**t Value**	**Pr > |t|**
**System**	Bellin	31.6626	0.8001	781	39.58	<.0001
**System**	UW Health	38.7945	0.8130	781	47.71	<.0001
**clinic_size**		-0.00008	0.000070	781	-1.10	0.2696
**t7**		-0.2073	0.1114	249	-1.86	0.0640
**PF_t7**		0.09873	0.1421	781	0.69	0.4874
**t13**		0.2158	0.1580	201	1.37	0.1736
**PF_t13**		-0.2547	0.2072	781	-1.23	0.2195
**PPC_t13**		-0.2681	0.09466	781	-2.83	0.0047
**PFandPPC_t13**		0.1889	0.1288	781	1.47	0.1428

© 2024 Quanbeck A et al. *JAMA Network Open*.

Estimates					
Label	Estimate	Standard Error	DF	t Value	Pr > |t|
NOINT Chg	-1.1414	0.8548	781	-1.34	0.1822
PF Only Chg	-2.4202	0.7903	781	-3.06	0.0023
PPC Only Chg	-4.3591	0.8796	781	-4.96	<.0001
PF & PPC Chg	-3.3708	0.8269	781	-4.08	<.0001
PF Only vs NOINT	-1.2789	1.1136	781	-1.15	0.2512
PPC Only vs NOINT	-3.2177	1.1359	781	-2.83	0.0047
PF Only vs PPC Only 1	1.9389	1.1307	781	1.71	0.0868
PF Only vs PPC Only 2	1.3465	1.2211	781	1.10	0.2705
PF & PPC Interaction	2.2671	1.5455	781	1.47	0.1428
PF & PPC vs NOINT	-2.2295	1.1406	781	-1.95	0.0510

© 2024 Quanbeck A et al. *JAMA Network Open*.

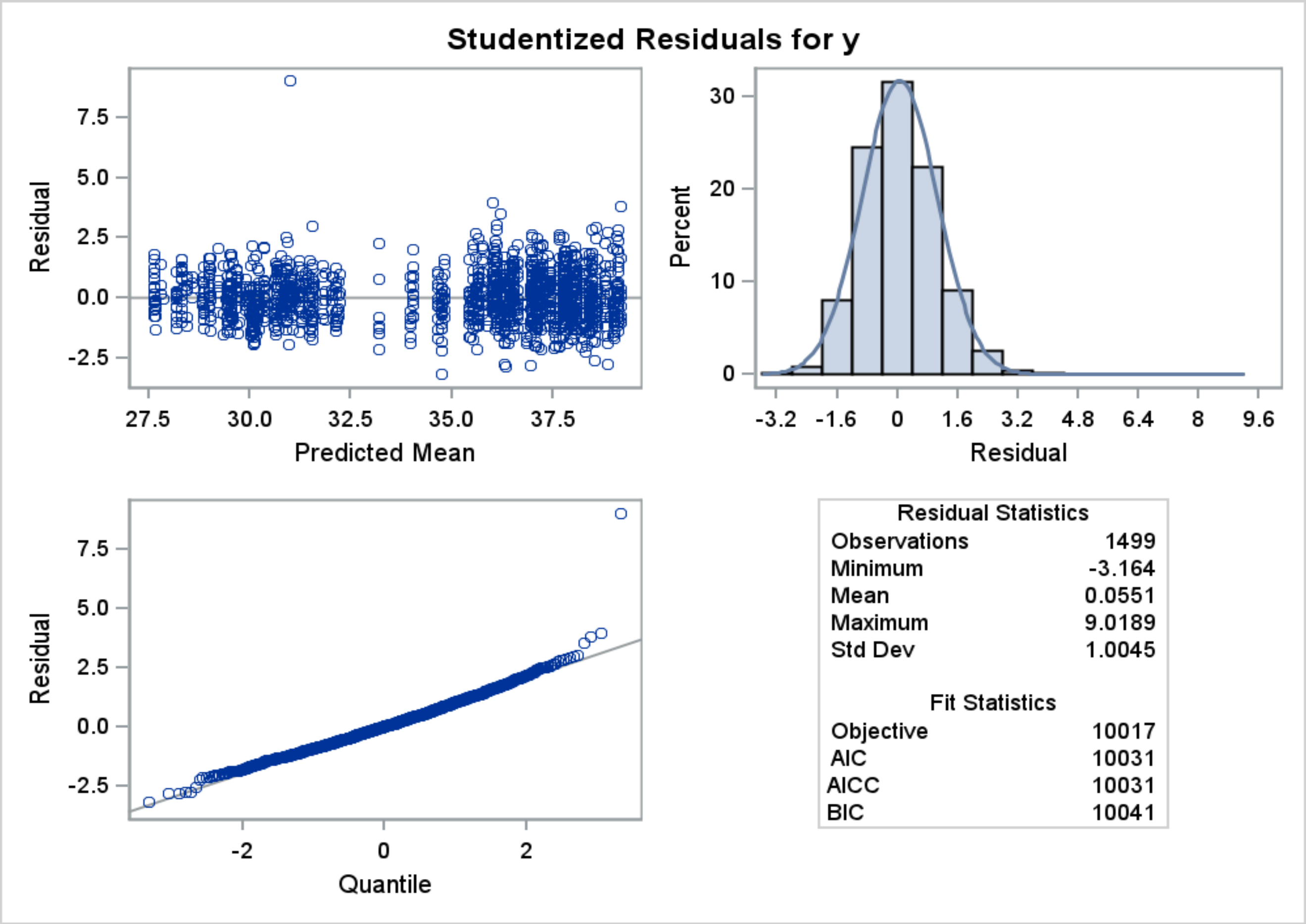

© 2024 Quanbeck A et al. *JAMA Network Open*.

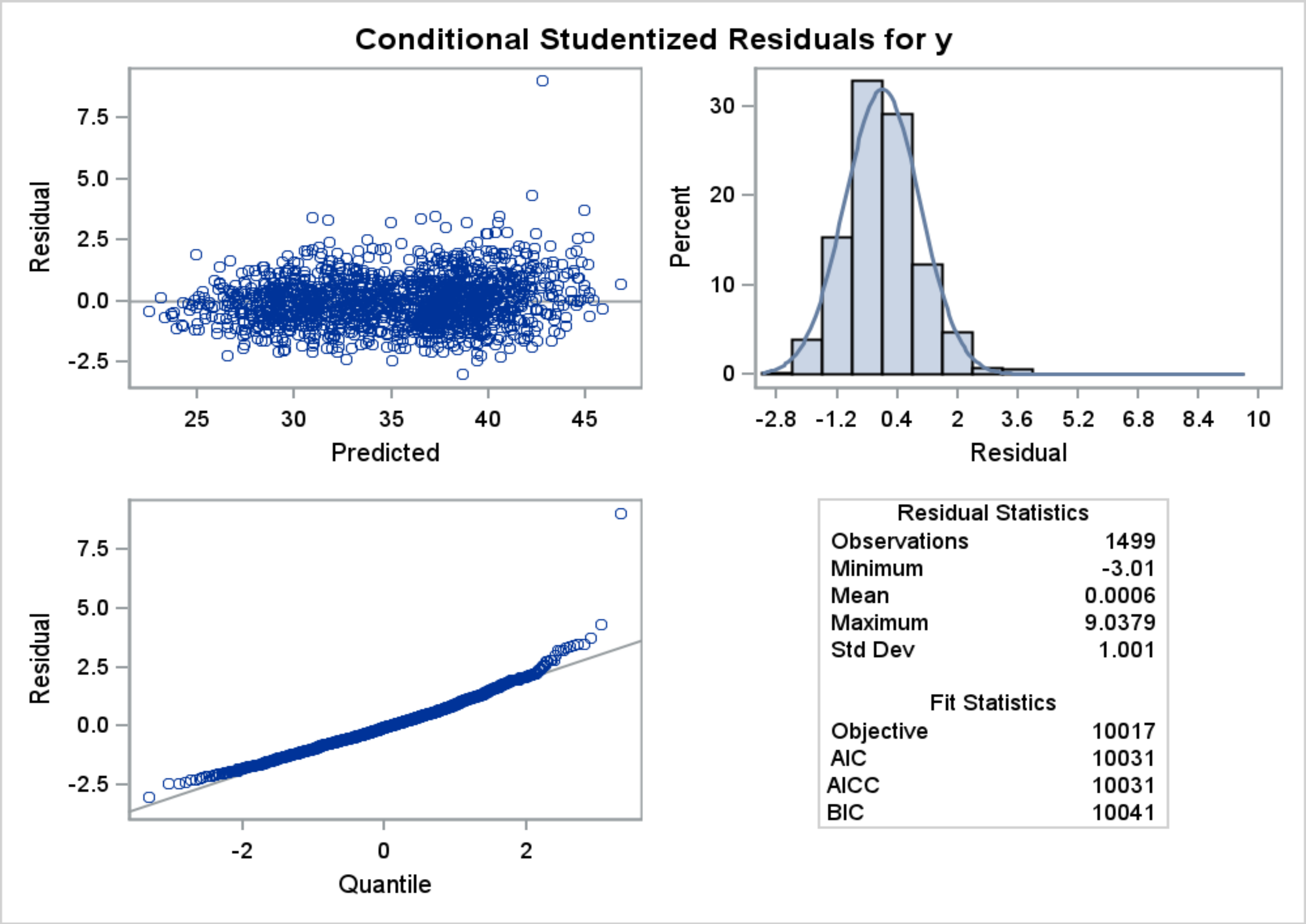

© 2024 Quanbeck A et al. *JAMA Network Open*.

Appendix C2 – Percent of Opioid Patients with Average MME ≥ 90 mg/day (Less than 3-Mth Opioid Patients)

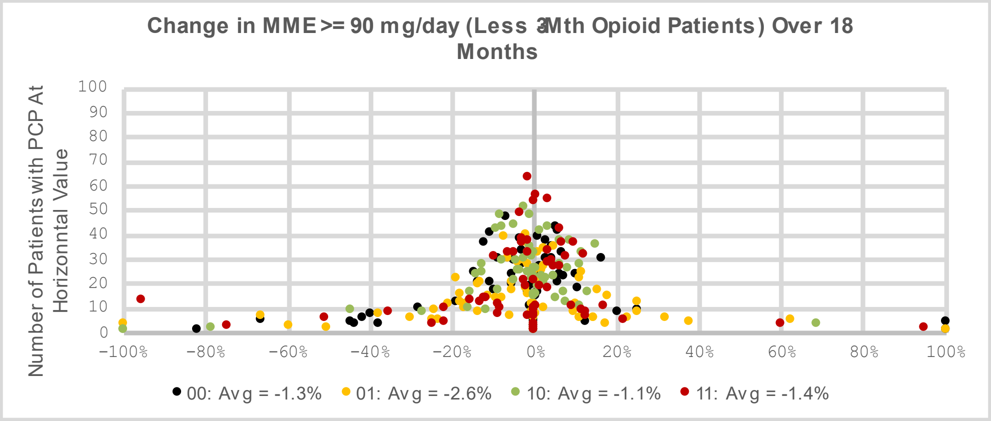

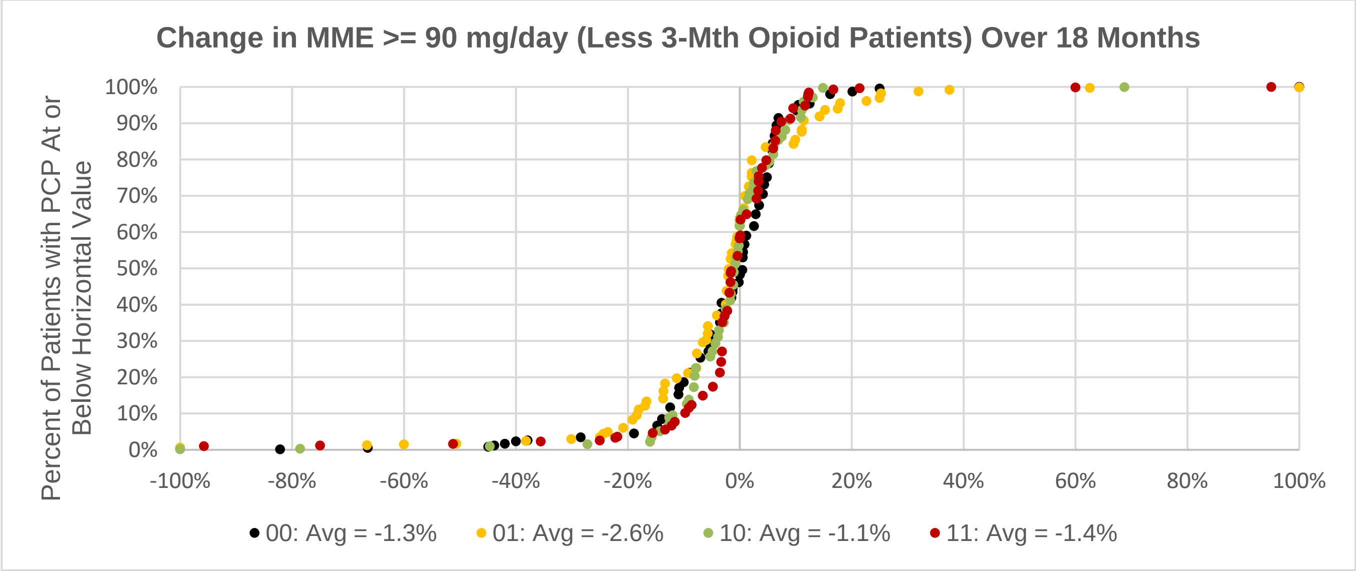

© 2024 Quanbeck A et al. *JAMA Network Open*.

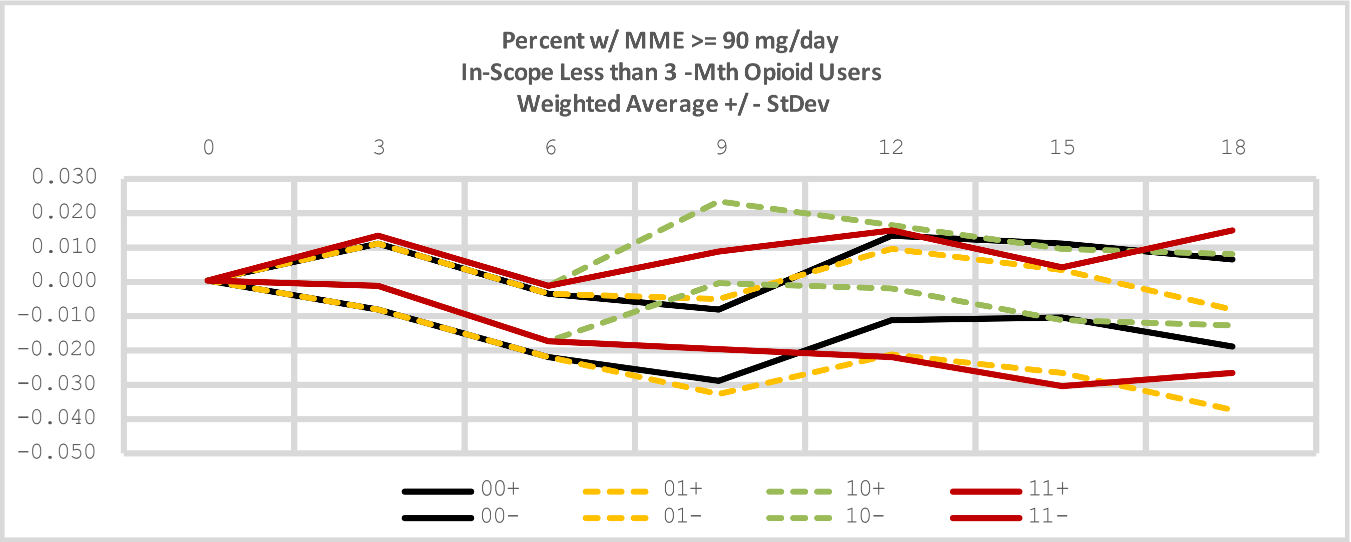

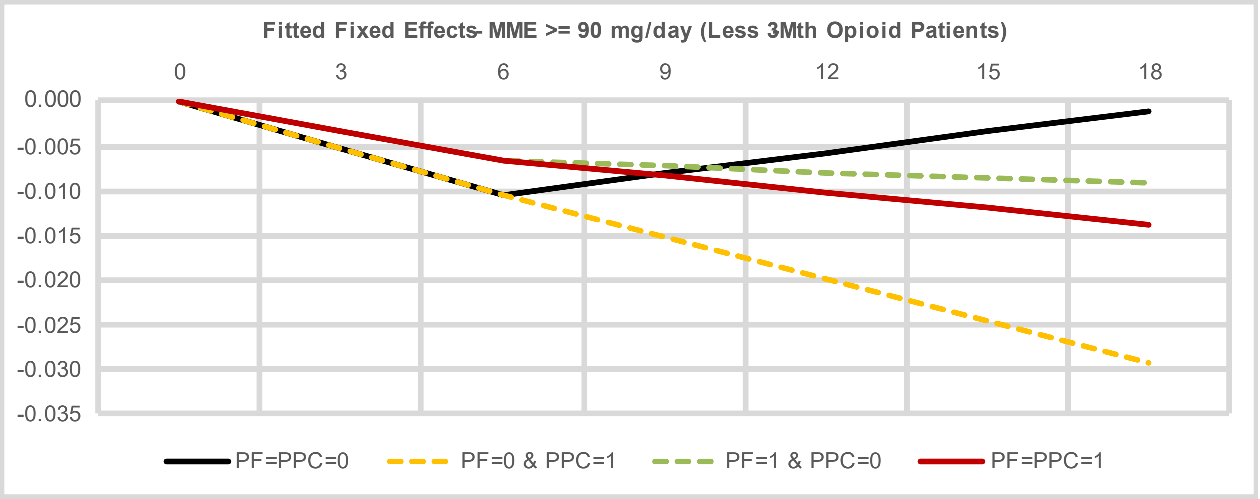

© 2024 Quanbeck A et al. *JAMA Network Open*.

MME >= 90 mg/day (Less 3-Mth Opioid Patients)							
Study Month	Estimated Fixed Effect Curves				Differences		
	PF=PPC=0	PF=0 & PPC=1	PF=1 & PPC=0	PF=PPC=1	PF=0 & PPC=1	PF=1 & PPC=0	PF=PPC=1 minus
0	0.000	0.000	0.000	0.000	0.000	0.000	0.000
3	-0.005	-0.005	-0.003	-0.003	0.000	0.002	0.002
6	-0.011	-0.011	-0.007	-0.007	0.000	0.004	0.004
9	-0.008	-0.015	-0.007	-0.008	-0.007	0.001	0.000
12	-0.006	-0.020	-0.008	-0.010	-0.014	-0.002	-0.004
15	-0.003	-0.025	-0.009	-0.012	-0.021	-0.005	-0.009
18	-0.001	-0.029	-0.009	-0.014	-0.028	-0.008	-0.013
Std Dev of Estimated Values							
0	0.000	0.000	0.000	0.000	0.000	0.000	0.000
3	0.003	0.003	0.003	0.003	0.000	0.003	0.003
6	0.006	0.006	0.006	0.006	0.000	0.007	0.007
9	0.005	0.005	0.005	0.005	0.002	0.006	0.006
12	0.005	0.005	0.005	0.005	0.005	0.006	0.006
15	0.006	0.006	0.006	0.006	0.007	0.007	0.007
18	0.007	0.007	0.007	0.007	0.010	0.009	0.009
t-ratios of Estimated Values							
0							
3	-1.73	-1.73	-1.18	-1.18		0.57	0.57
6	-1.73	-1.73	-1.18	-1.18		0.57	0.57
9	-1.54	-2.88	-1.47	-1.68	-2.94	0.16	-0.04
12	-1.10	-3.75	-1.59	-1.99	-2.94	-0.37	-0.74
15	-0.57	-4.01	-1.51	-2.03	-2.94	-0.73	-1.18
18	-0.15	-3.92	-1.35	-1.92	-2.94	-0.90	-1.36

Covariance Parameter Estimates		
Cov Parm	Subject	Estimate
Intercept	Clinic	0.0001
UN(1,1)	PCP	0.0004
UN(2,1)	PCP	(0.0000)
UN(2,2)	PCP	0.0000
UN(3,1)	PCP	(0.0000)
UN(3,2)	PCP	(0.0000)
UN(3,3)	PCP	-
Residual		0.0614

	Std Dev & Correlation		
Clinic	0.01		
PCP	0.02		
	-33.9%	0.00	
	#DIV/0!	#DIV/0!	-
Residual	0.25		

© 2024 Quanbeck A et al. *JAMA Network Open*.

Solution for Fixed Effects						
Effect	System	Estimate	Standard Error	DF	t Value	Pr > |t|
System	Bellin	0.04892	0.004799	784	10.19	<.0001
System	UW Health	0.09206	0.004816	784	19.12	<.0001
clinic_size		0	0	784	-Infty	<.0001
t7		-0.00175	0.001009	249	-1.74	0.0837
PF_t7		0.000659	0.001152	784	0.57	0.5676
t13		0.002535	0.001489	201	1.70	0.0902
PF_t13		-0.00167	0.001868	784	-0.89	0.3717
PPC_t13		-0.00235	0.000801	784	-2.93	0.0035
PFandPPC_t13		0.001970	0.001084	784	1.82	0.0694

Estimates					
Label	Estimate	Standard Error	DF	t Value	Pr > |t|
NOINT Chg	-0.00113	0.007417	784	-0.15	0.8792
PF Only Chg	-0.00931	0.006848	784	-1.36	0.1744
PPC Only Chg	-0.02931	0.007598	784	-3.86	0.0001
PF & PPC Chg	-0.01384	0.007166	784	-1.93	0.0537
PF Only vs NOINT	-0.00818	0.008984	784	-0.91	0.3627
PPC Only vs NOINT	-0.02818	0.009608	784	-2.93	0.0035
PF Only vs PPC Only 1	0.02000	0.009099	784	2.20	0.0283
PF Only vs PPC Only 2	0.01604	0.01142	784	1.40	0.1604
PF & PPC Interaction	0.02364	0.01300	784	1.82	0.0694
PF & PPC vs NOINT	-0.01272	0.009263	784	-1.37	0.1702

© 2024 Quanbeck A et al. *JAMA Network Open*.

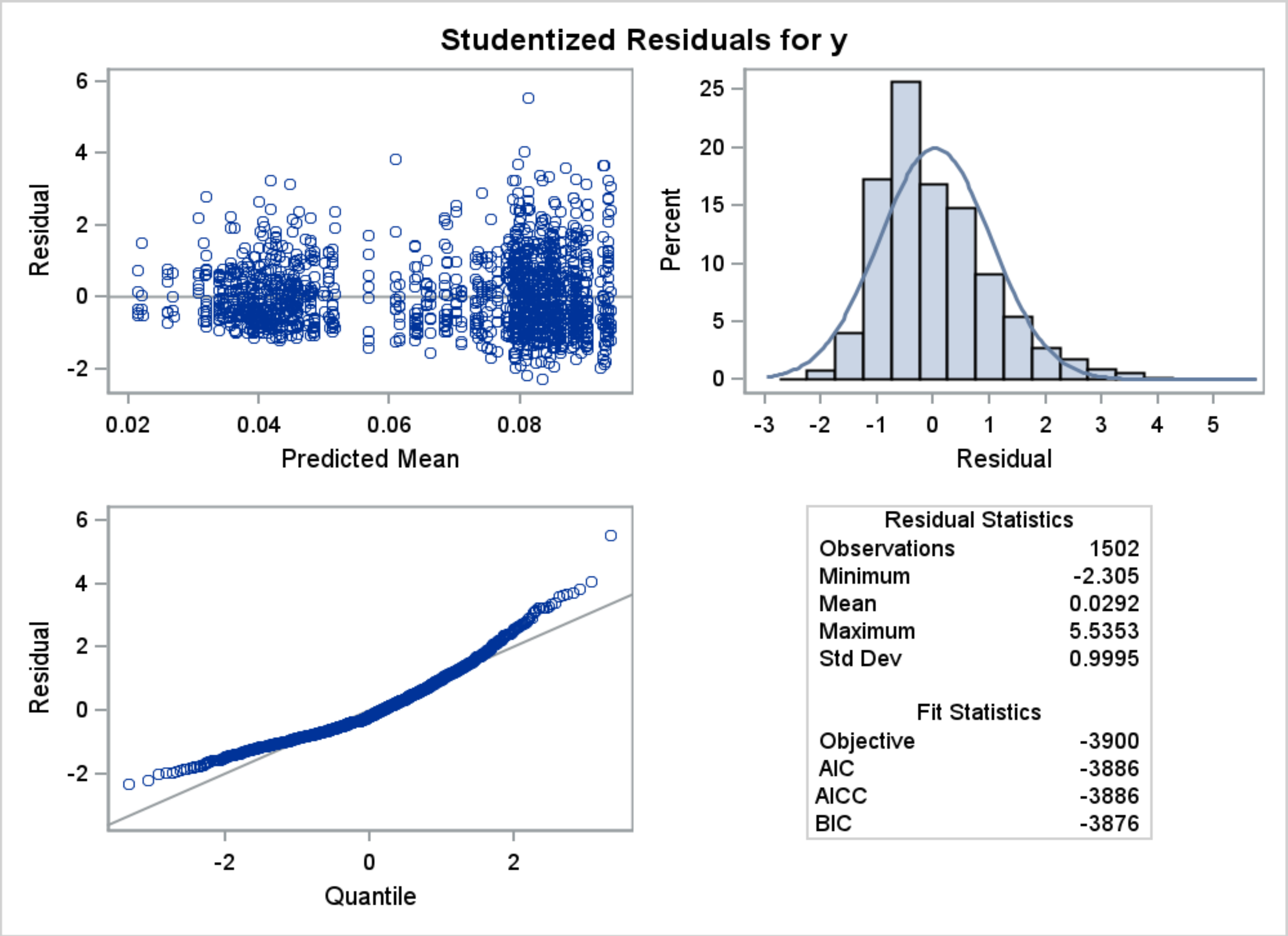

© 2024 Quanbeck A et al. *JAMA Network Open*.

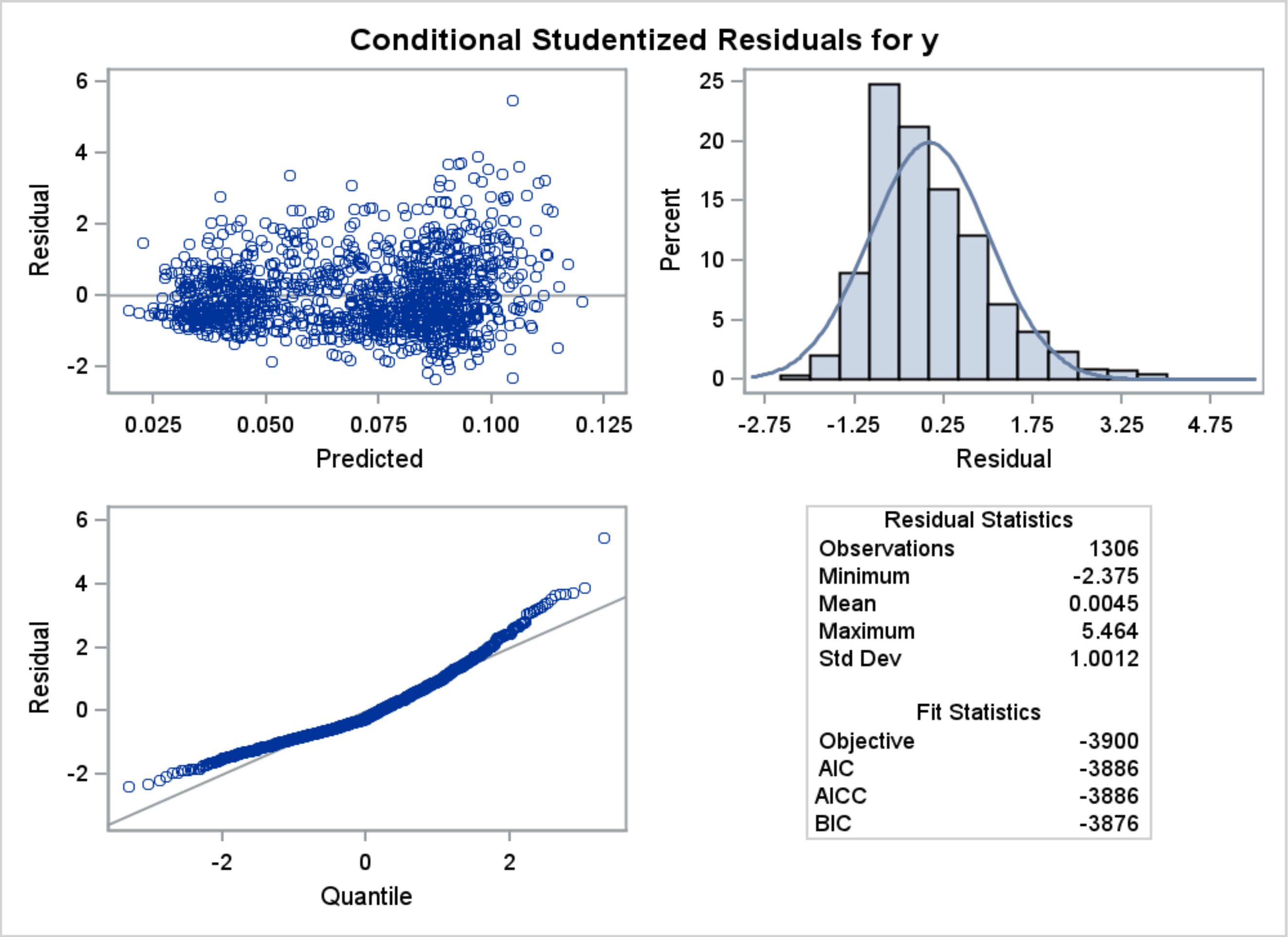

Appendix C3 – Percent of Opioid Patients with Benzo’s in Last 3 Months (Less than 3-Mth Opioid Patients)

© 2024 Quanbeck A et al. *JAMA Network Open*.

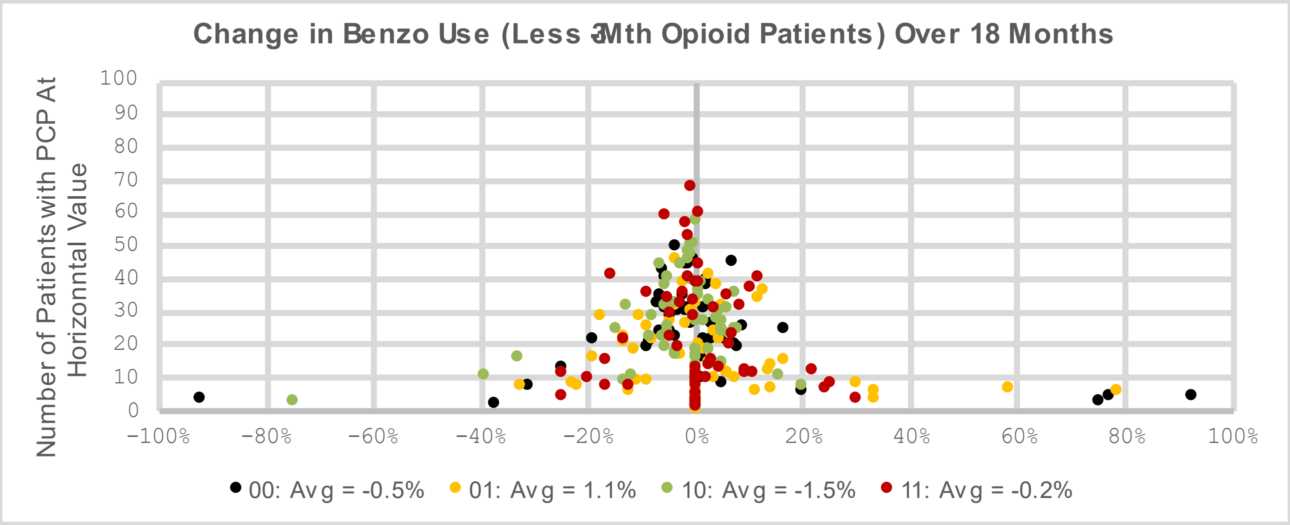

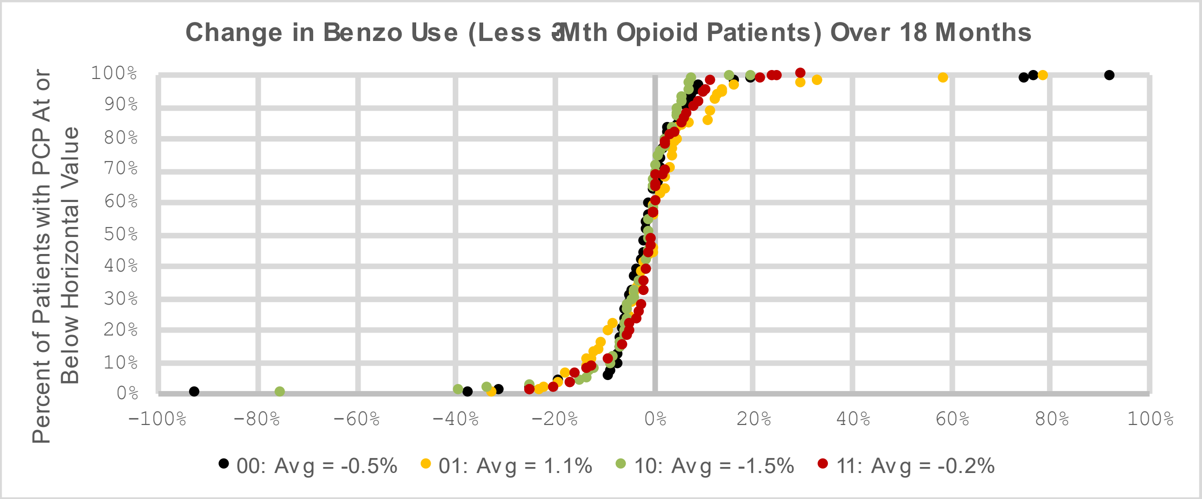

© 2024 Quanbeck A et al. *JAMA Network Open*.

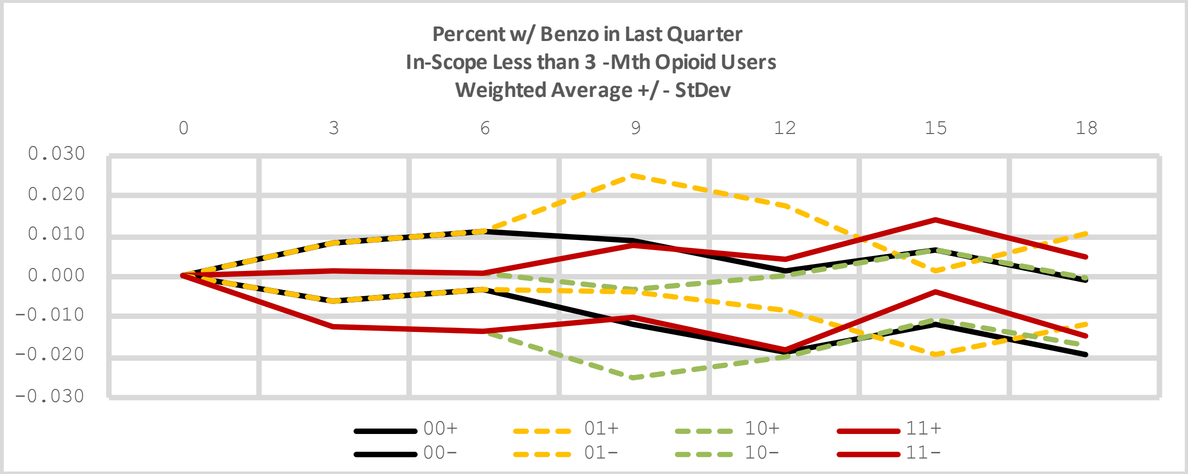

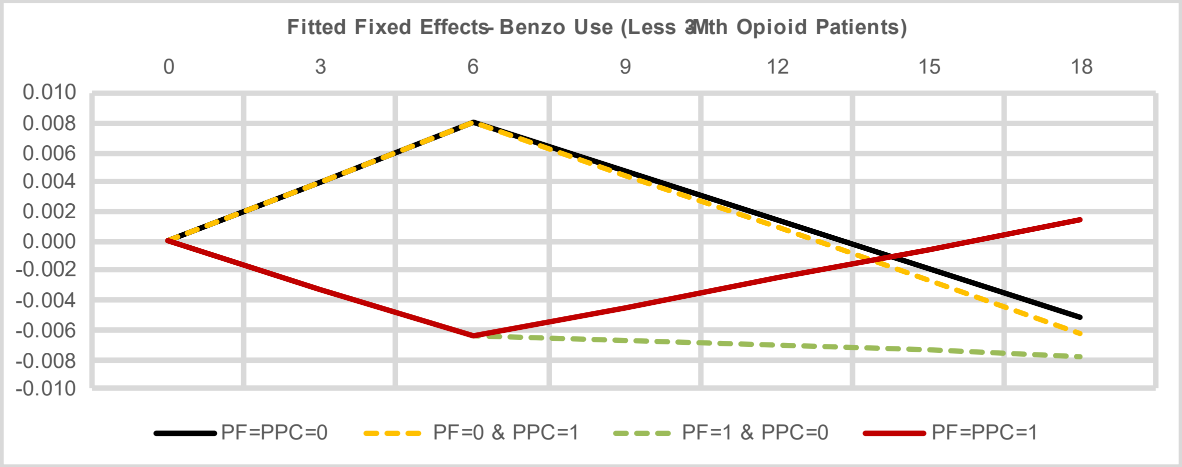

© 2024 Quanbeck A et al. *JAMA Network Open*.

Benzo Use (Less 3-Mth Opioid Patients)

Study Month	Estimated Fixed Effect Curves				Differences		
	PF=PPC=0	PF=0 & PPC=1	PF=1 & PPC=0	PF=PPC=1	PF=0 & PPC=1	PF=1 & PPC=0	PF=PPC=1 minus
0	0.000	0.000	0.000	0.000	0.000	0.000	0.000
3	0.004	0.004	-0.003	-0.003	0.000	-0.007	-0.007
6	0.008	0.008	-0.006	-0.006	0.000	-0.015	-0.015
9	0.005	0.005	-0.007	-0.004	0.000	-0.012	-0.009
12	0.001	0.001	-0.007	-0.003	-0.001	-0.009	-0.004
15	-0.002	-0.003	-0.007	-0.001	-0.001	-0.006	0.001
18	-0.005	-0.006	-0.008	0.001	-0.001	-0.003	0.007
**Std Dev of Estimated Values**							
0	0.000	0.000	0.000	0.000	0.000	0.000	0.000
3	0.003	0.003	0.003	0.003	0.000	0.004	0.004
6	0.006	0.006	0.006	0.006	0.000	0.008	0.008
9	0.006	0.006	0.005	0.005	0.003	0.006	0.007
12	0.006	0.006	0.005	0.005	0.005	0.007	0.007
15	0.006	0.007	0.006	0.006	0.008	0.008	0.008
18	0.007	0.008	0.007	0.007	0.010	0.010	0.010
**t-ratios of Estimated Values**							
0							
3	1.26	1.26	-1.07	-1.07		-1.93	-1.93
6	1.26	1.26	-1.07	-1.07		-1.93	-1.93
9	0.85	0.79	-1.27	-0.84	-0.11	-1.79	-1.43
12	0.27	0.16	-1.34	-0.47	-0.11	-1.32	-0.60
15	-0.30	-0.41	-1.25	-0.09	-0.11	-0.73	0.17
18	-0.70	-0.80	-1.09	0.20	-0.11	-0.27	0.68

Covariance Parameter Estimates				Std Dev & Correlation		
**Cov Parm**	**Subject**	**Estimate**				
**Intercept**	Clinic	0.000034	Clinic	0.0058		
**UN(1,1)**	PCP	0.000789				
**UN(2,1)**	PCP	-0.00003				
**UN(2,2)**	PCP	0.000017	PCP	0.0281		
**UN(3,1)**	PCP	0.000050		-25.9%	0.0041	
**UN(3,2)**	PCP	-0.00002		33.1%	-90.1%	0.0054
**UN(3,3)**	PCP	0.000029				
**Residual**		0.06528	Residual	0.2555		

© 2024 Quanbeck A et al. *JAMA Network Open*.

Solution for Fixed Effects						
**Effect**	**System**	**Estimate**	**Standard Error**	**DF**	**t Value**	**Pr > |t|**
**System**	Bellin	0.05995	0.005351	786	11.20	<.0001
**System**	UW Health	0.08827	0.005032	786	17.54	<.0001
**clinic_size**		0	0	786	Infty	<.0001
**t7**		0.001353	0.001077	249	1.26	0.2105
**PF_t7**		-0.00243	0.001257	786	-1.93	0.0535
**t13**		-0.00246	0.001554	201	-1.58	0.1145
**PF_t13**		0.003428	0.001926	786	1.78	0.0754
**PPC_t13**		-0.00009	0.000838	786	-0.10	0.9176
**PFandPPC_t13**		0.000857	0.001127	786	0.76	0.4471

Estimates

**Label**	**Estimate**	**Standard Error**	**DF**	**t Value**	**Pr > |t|**
**NOINT Chg**	-0.00521	0.007328	786	-0.71	0.4773
**PF Only Chg**	-0.00782	0.006806	786	-1.15	0.2509
**PPC Only Chg**	-0.00625	0.007812	786	-0.80	0.4238
**PF & PPC Chg**	0.001428	0.006962	786	0.21	0.8375
**PF Only vs NOINT**	-0.00261	0.009426	786	-0.28	0.7819
**PPC Only vs NOINT**	-0.00104	0.01005	786	-0.10	0.9176
**PF Only vs PPC Only 1**	-0.00157	0.009741	786	-0.16	0.8720
**PF Only vs PPC Only 2**	0.01301	0.01148	786	1.13	0.2575
**PF & PPC Interaction**	0.01029	0.01353	786	0.76	0.4471
**PF & PPC vs NOINT**	0.006638	0.009580	786	0.69	0.4885

© 2024 Quanbeck A et al. *JAMA Network Open*.

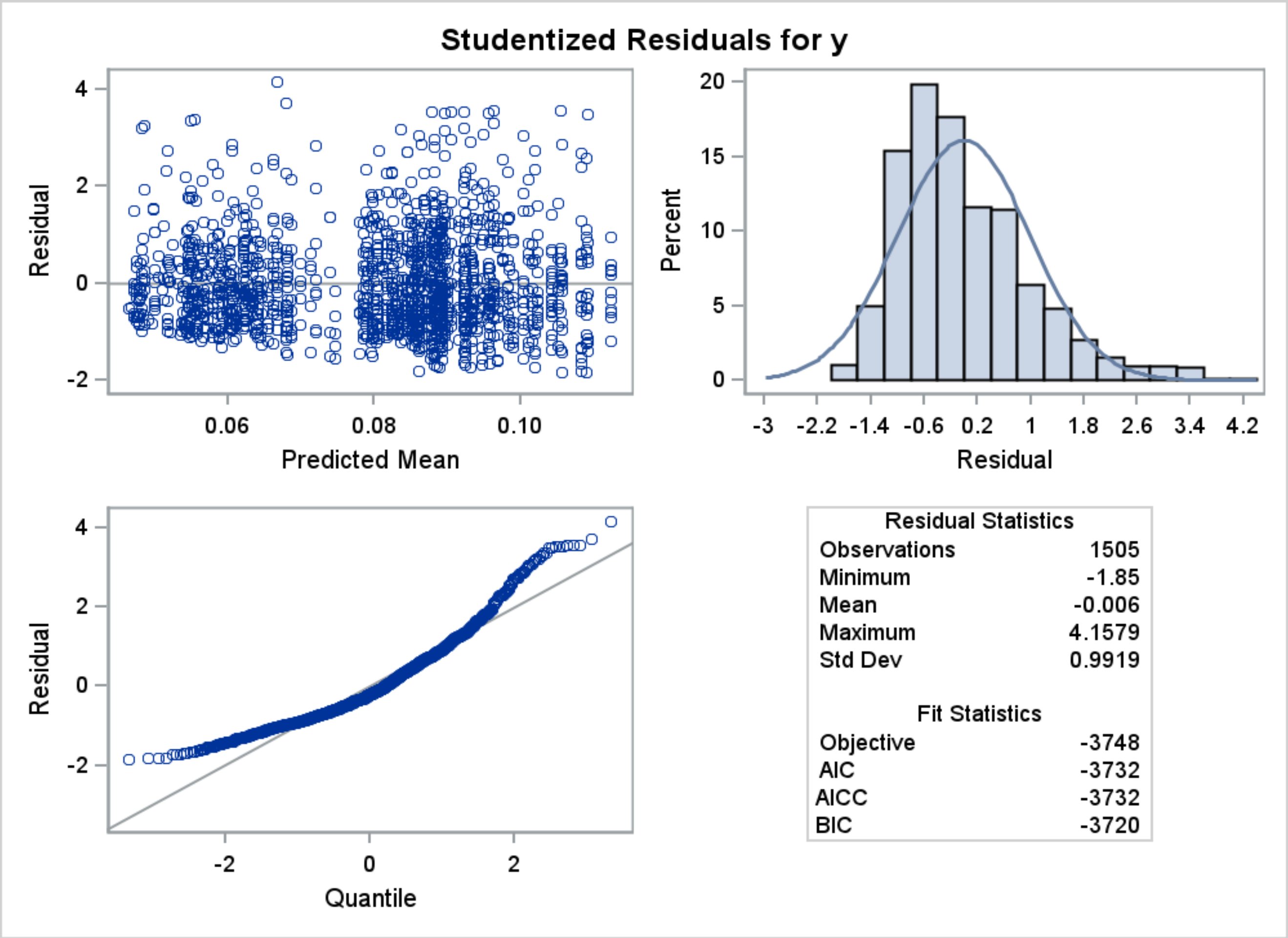

© 2024 Quanbeck A et al. *JAMA Network Open*.

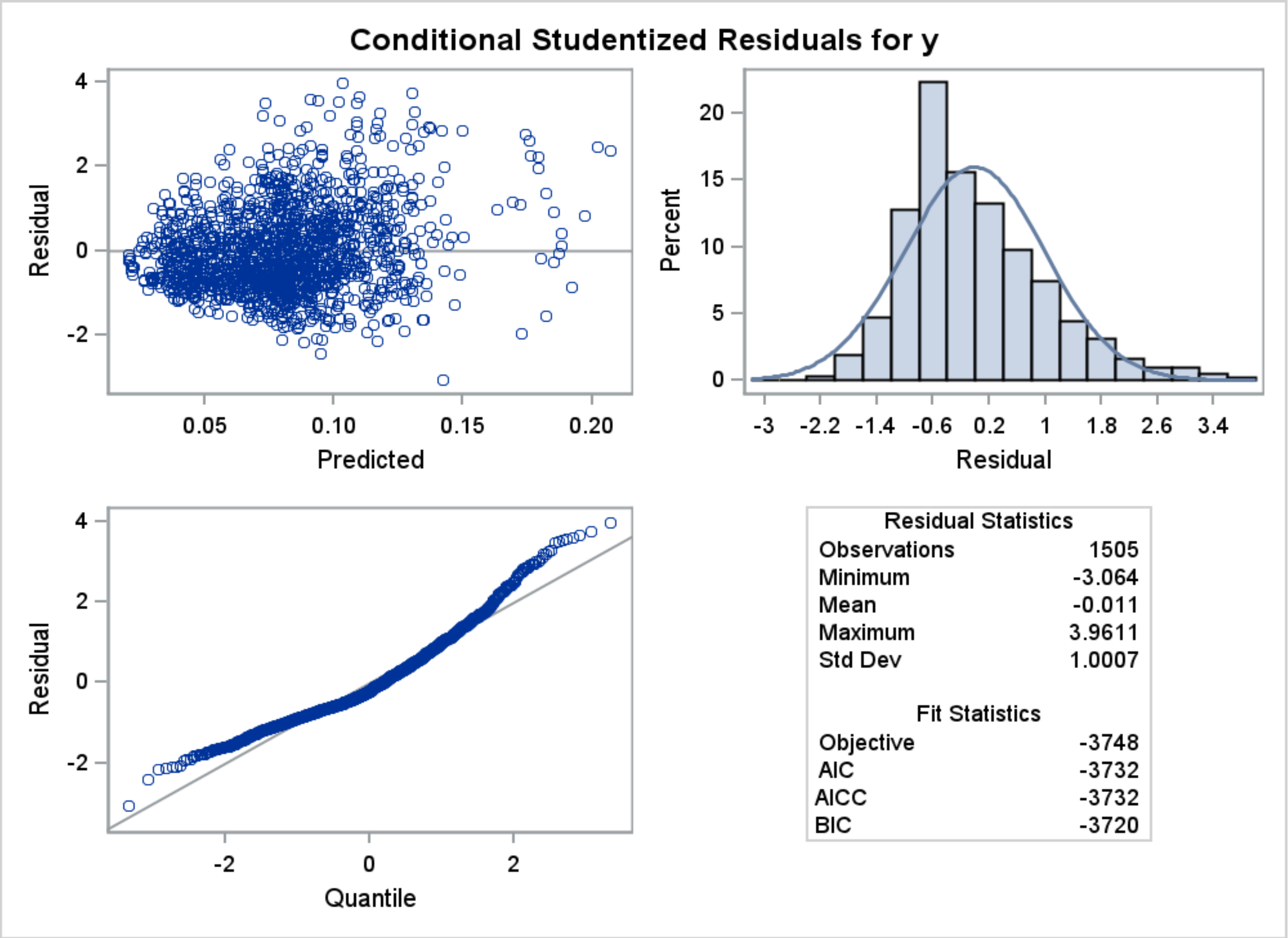

Appendix C4 – Percent of Opioid Patients with Urine Drug Screen in Last 12 Months (Less than 3-Mth opioid Patients)

© 2024 Quanbeck A et al. *JAMA Network Open*.

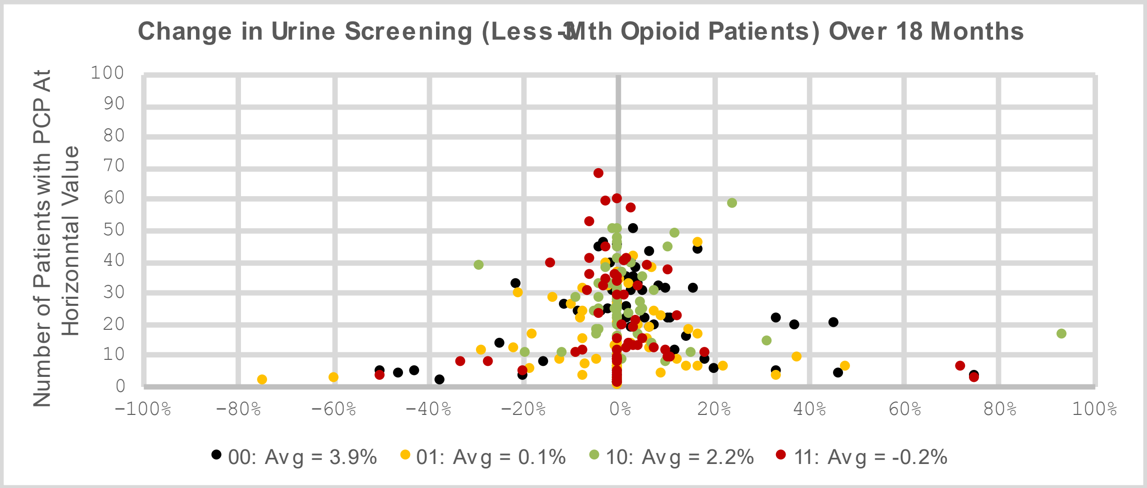

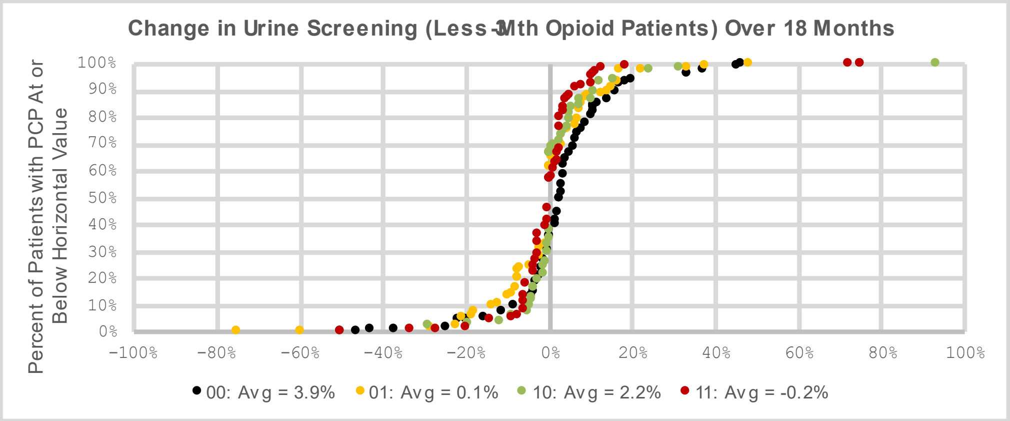

© 2024 Quanbeck A et al. *JAMA Network Open*.

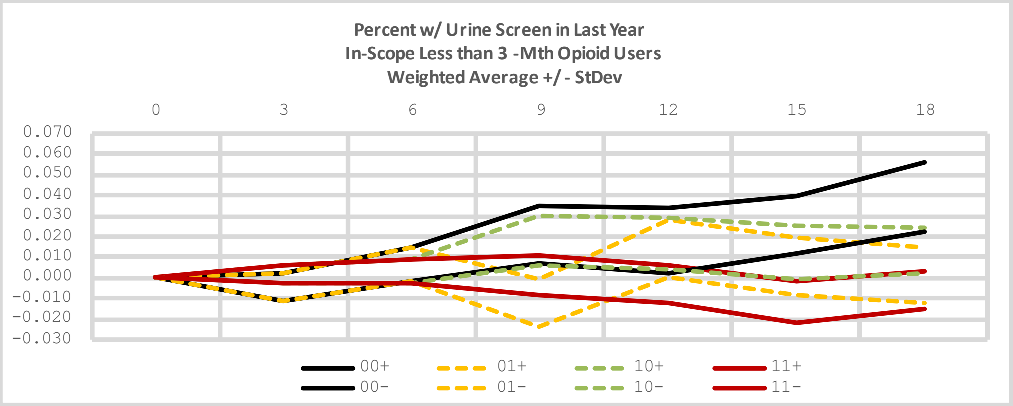

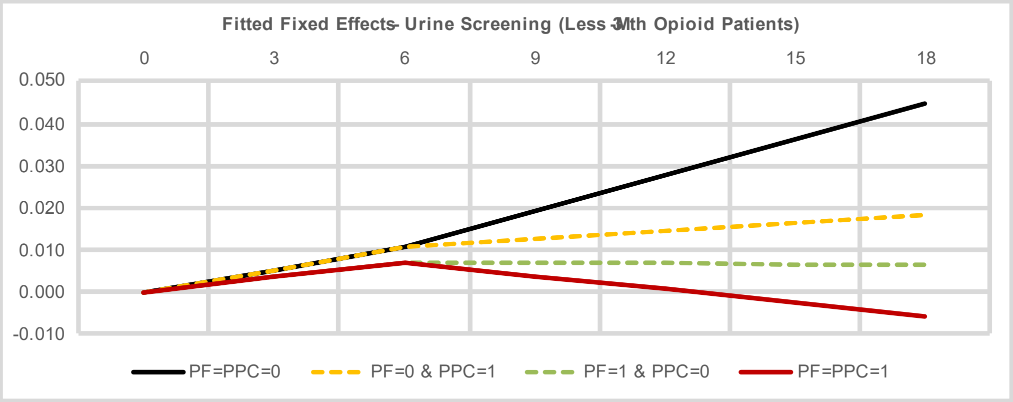

© 2024 Quanbeck A et al. *JAMA Network Open*.

Urine Screening (Less 3-Mth Opioid Patients)

Study Month	Estimated Fixed Effect Curves				Differences		
	PF=PPC=0	PF=0 & PPC=1	PF=1 & PPC=0	PF=PPC=1	PF=0 & PPC=1	PF=1 & PPC=0	PF=PPC=1 minus
0	0.000	0.000	0.000	0.000	0.000	0.000	0.000
3	0.005	0.005	0.003	0.003	0.000	-0.002	-0.002
6	0.010	0.010	0.007	0.007	0.000	-0.003	-0.003
9	0.019	0.012	0.007	0.004	-0.007	-0.012	-0.015
12	0.028	0.014	0.007	0.001	-0.013	-0.021	-0.027
15	0.036	0.016	0.006	-0.003	-0.020	-0.030	-0.039
18	0.045	0.018	0.006	-0.006	-0.026	-0.039	-0.051
**Std Dev of Estimated Values**							
0	0.000	0.000	0.000	0.000	0.000	0.000	0.000
3	0.004	0.004	0.003	0.003	0.000	0.005	0.005
6	0.007	0.007	0.007	0.007	0.000	0.009	0.009
9	0.007	0.007	0.007	0.007	0.003	0.009	0.009
12	0.008	0.008	0.007	0.007	0.007	0.010	0.010
15	0.009	0.009	0.009	0.009	0.010	0.012	0.012
18	0.011	0.011	0.011	0.011	0.014	0.015	0.015
**t-ratios of Estimated Values**							
0							
3	1.46	1.46	1.04	1.04		-0.37	-0.37
6	1.46	1.46	1.04	1.04		-0.37	-0.37
9	2.71	1.79	1.02	0.57	-1.91	-1.34	-1.67
12	3.56	1.88	0.89	0.08	-1.91	-2.05	-2.64
15	3.96	1.81	0.74	-0.29	-1.91	-2.43	-3.16
18	4.09	1.69	0.60	-0.54	-1.91	-2.61	-3.40

Covariance Parameter Estimates

Cov Parm	Subject	Estimate
Intercept	Clinic	0.000735
UN(1,1)	PCP	0.002317
UN(2,1)	PCP	-0.00020
UN(2,2)	PCP	0.000061
UN(3,1)	PCP	0.000135
UN(3,2)	PCP	-0.00006
UN(3,3)	PCP	0.000068
Residual		0.04382

Std Dev & Correlation

Clinic	0.0271		
PCP	0.0481		
	-53.2%	0.0078	
	34.0%	-93.2%	0.0082
Residual	0.2093		

© 2024 Quanbeck A et al. *JAMA Network Open*.

Solution for Fixed Effects						
Effect	System	Estimate	Standard Error	DF	t Value	Pr > |t|
System	Bellin	0.06105	0.009489	786	6.43	<.0001
System	UW Health	0.05636	0.009531	786	5.91	<.0001
clinic_size		0	0	786	-Infty	<.0001
t7		0.001744	0.001191	249	1.47	0.1442
PF_t7		-0.00058	0.001556	786	-0.37	0.7107
t13		0.001122	0.001601	201	0.70	0.4845
PF_t13		-0.00234	0.002142	786	-1.09	0.2747
PPC_t13		-0.00220	0.001153	786	-1.91	0.0570
PFandPPC_t13		0.001191	0.001598	786	0.75	0.4564

Estimates					
Label	Estimate	Standard Error	DF	t Value	Pr > |t|
NOINT Chg	0.04486	0.01093	786	4.11	<.0001
PF Only Chg	0.006378	0.01047	786	0.61	0.5426
PPC Only Chg	0.01849	0.01089	786	1.70	0.0899
PF & PPC Chg	-0.00569	0.01069	786	-0.53	0.5947
PF Only vs NOINT	-0.03848	0.01471	786	-2.62	0.0091
PPC Only vs NOINT	-0.02636	0.01383	786	-1.91	0.0570
PF Only vs PPC Only 1	-0.01212	0.01469	786	-0.82	0.4097
PF Only vs PPC Only 2	-0.00865	0.01394	786	-0.62	0.5350
PF & PPC Interaction	0.01429	0.01918	786	0.75	0.4564
PF & PPC vs NOINT	-0.05055	0.01488	786	-3.40	0.0007

© 2024 Quanbeck A et al. *JAMA Network Open*.

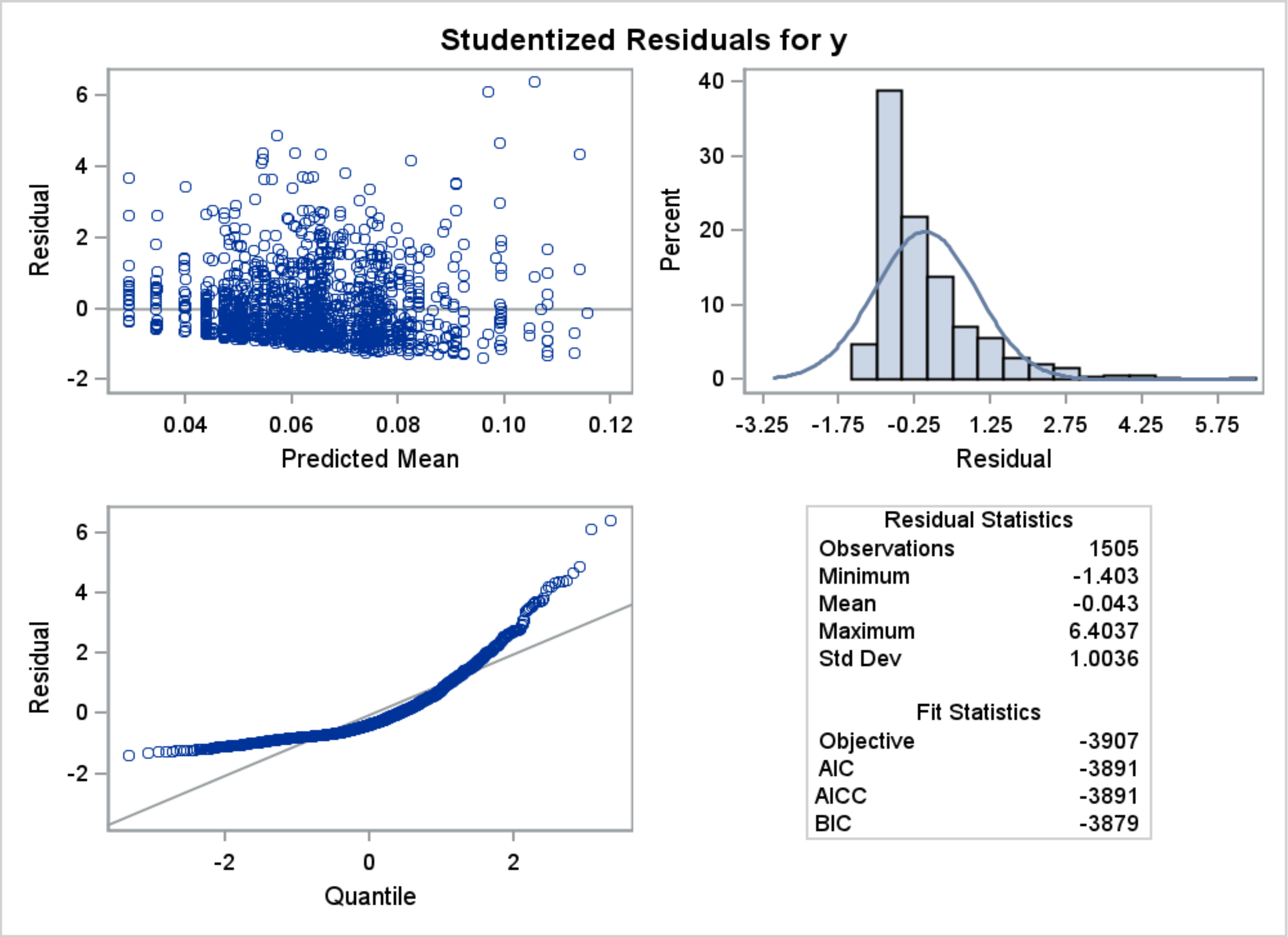

© 2024 Quanbeck A et al. *JAMA Network Open*.

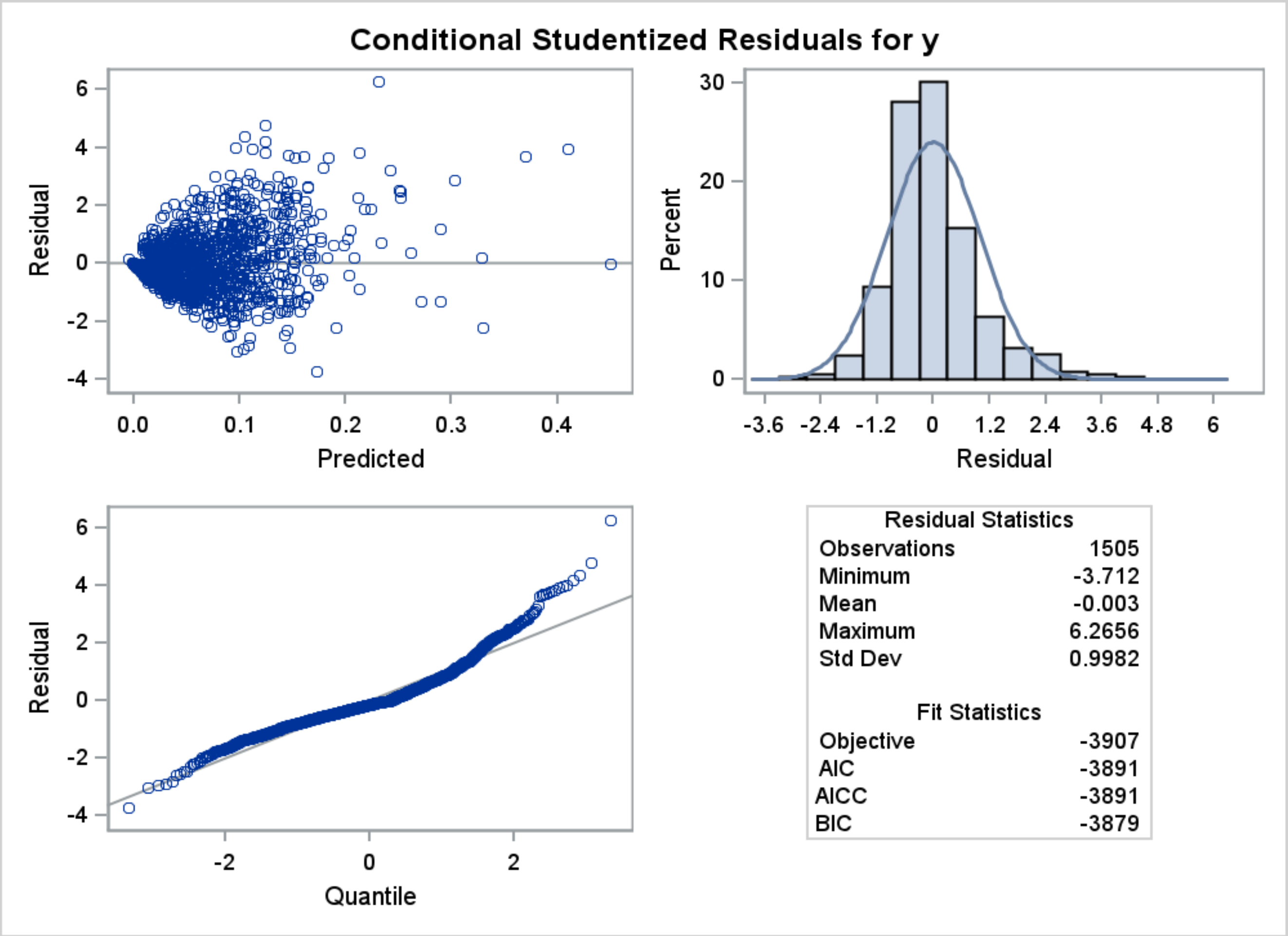

Appendix C5 – Percent of Opioid Patients with Treatment Agreement in Last 12 Months (Less than 3-Mth Opioid Patients)

© 2024 Quanbeck A et al. *JAMA Network Open*.

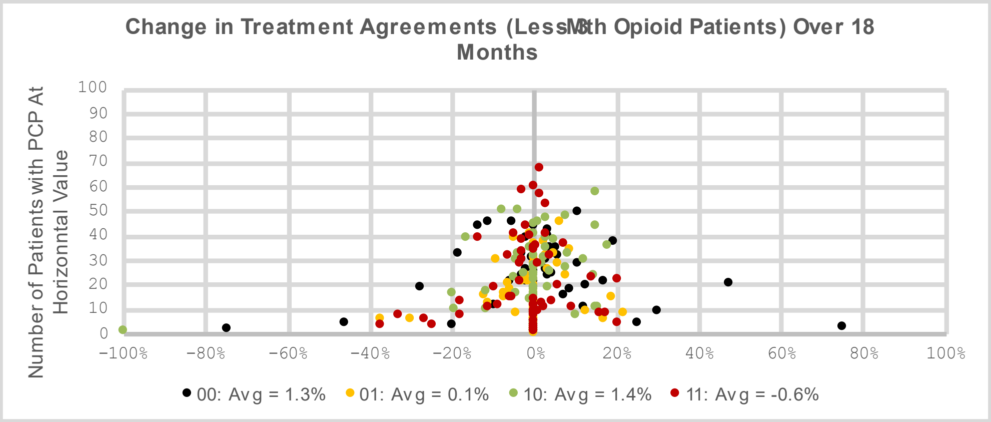

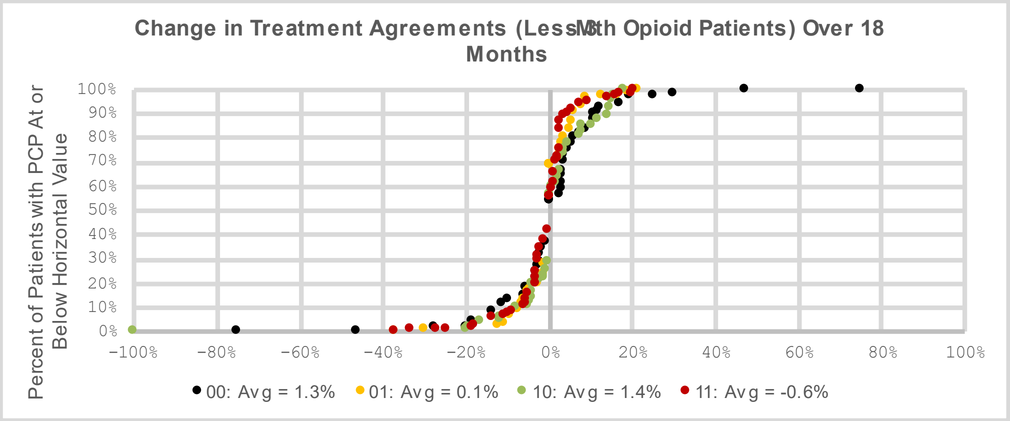

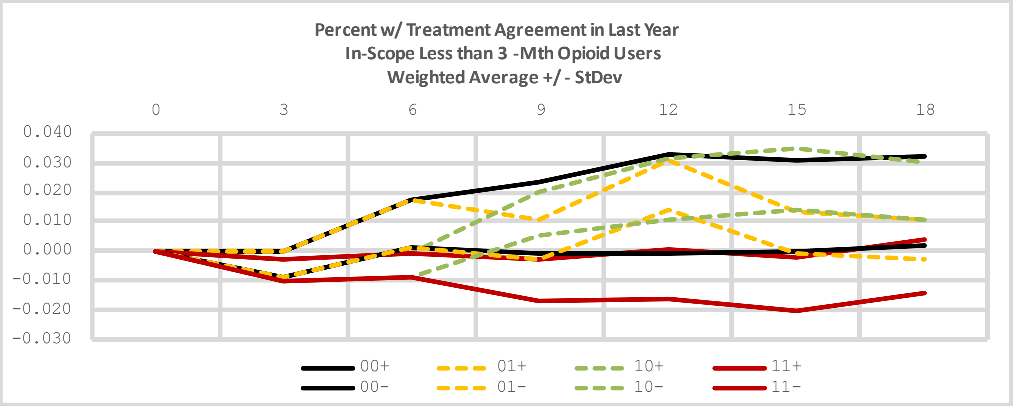

© 2024 Quanbeck A et al. *JAMA Network Open*.

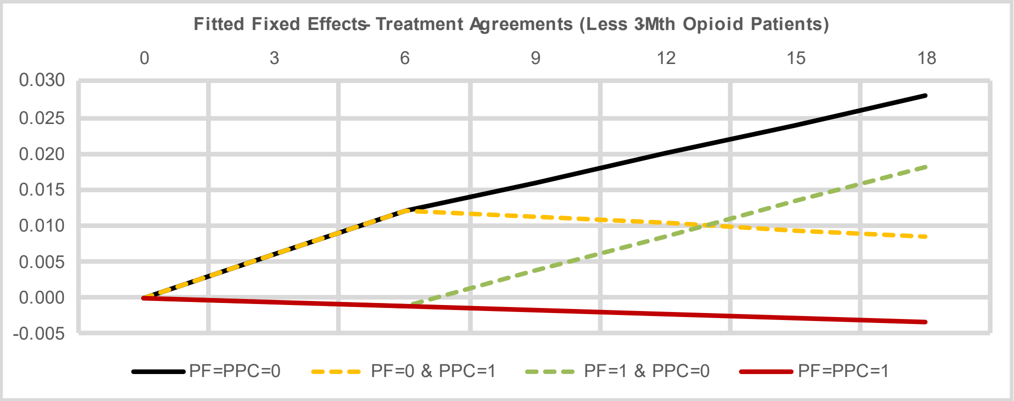

© 2024 Quanbeck A et al. *JAMA Network Open*.

Treatment Agreements (Less 3-Mth Opioid Patients)

Study Month	Estimated Fixed Effect Curves				Differences		
	PF=PPC=0	PF=0 & PPC=1	PF=1 & PPC=0	PF=PPC=1	PF=0 & PPC=1	PF=1 & PPC=0	PF=PPC=1 minus
0	0.000	0.000	0.000	0.000	0.000	0.000	0.000
3	0.006	0.006	-0.001	-0.001	0.000	-0.007	-0.007
6	0.012	0.012	-0.001	-0.001	0.000	-0.013	-0.013
9	0.016	0.011	0.004	-0.002	-0.005	-0.012	-0.018
12	0.020	0.010	0.009	-0.002	-0.010	-0.011	-0.022
15	0.024	0.009	0.013	-0.003	-0.015	-0.011	-0.027
18	0.028	0.009	0.018	-0.003	-0.019	-0.010	-0.031
**Std Dev of Estimated Values**							
0	0.000	0.000	0.000	0.000	0.000	0.000	0.000
3	0.003	0.003	0.003	0.003	0.000	0.004	0.004
6	0.006	0.006	0.006	0.006	0.000	0.008	0.008
9	0.006	0.006	0.006	0.006	0.003	0.008	0.008
12	0.007	0.007	0.007	0.007	0.007	0.010	0.010
15	0.009	0.009	0.009	0.009	0.010	0.012	0.012
18	0.011	0.011	0.011	0.011	0.014	0.015	0.015
**t-ratios of Estimated Values**							
0							
3	1.90	1.90	-0.20	-0.20		-1.60	-1.60
6	1.90	1.90	-0.20	-0.20		-1.60	-1.60
9	2.52	1.77	0.60	-0.29	-1.42	-1.49	-2.14
12	2.73	1.43	1.21	-0.32	-1.42	-1.19	-2.31
15	2.70	1.07	1.55	-0.33	-1.42	-0.90	-2.25
18	2.59	0.80	1.74	-0.32	-1.42	-0.67	-2.14

Covariance Parameter Estimates

Cov Parm	Subject	Estimate
Intercept	Clinic	0.000492
UN(1,1)	PCP	0.002421
UN(2,1)	PCP	-0.00014
UN(2,2)	PCP	0.000046
UN(3,1)	PCP	0.000044
UN(3,2)	PCP	-0.00004
UN(3,3)	PCP	0.000053
Residual		0.03334

Std Dev & Correlation

Clinic	0.0222		
PCP	0.0492		
	-42.0%	0.0068	
	12.3%	-81.0%	0.0073
Residual	0.1826		

© 2024 Quanbeck A et al. *JAMA Network Open*.

Solution for Fixed Effects						
Effect	System	Estimate	Standard Error	DF	t Value	Pr > |t|
System	Bellin	0.08513	0.008561	786	9.94	<.0001
System	UW Health	0.02178	0.008326	786	2.62	0.0091
clinic_size		0	0	786	-Infty	<.0001
t7		0.001989	0.001044	249	1.90	0.0580
PF_t7		-0.00219	0.001370	786	-1.60	0.1096
t13		-0.00065	0.001442	201	-0.45	0.6530
PF_t13		0.002470	0.001945	786	1.27	0.2046
PPC_t13		-0.00162	0.001143	786	-1.42	0.1560
PFandPPC_t13		-0.00018	0.001591	786	-0.11	0.9123

Estimates					
Label	Estimate	Standard Error	DF	t Value	Pr > |t|
NOINT Chg	0.02801	0.01077	786	2.60	0.0095
PF Only Chg	0.01816	0.01037	786	1.75	0.0805
PPC Only Chg	0.008536	0.01062	786	0.80	0.4219
PF & PPC Chg	-0.00342	0.01053	786	-0.32	0.7458
PF Only vs NOINT	-0.00985	0.01450	786	-0.68	0.4973
PPC Only vs NOINT	-0.01947	0.01371	786	-1.42	0.1560
PF Only vs PPC Only 1	0.009621	0.01441	786	0.67	0.5045
PF Only vs PPC Only 2	0.02278	0.01372	786	1.66	0.0973
PF & PPC Interaction	-0.00210	0.01909	786	-0.11	0.9123
PF & PPC vs NOINT	-0.03142	0.01463	786	-2.15	0.0320

© 2024 Quanbeck A et al. *JAMA Network Open*.

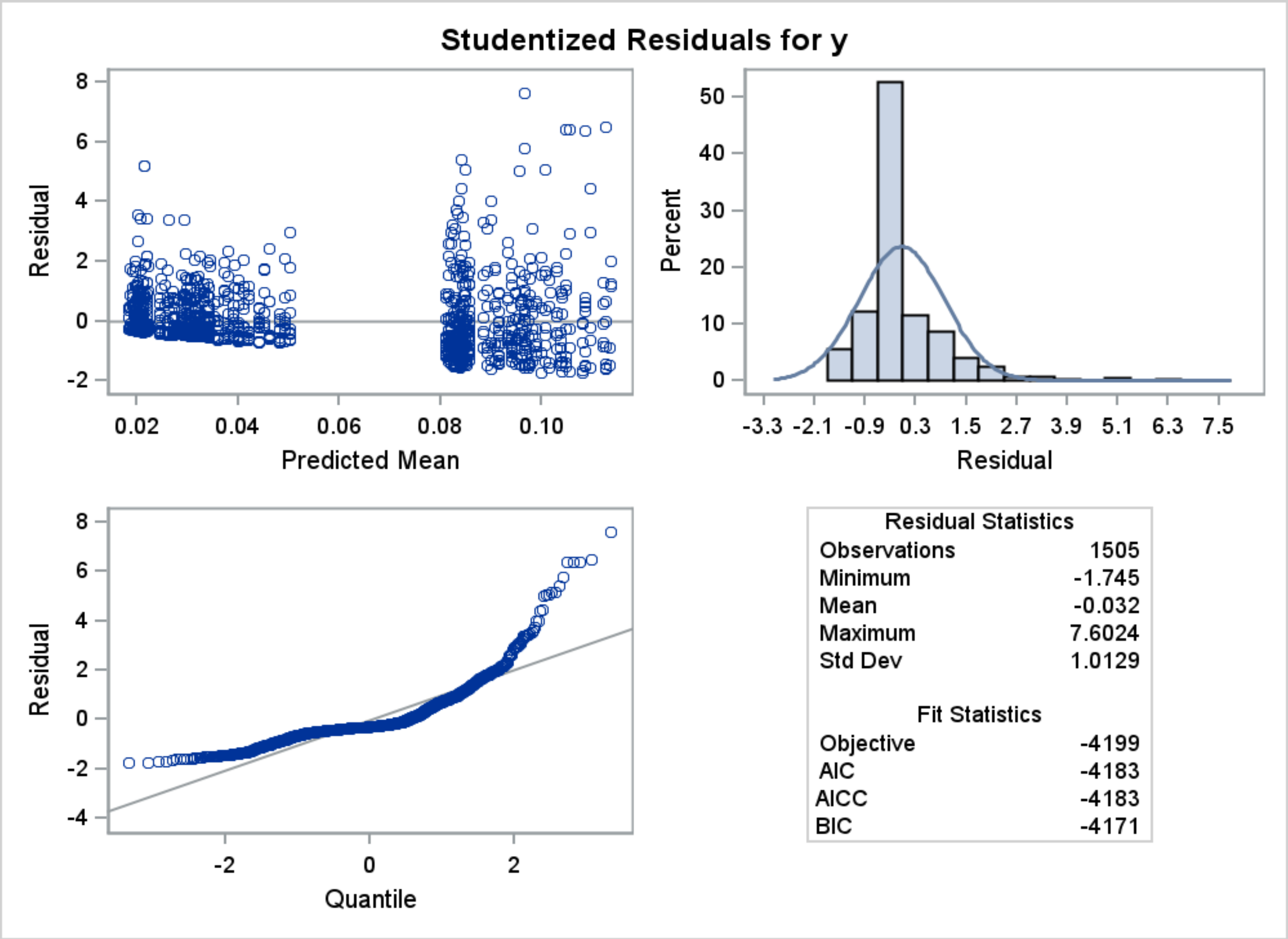

© 2024 Quanbeck A et al. *JAMA Network Open*.

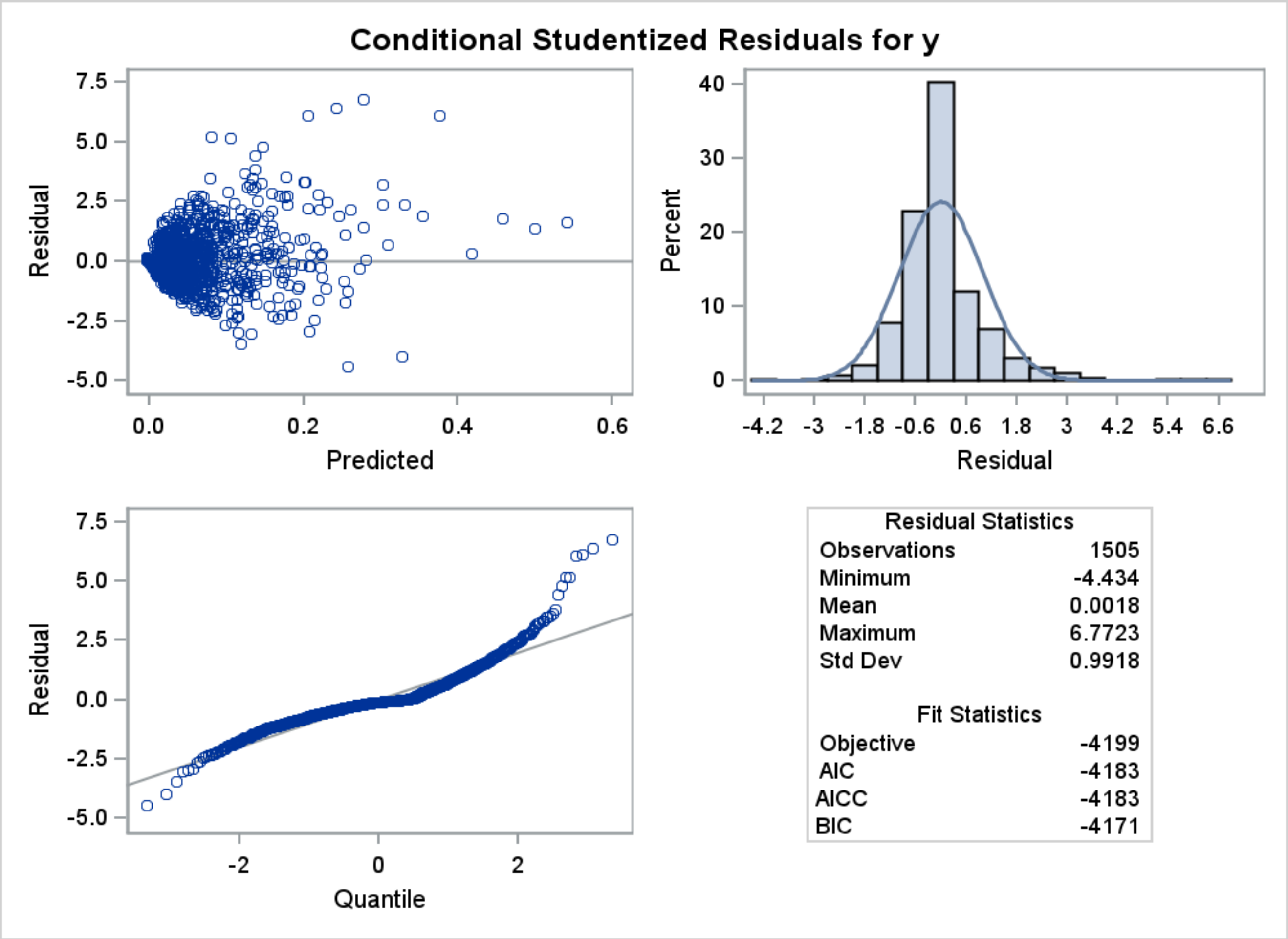

Appendix C6 – Percent of Opioid Patients with PHQ-9 Depression Screen in Last 12 Months (Less than 3-Mth Opioid Patients)

© 2024 Quanbeck A et al. *JAMA Network Open*.

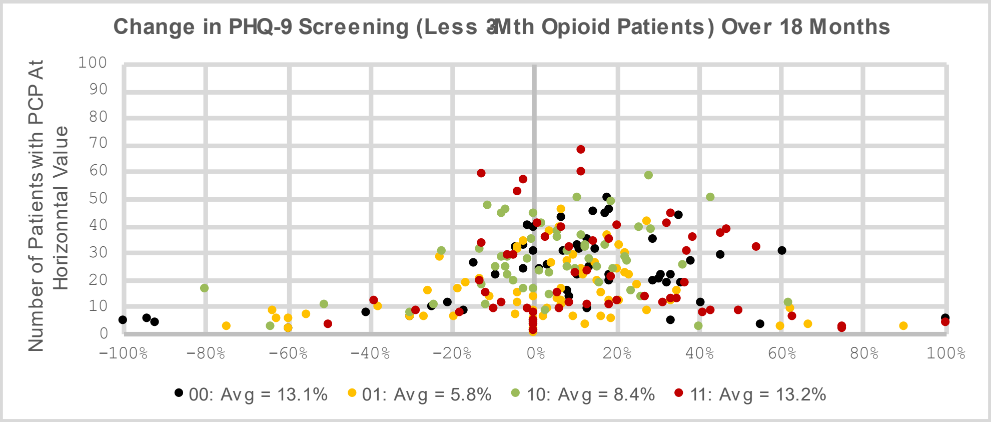

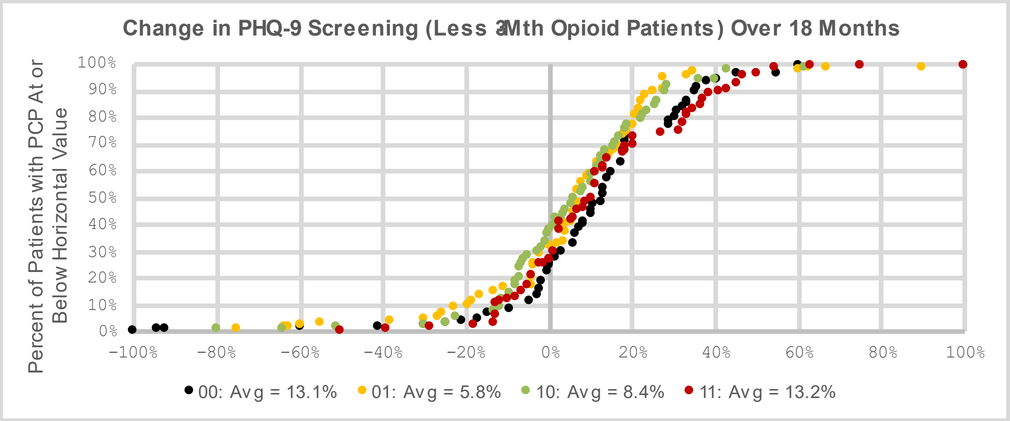

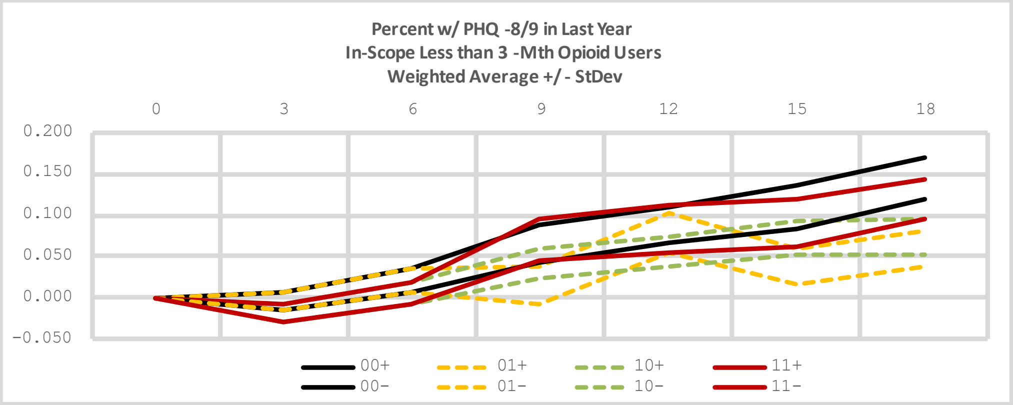

© 2024 Quanbeck A et al. *JAMA Network Open*.

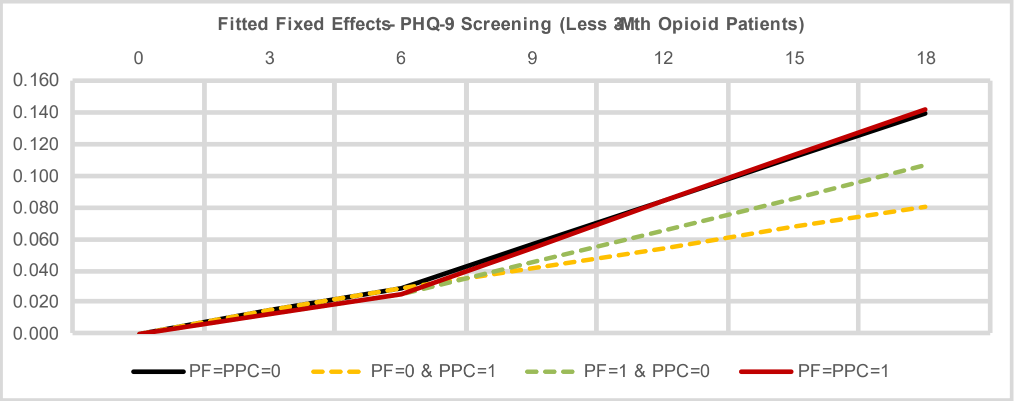

© 2024 Quanbeck A et al. *JAMA Network Open*.

PHQ-9 Screening (Less 3-Mth Opioid Patients)							
Study Month	Estimated Fixed Effect Curves				Differences		
	PF=PPC=0	PF=0 & PPC=1	PF=1 & PPC=0	PF=PPC=1	PF=0 & PPC=1	PF=1 & PPC=0	PF=PPC=1 minus
0	0.000	0.000	0.000	0.000	0.000	0.000	0.000
3	0.014	0.014	0.012	0.012	0.000	-0.002	-0.002
6	0.028	0.028	0.025	0.025	0.000	-0.004	-0.004
9	0.056	0.041	0.045	0.054	-0.015	-0.011	-0.002
12	0.084	0.054	0.066	0.084	-0.030	-0.019	0.000
15	0.112	0.067	0.086	0.113	-0.045	-0.026	0.001
18	0.140	0.080	0.106	0.143	-0.059	-0.033	0.003
**Std Dev of Estimated Values**							
0	0.000	0.000	0.000	0.000	0.000	0.000	0.000
3	0.007	0.007	0.006	0.006	0.000	0.009	0.009
6	0.014	0.014	0.013	0.013	0.000	0.018	0.018
9	0.014	0.014	0.014	0.013	0.007	0.019	0.019
12	0.015	0.015	0.016	0.016	0.013	0.022	0.022
15	0.018	0.018	0.019	0.020	0.020	0.026	0.026
18	0.022	0.022	0.023	0.024	0.026	0.031	0.032
**t-ratios of Estimated Values**							
0							
3	2.08	2.08	1.93	1.93		-0.21	-0.21
6	2.08	2.08	1.93	1.93		-0.21	-0.21
9	4.07	3.03	3.34	4.02	-2.25	-0.59	-0.11
12	5.44	3.57	4.13	5.25	-2.25	-0.86	-0.02
15	6.17	3.74	4.45	5.80	-2.25	-0.99	0.05
18	6.49	3.74	4.56	6.02	-2.25	-1.06	0.10

© 2024 Quanbeck A et al. *JAMA Network Open*.

Covariance Parameter Estimates				Std Dev & Correlation		
Cov Parm	Subject	Estimate				
Intercept	Clinic	0.007275	Clinic	0.0853		
UN(1,1)	PCP	0.009080				
UN(2,1)	PCP	-0.00071				
UN(2,2)	PCP	0.000189	PCP	0.0953		
UN(3,1)	PCP	0.000550		-54.2%	0.0137	
UN(3,2)	PCP	-0.00015		47.1%	-89.1%	0.0122
UN(3,3)	PCP	0.000150				
Residual		0.1681	Residual	0.4100		

Solution for Fixed Effects

Effect	System	Estimate	Standard Error	DF	t Value	Pr > |t|
System	Bellin	0.2964	0.02536	786	11.68	<.0001
System	UW Health	0.1947	0.02676	786	7.28	<.0001
clinic_size		0	0	786	-0.55	0.5803
t7		0.004743	0.002284	249	2.08	0.0389
PF_t7		-0.00063	0.003043	786	-0.21	0.8373
t13		0.004535	0.002941	201	1.54	0.1247
PF_t13		-0.00183	0.003950	786	-0.46	0.6433
PPC_t13		-0.00495	0.002203	786	-2.25	0.0250
PFandPPC_t13		0.007976	0.003035	786	2.63	0.0087

© 2024 Quanbeck A et al. *JAMA Network Open*.

Estimates					
**Label**	**Estimate**	**Standard Error**	**DF**	**t Value**	**Pr > |t|**
**NOINT Chg**	0.1398	0.02150	786	6.50	<.0001
**PF Only Chg**	0.1066	0.02049	786	5.20	<.0001
**PPC Only Chg**	0.08042	0.02148	786	3.74	0.0002
**PF & PPC Chg**	0.1429	0.02086	786	6.85	<.0001
**PF Only vs NOINT**	-0.03321	0.02933	786	-1.13	0.2579
**PPC Only vs NOINT**	-0.05937	0.02643	786	-2.25	0.0250
**PF Only vs PPC Only 1**	0.02616	0.02932	786	0.89	0.3726
**PF Only vs PPC Only 2**	0.02991	0.02606	786	1.15	0.2515
**PF & PPC Interaction**	0.09571	0.03642	786	2.63	0.0087
**PF & PPC vs NOINT**	0.003133	0.02959	786	0.11	0.9157

© 2024 Quanbeck A et al. *JAMA Network Open*.

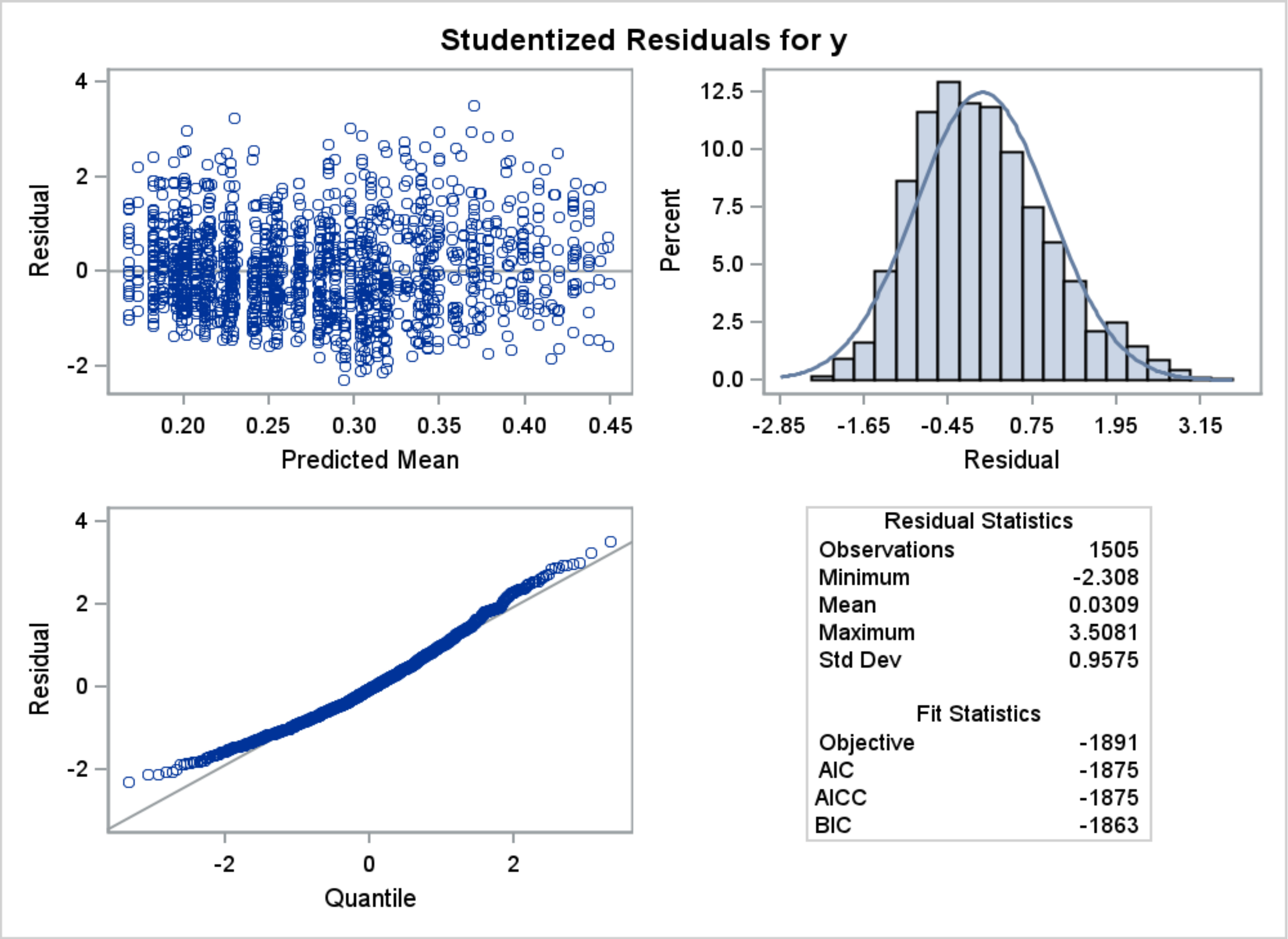

© 2024 Quanbeck A et al. *JAMA Network Open*.

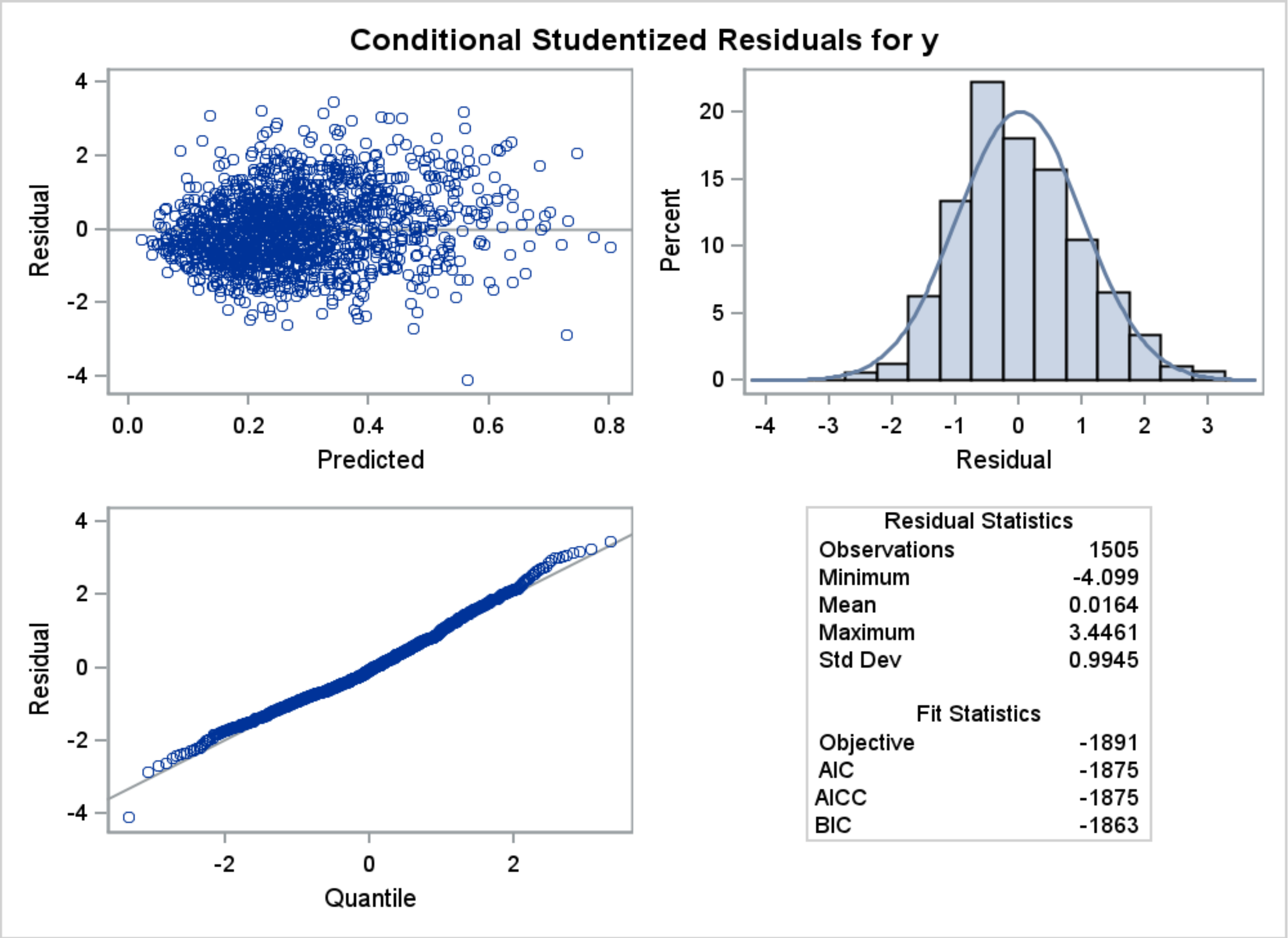

Appendix C7 – Percent of Opioid Patients with PEG-3 Pain Screen in Last 12 Months (Less than 3-Mth Opioid Patients)

© 2024 Quanbeck A et al. *JAMA Network Open*.

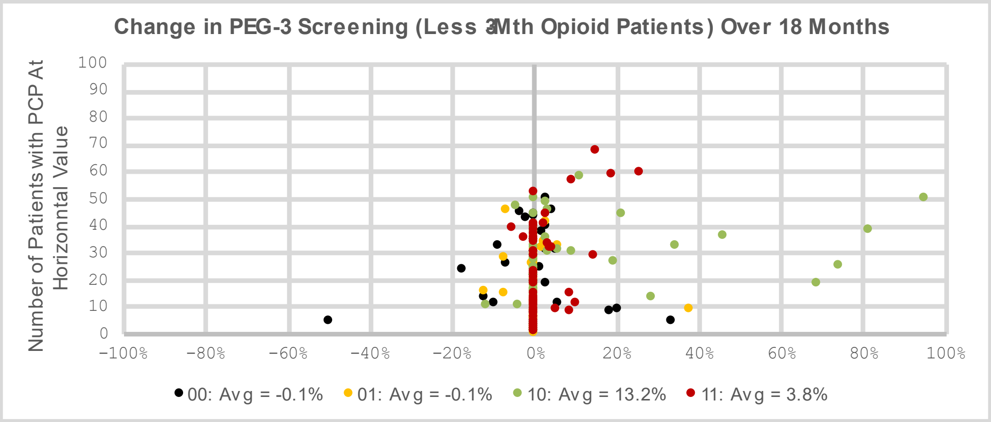

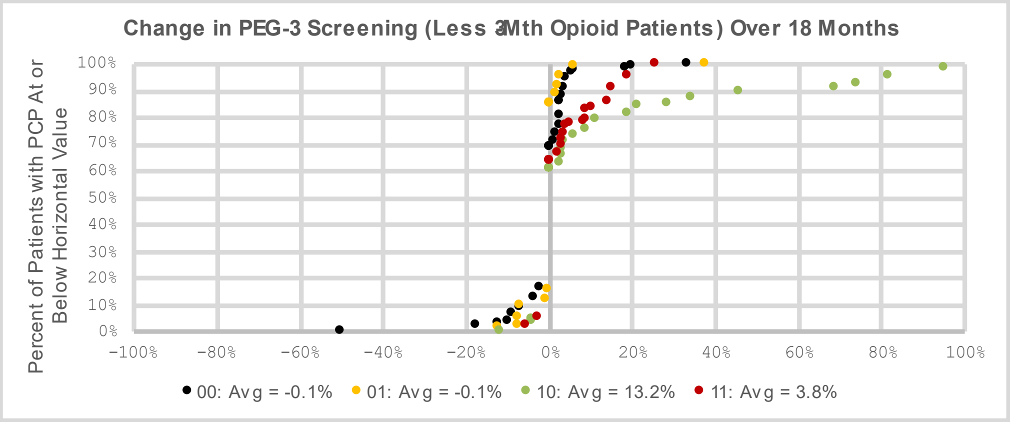

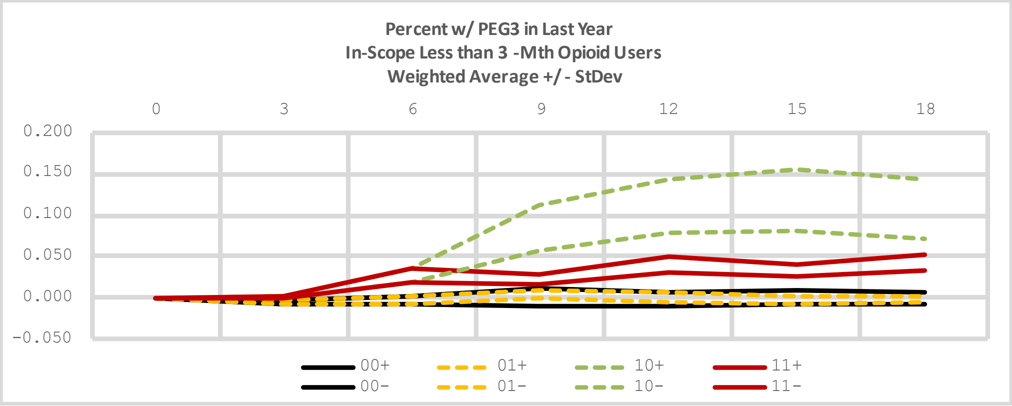

© 2024 Quanbeck A et al. *JAMA Network Open*.

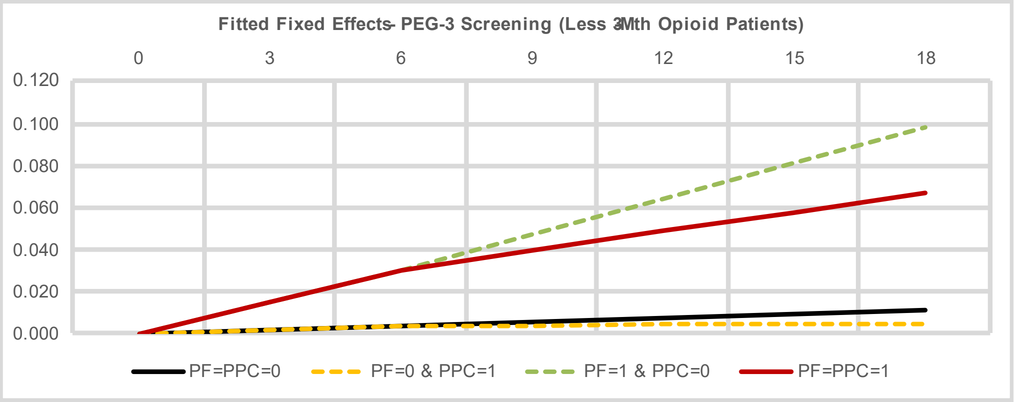

© 2024 Quanbeck A et al. *JAMA Network Open*.

PEG-3 Screening (Less 3-Mth Opioid Patients)

Study Month	Estimated Fixed Effect Curves				Differences		
	PF=PPC=0	PF=0 & PPC=1	PF=1 & PPC=0	PF=PPC=1	PF=0 & PPC=1	PF=1 & PPC=0	PF=PPC=1 minus
0	0.000	0.000	0.000	0.000	0.000	0.000	0.000
3	0.002	0.002	0.015	0.015	0.000	0.013	0.013
6	0.004	0.004	0.030	0.030	0.000	0.027	0.027
9	0.006	0.004	0.047	0.039	-0.002	0.042	0.034
12	0.008	0.004	0.064	0.049	-0.004	0.057	0.041
15	0.009	0.004	0.081	0.058	-0.005	0.072	0.048
18	0.011	0.004	0.098	0.067	-0.007	0.087	0.056
**Std Dev of Estimated Values**							
0	0.000	0.000	0.000	0.000	0.000	0.000	0.000
3	0.004	0.004	0.004	0.004	0.000	0.005	0.005
6	0.008	0.008	0.008	0.008	0.000	0.011	0.011
9	0.010	0.010	0.010	0.010	0.004	0.013	0.013
12	0.012	0.012	0.012	0.012	0.009	0.017	0.016
15	0.015	0.015	0.015	0.014	0.013	0.020	0.020
18	0.018	0.017	0.018	0.017	0.018	0.024	0.024
**t-ratios of Estimated Values**							
0							
3	0.47	0.47	3.87	3.87		2.48	2.48
6	0.47	0.47	3.87	3.87		2.48	2.48
9	0.58	0.40	4.91	4.15	-0.40	3.16	2.58
12	0.62	0.34	5.35	4.11	-0.40	3.43	2.50
15	0.63	0.28	5.53	3.99	-0.40	3.52	2.38
18	0.63	0.24	5.59	3.86	-0.40	3.54	2.28

Covariance Parameter Estimates				Std Dev & Correlation		
**Cov Parm**	**Subject**	**Estimate**				
**Intercept**	Clinic	0.000232	Clinic	0.0152		
**UN(1,1)**	PCP	0.000566				
**UN(2,1)**	PCP	-0.00014				
**UN(2,2)**	PCP	0.000155	PCP	0.0238		
**UN(3,1)**	PCP	0.000036		-47.3%	0.0124	
**UN(3,2)**	PCP	-0.00008		20.4%	-86.6%	0.0074
**UN(3,3)**	PCP	0.000055				
**Residual**		0.02478	Residual	0.1574		

© 2024 Quanbeck A et al. *JAMA Network Open*.

Solution for Fixed Effects						
Effect	System	Estimate	Standard Error	DF	t Value	Pr > |t|
System	Bellin	0.000608	0.005766	786	0.11	0.9161
System	UW Health	0.007940	0.005777	786	1.37	0.1697
clinic_size		0	0	786	Infty	<.0001
t7		0.000622	0.001325	249	0.47	0.6392
PF_t7		0.004427	0.001785	786	2.48	0.0133
t13		0.000011	0.001363	201	0.01	0.9938
PF_t13		0.000586	0.001846	786	0.32	0.7511
PPC_t13		-0.00059	0.001462	786	-0.40	0.6890
PFandPPC_t13		-0.00201	0.002012	786	-1.00	0.3186

Estimates					
Label	Estimate	Standard Error	DF	t Value	Pr > |t|
NOINT Chg	0.01133	0.01784	786	0.64	0.5256
PF Only Chg	0.09805	0.01748	786	5.61	<.0001
PPC Only Chg	0.004304	0.01749	786	0.25	0.8057
PF & PPC Chg	0.06693	0.01731	786	3.87	0.0001
PF Only vs NOINT	0.08672	0.02444	786	3.55	0.0004
PPC Only vs NOINT	-0.00702	0.01754	786	-0.40	0.6890
PF Only vs PPC Only 1	0.09374	0.02424	786	3.87	0.0001
PF Only vs PPC Only 2	0.06718	0.01828	786	3.67	0.0003
PF & PPC Interaction	-0.02410	0.02415	786	-1.00	0.3186
PF & PPC vs NOINT	0.05560	0.02434	786	2.28	0.0226

© 2024 Quanbeck A et al. *JAMA Network Open*.

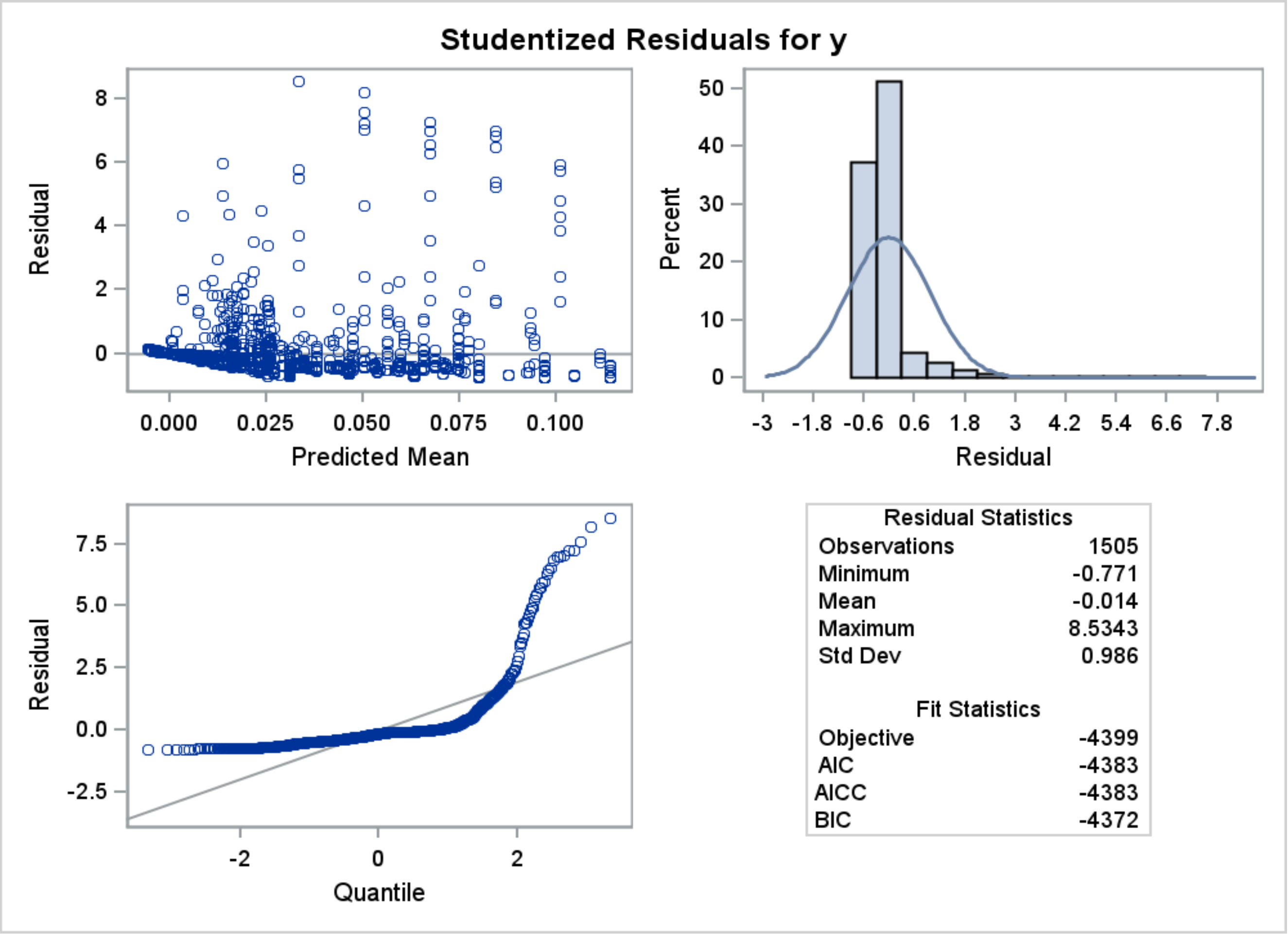

© 2024 Quanbeck A et al. *JAMA Network Open*.

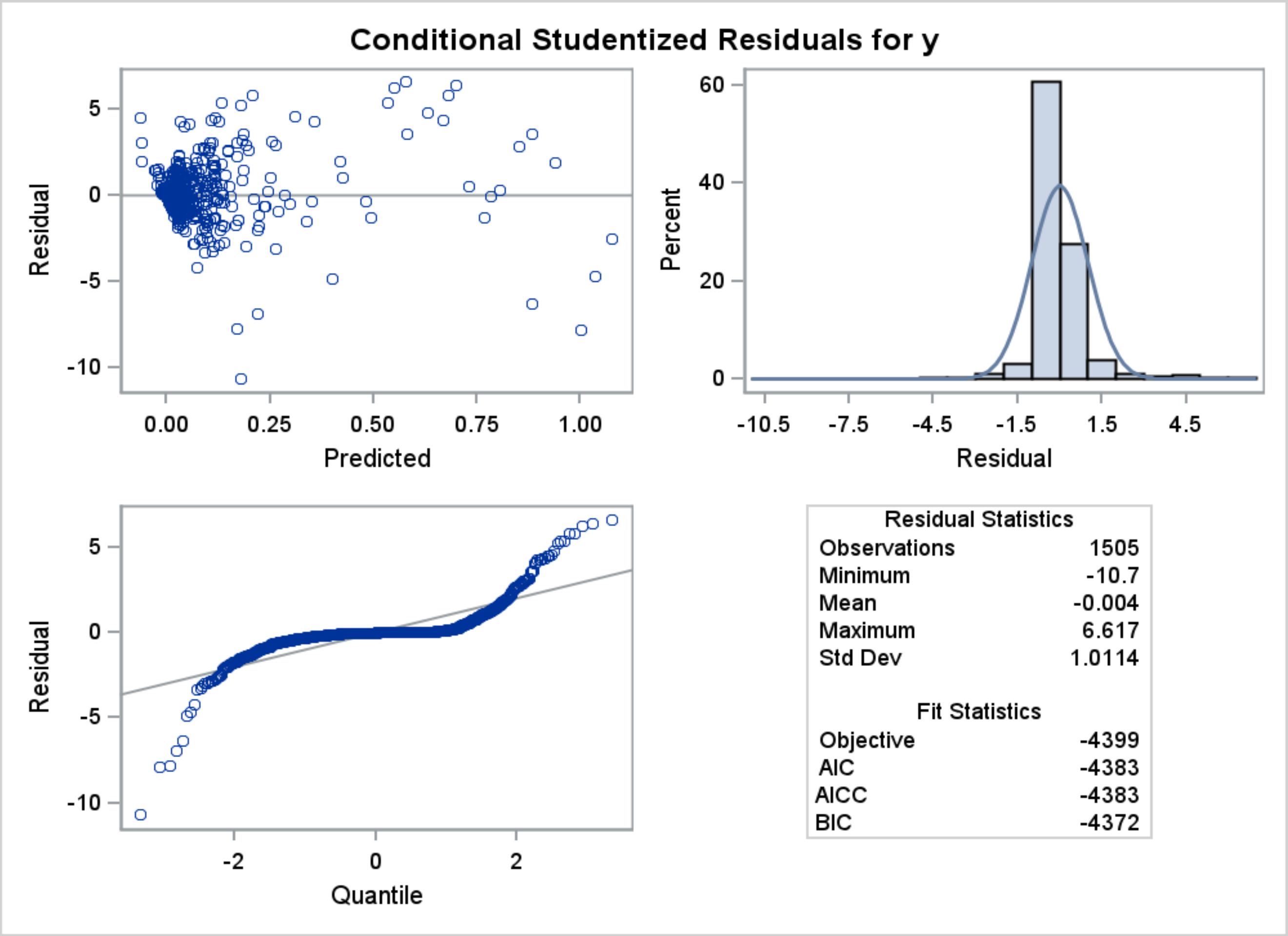

© 2024 Quanbeck A et al. *JAMA Network Open*.

Specification of the Balanced Opioid Initiative Implementation Strategies:

- Adoption decision/preparation activities, which introduce the project and research team to the staff of health systems and clinics and help prepare the health systems and clinics for undertaking the project.
- Educational meetings, which consists of didactic sessions devoted to explain the opioid prescribing guidelines.
- Audit and feedback, which involves clinicians monitoring their opioid prescribing practices using monthly feedback reports.
- Practice facilitation, which focuses on improving clinic workflows related to opioid prescribing.
- Prescriber peer consulting, in which physician experts in opioid prescribing consult with prescribers about difficult panels and cases.

**Temporality* in the table below, fifth column, shows the months each strategy was planned. Due to the COVID-19 pandemic, many of these dates were pushed back.

The table below is based on Proctor's recommendations for specifying implementation strategies.[2] Strategies named in the first column come from Powell's ERIC project unless otherwise noted.[3]

ADOPTION DECISION/PREPARATION

Strategy	Actor (stakeholder who delivers implementation strategy)	Actions (of research team health systems, clinicians, and others)	Target of the action	Temporality*	Dose or intensity	Targeted implementation outcomes	Justification

© 2024 Quanbeck A et al. *JAMA Network Open*.

Strategy	Actor (stakeholder who delivers implementation strategy)	Actions (of research team health systems, clinicians, and others)	Target of the action	Temporality*	Dose or intensity	Targeted implementation outcomes	Justification
**1**-Develop academic partnerships (between an academic unit and a health system)	Principal Investigator (PI) and other members of the research team	Research team: As part of developing the proposal for the study, the PI contacts health system leaders to explain the project and ask for letter of support. After funding is obtained, research team initiates regular communication with health systems leaders to engage their active participation.	Engagement of health system leaders. If they want to participate, then engagement of clinic leaders and staff.<br><br>*Note: We need to make sure*	Initial contact during the writing of the proposal. Meetings and site visits take place in Months 1-15, with frequency determined by research team and health system and clinic personnel on an as-needed basis.	Meetings are one hour. Site visits are 3-4 hours.	Increase the acceptability and adoption of opioid prescribing guidelines	Evidence suggests that the engagement of organizational leaders is critical to the success of change effort. References: Damschroder et al, CFIR, 2009; Aarons et al, Evidence-based
		Health systems: Health systems leaders write letter of support. After funding is obtained, leaders take part in meetings and other communications to move the study forward in a way that benefits the health system.	*we're getting green lights from people in a position to influence and/or take part in implementation.*	Email and phone contact also take place as necessary.			practice implem, 2011.

© 2024 Quanbeck A et al. *JAMA Network Open*.

Strategy	Actor (stakeholder who delivers implementation strategy)	Actions (of research team health systems, clinicians, and others)	Target of the action	Temporality*	Dose or intensity	Targeted implementation outcomes	Justification
**2**-Develop and organize quality monitoring systems	PI and other research team members; each health system's designated information technology (IT) coordinator	PI calls initial meeting of research team, each health system's designated IT leader, Wisconsin Collaborative for Healthcare Quality (WCHQ) (which collects data from health systems), and the UW Health Innovation Program (HIP) (which de-identifies data and provides data sets to researchers). Subsequent tasks include defining and operationalizing outcomes, scheduling any necessary programming, testing the	Ease of producing and effectiveness of monthly reports for clinicians and ease and accuracy of data collection for the study.	Initial quality monitoring system is constructed during months 3-12 and revised during months 13-15.	Initial and follow-up monthly meetings are usually one hour.	Increase acceptability, feasibility, and fidelity.	Agreeing on outcome definitions and data collection methods gives researchers, clinicians, and health systems staff a clear, agreed-upon set of goals.
		data collection plan, and revising the data collection plan and methods.					

EDUCATIONAL MEETINGS (EM)

© 2024 Quanbeck A et al. *JAMA Network Open*.

Strategy	Actor (stakeholder who delivers implementation strategy)	Actions (of research team health systems, clinicians, and others)	Target of the action	Temporality*	Dose or intensity	Targeted implementation outcomes	Justification
**3**-Develop educational materials	Research team	Research team develops presentation and training materials for regionally hosted, in-person training sessions. (Note: Due to COVID, sessions were all held virtually.)	Engagement, knowledge, and skills of clinic prescribers and other staff members	Materials (Powerpoint presentation, handouts) are developed in months 14-15. Training sessions take place starting in month 16.	6 quarterly meetings spread over 18 months.	Increase adoption and feasibility.	Prescribers may fall short of guideline concordance because they don't know what the guidelines say or lack the skills to implement guideline-concordant care. Increased knowledge and skills may suffice to improve guideline concordance.

Strategy	Actor (stakeholder who delivers implementation strategy)	Actions (of research team health systems, clinicians, and others)	Target of the action	Temporality*	Dose or intensity	Targeted implementation outcomes	Justification

© 2024 Quanbeck A et al. *JAMA Network Open*.

**4-** Conduct educational meetings, and<br>**5-** Distribute educational materials	Physician experts in opioid prescribing	In regionally hosted, in-person meeting, physician experts describe clinical guidelines for opioid prescribing, how workflows can affect outcomes, and clinical outcomes and issues (e.g., how to balance pain and function during dose reduction). Give attendees handouts, links, and other information. Elicit engagement and enthusiasm.	Engagement, knowledge, and skills of prescribers and other staff members directly involved in workflows related to opioid prescribing	Starting in month 16	One hour	Increase adoption, feasibility, and fidelity.	Same as for Strategy 3 above.

AUDIT AND FEEDBACK (AF)

Strategy	Actor (stakeholder who delivers implementation strategy)	Actions (of research team health systems, clinicians, and others)	Target of the action	Temporality*	Dose or intensity	Targeted implementation outcomes	Justification
**6-** Audit and provide feedback<br>**7-** Facilitate relay of	Designated IT coordinator at each health system	Work with prescribers to ensure they have reports that help them monitor their opioid prescribing.	Knowledge and skills mainly of prescribers, but consultation on using the EHR and the use of	Clinicians will receive monthly feedback reports during Months 16-36. EHR consultation will	One report monthly in months 16-36. Consultation	Increase feasibility and fidelity.	Prescribers need feedback on outcome data to assess their own performance because self-assessment and

© 2024 Quanbeck A et al. *JAMA Network Open*.

clinical data to providers<br><br>**8**-Conduct educational outreach visits (with providers)		This may involve programming or ensuring that programming gets done. Consult with prescribers and other clinicians on how to use the electronic health record (EHR) to monitor opioid prescribing and ensure that clinicians receive monthly reports. Consultation may be offered on the phone, in email, or during visits to clinics.	feedback reports might also target clinic staff members who work with patients on long-term opioid therapy.	be offered as needed during Months 16-36.	takes place as needed.		correction are essential to making and sustaining the changes. In Deming's quality improvement model, this falls under "how will you know if a change is an improvement?"<br><br>The EHR has tools for monitoring opioid prescribing. Knowing how to use these tools may ease prescribers' path to guideline concordance.

PRACTICE FACILITATION (PF)

© 2024 Quanbeck A et al. *JAMA Network Open*.

Strategy	Actor (stakeholder who delivers implementation strategy)	Actions (of research team health systems, clinicians, and others)	Target of the action	Temporality*	Dose or intensity	Targeted implementation outcomes	Justification
**9**-Recruit, designate, and train for leadership for the change effort at each clinic	Practice facilitator	Practice facilitator works with clinic medical director to identify a physician change leader for the clinic, who in turn selects about five other staff members to form a change team.	Engagement of respected clinic staff members whose work involves opioid prescriptions. This could include a receptionist who takes a call from a patient wanting a refill, a medical assistant who checks for a recent treatment agreement, a prescriber, and others.	Month 16	Will vary by clinic.	Increase acceptability, adoption, and feasibility.	Forming a team within a clinic to carry out the project locates responsibility for the work in the clinic and gives change team members colleagues in doing the work.

© 2024 Quanbeck A et al. *JAMA Network Open*.

Strategy	Actor (stakeholder who delivers implementation strategy)	Actions (of research team health systems, clinicians, and others)	Target of the action	Temporality*	Dose or intensity	Targeted implementation outcomes	Justification
**10-**Organize clinician implementation team meeting	Practice facilitator	Practice facilitator arranges a site visit.	Attendance, engagement, knowledge, and skills of change team members.	Month 19	TBD	Increase acceptability, adoption, and feasibility.	The implementation team (the change team) focuses on clinic processes related to opioid prescribing, based on Deming's argument that problems most often result from poor systems (rather than the actions of individuals). The site visit uncovers systems problems
							(bottlenecks, redundancy, etc.) in opioid prescribing and introduces tools for addressing them.

© 2024 Quanbeck A et al. *JAMA Network Open*.

Strategy	Actor (stakeholder who delivers implementation strategy)	Actions (of research team health systems, clinicians, and others)	Target of the action	Temporality*	Dose or intensity	Targeted implementation outcomes	Justification
**11-** Promote adaptability<br>**12-** Provide local technical assistance<br>**13-** Tailor strategies<br>**14-** Purposely reexamine the implementation	Practice facilitator	Practice facilitator teaches change team members to use tools such as these:<br>• Walkthroughs<br>• Flowcharting<br>• Nominal group technique<br>• Plan-Do-Study-Act cycles	Knowledge and skills of change team members	Months 19-36	On-site visit is described above. Thereafter, one-hour telecom- ferences or videocon- ferences take place.	Increase adoption, feasibility, and fidelity.	Clinicians may not understand how to fix systems problems. The systems engineering tools address these problems in a practical way.
**15**-Promote network weaving (building on working relationships and networks	Practice facilitator	Practice facilitator organizes a videoconference so members of different change teams can learn	Engagement, knowledge, and skills of change team members	Month 23	One-hour	Increase adoption, fidelity, penetration, and sustainability.	A videoconference makes known the identity of other change teams and the work they've done known. This may increase engagement,

© 2024 Quanbeck A et al. *JAMA Network Open*.

within and outside clinics)		from one another.					suggest new ideas or methods to try, and increase dissemination across the health system.
**16-**Stage implementation scale up	Practice facilitator	Practice facilitator works with clinic change team members to plan for dissemination of improved workflows across the clinic	Engagement, knowledge, and skills of change team members + all clinic staff	Months 23-24	Part of monthly practice facilitation meetings	Increase adoption, penetration, and sustainability.	Improvements identified by change teams likely require planned dissemination efforts to reach all clinic staff.

PRESCRIBER PEER CONSULTING (PPC)

**Strategy**	**Actor (stakeholder who delivers implementation strategy)**	**Actions (of research team health systems, clinicians, and others)**	**Target of the action**	**Temporality***	**Dose or intensity**	**Targeted implementation outcomes**	**Justification**
17-Facilitation (as the ERIC study defines it, i.e., interactive problem	Prescriber peer consultants	Research team members set up one-hour consultations with individual prescribers or small groups of prescribers and instruct prescribers to bring challenging cases to the	Knowledge and skills of prescribers	Quarterly in Months 25-36	One hour per teleconference or video conference	Increase feasibility and fidelity.	Helping prescribers think through specific challenging cases may improve treatment for

© 2024 Quanbeck A et al. *JAMA Network Open*.

Strategy	Actor (stakeholder who delivers implementation strategy)	Actions (of research team health systems, clinicians, and others)	Target of the action	Temporality*	Dose or intensity	Targeted implementation outcomes	Justification
solving and support)		session. Physician peer consultants conduct the consultations via teleconference or video-conference. Clinic prescribers describe difficult cases, and peer coaches help the prescriber(s) think through how to address the difficulty(ies).					specific patients and generalize to the treatment of other challenging patients.

eReferences.

1. Aarons GA, Hurlburt M, Horwitz SM. Advancing a conceptual model of evidence-based practice implementation in public service sectors. Adm Policy Ment Health. 2011;38(1):4-23.

2. Proctor EK, Powell BJ, McMillen JC. Implementation strategies: commendations for specifying and reporting. Implemt Sci. 2013;8:139.

3. Powell BJ, Waltz TJ, Chinman MJ et al. A refined compilation of implementation strategies: results from the Expert Recommendations for Implementing Change (ERIC) project. Implemt Sci. 2015;10:21.

© 2024 Quanbeck A et al. *JAMA Network Open*.
