# Supplementary material for: Strategies to Deimplement Opioid Prescribing in Primary Care: A Cluster Randomized Clinical Trial
Source: JAMA Netw Open. 2024 Oct 10;7(10):e2438325. doi: 10.1001/jamanetworkopen.2024.38325 (PMC11581553; doi:10.1001/jamanetworkopen.2024.38325)
Supplement: Supplement 3. — Data Sharing Statement [file jamanetwopen-e2438325-s003.pdf]

Data Sharing Statement

Quanbeck. Strategies to Deimplement Opioid Prescribing in Primary Care. *JAMA Netw Open*. Published October 10, 2024. doi:10.1001/jamanetworkopen.2024.38325

Data
Data available: Yes
Data types: Deidentified participant data, Data dictionary
How to access data: Access to study data may be requested by contacting corresponding author Andrew Quanbeck at andrew.quanbeck@fammed.wisc.edu.
When available: With publication

Supporting Documents
Document types: Statistical/analytic code, Other (please specify)
Additional Information: Implementation strategy specification.
How to access documents: Included as supplementary online material with publication.
When available: With publication

Additional Information
Who can access the data: The data will be made available to researchers whose proposed use of the data has been approved via a data sharing agreement with the Health Innovation Program at the University of Wisconsin.
Types of analyses: For a specified purpose.
Mechanisms of data availability: with a signed data access agreement
Any additional restrictions: Researchers will need to complete required onboarding and training on data security required by the Health Innovation Program at the University of Wisconsin.
